# *Cinchona* Organocatalyzed Enantioselective Amination for Quaternized Serines as Tertiary Amides

**DOI:** 10.1021/acs.orglett.4c03650

**Published:** 2024-10-16

**Authors:** Phathutshedzo Masithi, Ashlyn D. Bhana, Gerhard A. Venter, Hong Su, Christopher D. Spicer, Wade F. Petersen, Roger Hunter

**Affiliations:** † Department of Chemistry, University of Cape Town, Rondebosch, Cape Town 7700, South Africa; ‡ Department of Chemistry, University of York, Heslington, York YO10 5DD, U.K.

## Abstract

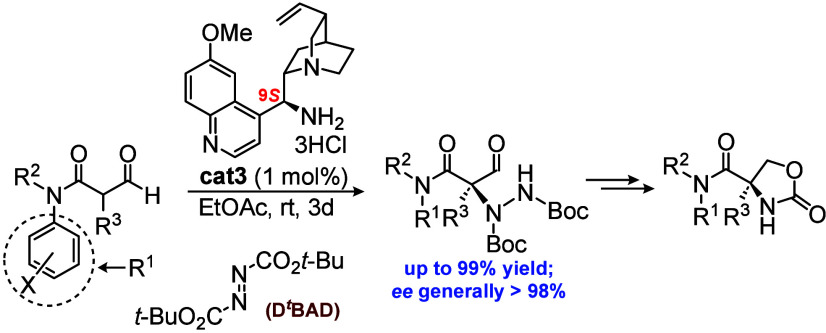

Herein, we describe a *Cinchona*-aminocatalyzed enantioselective α-hydrazination of an α-formyl amide for the production of protected quaternized serines as tertiary amides with *ee*’s of generally >98% and ≤99% yields. The proposed TS model supported by density functional theory calculations involves a quinuclidinium ion Brønsted acid-assisted delivery of D^*t*^BAD, which occurs from the *Re* face of an H-bonded enaminone when using a 9*S*-cinchonamine catalyst, resulting in a hydrazide with the *R*-configuration as determined by X-ray analysis.

Chiral α-tertiary amine (ATA) motifs are ubiquitous in natural products and play an important role both in pharmaceutical and agrochemical drugs.^1^ To date, various stereoselective syntheses of ATAs have been reported that have generated a vast literature.^2^ A very important subclass of ATAs as a primary focus in this work is the chiral α-quaternized serine motif, which appear in many natural products.^3^ A fair number of methods for producing quaternized serines in nonracemic form have been reported but suffer from a mix of moderate *ee*’s and limited scope of the α-R group. For instance, diastereoselective approaches are well-known, with Seebach’s SRS oxazoline methodology,^4^ Davis’ sulfinimine^5^ and Ellman’s sulfinamide^6^ variants being the most prominent exponents. Similarly, a number of enantioselective approaches have also been reported as shown in Figure 1, covering C-alkylation/acylation (Figure 1a)^7^ of both cyclic and acyclic templates, oxidative desymmetrization (Figure 1b)^8^ (enzyme-mediated methods^9^ are also known but not shown in the Scheme) and electrophilic amination (Figure 1c),^10^ the latter being the topic of this communication.

**Figure 1:**
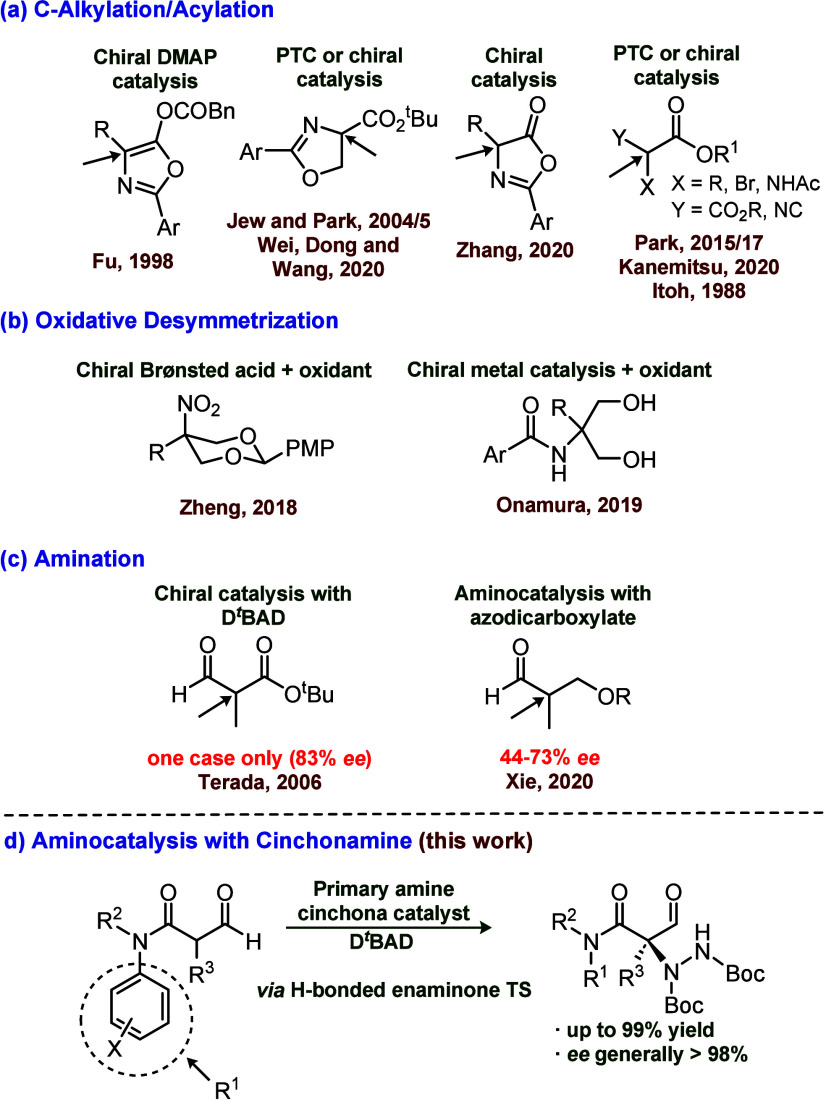
Enantioselective methods for α-quaternized serines.

Enantioselective α-aminations of a 1,3-dicarbonyl template mainly use a β-ketoester^11^ as starting material, which is functionally unsuitable for accessing a serine product. This gap (see Figure 1c) led us to consider an α-formyl amide (see Figure 1d) using an organocatalysis approach with a *Cinchona* catalyst, particularly since a method for accessing the α-formyl amide (*via* Lewis-acid promoted α-formylation of a tertiary amide) had recently become available from our group.^12^

While the formylation failed with Weinreb and secondary amides, it worked well with tertiary amides, allowing facile variation of the α-R group. Regarding amination, the carbamoyl group of the α-formyl amide (Figure 1d) was expected to activate the aldehyde carbonyl toward enaminone formation with the amine organocatalyst. Here, the enaminone was expected to adopt a fixed (*Z*)-configuration due to H-bonding between the NH and the carbamoyl carbonyl oxygen, circumventing the *E/Z*-configurational problem known for simple α-substituted aldehydes.^13^ For the organocatalyst, we also noted a gap in the *Cinchona* repertoire for amination, which for β-ketoesters and related substrates had enjoyed only modest enantioselectivities presumably due to a modest facial control in the amination step involving an ion-pair.^14^ Recently, Guin^15^ has reported on the enantioselective amination of a 1,3-dicarbonyl compound using a chiral azolium catalyst, but in their case the α-carbonyl moiety to the amide was either ester or ketone-based, which for accessing serines suffers from functional group manipulation in the end-game as alluded to previously. Herein, we present a new enantioselective α-amination of α-formyl tertiary amides using a primary amine *Cinchona* catalyst to access chiral α-quaternized serine tertiary amides (Figure 1d) in very high *ee* and good scope.

Model α-formyl amide **2a** was initially chosen for catalyst screening using a range of *Cinchona* alkaloid catalysts in CH_2_Cl_2_ covering a range of aminating agents in which D^*t*^BAD was found to be superior. At room temperature (23 °C), on a 100 mg (0.52 mmol) scale of **2a**, the reaction took 3 days to reach completion at a 1 mol % catalyst loading, with enantioselectivity measured by chiral HPLC. Notably, (8α, 9*S*)-6′-methoxycinchonan-9-amine trihydrochloride, (**cat3**) gave the best *ee* (94%) in high yield (91%) (Figure 2, while a 9-amino catalyst in free amine form (**cat4**) or functionalized as a thiourea derivative (**cat5**) both gave relatively poor ee’s (42% and 38%, respectively; see the Supporting Information), which was considered to be mechanistically significant.

**Figure 2:**
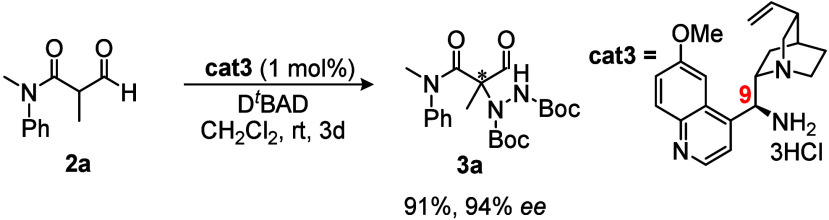
Yield and ee of hydrazination of **2a** with **cat3**.

A follow-up study on the optimization of catalyst loading and solvent using D^*t*^BAD as aminating agent (see the Table on page S4 of the Supporting Information), identified a 1 mol % catalyst loading in EtOAc as solvent for 3 days at room temperature (23 °C) with D^*t*^BAD as hydrazinating agent as optimal, returning an *ee* of 98% and a yield of 88% on a 0.52 mmol scale of **2a** to **3a**. On a 1 grm (5 mmol) scale of α-formyl amide **2a** under the same conditions pleasingly also returned an *ee* of 98% albeit in a slightly lower yield (82%) and longer reaction time (18 days).

The substrate scope was subsequently established varying the groups on nitrogen for tertiary amide synthesis as well as the α-R^3^ group. As shown in Scheme 1 (entries **3b**–3**u**), the method returned excellent *ee*’s (mostly 98% and above) and generally very good yields throughout with R^3^ = the standard methyl group while varying groups on nitrogen (entries **3b**–**3m**).

**Scheme 1:**
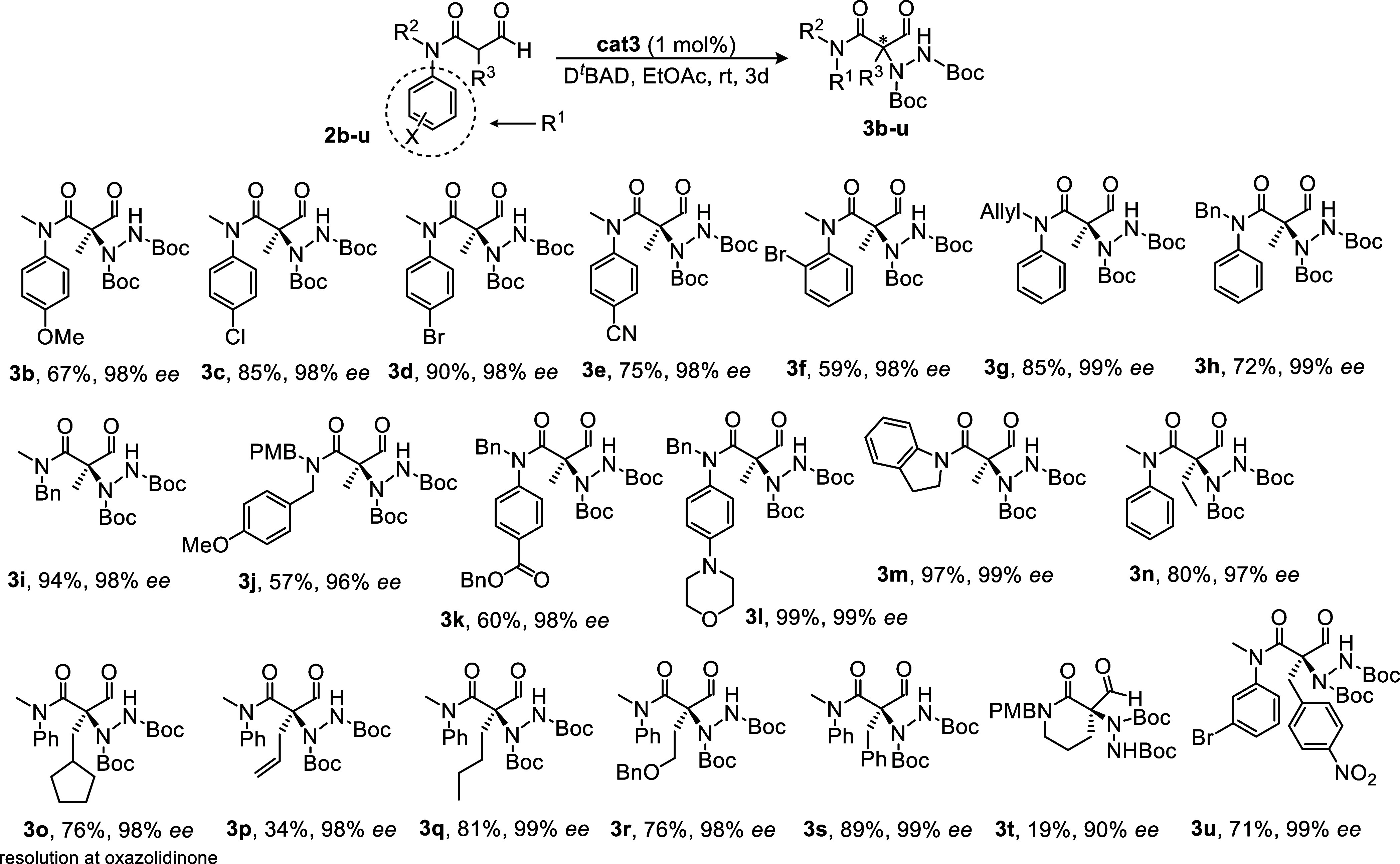
Substrate Scope for Enantioselective α-Hydrazination All of the reactions were carried out with **2** on an ∼0.4 mmol scale with D^*t*^BAD (1.2 equiv) and **cat3** (1 mol %) in EtOAc (1 mL) at room temperature under air. Yields refer to the isolated products after column chromatography, while the enantiomeric excesses (*ee*’s) were determined by HPLC analysis using a chiral stationary phase.

The α-methyl library also included a substrate (**3f**) containing an amide atrop-axis due to an *ortho*-substituted *N*-aryl group and a substrate (**3i**) consisting of a 2:1 mixture of s-*cis*/s-*trans* amide stereoisomers. Importantly, none of these stereogenic elements impacted the enantioselectivity to any great extent, indicating stereoselectivity to be reagent (catalyst) controlled. Additionally, and importantly for the methodology was the success with non-methyl R^3^ groups, covering a range of functionalized chains and rings (**3n**-**3u**). However, α-formyl amides containing relatively bulky groups close to the α-position (cyclopropyl and phenyl), gave no reaction by TLC (starting material was recovered quantitatively), presumably due to steric congestion^16^ (also see the methyl rule for β-ketoesters).^11h^ Enaminone resonance stabilization in the case of R^3^ = phenyl was also a likely factor. Indeed, α-tertiary amines bearing α-aryl groups are not easy to access in high *ee*, with the Clayden anionic N to C rearrangement being one of the few effective methods.^16^

The absolute configurations of two of the hydrazide products, **3h** and **3m**, *via* single-crystal X-ray determinations returned (*R*)-configurations for both compounds as shown in Figure 3 for **3m**.

**Figure 3:**
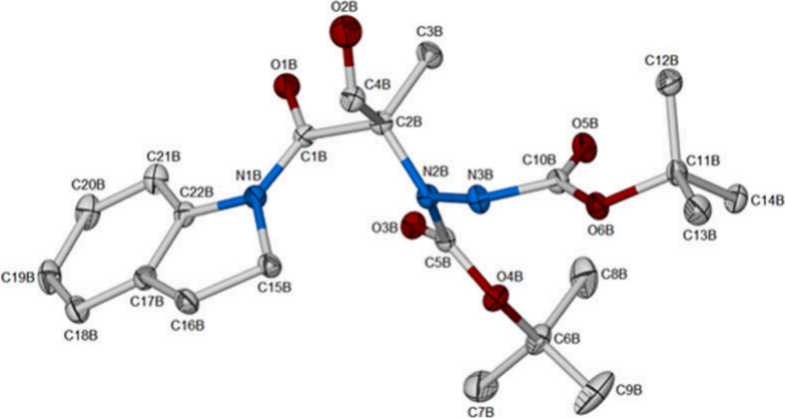
X-ray structure of **3m**.

For explaining these enantioselectivities, it was first important to note that without HCl (provided from the catalyst HCl salt), yields and *ee*’s were impaired (see page S3 of the Supporting Information), strongly pointing toward a Brønsted acid-catalyzed aminocatalysis mechanism being operative. This was supported by DFT calculations, which revealed the lowest energy transition state (see **TS1** in the Supporting Information) to involve *Re*-face attack of D^*t*^BAD onto an intramolecularly H-bonded *Z*-enaminone with Brønsted acid assistance provided by the *Cinchona* quinuclidinium ion to the D^*t*^BAD nitrogen. These features are depicted in Figure 4 for **TS1** involving (*E*)-D^*t*^BAD addition to the enaminone *Re*-face leading to an *R*-configured product in agreement with the X-ray results. These results are in keeping with literature precedent on Brønsted acid-assisted delivery of a diazo reagent.^13,17^

**Figure 4:**
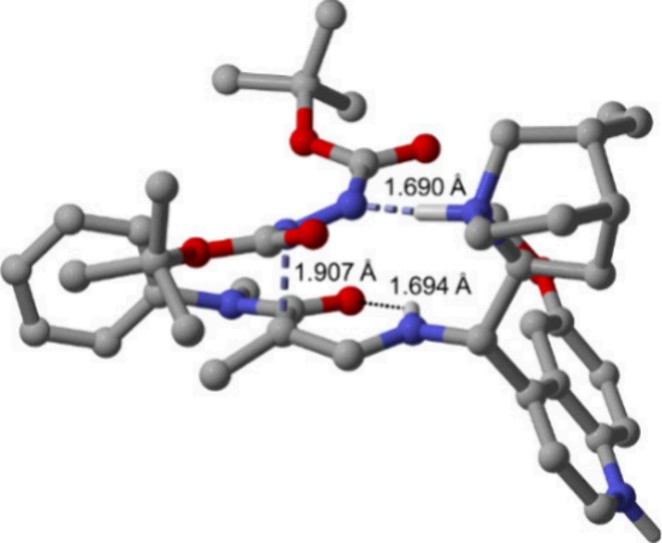
*Re*-face addition in the transition state (TS1 in the Supporting Information) for enantioselective hydrazination of **2a** with (*E*)-D^*t*^BAD.

To illustrate the synthetic utility of this method, hydrazides **3a**, **3c**, **3j**, **3n** and **3o** were transformed into oxazolidinone hydrazides **4a**, **4c**, **4j**, **4n** and **4o** in good yields and excellent ee’s *via* formyl reduction and cyclization (Scheme 2). Nitrous acid has been used for N–N cleavage in such quaternary systems, albeit under harsh conditions (110 °C),^15^ so we were gratified to find that *tert-*butyl nitrite could achieve the same transformation but at room temperature to afford oxazolidinones **5a** and **5j**, the latter transformable into its primary amide **6j** as shown in Scheme 2.

**Scheme 2:**
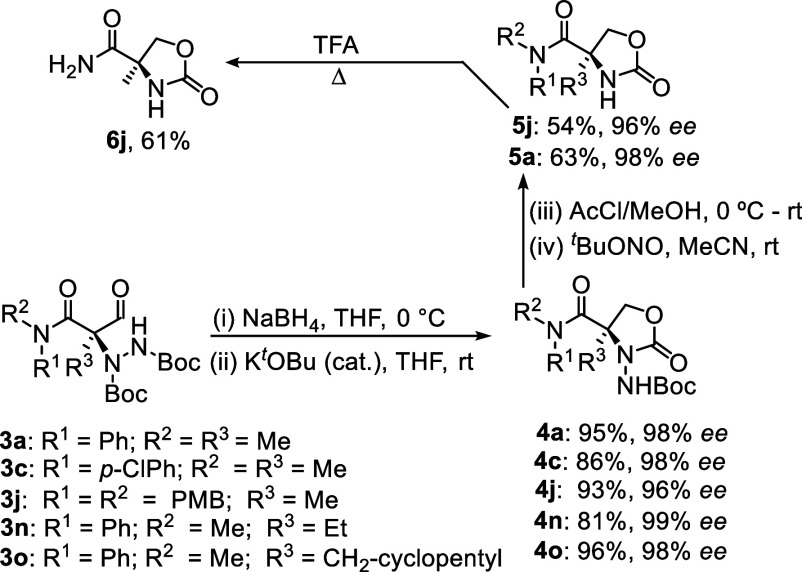
Synthetic Applications

In summary, we have developed the first highly enantioselective amination of α-formyl tertiary amides for gaining facile access to quaternized serine tertiary amides in extremely high *ee*’s and good yields. Such tertiary amides are of value for studies on natural product mimics (e.g., the amicetins^18^). Mechanistic evidence for the reaction strongly points toward a Brønsted-acid-assisted aminocatalysis mechanism being operative, adding further support for this powerful stereodirecting manifold.^13,17^

## Data Availability

The data underlying this study are available in the published article and its Supporting Information.

## Supporting Information Available

Experimental optimizations, procedures, compound characterization data, computational and X-ray crystal data, and NMR spectra and HPLC traces of the products (PDF)
