# Supplementary material for: Cinchona Organocatalyzed Enantioselective Amination for Quaternized Serines as Tertiary Amides
Source: Org Lett. 2024 Oct 16;26(42):9162–7. doi: 10.1021/acs.orglett.4c03650 (PMC11519915; doi:10.1021/acs.orglett.4c03650)
Supplement: Supplementary file 1 — ol4c03650_si_001.pdf [file ol4c03650_si_001.pdf]

Cinchona Organocatalyzed Enantioselective Amination for Quaternized Serines as Tertiary Amides

Phathutshedzo Masithi,[a] Ashlyn D. Bhana,[a] Gerhard A. Venter,[a] Hong Su,[a] Christopher D. Spicer,[b] Wade F. Petersen,[a,]* Roger Hunter.[a,]*

[a]Department of Chemistry, University of Cape Town, Rondebosch, Cape Town, 7700, South Africa.

[b]Department of Chemistry, University of York, Heslington, York, YO10 5DD, UK.

Email: roger.hunter@uct.ac.za

Table of Contents:

1. General information……………………………………………………………......... S2

2. Proposed *Z*-configuration for the enaminone intermediate…………………………... S3

3. Reaction optimization of catalyst and solvent ……………………………………… S3

4. Procedures for α-formyl amide synthesis…………………………………………… S4

   Step 1: Synthesis of anilides **1a-u**………………………………………………....... S4

   (i) Synthesis of anilides from acid chlorides……………………………………....... S4

   (ii) Synthesis of anilides from carboxylic acids……………………………………… S5

   (iii) Synthesis of anilides from secondary amides…………………………………… S5

   Characterization data for **1a-u**…………………………………………………….. S5

   Step 2: Synthesis of α-formyl amides **2a-u**……………………………………......... S12

   Characterization data for **2a-u**…………………………………………………….. S13

5. General procedure for enantioselective hydrazination of α-formyl amides **2** into **3**…. S20

   Characterization data for **3a-u**…………………………………………………….. S21

6. N-N bond cleavage studies…………………………………………...................... S33

   (i) Step 1: Reductive cyclization to oxazolidinone hydrazides **4a**, **4c**, **4j**, **4n** and **4o**…. S33

   (ii) Step 2: N-N bond cleavage to oxazolidinones **5a** and **5j**………………………...... S36

   (iii) Step 3: Deprotection of PMB groups of **5j** to afford **6j**…………………….......... S37

7. Enaminone transition structures from computer modelling………………………...... S38

8. X-Ray crystallographic data for compound **3h** and **3m**.................................... S43

9. References.. S46

10. NMR spectra of of **1-3a-u**, **4a**, **4c**, **4j**, **4n**, **4o**, **5a**, **5j**, **6j**.................................. S47

11. HPLC traces of **1-3a-u**, **4a**, **4c**, **4j**, **4n**, **4o**, **5a**, **5j**... S119

12. The End.. S145

1. General Information

Unless otherwise specified, all reagents were purchased commercially and used without further purification. THF was freshly distilled from sodium wire and benzophenone, and CH_2Cl_2 was distilled from phosphorus pentoxide. Where necessary, reactions were run under an inert atmosphere (argon). All of the heated reactions were done so using a thermostat-controlled heating block. ^{1}H NMR and ^{13}C NMR spectra were recorded on Varian Mercury 300 MHz (75.5 MHz for ^{13}C), Bruker 400 MHz (101.1 MHz for ^{13}C), or Bruker 600 MHz (151.0 MHz for ^{13}C) instruments. All spectral data were acquired at 298 K. Chemical shifts are reported in parts per million (ppm, δ), downfield from tetramethylsilane (TMS, δ = 0.00 ppm), and are referenced to residual solvent [$CDCl_3$, δ = 7.26 ppm (^{1}H) and 77.16 ppm (^{13}C)]. Coupling constants (*J*) are reported in Hertz (Hz). The multiplicity abbreviations used are br (broad), s (singlet), d (doublet), t (triplet), q (quartet), p (pentet), sext (sextet), m (multiplet), app (apparent). Infrared (IR) spectra were recorded on a PerkinElmer Spectrum 100 FT-IR spectrometer. High-resolution mass spectra were obtained from the Central Analytical Facility at Stellenbosch University and recorded in electrospray positive mode with a time-of-flight analyzer system on a Waters Synapt G2 machine. Thin-layer chromatography was carried out on Merck silica gel $60F_{254}$ precoated aluminum foil sheets and were visualized using UV light (254 nm) or by staining with acidic *p*-anisaldehyde or ninhydrin solutions. Melting points were obtained using a Reichert-Jung Thermovar hot-stage microscope. Flash column chromatography was carried out using silica gel 60 (Merck 7734), eluting with the specified solvent system. Optical rotations were obtained using a Perkin Elmer 343 polarimeter at λ = 589 nm and 20.0 °C. The concentration *c* refers to g/100mL. The enantiomeric excess (*ee*) of the products were determined by HPLC on an Agilent 1220 Series using a Daicel Chiralpak IA (250 × 4.6 mm), Daicel Chiralpak AD (250 × 4.6 mm), or Daicel Chiralcel OD column (250 × 4.6 mm).

2. Proposed *Z*-configuration for the enaminone intermediate

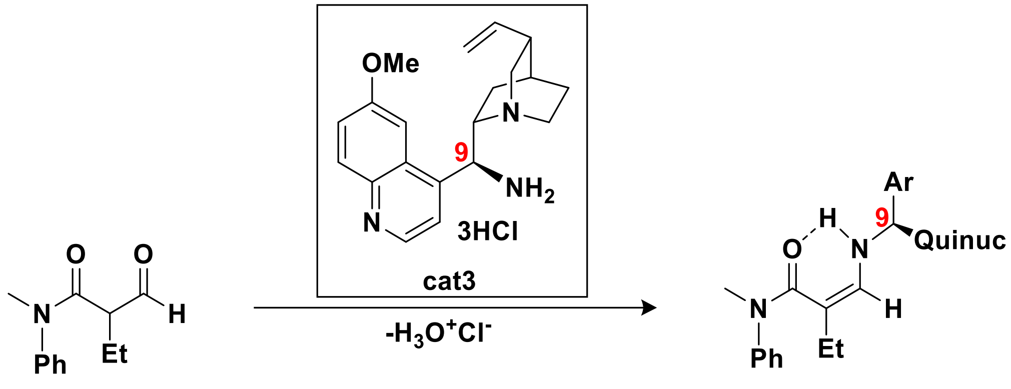

3. Reaction optimization of catalyst and solvent

(i) Catalyst screening in DCM at a catalyst loading of 1 mol%

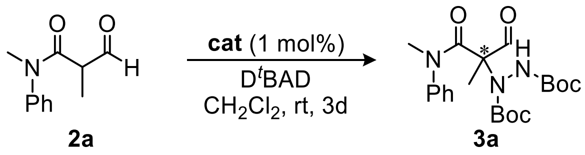

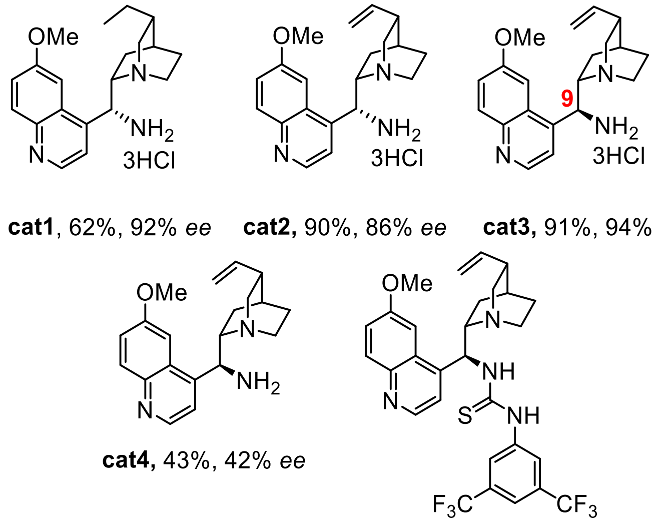

cat1, 62%, 92% *ee* **cat2,** 90%, 86% *ee* **cat3,** 91%, 94% *ee*

cat4, 43%, 42% *ee*

cat5, 32%, 38% *ee*

(ii) Solvent and catalyst loading screening

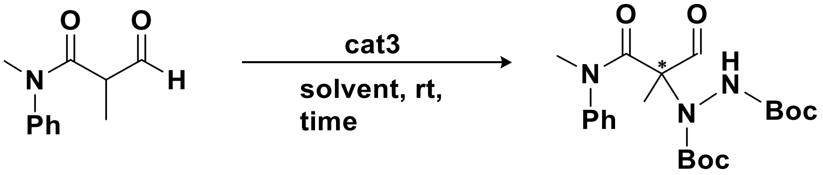

2a **3a**

Entry	Equiv. of cat3 (mol%)	Solvent	Nitrogen source	Reaction time	Yield (%)	*ee* (%)
1.	1	MeCN	D*t*BAD	3d	90	88
2.	1	EtOAc	D*t*BAD	3d	88	98
3.	1	PhMe	D*t*BAD	3d	82	98
4.	1	Et_2O	D*t*BAD	3d	81	98
5.	5	EtOAc	D*t*BAD	2d	68	98
6.	10	EtOAc	D*t*BAD	2d	61	98
7.	1	EtOAc	DBAD	3d	86	83
8.	1	EtOAc	Trisyl azide	10d	RSM (34)	ND
9.	1	EtOAc	D*t*BAD	18d	82	98[a]
10.	10	EtOAc	D*t*BAD	12d	43	41[b]

Unless otherwise stated: (i) reactions were carried out using **cat3** at room temperature under air using **2a** (0.52 mmol scale. [a]SM (starting material) = 1.00 g (~ 5 mmol). [b]catalyst as a free NH_2. DBAD = dibenzyl azodicarboxylate. Trisyl azide = 2,4,6-Triisopropylbenzenesulfonyl azide**.** RSM = recovered SM. ND = not determined.

4. Procedures for α-formyl amide synthesis of 2a-u

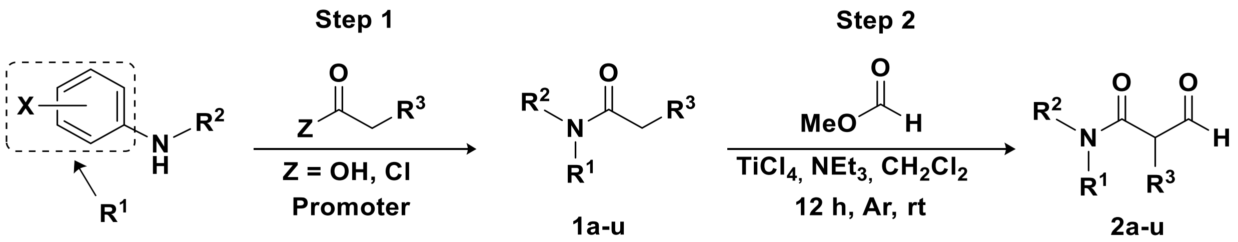

4(a) Step 1: Synthesis of anilides 1a-u

(i) Synthesis of anilides from acid chlorides

To a solution of the aniline derivative (1.0 mmol) in anhydrous CH_2Cl_2 (3 mL/mmol of substrate) cooled to 0 °C was added acid chloride (1.5 eq), prepared by reaction of the carboxylic acid with either thionyl chloride or oxalyl chloride. NEt_3 (3.0 eq) was added dropwise, and the solution allowed to warm to room temperature. After the reaction reached completion, as indicated by TLC (1.5 - 4 h), HCl (1 M) was added, and the organic layer separated. The aqueous phase was then extracted with CH_2Cl_2 (x 2). The combined organic extracts were washed with sat. $NaHCO_3$, dried over $MgSO_4$ and filtered. The solvent was concentrated under reduced pressure and the crude product was purified by flash chromatography using an ethyl acetate/hexane mixture as eluent. For compound **1i** which exists as s-*cis*/s-*trans* stereoisomers, the NMR data for the minor stereoisomer is reported in brackets.

(ii) Synthesis of anilides from carboxylic acids

To a solution of 2-chloro-1-methylpyridinium iodide (1.4 eq) in anhydrous CH_2Cl_2 (3 mL/mmol of substrate) cooled to 0 °C under argon was added carboxylic acid (1.2 eq) followed by the aniline derivative (1.0 eq), and the solution stirred at this temperature for 30 min. NEt_3 (3.0 eq) was added dropwise to the reaction mixture, and the solution allowed to warm to room temperature and stirred overnight. HCl (1 M) was added, and the organic layer was separated. The aqueous phase was extracted with CH_2Cl_2 (x 2) and the combined organic extracts were washed with sat. $NaHCO_3$, dried over $MgSO_4$ and filtered. The solvent was removed under reduced pressure and the crude product was purified by flash column chromatography using an ethyl acetate/hexane mixture as eluent.

(iii) Synthesis of anilides from secondary amides

To a solution of secondary amide (1.0 eq) in dry acetonitrile (2 mL/mmol of substrate) was added an alkylating reagent (1.2 eq) followed by Cs_2CO_3 (1.5 eq), and NaI (20 mol%). The mixture was refluxed overnight. Thereafter, the reaction was cooled to room temperature, quenched with H_2O, and the solution extracted with ethyl acetate (x 2). The combined organic layers were dried over $MgSO_4$ and filtered. The solvent was removed under reduced pressure and the crude product was purified by flash column chromatography using an ethyl acetate/hexane mixture as eluent.

Characterization data for 1a-u

***N*-Methyl-*N*-phenylpropionamide[1] (1a)**

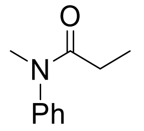

Produced using procedure (ii) with 2-chloro-1-methylpyridinium iodide (11.4 g, 44.8 mmol), propionic acid (3.4 mL, 45 mmol), *N*-methylaniline (4.0 mL, 37 mmol), and NEt_3 (15.6 mL, 112 mmol). Chromatography on silica gel with EtOAc/hexane (10–20%) afforded **1a** as an off-white crystalline solid (4.0 g, 66%). $\mathbf{R_f}$ = 0.24 (3:7 EtOAc: hexane); **^{1}H NMR** (400 MHz, $CDCl_3$) δ 7.41 – 7.30 (m, 3H), 7.16 (d, J = 7.2 Hz, 2H), 3.25 (s, 3H), 2.07 (q, J = 7.5 Hz, 2H), 1.03 (t, J = 7.5 Hz, 3H); **^{13}C NMR** (151 MHz, $CDCl_3$) δ 173.9, 144.2, 129.6, 127.6, 127.2, 37.2, 27.4, 9.6. All recorded data were in accordance with those previously reported.[1]

***N*-4-Methoxyphenyl-*N*-methylpropionamide[2] (1b)**

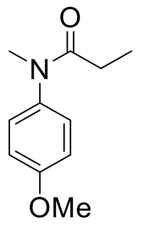

Produced using procedure (ii) with 2-chloro-1-methylpyridinium iodide (11.2 g, 43.7 mmol), propionic acid (3.3 mL, 6.9 mmol), 4-methoxy-*N*-methylaniline (5.0 g, 37 mmol), and NEt_3

(15.3 mL, 110 mmol). Chromatography on silica gel with EtOAc/hexane (10–20%) afforded **1b** as a brown solid (5.6 g, 79%). **R_f** = 0.20 (3:7 EtOAc: hexane); **^{1}H NMR** (400 MHz, $CDCl_3$) δ 7.08 (d, *J* = 8.8 Hz, 2H), 6.90 (d, *J* = 8.8 Hz, 2H), 3.81 (s, 3H), 3.21 (s, 3H), 2.05 (q, *J* = 7.5 Hz, 2H), 1.03 (t, *J* = 7.5 Hz, 3H); **^{13}C NMR** (101 MHz, $CDCl_3$) δ 174.3, 158.8, 137.1, 128.3, 114.8, 55.5, 37.4, 27.4, 9.7. All recorded data were in accordance with those previously reported.[2]

***N*-(4-Chlorophenyl)-*N*-methylpropionamide[2] (1c)**

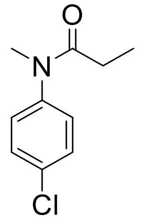

Produced using procedure (ii) with 2-chloro-1-methylpyridinium iodide (10.8 g, 42.4 mmol), propionic acid (3.1 mL, 42 mmol), 4-chloro-*N*-methylaniline (5.0 g, 35 mmol), and NEt_3 (10.7 mL, 106 mmol). Chromatography on silica gel with EtOAc/hexane (10–20%) afforded **1c** as a brown oil (3.6 g, 52%). **R_f** = 0.34 (3:7 EtOAc: hexane); **^{1}H NMR** (400 MHz, $CDCl_3$) δ 7.30 (d, *J* = 8.6 Hz, 2H), 7.06 (d, *J* = 8.6 Hz, 2H), 3.16 (s, 3H), 2.00 (q, *J* = 7.5 Hz, 2H), 0.97 (t, *J* = 7.5 Hz, 3H); **^{13}C NMR** (101 MHz, $CDCl_3$) δ 173.9, 143.0, 133.7, 130.1, 128.9, 37.5, 27.7, 9.8. All recorded data were in accordance with those previously reported.[2]

***N*-(4-Bromophenyl)-*N*-methylpropionamide[2] (1d)**

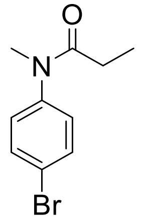

Produced using procedure (ii) with 2-chloro-1-methylpyridinium iodide (6.6 g, 26 mmol), propionic acid (1.9 mL, 26 mmol), 4-bromo-*N*-methylaniline (4.0 g, 22 mmol), and NEt_3 (8.4 mL, 65 mmol). Chromatography on silica gel with EtOAc/hexane (10–20%) afforded **1d** as a brown oil (4.1 g, 80%). **R_f** = 0.42 (3:7 EtOAc: hexane); **^{1}H NMR** (300 MHz, $CDCl_3$) δ 7.52 (d, *J* = 8.6 Hz, 2H), 7.05 (d, *J* = 8.6 Hz, 2H), 3.22 (s, 3H), 2.11 – 2.04 (m, 2H), 1.04 (t, *J* = 7.4 Hz, 3H); **^{13}C NMR** (101 MHz, $CDCl_3$) δ 173.4, 143.2, 132.9, 129.0, 121.3, 37.2, 27.5, 9.6. All recorded data were in accordance with those previously reported.[2]

***N*-(4-Cyanophenyl)-*N*-methylpropionamide (1e)**

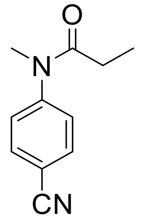

Produced using procedure (i) with 4-(*N*-methylamino)benzonitrile (1.0 g, 7.8 mmol), propionyl chloride (1.0 mL, 11 mmol), and NEt_3 (3.3 mL, 23 mmol). Chromatography on silica gel with EtOAc/hexane (10–20%) afforded **1e** as a white solid (1.2 g, 86%). $\mathbf{R_f}$ = 0.33 (3:7 EtOAc: hexane); **IR** (film, ν_{max}/cm^{-1}) 2927, 2226, 1648; ^{1}**H NMR** (400 MHz, $CDCl_3$) δ 7.71 (d, *J* = 8.6 Hz, 2H), 7.32 (d, *J* = 8.6 Hz, 2H), 3.29 (s, 3H), 2.16 (q, *J* = 7.4 Hz, 2H), 1.08 (t, *J* = 7.4 Hz, 3H); ^{13}**C NMR** (101 MHz, $CDCl_3$) δ 173.8, 148.6, 134.0, 128.2, 118.5, 111.5, 37.7, 28.2, 9.9; **HRMS** (ESI^+) *m/z*: $[M+H]^+$ calculated for $C_{11}H_{13}N_2O^+$ 189.1022; found 189.1028.

N-(2-Bromophenyl)-*N*-methylpropionamide[3] (1f)

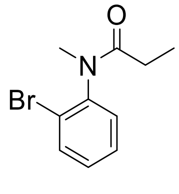

Produced using procedure (i) with 2-bromo-*N*-methylaniline (1.0 g, 5.4 mmol), propionyl chloride (0.7 mL, 8.1 mmol), and NEt_3 (2.2 mL, 16 mmol). Chromatography on silica gel with EtOAc/hexane (10–20%) afforded **1f** as a yellow oil (1.2 g, 92%). $\mathbf{R_f}$ = 0.39 (1:4 EtOAc: hexane); ^{1}**H NMR** (300 MHz, $CDCl_3$) δ 7.62 (d, *J* = 7.9 Hz, 1H), 7.34 (t, *J* = 7.2 Hz, 1H), 7.24 – 7.17 (m, 2H), 3.13 (s, 3H), 1.92 (q, *J* = 7.5 Hz, 2H), 0.99 (t, *J* = 7.5 Hz, 3H); ^{13}**C NMR** (101 MHz, $CDCl_3$) δ 173.5, 142.4, 133.6, 129.5, 129.5, 128.7, 123.2, 35.5, 27.0, 9.1. All recorded data were in accordance with those previously reported.[3]

N-Allyl-*N*-phenylpropionamide (1g)

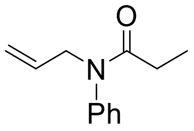

Produced using procedure (ii) with 2-chloro-1-methylpyridinium iodide (9.2 g, 36 mmol), propionic acid (2.7 mL, 36 mmol), *N*-allylaniline (4.00 g, 30.0 mmol), and NEt_3 (12.5 mL, 90.1 mmol). Chromatography on silica gel with EtOAc/hexane (10–15%) afforded **1g** as a yellow oil (4.4 g, 78%). $\mathbf{R_f}$ = 0.48 (3:7 EtOAc: hexane); **IR** (film, ν_{max}/cm^{-1}) 2978, 1655, 1458, 1357; ^{1}**H NMR** (400 MHz, $CDCl_3$) δ 7.39 – 7.26 (m, 3H), 7.13 (d, *J* = 7.5 Hz, 2H), 5.89 – 5.79 (ddt, *J* = 16.7, 10.2, 6.3 Hz, 1H), 5.09 – 5.01 (m, 2H), 4.27 (d, *J* = 6.3 Hz, 2H), 2.04 (q, *J* = 7.4 Hz, 2H), 1.03 (t, *J* = 7.4 Hz, 3H); ^{13}**C NMR** (101 MHz, $CDCl_3$) δ 173.2, 142.3, 133.0, 129.2, 128.0, 127.5, 117.4, 51.9, 27.5, 9.3; **HRMS** (ESI^+) *m/z*: $[M+H]^+$ calculated for $C_{12}H_{16}NO^+$ 190.1226; found 190.1233.

N-Benzyl-*N*-phenylpropionamide[4] (1h)

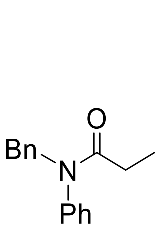

Produced using procedure (ii) with 2-chloro-1-methylpyridinium iodide (3.4 g, 13 mmol), propionic acid (1.0 mL, 13 mmol), *N*-benzylaniline (2.0 g, 11 mmol), and NEt_3 (4.6 mL, 33 mmol). Chromatography on silica gel with EtOAc/hexane (10–15%) afforded **1h** as a yellow oil (2.0 g, 75%). $\mathbf{R_f}$ = 0.37 (3:7 EtOAc: hexane); **^{1}H NMR** (400 MHz, $CDCl_3$) δ 7.34 – 7.19 (m, 8H), 6.98 (d, *J* = 7.7 Hz, 2H), 4.89 (s, 2H), 2.09 (q, *J* = 7.4 Hz, 2H), 1.08 (t, *J* = 7.4 Hz, 3H); **^{13}C NMR** (101 MHz, $CDCl_3$) δ 174.1, 142.9, 138.1, 129.8, 129.2, 128.8, 128.7, 128.2, 127.6, 53.4, 28.2, 10.0. All recorded data were in accordance with those previously reported.[4]

_N_-Benzyl-_N_-methylpropionamide[5] (1i)

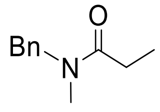

Produced using procedure (i) with *N*-benzylmethylamine (1.0 g, 8.3 mmol), propionyl chloride (1.0 mL, 11 mmol), and NEt_3 (3.1 mL, 22 mmol). Chromatography on silica gel with EtOAc/hexane (10–30%) afforded **1i** as a colorless oil (1.3 g, 89%). $\mathbf{R_f}$ = 0.33 (3:7 EtOAc: hexane); **^{1}H NMR** (300 MHz, $CDCl_3$) (s-*cis*/s-*trans*) δ 7.35 – 7.14 (m, 5H), 4.59 (4.52) (s, 2H), 2.89 (2.95) (s, 3H), 2.39 (q, *J* = 7.4 Hz, 2H), 1.22 – 1.14 (m, 3H); **^{13}C NMR** (101 MHz, $CDCl_3$) δ 173.9 (174.2), 137.6 (136.8), 128.9, 128.6, 128.0 (127.5, 127.3, 126.3), 50.7 (53.2), 34.6 (33.9), 26.7 (26.3), 9.4 (9.6). All recorded data were in accordance with those previously reported.[5]

_N_,_N_-Bis(4-methoxybenzyl)propionamide (1j)

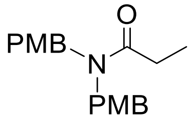

Produced using procedure (i) with bis(4-methoxybenzyl)amine (1.0 g, 4.0 mmol), propionyl chloride (0.5 mL, 5.7 mmol), and NEt_3 (1.6 mL, 12 mmol). Chromatography on silica gel with EtOAc/hexane (10–20%) afforded **1j** as a yellow solid (0.85 g, 70%). $\mathbf{R_f}$ = 0.20 (3:7 EtOAc: hexane); **IR** (film, ν_{max}/cm^{-1}) 2995, 1639, 1246; **^{1}H NMR** (400 MHz, $CDCl_3$) δ 7.15 (d, *J* = 8.6 Hz, 2H), 7.06 (d, *J* = 8.6 Hz, 2H), 6.88 (d, *J* = 8.7 Hz, 2H), 6.84 (d, *J* = 8.7 Hz, 2H), 4.51 (s, 2H), 4.36 (s, 2H), 3.80 (s, 3H), 3.78 (s, 3H), 2.43 (q, *J* = 7.4 Hz, 2H), 1.19 (t, *J* = 7.4 Hz, 3H); **^{13}C NMR** (101 MHz, $CDCl_3$) δ 174.1, 159.0, 158.9, 129.6, 128.4, 128.4, 127.6, 114.2, 113.9, 55.3, 55.2, 49.0, 47.2, 26.5, 9.6; **HRMS** (ESI$^+$) *m/z*: $[M+H]^+$ calculated for $C_{19}H_{24}NO_3^+$ 314.1751; found 314.1751.

Benzyl-4-(_N_-benzylpropionamido)benzoate (1k)

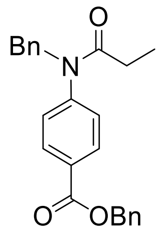

Produced using procedure (iii) with benzyl 4-propionamidobenzoate (2.9 g, 15 mmol), Cs_2CO_3 (7.3 g, 23 mmol), and benzyl bromide (2.1 mL, 18 mmol). Chromatography on silica gel with EtOAc/hexane (15-20%) afforded **1k** as a transparent oil (5.4 g, 96%). **R_f** = 0.38 (3:7 EtOAc: hexane); **IR** (film, ν_{max}/cm^{-1}) 2991, 1721, 1643, 1265; **^{1}H NMR** (300 MHz, $CDCl_3$) δ 8.03 (d, J = 8.5 Hz, 2H), 7.47 – 7.31 (m, 5H), 7.26 – 7.13 (m, 5H), 7.06 (d, J = 8.5 Hz, 2H), 5.36 (s, 2H), 4.91 (s, 2H), 2.10 (q, J = 7.4 Hz, 2H), 1.09 (t, J = 7.4 Hz, 3H); **^{13}C NMR** (151 MHz, $CDCl_3$) δ 173.2, 165.4, 146.7, 137.1, 135.7, 131.0, 129.5, 128.6, 128.5, 128.4, 128.3, 128.3, 128.2, 127.4, 66.9, 52.8, 27.9, 9.6; **HRMS** (ESI$^+$) *m/z*: [M+H]$^+$ for $C_{24}H_{23}NO_3^+$ 374.1756; found 374.1763.

***N*-Benzyl-*N*-(4-morpholinophenyl)propionamide (1l)**

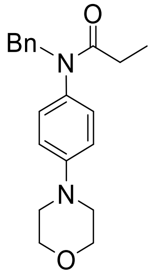

Produced using procedure (iii) with *N*-(4-morpholinophenyl)propionamide (1.0 g, 4.3 mmol), Cs_2CO_3 (2.1 g, 6.4 mmol), benzyl bromide (0.6 mL, 5.3 mmol), and NaI (0.13 g, 0.85 mmol). Chromatography on silica gel with EtOAc/hexane (10-30%) afforded **1l** as a yellow solid (1.3 g, 95%). **R_f** = 0.37 (6:4 EtOAc: hexane); **IR** (film, ν_{max}/cm^{-1}) 1650, 1338, 1120; **^{1}H NMR** (300 MHz, $CDCl_3$) δ 7.26 – 7.16 (m, 5H), 6.84 – 6.76 (m, 4H), 4.81 (s, 2H), 3.83 – 3.79 (m, 4H), 3.13 – 3.10 (t, 4H), 2.06 (q, J = 7.5 Hz, 2H), 1.04 (t, J = 7.5 Hz, 3H); **^{13}C NMR** (101 MHz, $CDCl_3$) δ 174.6, 150.8, 138.3, 134.6, 129.4, 129.3, 128.6, 127.5, 116.1, 67.1, 53.4, 49.2, 28.1, 10.1; **HRMS** (ESI$^+$) *m/z*: [M+H]$^+$ calculated for $C_{20}H_{25}N_2O_2^+$ 325.1911; found 325.1920.

1-(Indolin-1-yl)propan-1-one[6] (1m)

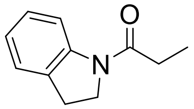

Produced using procedure (i) with indoline (1.0 g, 8.4 mmol), propionyl chloride (1.2 mL, 13 mmol), and NEt_3 (3.5 mL, 25 mmol) afforded **1m** as a crystalline white solid (1.4 g, 95%). No further purification was required, and the product was taken through to the next step as is. **R_f** = 0.27 (3:7 EtOAc: hexane); **^{1}H NMR** (300 MHz, $CDCl_3$) δ 8.24 (d, J = 7.9 Hz, 1H), 7.21 – 7.15 (m, 2H), 6.99 (td, J = 7.9, 1.1 Hz, 1H), 4.02 (t, J = 8.5 Hz, 2H), 3.17 (t, J = 8.5 Hz, 2H), 2.43 (q, J = 7.4 Hz, 2H), 1.23 (t, J = 7.4 Hz, 3H); **^{13}C NMR** (151 MHz, $CDCl_3$) δ 171.9, 143.0, 130.9, 127.4, 124.3, 123.3, 116.8, 47.7, 29.0, 27.9, 8.6; **HRMS** (ESI$^+$) *m/z*: [M+H]$^+$ calculated for $C_{11}H_{14}NO^+$ 176.1070; found 176.1079. All recorded data were in accordance with those previously reported.[6]

_N_-Methyl-_N_-phenylbutyramide[7] (1n)

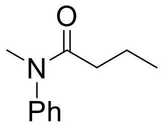

Produced using procedure (i) with _N_-methylaniline (4.0 mL, 37 mmol), butyryl chloride (5.8 mL, 56 mmol), and NEt_3 (15.6 mL, 112 mmol). Chromatography on silica gel with EtOAc/hexane (10–20%) afforded **1n** as a yellow liquid (6.2 g, 93%). $\mathbf{R_f}$ = 0.37 (3:7 EtOAc: hexane); **^{1}H NMR** (300 MHz, $CDCl_3$) δ 7.35 – 7.21 (m, 3H), 7.09 (d, _J_ = 7.0 Hz, 2H) 3.17 (s, 3H), 1.97 (t, _J_ = 7.4 Hz, 2H), 1.51 (sext, 2H), 0.73 (t, _J_ = 7.4 Hz, 3H); **^{13}C NMR** (151 MHz, $CDCl_3$) δ 173.2, 144.5, 129.9, 127.8, 127.5, 37.4, 36.2, 19.1, 14.0. All recorded data were in accordance with those previously reported.[7]

3-Cyclopentyl-_N_-methyl-_N_-phenylpropanamide[2] (1o)

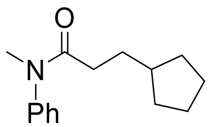

Produced using procedure (ii) with 2-chloro-1-methylpyridinium iodide (3.6 g, 14 mmol), 3-cyclopentylpropionic acid (2.0 g, 14 mmol), _N_-methylaniline (1.2 mL, 11 mmol), and NEt_3 (4.9 mL, 35 mmol). Chromatography on silica gel with EtOAc/hexane (10–20%) afforded **1o** as a yellow oil (2.60 g, 97%). $\mathbf{R_f}$ = 0.37 (3:7 EtOAc: hexane); **^{1}H NMR** (400 MHz, $CDCl_3$) δ 7.38 – 7.28 (m, 3H), 7.13 (d, _J_ = 7.6 Hz, 2H), 3.20 (s, 3H), 2.03 (t, _J_ = 7.3 Hz, 2H), 1.54 – 1.35 (m, 9H), 0.90 – 0.84 (m, 2H); ^{13}C **NMR** (101 MHz, $CDCl_3$) δ 173.1, 143.9, 129.3, 127.3, 126.9, 39.2, 36.9, 33.0, 31.9, 31.4, 24.6. All recorded data were in accordance with those previously reported.[2]

_N_-Methyl-_N_-phenylpent-4-enamide[8] (1p)

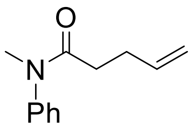

Produced using procedure (ii) with 2-chloro-1-methylpyridinium iodide (2.9 g, 11 mmol), pent-4-enoic acid (1.1 mL, 11 mmol), _N_-methylaniline (1.0 mL, 9.3 mmol), and NEt_3 (4.0 mL, 29 mmol). Chromatography on silica gel with EtOAc/hexane (10–20%) afforded **1p** as a brown oil (1.50 g, 83%). $\mathbf{R_f}$ = 0.18 (1:4 EtOAc: hexane); **^{1}H NMR** (400 MHz, $CDCl_3$) δ 7.41 – 7.32 (m, 2H), 7.30 – 7.26 (m, 1H), 7.14 – 7.12 (m, 2H), 5.72 – 5.62 (m, 1H), 4.93 – 4.80 (m, 2H), 3.21 (s, 3H), 2.28 (q, _J_ = 7.5 Hz, 2H), 2.11 (t, _J_ = 7.5 Hz, 2H); **^{13}C NMR** (101 MHz, $CDCl_3$) δ 172.1, 144.0, 137.4, 129.7, 127.7, 127.2, 114.9, 37.2, 33.3, 29.3. All recorded data were in accordance with those previously reported.[8]

N-Methyl-N-phenylhexanamide (1q)

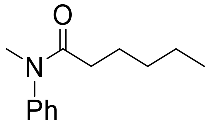

Produced using procedure (ii) with 2-chloro-1-methylpyridinium iodide (2.9 g, 11 mmol), hexanoic acid (1.3 mL, 11 mmol), N-methylaniline (1.0 mL, 9.3 mmol), and NEt_3 (4.0 mL, 28 mmol). Chromatography on silica gel with EtOAc/hexane (10–20%) afforded **1q** as a brown oil (1.30 g, 68%). $\mathbf{R_f}$ = 0.17 (1.5:8.5 EtOAc: hexane); **IR** (film, ν_{max}/cm^{-1}) 2986, 1641; **^{1}H NMR** (400 MHz, $CDCl_3$) δ 7.35 – 7.31 (m, 2H), 7.27 – 7.23 (m, 1H), 7.11 – 7.08 (m, 2H), 3.18 (s, 3H), 1.98 (t, J = 7.6 Hz, 2H), 1.49 (p, J = 7.4 Hz, 2H), 1.23 – 0.99 (m, 4H), 0.74 (t, J = 6.9 Hz, 3H); **^{13}C NMR** (101 MHz, $CDCl_3$) δ 173.1, 144.2, 129.5, 127.5, 127.2, 37.1, 33.9, 31.3, 25.1, 22.2, 13.7; **HRMS** (ESI$^+$) m/z: $[M+H]^+$ calculated for $C_{13}H_{20}NO^+$ 206.1540; found 206.1543.

4-(Benzyloxy)-N-methyl-N-phenylbutanamide[2] (1r)

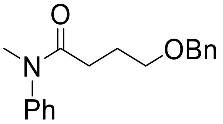

Produced using procedure (ii) with 2-chloro-1-methylpyridinium iodide (1.3 g, 5.2 mmol), 4-(benzyloxy)butanoic acid (1.0 g, 5.2 mmol), N-methylaniline (0.5 mL, 4.6 mmol), and NEt_3 (1.8 mL, 13 mmol). Chromatography on silica gel with EtOAc/hexane (10–20%) afforded **1r** as a yellow oil (0.65 g, 54%). $\mathbf{R_f}$ = 0.15 (3:7 EtOAc: hexane); **^{1}H NMR** (400 MHz, $CDCl_3$) δ 7.42 – 7.14 (m, 10H), 4.41 (s, 2H), 3.43 (t, J = 6.2 Hz, 2H), 3.26 (s, 3H), 2.19 (t, J = 7.4 Hz, 2H), 1.91 (p, J = 6.7 Hz, 2H); **^{13}C NMR** (101 MHz, $CDCl_3$) δ 172.8, 144.2, 138.6, 129.8, 128.3, 127.8, 127.6, 127.5, 127.4, 72.6, 69.5, 37.3, 30.8, 25.5. All recorded data were in accordance with those previously reported.[2]

N-Methyl-N,3-diphenylpropanamide[2] (1s)

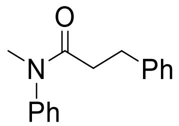

Produced using procedure (ii) with 2-chloro-1-methylpyridinium iodide (5.1 g, 22 mmol), 3-phenylpropanoic acid (3.4 g, 22 mmol), N-methylaniline (2.0 mL, 19 mmol), and NEt_3 (7.8 mL, 56 mmol). Chromatography on silica gel with EtOAc/hexane (10–20%) afforded **1s** as a yellow oil (4.10 g, 92%). $\mathbf{R_f}$ = 0.46 (3:7 EtOAc: hexane); **^{1}H NMR** (400 MHz, $CDCl_3$) δ 7.38 – 7.16 (m, 6H), 7.07 – 7.01 (m, 4H), 3.25 (s, 3H), 2.91 (t, J = 7.8 Hz, 2H), 2.37 (t, J = 7.8 Hz, 2H); **^{13}C NMR** (101 MHz, CDCl3) δ 172.0, 143.8, 141.0, 129.5, 128.2, 128.1, 127.6, 127.1, 125.8, 37.1, 35.8, 31.6. All recorded data were in accordance with those previously reported.[2]

1-(4-Methoxybenzyl)piperidin-2-one[9] (1t)

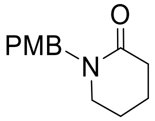

Produced using procedure (iii) with piperidin-2-one (1.00 g, 10.1 mmol), NaH (0.44 g, 11.1 mmol), NaI (0.30 g, 2.02 mmol), and 4-methoxybenzyl chloride (1.5 mL, 11 mmol) in THF. Chromatography on silica gel with EtOAc/hexane (20–50%) afforded **1t** as a colorless oil (1.30 g, 57%). $\mathbf{R_f}$ = 0.16 (4:1 EtOAc: hexane); **^{1}H NMR** (400 MHz, $CDCl_3$) δ 7.02 (d, *J* = 8.6 Hz, 2H), 6.67 (d, *J* = 8.6 Hz, 2H), 4.35 (s, 2H), 3.59 (s, 3H), 2.99 (t, *J* = 5.6 Hz, 2H), 2.25 (t, *J* = 6.3 Hz, 2H), 1.63 – 1.51 (m, 4H); **^{13}C NMR** (101 MHz, $CDCl_3$) δ 169.1, 158.5, 129.0, 129.0, 113.4, 54.7, 48.9, 46.5, 32.0, 22.7, 20.9. All recorded data were in accordance with those previously reported.[9]

***N*-(3-Bromophenyl)-*N*-methyl-3-(4-nitrophenyl)propanamide (1u)**

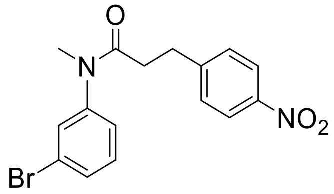

Produced using procedure (ii) with 2-chloro-1-methylpyridinium iodide (2.5 g, 9.7 mmol), 3-(4-nitrophenyl)propanoic acid (2.0 g, 9.7 mmol), 3-bromo-*N*-methylaniline (1.0 mL, 8.1 mmol), and NEt_3 (3.4 mL, 24 mmol). Chromatography on silica gel with EtOAc/hexane (10–30%) afforded **1u** as a brown solid (2.0 g, 66%). $\mathbf{R_f}$ = 0.14 (3:7 EtOAc: hexane); **IR** (film, ν_{max}/cm^{-1}) 2928, 1651, 1511, 1473; **^{1}H NMR** (300 MHz, $CDCl_3$) δ 8.08 – 8.03 (m, 2H), 7.44 – 7.40 (m, 1H), 7.26 – 7.21 (m, 3H), 7.09 (s, 1H), 6.99 (d, *J* = 8.8 Hz, 1H), 3.18 (s, 3H), 2.98 (t, *J* = 7.4 Hz, 2H), 2.38 (t, *J* = 7.4 Hz, 2H); **^{13}C NMR** (151 MHz, $CDCl_3$) δ 171.2, 149.2, 146.7, 145.1, 131.4, 131.3, 130.7, 129.6, 126.2, 123.8, 123.2, 37.6, 35.3, 31.5; **HRMS** (ESI$^+$) *m/z*: $[M+H]^+$ calculated for $C_{16}H_{16}BrN_2O_3^+$ 363.0339; found 363.0344.

....

4(b) Step 2: Synthesis of α-formyl amides (2a-u)

To a solution of **1** (1.0 eq) in anhydrous CH_2Cl_2 (3 mL/mmol of anilide) at 0 °C under argon was added methyl or ethyl formate (6.0 eq), which was stirred at this temperature for 10 min. $TiCl_4$ (3.0 eq) was added dropwise, and the solution stirred at 0 °C for 40 min. NEt_3 (3.5 eq) was added dropwise over a period of 5 min and the solution allowed to warm to room temperature overnight. The reaction mixture was quenched with H_2O and the organic layer was separated. The aqueous phase was extracted with CH_2Cl_2 (x 2), and the combined organic extracts were dried over $MgSO_4$ and filtered. The solvent was removed under reduced pressure and the crude product was purified by flash column chromatography using ethyl acetate/hexane mixtures as eluent. For the NMR data, minor stereoisomers are reported in brackets.

Characterization data for 2a-u

***N*,2-Dimethyl-3-oxo-*N*-phenylpropanamide[2] (2a)**

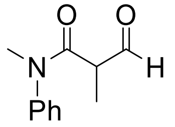

With **1a** (3.00 g, 18.4 mmol), methyl formate (6.8 mL, 110 mmol), $TiCl_4$ (6.1 mL, 56 mmol), and NEt_3 (9.0 mL, 65 mmol). Chromatography on silica gel with EtOAc/hexane (10–30%) afforded **2a** as a brown oil (3.40 g, 98%). $\mathbf{R_f}$ = 0.18 (3:7 EtOAc: hexane); **^{1}H NMR** (300 MHz, $CDCl_3$) δ 9.52 (d, J = 1.7 Hz, 1H), 7.46 – 7.30 (m, 3H), 7.21 – 7.14 (m, 2H), 3.32 – 3.19 (m, 4H), 1.23 (1.06) (d, J = 7.1 Hz, 3H); **^{13}C NMR** (101 MHz, $CDCl_3$) δ 198.9, 170.2, 143.4, 130.3, 128.7, 127.6, 50.6, 37.8, 12.6. All recorded data were in accordance with those previously reported.[2]

***N*-(4-Methoxyphenyl)-*N*,2-dimethyl-3-oxopropanamide[2] (2b)**

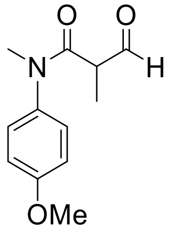

With **1b** (2.00 g, 10.4 mmol), methyl formate (3.4 mL, 62 mmol), $TiCl_4$ (3.4 mL, 31 mmol), and NEt_3 (5.1 mL, 36 mmol). Chromatography on silica gel with EtOAc/hexane (10–30%) afforded **2b** as a yellow oil (2.20 g, 97%). $\mathbf{R_f}$ = 0.11 (3:7 EtOAc: hexane); **^{1}H NMR** (400 MHz, $CDCl_3$) δ 9.56 (d, J = 1.7 Hz, 1H), 7.10 (d, J = 6.9 Hz, 2H), 6.92 (d, J = 6.9 Hz, 2H), 3.82 (s, 3H), 3.31 – 3.26 (m, 4H), 1.27 (1.08) (d, J = 7.0 Hz, 3H), 1.08; **^{13}C NMR** (101 MHz, $CDCl_3$) δ 199.4, 170.7, 159.8, 136.3, 128.9, 115.6, 56.0, 50.8, 38.1, 12.9. All recorded data were in accordance with those previously reported.[2]

***N*-(4-Chlorophenyl)-*N*,2-dimethyl-3-oxopropanamide[2] (2c)**

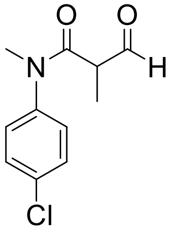

With **1c** (2.00 g, 10.1 mmol), methyl formate (3.7 mL, 60.7 mmol), $TiCl_4$ (3.3 mL, 30.4 mmol), and NEt_3 (4.9 mL, 35 mmol). Chromatography on silica gel with EtOAc/hexane (10–30%) afforded **2c** as a yellow oil (2.10 g, 92%). $\mathbf{R_f}$ = 0.17 (3:7 EtOAc: hexane); **^{1}H NMR** (300 MHz, $CDCl_3$) δ 9.47 (d, J = 1.7 Hz, 1H), 7.35 (d, J = 8.6 Hz, 2H), 7.11 (d, J = 8.6 Hz, 2H), 3.25 – 3.17 (m, 4H), 1.23 (1.03) (d, J = 7.1 Hz, 3H); **^{13}C NMR** (101 MHz, $CDCl_3$) δ 199.0, 170.2, 142.1,

134.9, 130.8, 129.3, 50.8, 38.0, 12.9. All recorded data were in accordance with those previously reported.[2]

***N*-(4-Bromophenyl)-*N*,2-dimethyl-3-oxopropanamide[2] (2d)**

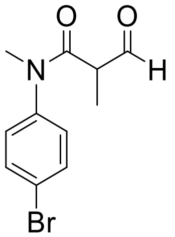

With **1d** (2.00 g, 8.26 mmol), methyl formate (3.0 mL, 50 mmol), $TiCl_4$ (2.7 mL, 25 mmol), and NEt_3 (4.0 mL, 29 mmol). Chromatography on silica gel with EtOAc/hexane (10–30%) afforded **2d** as a brown oil (2.10 g, 96%). $\mathbf{R_f}$ = 0.15 (3:7 EtOAc: hexane); **^{1}H NMR** (300 MHz, $CDCl_3$) δ 9.46 (d, *J* = 1.8 Hz, 1H), 7.50 (d, *J* = 8.6 Hz, 2H), 7.05 (d, *J* = 8.6 Hz, 2H), 3.28 – 3.16 (m, 4H), 1.22 (1.03) (d, *J* = 7.1 Hz, 3H); **^{13}C NMR** (101 MHz, $CDCl_3$) δ 199.1, 170.1, 142.6, 133.8, 129.6, 122.8, 50.8, 38.0, 12.9. All recorded data were in accordance with those previously reported.[2]

***N*-(4-Cyanophenyl)-*N*,2-dimethyl-3-oxopropanamide (2e)**

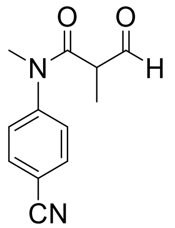

With **1e** (1.00 g, 5.31 mmol), methyl formate (1.9 mL, 32 mmol), $TiCl_4$ (1.8 mL, 16 mmol), and NEt_3 (2.6 mL, 18.8 mmol). Chromatography on silica gel with EtOAc/hexane (10–30%) afforded **2e** as a yellow solid (0.78 g, 67%). $\mathbf{R_f}$ = 0.25 (2:3 EtOAc: hexane); **IR** (film, ν_{max}/cm^{-1}) 1728, 1642; **M.P.** 68–73 ˚C; **^{1}H NMR** (400 MHz, $CDCl_3$) δ 9.52 (brs, 1H), 7.74 (d, *J* = 8.2 Hz, 2H), 7.34 (d, *J* = 8.2 Hz, 2H), 3.35 – 3.23 (m, 4H), 1.33 (1.09) (d, *J* = 7.1 Hz, 3H); **^{13}C NMR** (101 MHz, $CDCl_3$) δ 198.1, 169.1, 146.8, 133.7, 128.0, 117.5, 112.0, 50.2, 37.3, 12.2; **HRMS** (ESI$^+$) *m/z*: $[M+H]^+$ calculated for $C_{12}H_{13}N_2O_2^+$ 217.0972; found 217.0977.

***N*-(2-Bromophenyl)-*N*,2-dimethyl-3-oxopropanamide (2f)**

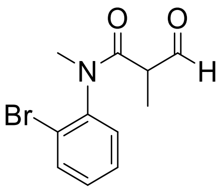

With **1f** (1.00 g, 4.13 mmol), ethyl formate (1.8 mL, 25 mmol), $TiCl_4$ (1.4 mL, 13 mmol), and NEt_3 (2.0 mL, 15 mmol). Chromatography on silica gel with EtOAc/hexane (10–30%) afforded **2f** as a yellow oil (0.80 g, 72%). $\mathbf{R_f}$ = 0.38 (3:7 EtOAc: hexane); **IR** (film, ν_{max}/cm^{-1}) 1719, 1653;

^{1}H NMR (300 MHz, $CDCl_3$) (atropisomers) δ 9.71 (9.47) (s, 1H), 7.72 – 7.68 (m, 1H), 7.48 – 7.29 (m, 3H), 3.23 (s, 3H), 3.12 (3.00) (app q, *J* = 7.2 Hz, 1H), 1.34 (1.20) (d, *J* = 7.1 Hz, 3H); **^{13}C NMR** (75 MHz, $CDCl_3$) δ 198.3 (198.1), 170.8 (169.2), 141.7 (141.6), 134.0 (133.9), 130.4 (129.5), 130.3 (130.3), 129.3 (129.2), 123.2 (123.1), 50.8 (50.4), 36.0 (35.9), 12.5 (12.0); **HRMS** (ESI^+) *m/z*: $[M+H]^+$ calculated for $C_{11}H_{13}BrNO_2^+$ 270.0125; found 270.0123.

***N*-Allyl-2-methyl-3-oxo-*N*-phenylpropanamide (2g)**

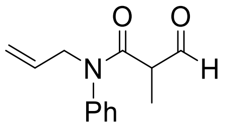

With **1g** (2.00 g, 10.6 mmol), methyl formate (3.9 mL, 64 mmol), $TiCl_4$ (3.5 mL, 32 mmol), and NEt_3 (5.2 mL, 37 mmol). Chromatography on silica gel with EtOAc/hexane (10–20%) afforded **2g** as a yellow oil (1.90 g, 83%). **R_f** = 0.29 (3:7 EtOAc: hexane); **IR** (film, ν_{max}/cm^{-1}) 1718, 1643; **^{1}H NMR** (400 MHz, $CDCl_3$) δ 9.56 (d, *J* = 1.8 Hz, 1H), 7.43 – 7.34 (m, 3H), 7.17 – 7.14 (m, 2H), 5.86 (ddt, *J* = 16.6, 10.1, 6.3 Hz, 1H), 5.14 – 5.06 (m, 2H), 4.36 – 4.26 (m, 2H), 3.24 (qd, *J* = 7.1, 1.8 Hz, 1H), 1.28 (d, *J* = 7.1 Hz, 3H); **^{13}C NMR** (101 MHz, $CDCl_3$) δ 198.8, 169.7, 141.7, 132.7, 130.0, 128.7, 128.6, 118.6, 52.6, 50.8, 12.6; **HRMS** (ESI^+) *m/z*: $[M+H]^+$ calculated for $C_{13}H_{16}NO_2^+$ 218.1176; found 218.1180.

***N*-Benzyl-2-methyl-3-oxo-*N*-phenylpropanamide[2] (2h)**

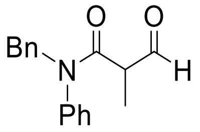

With **1h** (1.00 g, 4.18 mmol), methyl formate (1.5 mL, 25 mmol), $TiCl_4$ (1.4 mL, 13 mmol), and NEt_3 (2.0 mL, 15 mmol). Chromatography on silica gel with EtOAc/hexane (10–20%) afforded **2h** as a yellow oil (1.00 g, 86%). **R_f** = 0.15 (3:7 EtOAc: hexane); **^{1}H NMR** (400 MHz, $CDCl_3$) δ 9.58 (d, *J* = 1.7 Hz, 1H), 7.33 – 7.18 (m, 8H), 6.97 (dd, *J* = 6.7, 3.0 Hz, 2H), 4.94 (d, *J* = 14.2 Hz, 1H), 4.88 (d, *J* = 14.2 Hz, 1H), 3.25 (qd, *J* = 7.1, 1.7 Hz, 1H), 1.30 (d, *J* = 7.1 Hz, 3H); **^{13}C NMR** (101 MHz, $CDCl_3$) δ 198.8, 170.0, 141.4, 137.0, 129.9, 129.6, 128.9, 128.7, 128.6, 127.7, 53.3, 50.7, 12.5. All recorded data were in accordance with those previously reported.[2]

***N*-Benzyl-*N*,2-dimethyl-3-oxopropanamide (2i)**

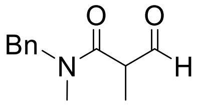

With **1i** (1.00 g, 5.7 mmol), methyl formate (2.0 mL, 34 mmol), $TiCl_4$ (1.9 mL, 17 mmol), and NEt_3 (2.8 mL, 20 mmol). Chromatography on silica gel with EtOAc/hexane (10–40%) afforded **2i** as a colorless oil (0.70 g, 62%). **R_f** = 0.17 (3:7 EtOAc: hexane); **IR** (film, ν_{max}/cm^{-1}) 1726, 1626; **^{1}H NMR** (300 MHz, $CDCl_3$) (s-*cis*/s-*trans*) δ 9.55 (9.50) (d, *J* = 1.7 Hz, 1H), 7.24 – 7.02 (m, 5H), 4.53 – 4.37 (m, 2H), 3.56 – 3.43 (m, 1H), 2.82 (2.83) (s, 3H), 1.28 (1.23) (d, *J* = 7.0 Hz, 3H); **^{13}C NMR** (101 MHz, $CDCl_3$) δ 198.4 (198.3), 169.9 (170.3), 136.5 (135.9), 128.4 (128.7), 127.6

(127.5), 127.2 (126.0), 49.2 (53.0, 50.5, 49.2), 34.6 (34.6, 33.8, 33.8), 11.3 (11.9); **HRMS** (ESI$^+$) *m/z*: $[M+H]^+$ calculated for $C_{12}H_{16}NO_2^+$ 206.1176; found 206.1181.

***N,N*-Bis(4-methoxybenzyl)-2-methyl-3-oxopropanamide (2j)**

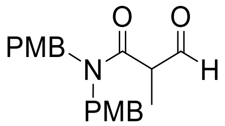

With **1j** (1.00 g, 3.2 mmol), ethyl formate (1.4 mL, 19 mmol), $TiCl_4$ (1.1 mL, 9.6 mmol), and NEt_3 (1.6 mL, 11 mmol). Chromatography on silica gel with EtOAc/hexane (10–30%) afforded **2j** as a brown oil (0.82 g, 75%). $\mathbf{R_f}$ = 0.13 (3:7 EtOAc: hexane); **IR** (film, ν_{max}/cm^{-1}) 1725, 1620; **^{1}H NMR** (400 MHz, $CDCl_3$) δ 9.69 (d, *J* = 2.0 Hz, 1H), 7.14 (d, *J* = 8.7 Hz, 2H), 7.05 (d, *J* = 8.7 Hz, 2H), 6.90 (d, *J* = 8.7 Hz, 2H), 6.85 (d, *J* = 8.7 Hz, 2H), 4.62 – 4.31 (m, 4H), 3.81 (s, 3H), 3.80 (s, 3H), 3.58 (qd, *J* = 7.0, 2.0 Hz, 1H), 1.43 (d, *J* = 7.0 Hz, 3H); **^{13}C NMR** (101 MHz, $CDCl_3$) δ 199.0, 170.7, 159.4, 159.2, 129.7, 129.0, 127.9, 127.7, 114.6, 114.2, 55.5, 55.4, 49.9, 49.4, 47.7, 12.6; **HRMS** (ESI$^+$) *m/z*: $[M+Na]^+$ calculated for $C_{20}H_{23}NNaO_4^+$ 364.1525; found 364.1519.

Benzyl 4-(*N*-benzyl-2-methyl-3-oxopropanamido)benzoate (2k)

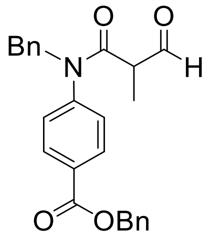

With **1k** (1.80 g, 4.7 mmol), methyl formate (1.8 mL, 28.3 mmol), $TiCl_4$ (1.6 mL, 14.1 mmol), and NEt_3 (2.0 mL, 14.1 mmol). Chromatography on silica gel with EtOAc/hexane (15–30%) afforded **2k** as a yellow oil (1.50 g, 80%). $\mathbf{R_f}$ = 0.19 (3:7 EtOAc: hexane); **IR** (film, ν_{max}/cm^{-1}) 1731, 1717, 1646; **^{1}H NMR** (300 MHz, $CDCl_3$) δ 9.58 (d, *J* = 1.7 Hz, 1H), 8.04 (d, *J* = 8.5 Hz, 2H), 7.46 – 7.27 (m, 6H), 7.26 – 7.13 (m, 4H), 7.06 (d, *J* = 8.5 Hz, 2H), 5.35 (s, 2H), 4.97 (d, *J* = 14.3 Hz, 1H), 4.89 (d, *J* = 14.3 Hz, 1H), 3.21 (app q, *J* = 7.0 Hz, 1H), 1.33 (d, *J* = 7.0 Hz, 3H); **^{13}C NMR** (151 MHz, $CDCl_3$) δ 198.3, 169.4, 165.2, 145.4, 136.4, 135.6, 131.2, 128.7, 128.6, 128.5, 128.5, 128.4, 128.4, 128.2, 127.7, 67.0, 53.1, 50.7, 12.4; **HRMS** (ESI$^+$) *m/z*: $[M+H]^+$ calculated for $C_{25}H_{24}NO_4^+$ 402.1705; found 402.1700.

N-Benzyl-2-methyl-N-(4-morpholinophenyl)-3-oxopropanamide (2l)

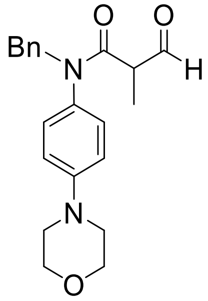

With **1l** (1.00 g, 3.1 mmol), methyl formate (1.1 mL, 18.5 mmol), $TiCl_4$ (1.0 mL, 9.3 mmol), and NEt_3 (1.5 mL, 10.8 mmol). Chromatography on silica gel with EtOAc/hexane (10–40%) afforded **2l** as a white solid (0.90 g, 83%). **R_f** = 0.11 (3:7 EtOAc: hexane); **IR** (film, ν_{max}/cm^{-1}) 1722, 1637; **^{1}H NMR** (300 MHz, $CDCl_3$) δ 9.61 (d, *J* = 1.6 Hz, 1H), 7.31 – 7.20 (m, 5H), 6.88 – 6.80 (m, 4H), 4.92 (d, *J* = 14.1 Hz, 1H), 4.85 (d, *J* = 14.1 Hz, 1H), 3.87 – 3.84 (m, 4H), 3.33 (qd, *J* = 7.1, 1.6 Hz, 1H), 3.18 – 3.15 (m, 4H), 1.32 (d, *J* = 7.1 Hz, 3H); **^{13}C NMR** (151 MHz, $CDCl_3$) δ 198.9, 170.4, 150.9, 137.2, 132.8, 129.2, 128.9, 128.5, 127.5, 115.8, 66.7, 53.3, 50.5, 48.6, 12.5; **HRMS** (ESI$^+$) *m/z*: [M+H]$^+$ calculated for $C_{21}H_{25}N_2O_3^+$ 353.1860; found 353.1867.

3-(Indolin-1-yl)-2-methyl-3-oxopropanal (2m)

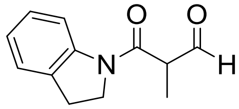

With **1m** (3.50 g, 20.0 mmol), methyl formate (7.4 mL, 120 mmol), $TiCl_4$ (6.6 mL, 60 mmol), and NEt_3 (11.0 mL, 78.9 mmol). Chromatography on silica gel with EtOAc/hexane (20–40%) afforded **2m** as a brown solid (4.00 g, 99%). **R_f** = 0.21 (3:7 EtOAc: hexane); **IR** (film, ν_{max}/cm^{-1}) 1721, 1646; **^{1}H NMR** (300 MHz, $CDCl_3$) δ 9.70 (d, *J* = 2.4 Hz, 1H), 8.23 (d, *J* = 8.1 Hz, 1H), 7.24 – 7.15 (m, 2H), 7.20 (td, *J* = 7.5 Hz, *J* = 0.9 Hz, 1H), 4.23 – 4.02 (m, 2H), 3.56 (qd, *J* = 7.0 Hz, 2.4 Hz, 1H), 3.21 (app t, *J* = 8.4 Hz, 1H), 1.49 (d, *J* = 7.0 Hz, 1H); **^{13}C NMR** (151 MHz, $CDCl_3$) δ 198.7, 167.7, 142.7, 131.4, 127.8, 124.8, 124.5, 117.5, 52.9, 48.3, 28.1, 12.0; **HRMS** (ESI$^+$) *m/z*: [M+H]$^+$ calculated for $C_{12}H_{14}NO_2^+$ 204.1025; found 204.1027.

2-Formyl-N-methyl-N-phenylbutanamide[2] (2n)

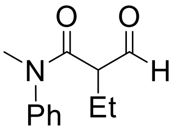

With **1n** (3.00 g, 16.9 mmol), methyl formate (6.3 mL, 101.6 mmol), $TiCl_4$ (5.6 mL, 50.8 mmol), and NEt_3 (8.3 mL, 59.3 mmol). Chromatography on silica gel with EtOAc/hexane (10–30%) afforded **2n** as a yellow oil (3.10 g, 94%). **R_f** = 0.32 (3:7 EtOAc: hexane); **^{1}H NMR** (300 MHz, $CDCl_3$) δ 9.52 (d, *J* = 2.6 Hz, 1H), 7.42 – 7.31 (m, 3H), 7.15 – 7.09 (m, 2H), 3.28 (s, 3H), 3.10

(ddd, J = 8.6, 6.3, 2.6 Hz, 1H), 1.95 – 1.70 (m, 2H), 0.81 (t, J = 7.4 Hz, 3H); **^{13}C NMR** (151 MHz, $CDCl_3$) δ 199.2, 168.8, 143.0, 129.0, 128.2, 127.4, 57.8, 37.4, 21.6, 11.5. All recorded data were in accordance with those previously reported.[2]

3-Cyclopentyl-2-formyl-*N*-methyl-*N*-phenylpropanamide[2] (2o)

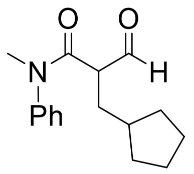

With **1o** (1.00 g, 4.3 mmol), methyl formate (1.6 mL, 26.0 mmol), $TiCl_4$ (1.4 mL, 13.0 mmol), and NEt_3 (2.1 mL, 15.1 mmol). Chromatography on silica gel with EtOAc/hexane (10–20%) afforded **2o** as a yellow oil (0.83 g, 74%). **R_f** = 0.29 (3:7 EtOAc: hexane); **^{1}H NMR** (300 MHz, $CDCl_3$) δ 9.54 (d, J = 2.7 Hz, 1H), 7.46 – 7.34 (m, 3H), 7.16 (d, J = 7.4 Hz, 2H), 3.30 – 3.21 (m, 4H), 1.98 – 1.89 (m, 1H), 1.78 – 1.40 (m, 8H), 0.92 – 0.82 (m, 2H); **^{13}C NMR** (151 MHz, $CDCl_3$) δ 199.6, 169.2, 143.2, 130.1, 128.5, 127.6, 56.2, 37.8, 37.7, 34.5, 32.6, 32.5, 25.1, 25.1. All recorded data were in accordance with those previously reported.[2]

2-Formyl-*N*-methyl-*N*-phenylpent-4-enamide (2p)

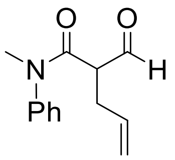

With **1p** (1.00 g, 5.3 mmol), ethyl formate (2.3 mL, 31.7 mmol), $TiCl_4$ (1.7 mL, 16.0 mmol), and NEt_3 (2.6 mL, 18.6 mmol). Chromatography on silica gel with EtOAc/hexane (10–30%) afforded **2p** as a brown oil (0.91 g, 79%). **R_f** = 0.20 (3:7 EtOAc: hexane); **IR** (film, ν_{max}/cm^{-1}) 1726, 1625; **^{1}H NMR** (400 MHz, $CDCl_3$) δ 9.48 (d, J = 2.2 Hz, 1H), 7.48 – 7.27 (m, 3H), 7.22 – 7.09 (m, 2H), 5.72 – 5.49 (m, 1H), 5.09 – 4.90 (m, 2H), 3.37 – 3.19 (m, 4H), 2.70 – 2.54 (m, 1H), 2.54 – 2.38 (m, 1H); **^{13}C NMR** (101 MHz, $CDCl_3$) δ 198.2, 168.1, 142.9, 133.7, 130.0, 128.4, 127.6, 117.7, 55.9, 37.5, 32.0; **HRMS** (ESI$^+$) *m/z*: [M+Na]$^+$ calculated for $C_{13}H_{15}NNaO_2^+$ 240.1000; found 240.0995.

2-Formyl-*N*-methyl-*N*-phenylhexanamide (2q)

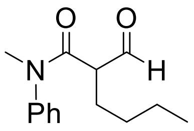

With **1q** (1.00 g, 5.0 mmol), methyl formate (1.8 mL, 29.2 mmol), $TiCl_4$ (1.6 mL, 14.6 mmol), and NEt_3 (2.4 mL, 17.2 mmol). Chromatography on silica gel with EtOAc/hexane (10–30%) afforded **2q** as a brown oil (0.87 g, 75%). **R_f** = 0.23 (1:4 EtOAc: hexane); **IR** (film, ν_{max}/cm^{-1}) 1725; 1642; **^{1}H NMR** (400 MHz, $CDCl_3$) δ 9.53 (d, J = 2.7 Hz, 1H), 7.45 – 7.33 (m, 3H), 7.24 – 7.10 (m, 2H),

3.32 – 3.16 (m, 4H), 1.94 – 1.85 (m, 1H), 1.77 – 1.68 (m, 1H), 1.25 – 1.07 (m, 4H), 0.82 (t, J = 7.1 Hz, 3H); **^{13}C NMR** (101 MHz, $CDCl_3$) δ 199.5, 169.1, 143.2, 130.1, 128.5, 127.6, 56.5, 37.6, 29.2, 28.0, 22.4, 13.8; **HRMS** (ESI$^+$) *m/z*: $[M+Na]^+$ calculated for $C_{14}H_{19}NNaO_2^+$ 256.1313; found 256.1308.

4-(Benzyloxy)-2-formyl-*N*-methyl-*N*-phenylbutanamide[2] (2r)

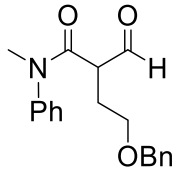

With **1r** (0.50 g, 1.8 mmol), ethyl formate (0.80 mL, 10.6 mmol), $TiCl_4$ (0.60 mL, 5.30 mmol), and NEt_3 (0.90 mL, 6.3 mmol). Chromatography on silica gel with EtOAc/hexane (10–30%) afforded **2r** as a brown oil (0.37 g, 66%). **R_f** = 0.21 (1:4 EtOAc: hexane); **^{1}H NMR** (400 MHz, $CDCl_3$) δ 9.49 (d, J = 1.9 Hz, 1H), 7.34 – 7.26 (m, 6H), 7.23 – 7.19 (m, 2H), 7.17 – 7.09 (m, 2H), 4.45 – 4.34 (m, 2H), 3.59 – 3.39 (m, 4H), 3.27 (s, 3H), 2.27 – 2.02 (m, 2H); **^{13}C NMR** (101 MHz, $CDCl_3$) δ 198.3, 168.3, 143.0, 138.1, 129.9, 128.3, 127.5, 127.5, 127.5, 127.4, 72.7, 67.4, 53.9, 37.5, 27.7. All recorded data were in accordance with those previously reported.[2]

2-Benzyl-*N*-methyl-3-oxo-*N*-phenylpropanamide[2] (2s)

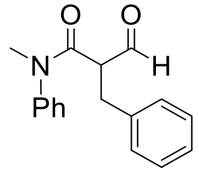

With **1s** (2.00 g, 8.4 mmol), methyl formate (3.1 mL, 50.4 mmol), $TiCl_4$ (2.8 mL, 25.2 mmol), and NEt_3 (4.1 mL, 29.4 mmol). Chromatography on silica gel with EtOAc/hexane (10-30%) afforded **2s** as a yellow oil (0.90 g, 40%). **R_f** = 0.21 (3:7 EtOAc: hexane); **^{1}H NMR** (400 MHz, $CDCl_3$) δ 9.67 (d, J = 1.9 Hz, 1H), 7.34 – 7.26 (m, 7H), 7.08 – 7.00 (m, 2H), 6.63 (s, 1H), 3.53 – 3.48 (m, 1H), 3.23 (s, 3H), 3.30 – 3.17 (m, 1H), 3.08-3.00 (m, 1H); **^{13}C NMR** (101 MHz, $CDCl_3$) δ 198.6, 168.6, 143.2 (143.5), 138.1, 130.3, 129.5 (129.6), 129.0, 128.7 (128.8), 127.9 (128.2), 127.2, 59.0, 37.8, 34.7. All recorded data were in accordance with those previously reported.[2]

1-(4-Methoxybenzyl)-2-oxopiperidine-3-carbaldehyde (2t)

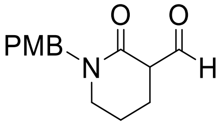

With **1t** (1.00 g, 4.7 mmol), methyl formate (1.6 mL, 27.4 mmol), $TiCl_4$ (1.5 mL, 13.7 mmol), and NEt_3 (2.2 mL, 16.0 mmol). Chromatography on silica gel with EtOAc/hexane (10–30%) afforded **2t** as a brown oil (0.52 g, 45%). **R_f** = 0.48 (2:3 EtOAc: hexane); **IR** (film, ν_{max}/cm^{-1}) 1727, 1643;

^{1}**H NMR** (400 MHz, $CDCl_3$) (in enol form) δ 7.20 (d, *J* = 8.8 Hz, 2H), 6.97 – 6.92 (m, 1H), 6.86 (d, *J* = 8.8 Hz, 2H), 4.55 (s, 2H), 3.80 (s, 3H), 3.24 – 3.18 (m, 2H), 2.35 – 2.29 (m, 2H), 1.80 – 1.72 (m, 2H); ^{13}**C NMR** (101 MHz, $CDCl_3$) δ 169.6, 159.2, 158.1, 129.5, 129.1, 114.2, 101.1, 55.4, 49.0, 46.7, 24.7, 23.0; **HRMS** (ESI^+) *m/z*: $[M+Na]^+$ calculated for $C_{14}H_{17}NNaO_3^+$ 270.1106; found 270.1101.

***N*-(3-Bromophenyl)-2-formyl-*N*-methyl-3-(4-nitrophenyl)propanamide (2u)**

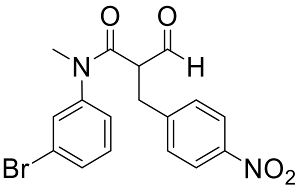

With **1u** (1.00 g, 2.8 mmol), methyl formate (1.0 mL, 16.8 mmol), $TiCl_4$ (0.9 mL, 8.4 mmol), and NEt_3 (1.3 mL, 9.8 mmol). Chromatography on silica gel with EtOAc/hexane (20–60%) afforded **2u** as a yellow oil (0.80 g, 75%). $\mathbf{R_f}$ = 0.21 (1:1 EtOAc: hexane); **IR** (film, ν_{max}/cm^{-1}) 1731, 1636; ^{1}**H NMR** (300 MHz, $CDCl_3$) δ 9.59 (d, *J* = 1.6 Hz, 1H), 8.17 – 8.10 (m, 2H), 7.43 (ddd, *J* = 8.1, 1.9, 1.0 Hz, 1H), 7.26 – 7.11 (m, 3H), 6.77 – 6.53 (m, 2H), 3.50 (ddd, *J* = 10.6, 4.4, 1.6 Hz, 1H), 3.37 – 3.27 (m, 1H), 3.16 (s, 3H), 3.12 – 3.06 (m, 1H); ^{13}**C NMR** (101 MHz, $CDCl_3$) δ 196.0, 166.9, 146.7, 145.1, 143.3, 131.5, 131.0, 130.2, 129.7, 125.8, 123.6, 122.9, 57.6, 37.2, 33.3; **HRMS** (ESI^+) *m/z*: $[M+H]^+$ calculated for $C_{17}H_{16}BrN_2O_4^+$ 391.0288; found 391.0293.

....

5. General procedure for enantioselective hydrazination of α-formyl amides 2 into 3

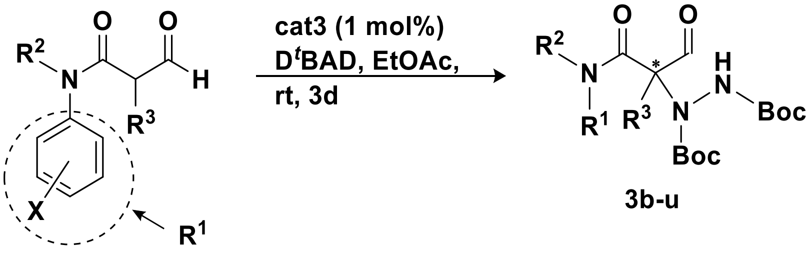

2b-u **3b-u**

To a solution of **2** (1.0 eq) in ethyl acetate (1 mL) was added di-*tert*-butyl azodicarboxylate (D^{t}BAD) (1.2 eq) followed by (8α, 9*S*)-6'-methoxycinchonan-9-amine trihydrochloride (**cat3**, 1 mol%), and the solution stirred at room temperature for 36 h. The reaction mixture was then quenched with H_2O and the organic layer separated. The aqueous phase was extracted with ethyl acetate (x 3) and the combined organic extracts were dried over $MgSO_4$ and filtered. The solvent was removed under reduced pressure and the crude product was purified by flash column chromatography using ethyl acetate/hexane mixtures as eluent. The NMR spectra revealed that compounds **3** existed as rotamers due to the hydrazide moiety. For the NMR data, minor rotamers are reported in brackets.

Characterization data for 3a-u

Di-*tert*-butyl (*R*)-1-(2-methyl-1-(methyl(phenyl)amino)-1,3-dioxopropan-2-yl)hydrazine-1,2-dicarboxylate (3a)

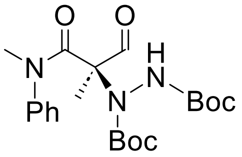

With **2a** (0.100 g, 0.523 mmol), D^{t}BAD (0.145 g, 0.630 mmol) and **cat3** (0.002 g, 0.005 mmol). Chromatography on silica gel with EtOAc/hexane (10–30%) afforded **3a** as a colorless solid (0.193 g, 88%). **R$_f$** = 0.38 (3:7 EtOAc: hexane); **IR** (film, ν_{max}/cm^{-1}) 1723, 1704; **^{1}H NMR** (300 MHz, $CDCl_3$) δ 9.84 (9.91, 9.81, 9.74) (s, 1H), 7.50 – 7.40 (m, 3H), 7.26 – 7.22 (m, 2H), 4.21 (6.30, 4.99, 4.43) (s, 1H), 3.27 (3.89, 3.68, 3.59) (s, 3H), 1.47, 1.37, (1.76, 1.67, 1.53) (2 x s, 18H), 1.41 (s, 3H); **^{13}C NMR** (101 MHz, $CDCl_3$) δ 192.8 (197.0, 193.7, 191.8), 170.2 (170.9, 169.8), 154.9, 154.4, 142.6, 130.0, 128.9, 127.7, (129.9, 128.6, 128.0), 82.9, 82.4, (84.0, 81.5), 71.1 (72.3, 72.2, 72.0), 40.1 (38.5), 28.3, 28.1, 19.1 (19.5); **HRMS** (ESI$^+$) *m/z*: [M+H]$^+$ calculated for $C_{21}H_{32}N_3O_6^+$ 422.2286; found 422.2293.

The ^{1}H VT NMR at 353K is provided in the appendix.

$[\alpha]_D^{20}$ = +11.5 (*c* =1.0, $CHCl_3$).

HPLC: The enantiomeric excess (98%) was determined by HPLC analysis using a Daicel Chiralpak IA column: *n*-hexane: isopropanol = 90:10, flow rate = 1.0 mL/min, λ = 254 nm, R_t (minor) = 9.337 min, R_t (major) = 12.053 min.

LARGE SCALE: With **2a** (1.00 g, 5.23 mmol), D^{t}BAD (1.50 g, 6.51 mmol) and **cat3** (0.023 g, 0.052 mmol) for 18d. Chromatography on silica gel with EtOAc/hexane (10–30%) afforded **3a** as a colorless solid (1.80 g, 82%, 98% *ee*).

Di-*tert*-butyl (*R*)-1-(1-((4-methoxyphenyl)(methyl)amino)-2-methyl-1,3-dioxopropan-2-yl)hydrazine-1,2-dicarboxylate (3b)

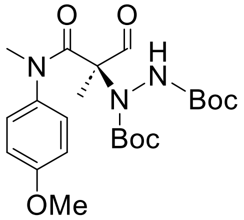

With **2b** (0.100 g, 0.452 mmol), D^{t}BAD (0.124 g, 0.538 mmol) and **cat3** (0.002 g, 0.005 mmol). Chromatography on silica gel with EtOAc/hexane (10–30%) afforded **3b** as a colorless wax (0.136 g, 67%). **R$_f$** = 0.21 (3:7 EtOAc: hexane); **IR** (film, ν_{max}/cm^{-1}) 1705, 1646; **^{1}H NMR** (300 MHz, $CDCl_3$) δ 9.72 (9.83, 9.77, 9.62) (s, 1H), 7.13 – 7.04 (m, 2H), 6.92 – 6.86 (m, 2H), 4.63 (6.31, 5.37, 4.22) (s, 1H), 3.80 (3.79, 3.77, 3.75) (s, 3H), 3.20 (3.35, 3.34, 3.33) (s, 3H), 1.45, 1.35, (2.07 – 1.66) (3 x s, 21H); **^{13}C NMR** (101 MHz, $CDCl_3$) δ 191.9 (193.0, 192.8, 190.7), 170.0

(169.6), 159.3, 154.6, 154.2, 134.5, 128.7 (129.1), 115.7 – 113.4, 82.5, 82.1, (83.7, 81.2), 71.0 (72.2, 71.8, 71.4), 55.4, 39.9, 28.0, 27.8, 18.6; **HRMS** (ESI^+) *m/z*: $[M+H]^+$ calculated for $C_{22}H_{34}N_3O_7^+$ 452.2391; found 452.2398.

[α]$_D^{20}$ = +66.9 (*c* =1.0, $CHCl_3$).

HPLC: The enantiomeric excess (98%) was determined by HPLC analysis using a Daicel Chiralpak IA column: *n*-hexane: isopropanol = 95:5, flow rate = 1.0 mL/min, λ = 254 nm, R_t (minor) = 19.768 min, R_t (major) = 25.513 min.

Di-*tert*-butyl (*R*)-1-(1-((4-chlorophenyl)(methyl)amino)-2-methyl-1,3-dioxopropan-2-yl)hydrazine-1,2-dicarboxylate (3c)

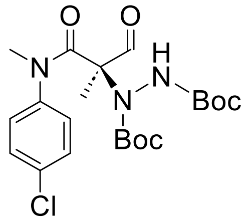

With **2c** (0.100 g, 0.443 mmol), D^{t}BAD (0.123 g, 0.534 mmol) and **cat3** (0.002 mg, 0.005 mmol). Chromatography on silica gel with EtOAc/hexane (10–30%) afforded **3c** as a colorless sticky solid (0.170 g, 85%). **R$_f$** = 0.36 (3:7 EtOAc: hexane); **IR** (film, ν_{max}/cm^{-1}) 1705, 1639; **^{1}H NMR** (300 MHz, $CDCl_3$) δ 9.69 (9.79, 9.62, 9.55) (s, 1H), 7.42 – 7.34 (m, 2H), 7.22 – 7.09 (m, 2H), 6.29 (5.54, 4.81, 4.24) (s, 1H), 3.24 (3.76, 3.64, 3.50) (s, 3H), 1.48, 1.39, (1.75. 1.67) (3 x s, 21H); **^{13}C NMR** (101 MHz, $CDCl_3$) δ 191.7 (196.8, 192.9, 190.9), 169.7 (170.3, 169.4), 156.5 – 154.4, 141.0 (142.8, 141.3), 130.1, 129.5, 129.2, (134.6, 132.6, 128.0), 83.1, 81.7, (84.3, 82.7), 71.6 (72.8, 72.3, 72.2), 40.2 (38.4), 28.2, 28.1, 18.5 (16.2); **HRMS** (ESI^+) *m/z*: $[M+H]^+$ calculated for $C_{21}H_{31}ClN_3O_6^+$ 456.1896; found 456.1901.

[α]$_D^{20}$ = +77.7 (*c* =1.0, $CHCl_3$).

HPLC: The enantiomeric excess (98%) was determined by HPLC analysis using a Daicel Chiralpak IA column: *n*-hexane: isopropanol = 90:10, flow rate = 1.0 mL/min, λ = 254 nm, R_t (minor) = 9.791 min, R_t (major) = 12.637 min.

Di-*tert*-butyl (*R*)-1-(1-((4-bromophenyl)(methyl)amino)-2-methyl-1,3-dioxopropan-2-yl)hydrazine-1,2-dicarboxylate (3d)

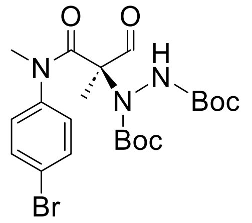

With **2d** (0.100 g, 0.370 mmol), D^{t}BAD (0.103 g, 0.447 mmol) and **cat3** (0.002 g, 0.005 mmol). Chromatography on silica gel with EtOAc/hexane (10–30%) afforded **3d** as a colorless sticky solid

(0.166 g, 90%). **R$_f$** = 0.35 (3:7 EtOAc: hexane); **IR** (film, ν_{max}/cm^{-1}) 1708, 1643; **^{1}H NMR** (300 MHz, $CDCl_3$) δ 9.67 (9.75) (s, 1H), 7.55 (d, *J* = 8.2 Hz, 2H), 7.11 (d, *J* = 8.2 Hz, 2H), 6.28 (5.57, 4.93, 4.38) (s, 1H), 3.22 (3.62, 3.45) (s, 3H), 1.46, 1.38, (1.80, 1.73) (3 x s, 21H); **^{13}C NMR** (151 MHz, $CDCl_3$) δ 191.6 (196.5, 192.3, 191.0), 169.6 (170.1, 169.3), 156.7 – 154.3, 141.9 (143.4, 141.7), 133.0 (132.5), 129.5 (128.4), 122.4 (120.5), 83.1, 81.6, (84.2, 82.6), 72.7 (73.5, 72.3, 71.6), 40.0 (38.3), 28.2, 28.0, 18.5 (16.2); **HRMS** (ESI$^+$) *m/z*: [M+H]$^+$ calculated for $C_{21}H_{31}BrN_3O_6^+$ 500.1391; found 500.1395.

[α]$_D^{20}$ = +70.6 (*c* =1.0, $CHCl_3$).

HPLC: The enantiomeric excess (98%) was determined by HPLC analysis using a Daicel Chiralpak IA column: *n*-hexane: isopropanol = 90:10, flow rate = 1.0 mL/min, λ = 254 nm, R_t (minor) = 10.536 min, R_t (major) = 13.862 min.

Di-*tert*-butyl (*R*)-1-(1-((4-cyanophenyl)(methyl)amino)-2-methyl-1,3-dioxopropan-2-yl)hydrazine-1,2-dicarboxylate (3e)

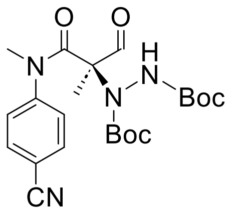

With **2e** (0.100 g, 0.463 mmol), D^{t}BAD (0.128 g, 0.556 mmol) and **cat3** (0.002 g, 0.005 mmol). Chromatography on silica gel with EtOAc/hexane (10–30%) afforded **3e** as a colorless sticky solid (0.154 g, 75%). **R$_f$** = 0.23 (3:7 EtOAc: hexane); **IR** (film, ν_{max}/cm^{-1}) 1708, 1637; **^{1}H NMR** (300 MHz, DMSO-*d*$_6$) δ 9.50 (9.72, 9.43, 9.41) (s, 1H), 7.97 – 7.75 (m, 2H), 7.62 – 7.33 (m, 2H), 3.29 (3.32, 3.30, 3.23, 3.16) (s, 3H), 1.42, 1.38, (1.45, 1.37, 1.33, 1.32) (3 x s, 21H); **^{13}C NMR** (101 MHz, DMSO-*d*$_6$) δ 190.4 (192.6, 1912.0, 190.9), 169.0 (169.5, 167.2), 156.2 – 151.2, 148.0 (148.7), 133.3, 133.1, 127.5, 118.67, (133.6, 126.7), 108.2 (109.4, 108.9), 83.0 – 78.4, 70.2 (71.4), 37.0 (37.8, 37.4), 28.0, 27.7, (28.1, 27.5), 14.2 (15.0, 13.7). **HRMS** (ESI$^+$) *m/z*: [M+H]$^+$ calculated for $C_{22}H_{31}N_4O_6^+$ 447.2238; found 447.2240.

[α]$_D^{20}$ = +70.1 (*c* =1.0, $CHCl_3$).

HPLC: The enantiomeric excess (98%) was determined by HPLC analysis using a Daicel Chiralpak IA column: *n*-hexane: isopropanol = 90:10, flow rate = 1.0 mL/min, λ = 254 nm, R_t (minor) = 21.852 min, R_t (major) = 27.585 min.

Di-*tert*-butyl (*R*)-1-(1-((2-bromophenyl)(methyl)amino)-2-methyl-1,3-dioxopropan-2-yl)hydrazine-1,2-dicarboxylate (3f)

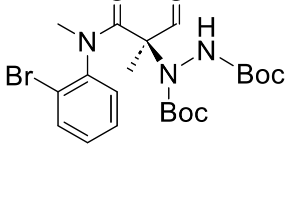

With **2f** (0.100 g, 0.370 mmol), D^{t}BAD (0.099 g, 0.430 mmol) and **cat3** (0.002 g, 0.005 mmol). Chromatography on silica gel with EtOAc/hexane (5–20%) afforded **3f** as a white solid (0.106 g, 59%). **R$_f$** = 0.36 (3:7 EtOAc: hexane); **IR** (film, ν_{max}/cm^{-1}) 1707, 1640; **^{1}H NMR** (300 MHz, $CDCl_3$) δ 9.80 (9.89, 9.18, 9.15) (s, 1H), 7.78 – 7.54 (m, 1H), 7.50 – 7.10 (m, 3H), 6.63 (6.42, 6.02, 4.28) (s, 1H), 3.22 (3.76, 3.61, 3.48) (s, 3H), 1.51, 1.48, (1.91, 1.81, 1.70, 1.68, 1.40, 1.37) (2 x s, 18H), 1.35 (1.25) (s, 3H); **^{13}C NMR** (101 MHz, $CDCl_3$) δ 197.1 (197.8, 196.7, 193.7, 193.4, 192.8, 191.1, 189.4, 189.0), 170.6 – 168.4, 156.8 – 154.1, 143.2 – 140.3, 133.3, 129.4, 129.3, 129.0, 128.6, (134.1, 133.9, 131.8, 131.5, 131.1, 130.9, 130.5, 130.2, 124.3, 123.4, 122.3), 85.1 – 80.4 , 75.9 – 70.5, 39.8 – 36.8, 28.3, 28.0, 19.2 – 15.6; **HRMS** (ESI$^+$) *m/z*: [M+H]$^+$ calculated for $C_{21}H_{31}BrN_3O_6^+$ 500.1391; found 500.1396.

[α]$_D$^{20} = +17.9 (*c* =1.0, $CHCl_3$).

HPLC: The enantiomeric excess (98%) was determined by HPLC analysis using a Daicel Chiralpak IA column: *n*-hexane: isopropanol = 90:10, flow rate = 1.0 mL/min, λ = 254 nm, R$_t$ (minor) = 15.467 min, R$_t$ (major) = 20.324 min.

Di-*tert*-butyl (*R*)-1-(1-(allyl(phenyl)amino)-2-methyl-1,3-dioxopropan-2-yl)hydrazine-1,2-dicarboxylate (3g)

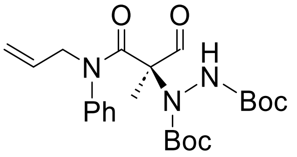

With **2g** (0.100 g, 0.460 mmol), D^{t}BAD (0.127 g, 0.55 mmol) and **cat3** (0.002 g, 0.005 mmol). Chromatography on silica gel with EtOAc/hexane (10–20%) afforded **3g** as a colorless, sticky solid (0.175 g, 85%). **R$_f$** = 0.32 (3:7 EtOAc: hexane); **IR** (film, ν_{max}/cm^{-1}) 1708, 1642; **^{1}H NMR** (300 MHz, $CDCl_3$) δ 9.85 (9.94, 9.76) (s, 1H), 7.46 – 7.38 (m, 3H), 7.21 – 7.18 (m, 2H), 5.93 – 5.84 (m, 1H), 5.15 – 5.05 (m, 2H), 4.33 – 4.21 (m, 2H), 1.48, 1.37, (1.75, 1.56) (2 x s, 18H), 1.40 (s, 3H); **^{13}C NMR** (101 MHz, $CDCl_3$) δ 192.5 (193.7, 191.4), 169.5, 154.5, 154.0, 140.5, 132.1, 129.4, 128.6, 128.4, 118.3 (119.1), 82.5, 82.0, (81.1), 70.8 (71.7), 54.6, 27.9, 27.7, 18.7; **HRMS** (ESI$^+$) *m/z*: [M+H]$^+$ calculated for $C_{23}H_{34}N_3O_6^+$ 448.2442; found 448.2448.

[α]$_D$^{20} = +85.5 (*c* =1.0, $CHCl_3$).

HPLC: The enantiomeric excess (99%) was determined by HPLC analysis using a Daicel Chiralpak IA column: *n*-hexane: isopropanol = 90:10, flow rate = 1.0 mL/min, λ = 254 nm, R$_t$ (minor) = 7.172 min, R$_t$ (major) = 8.855 min.

Di-*tert*-butyl (*R*)-1-(1-(benzyl(phenyl)amino)-2-methyl-1,3-dioxopropan-2-yl)hydrazine-1,2-dicarboxylate (3h; X-ray achieved)

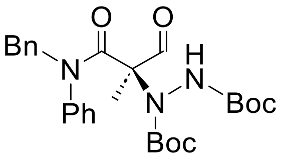

With **2h** (0.100 g, 0.374 mmol), D^{t}BAD (0.103 g, 0.447 mmol) and **cat3** (0.002 g, 0.005 mmol). Chromatography on silica gel with EtOAc/hexane (5–10%) afforded **3h** as a colorless, sticky solid (0.133 g, 72%). **R$_f$** = 0.39 (3:7 EtOAc: hexane); **IR** (film, ν_{max}/cm^{-1}) 1704, 1633; **M.P.** 173 – 177 °C (chloroform/hexane); ^{1}**H NMR** (300 MHz, $CDCl_3$) δ 9.92 (9.99, 9.82) (s, 1H), 7.39 – 7.17 (m, 8H), 6.96 – 6.92 (m, 2H), 4.92 – 4.82 (m, 2H), 4.13 (4.26, 3.69) (s, 1H), 1.46, 1.36, (1.76, 1.67, 1.57) (2 x s, 18H), 1.39 (s, 3H); ^{13}**C NMR** (101 MHz, $CDCl_3$) δ 192.8 (194.0, 191.9), 170.3, 154.8, 154.3, 140.5, 136.8, 129.6, 129.1, 128.9, 128.5, 127.9, 127.6, 82.8, 82.4, (84.1, 81.4), 71.2 (72.0), 55.3 (55.7), 28.2, 28.0, 18.9 (19.8); **HRMS** (ESI$^+$) *m/z*: $[M+H]^+$ calculated for $C_{27}H_{36}N_3O_6^+$ 498.2599; found 498.2602.

[α]$_D$^{20} = +61.7 (*c* =1.0, $CHCl_3$).

HPLC: The enantiomeric excess (99%) was determined by HPLC analysis using a Daicel Chiralpak IA column: *n*-hexane: isopropanol = 90:10, flow rate = 1.0 mL/min, λ = 254 nm, R_t (minor) = 10.638 min, R_t (major) = 11.564 min.

Di-*tert*-butyl (*R*)-1-(1-(benzyl(methyl)amino)-2-methyl-1,3-dioxopropan-2-yl)hydrazine-1,2-dicarboxylate (3i)

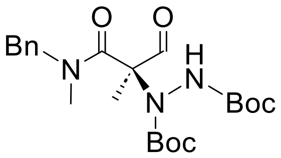

With **2i** (0.100 g, 0.487 mmol), D^{t}BAD (0.135 g, 0.586 mmol) and **cat3** (0.002 g, 0.005 mmol). Chromatography on silica gel with EtOAc/hexane (10–30%) afforded **3i** as a white solid (0.201 g, 94%). **R$_f$** = 0.25 (3:7 EtOAc: hexane); **IR** (film, ν_{max}/cm^{-1}) 1708, 1624; ^{1}**H NMR** (300 MHz, $CDCl_3$) δ 9.94 – 9.63 (m, 1H), 7.40 – 7.15 (m, 5H), 6.27 (6.51, 6.21, 5.99) (s, 1H), 6.11 – 4.39 (m, 2H), 3.28, 2.85, (3.69, 3.37, 3.14, 3.06, 2.95, 2.74) (s, 3H), 1.44 (1.76, 1.66, 1.60, 1.46, 1.41) (3 x s, 21H); ^{13}**C NMR** (151 MHz, $CDCl_3$) δ 197.1 (196.4, 191.8 – 190.7), 169.8, 156.4 – 155.2, 136.8, 128.5, 128.0, 127.6 – 126.8, 84.1 – 80.8, 73.9 – 71.4, 52.8 – 51.2, 35.9 – 33.4, 28.1, 27.8, (27.9), 17.6 – 15.6; **HRMS** (ESI$^+$) *m/z*: $[M+H]^+$ calculated for $C_{22}H_{34}N_3O_6^+$ 436.2442; found 436.2448.

[α]$_D$^{20} = +21.4 (*c* =1.0, $CHCl_3$).

HPLC: The enantiomeric excess (98%) was determined by HPLC analysis using a Daicel Chiralpak IA column: *n*-hexane: isopropanol = 90:10, flow rate = 1.0 mL/min, λ = 254 nm, R_t (minor) = 25.715 min, R_t (major) = 32.289 min.

Di-*tert*-butyl (*R*)-1-(1-(bis(4-methoxybenzyl)amino)-2-methyl-1,3-dioxopropan-2-yl)hydrazine-1,2-dicarboxylate (3j)

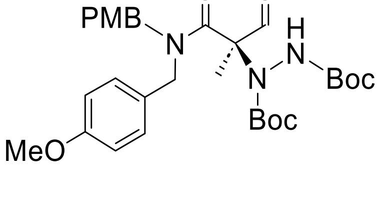

With **2j** (0.100 g, 0.293 mmol), D^{t}BAD (0.081 g, 0.352 mmol), and **cat3** (0.001 g, 0.002 mmol). Chromatography on silica gel with EtOAc/hexane (10–30%) afforded **3j** as a colorless sticky solid (0.095 g, 57%). **R$_f$** = 0.29 (3:7 EtOAc: hexane); **IR** (film, ν_{max}/cm^{-1}); 1739, 1702; **^{1}H NMR** (400 MHz, $CDCl_3$) δ 9.93 (9.84, 9.74) (s, 1H), 7.45 – 7.04 (m, 4H), 6.87 – 6.71 (m, 4H), 6.24 (6.55, 6.49, 5.98) (s, 1H), 5.62 – 4.21 (m, 4H), 3.80 (s, 3H), 3.77 (s, 3H), 1.84 – 1.19 (m, 21H); **^{13}C NMR** (101 MHz, $CDCl_3$) δ 197.0 (196.1, 191.4), 170.3 (169.9, 169.3), 159.0, 158.9 (159.1, 158.7), 156.0, 155.5, 130.2, 129.7, 128.7, 114.3, 114.0, 113.7, (127.7), 83.0, 81.2, (83.4, 81.6), 72.7 (73.5), 55.3 (55.3), 47.6, 46.5 (49.1, 47.3), 28.1, 28.0, 17.0 (17.3); **HRMS** (ESI$^+$) *m/z*: [M+Na]$^+$ calculated for $C_{30}H_{41}N_3NaO_8^+$ 594.2791; found 594.2786.

[α]$_D$^{21} = +25.5 (*c* =1.3, $CHCl_3$).

HPLC: The enantiomeric excess (96%) was determined by HPLC analysis using Daicel Chiralpak IA column: *n*-hexane: isopropanol = 80:20, flow rate = 1.0 mL/min, λ = 254 nm, R$_t$ (minor) = 11.751 min, R$_t$ (major) = 16.023 min.

Di-*tert*-butyl (*R*)-1-(1-(benzyl(4-((benzyloxy)carbonyl)phenyl)amino)-2-methyl-1,3-dioxopropan-2-yl)hydrazine-1,2-dicarboxylate (3k)

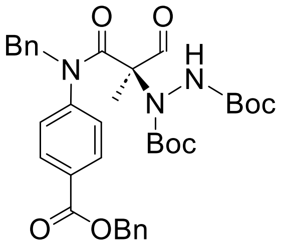

With **2k** (0.100 g, 0.249 mmol), D^{t}BAD (0.068 g, 0.295 mmol) and **cat3** (0.001 g, 0.002 mmol). Chromatography on silica gel with EtOAc/hexane (15–20%) afforded **3k** as a colorless solid (0.095 g, 60%). **R$_f$** = 0.28 (3:7 EtOAc: hexane); **IR** (film, ν_{max}/cm^{-1}) 1710, 1643; **^{1}H NMR** (300 MHz, $CDCl_3$) δ 9.82 (9.90, 9.75) (s, 1H), 8.02 (d, *J* = 8.2 Hz, 2H), 7.51 – 7.34 (m, 5H), 7.23 (s, 3H), 7.16 (s, 2H), 7.02 (d, *J* = 8.2 Hz, 2H), 5.35 (s, 2H), 5.02 – 4.72 (m, 2H), 4.48 (5.17, 4.66, 4.52) (s, 1H), 1.47, 1.36, (1.73, 1.68, 1.62) (3 x s, 21H); **^{13}C NMR** (101 MHz, $CDCl_3$) δ 192.5 (193.6, 191.8, 191.2), 170.0 (169.7), 165.7 (165.4), 157.1 – 154.2, 144.9 (145.1), 136.6 (135.9), 131.8 – 130.2, 129.4, 129.1, 128.9, 128.2, (129.6, 129.3, 128.3), 83.6, 83.1, (82.1, 81.3), 71.8 (73.0, 72.6), 67.7, 55.7, 28.5, 28.4, (28.6), 19.0; **HRMS** (ESI$^+$) *m/z*: [M+H]$^+$ calculated for $C_{35}H_{42}N_3O_8^+$ 632.2972; found 632.2968.

[α]$_D$^{20} = +46.5 (*c* =1.0, $CHCl_3$).

HPLC: The enantiomeric excess (98%) was determined by HPLC analysis using a Daicel Chiralpak AD column: *n*-hexane: isopropanol = 90:10, flow rate = 1.0 mL/min, λ = 254 nm, R$_t$ (major) = 11.059 min, R$_t$ (minor) = 16.005 min.

Di-*tert*-butyl (*R*)-1-(1-(benzyl(4-morpholinophenyl)amino)-2-methyl-1,3-dioxopropan-2-yl)hydrazine-1,2-dicarboxylate (3l)

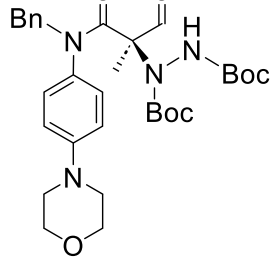

With **2l** (0.100 g, 0.284 mmol), D^{t}BAD (0.079 g, 0.343 mmol) and **cat3** (0.001 g, 0.002 mmol). Chromatography on silica gel with EtOAc/hexane (10–30%) afforded **3l** as a colorless, sticky solid (167 mg, 99%). **R$_f$** = 0.12 (3:7 EtOAc: hexane); **IR** (film, ν_{max}/cm^{-1}) 1706, 1638; **^{1}H NMR** (300 MHz, CDCl$_3$) δ 9.85 (9.99, 9.73) (s, 1H), 7.29 – 7.17 (m, 5H), 6.96 – 6.69 (m, 4H), 4.90 – 4.68 (m, 2H), 4.55 (5.18, 4.34) (s, 1H), 3.88 – 3.77 (m, 4H), 3.21 – 3.08 (m, 4H), 1.45, 1.37, (1.47, 1.40) (2 x s, 18H), 1.34 (s, 3H); **^{13}C NMR** (101 MHz, CDCl$_3$) δ 192.1 (193.9,192.8, 191.0), 170.3 (170.6), 154.7, 154.1, (154.5, 152.6), 150.8 (151.0,150.7, 150.6), 136.7 (136.5), 131.0 (131.2), 129.4, 128.8, 128.1, 127.2, (129.9, 129.1, 128.2, 127.5), 116.3 – 114.2, 82.3, 82.0, (83.7, 81.8, 81.0), 71.0 (71.9, 70.6), 66.4, 55.1 (55.4), 48.2 (48.5), 27.9, 27.8, 18.4 (19.3, 18.7); **HRMS** (ESI$^+$) *m/z*: [M+H]$^+$ calculated for $C_{31}H_{43}N_4O_7^+$ 583.3126; found 583.3132.

[α]$_D^{20}$ = +41.8 (*c* =1.0, CHCl$_3$).

HPLC: The enantiomeric excess (>99%) was determined by HPLC analysis using a Daicel Chiralpak IA column: *n*-hexane: isopropanol = 90:10, flow rate = 1.0 mL/min, λ = 254 nm, R$_t$ (major) = 22.869 min, R$_t$ (minor) = 27.631 min.

Di-*tert*-butyl (*R*)-1-(1-(indolin-1-yl)-2-methyl-1,3-dioxopropan-2-yl)hydrazine-1,2-dicarboxylate (3m; X-ray achieved)

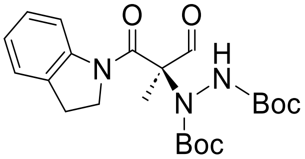

With **2m** (0.100 g, 0.492 mmol), D^{t}BAD (0.136 g, 0.591 mmol) and **cat3** (0.002 g, 0.005 mmol). Chromatography on silica gel with EtOAc/hexane (20–30%) afforded **3m** as a white solid (0.205 g, 97%). **R$_f$** = 0.26 (3:7 EtOAc: hexane); **IR** (film, ν_{max}/cm^{-1}) 1730, 1634; **M.P.** 165 – 169 °C (chloroform/hexane); **^{1}H NMR** (300 MHz, CDCl$_3$) δ 9.96 (9.78) (s, 1H), 8.20 (d, *J* = 8.0 Hz, 1H), 7.25 – 7.16 (m, 2H), 7.06 (td, *J* = 7.4, 1.1 Hz, 1H), 6.27 (6.47) (s, 1H), 4.74 – 3.92 (m, 2H), 3.27 – 3.02 (m, 2H), 1.74 (1.79) (s, 3H), 1.55 – 1.17 (m, 18H); **^{13}C NMR** (101 MHz, CDCl$_3$) δ 197.7 (191.9), 168.5 (166.9), 156.2, 155.8, 143.5, 131.9, 131.0, 127.5, 124.6, 118.2, 84.3, 81.6, (83.3), 74.09 (73.2), 48.6 29.4 (29.12, 28.3, 27.9, (28.1), 16.8; **HRMS** (ESI$^+$) *m/z*: [M+H]$^+$ calculated for $C_{22}H_{32}N_3O_6^+$ 434.2291; found 434.2303.

[α]D[20] = +117.4 (c =1.0, $CHCl_3$).

HPLC: The enantiomeric excess (99%) was determined by HPLC analysis using a Daicel Chiralpak IA column: *n*-hexane: isopropanol = 90:10, flow rate = 1.0 mL/min, λ = 254 nm, R_t (minor) = 23.388 min, R_t (major) = 27.510 min.

Di-*tert*-butyl (*R*)-1-(2-formyl-1-(methyl(phenyl)amino)-1-oxobutan-2-yl)hydrazine-1,2-dicarboxylate (3n)

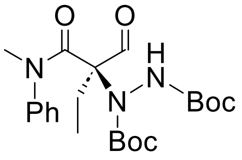

With **2n** (0.100 g, 0.487 mmol), D^{t}BAD (0.135 g, 0.586 mmol) and **cat3** (0.002 g, 0.005 mmol). Chromatography on silica gel with EtOAc/hexane (10–30%) afforded **3n** as a colorless sticky solid (0.171 g, 80%). **R_f** = 0.39 (3:7 EtOAc: hexane); **IR** (film, ν_{max}/cm^{-1}) 1702, 1653; **^{1}H NMR** (300 MHz, $CDCl_3$) δ 9.96 (10.03, 9.94) (s, 1H), 7.53 – 7.37 (m, 3H), 7.23 – 7.14 (m, 2H), 3.90 (6.32, 4.46, 4.08) (s, 1H), 3.29 (3.70, 3.54, 3.27) (s, 3H), 1.96 – 1.78, 1.68 – 1.58 (2 x m, 2H), 1.47, 1.36, (2.25 – 2.02, 1.71, 1.34) (2 x s, 18H), 0.98 (t, J = 7.3 Hz, 3H); **^{13}C NMR** (101 MHz, $CDCl_3$) δ 193.6 (198.2, 194.8, 193.1), 168.6, 156.2, 154.8, (154.3, 153.5), 142.4, 130.0, 128.8, 127.5, (129.8, 129.4, 128.4), 82.9, 82.4, (84.0, 81.5), 72.7 (73.5, 73.4, 73.0), 39.8 (38.5), 28.3, 28.1, 25.1 (26.0), 9.3 (10.3); **HRMS** (ESI$^+$) *m/z*: [M+H]$^+$ calculated for $C_{22}H_{34}N_3O_6^+$ 436.2442; found 436.2448.

[α]D[20] = +111.1 (c =1.0, $CHCl_3$).

HPLC: The enantiomeric excess (97%) was determined by HPLC analysis using a Daicel Chiralcel OD column: *n*-hexane: *i*-Propanol = 98:2, flow rate = 1.0 mL/min, λ = 254 nm, R_t (minor) = 9.824 min, R_t (major) = 11.255 min.

Di-*tert*-butyl (*R*)-1-(3-cyclopentyl-2-formyl-1-(methyl(phenyl)amino)-1-oxopropan-2-yl)hydrazine-1,2-dicarboxylate (3o)

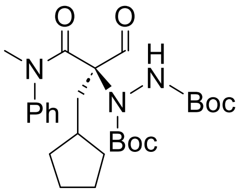

With **2o** (0.150 g, 0.578 mmol), D^{t}BAD (0.161 g, 0.699 mmol), and **cat3** (0.003 g, 0.007 mmol) in ethyl acetate (1.5 mL). Chromatography on silica gel with EtOAc/hexane (10–20%) afforded **3o** as a beige glass (0.217 g, 76%). **R_f** = 0.23 (3:7 EtOAc: hexane); **IR** (film, ν_{max}/cm^{-1}) 1729, 1706; **^{1}H NMR** (300 MHz, $CDCl_3$) δ 10.20 (10.46, 10.30) (s, 1H), 7.54 – 7.28 (m, 3H), 7.22 – 7.10 (m, 2H), 3.69 (6.36, 6.22, 3.62) (s, 1H), 3.25 (3.30, 3.28) (s, 3H), 2.14 – 1.21 (m, 27H), 1.03 – 0.80 (m, 2H); **^{13}C NMR** (101 MHz, $CDCl_3$) δ 195.8 (200.6, 199.7, 197.2), 173.6 (170.7, 169.1, 169.0), 156.2, 155.9, (155.1, 154.7, 154.3), 144.5 (142.8), 129.8, 127.8, 127.4, (130.1, 129.4,

128.7, 127.6, 127.0, 126.5), 82.8, 81.6, (83.8, 83.4, 82.3), 72.0 (74.9, 72.6), 39.8 (40.2), 37.4, 36.1, 33.5, 32.5, 32.0, (38.9, 38.7, 38.2,35.9, 34.7, 34.3, 34.1, 29.8, 29.5), 28.3, 28.1, 25.2 (25.4, 24.9); **HRMS** (ESI$^+$) *m/z*: [M+H]$^+$ calculated for $C_{29}H_{40}N_3O_8^+$ 490.2912; found 490.2916.

HPLC: chiral analysis was done of a functionalized oxazolidinone to give a 98% *ee* (see **4o**).

Di-*tert*-butyl (*R*)-1-(2-formyl-1-(methyl(phenyl)amino)-1-oxopent-4-en-2-yl)hydrazine-1,2-dicarboxylate (3p)

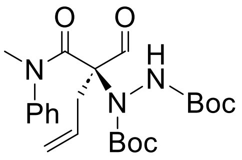

With **2p** (0.100 g, 0.460 mmol), D^{t}BAD (0.127 g, 0.552 mmol) and **cat3** (0.002 g, 0.005 mmol) for 5d. Chromatography on silica gel with EtOAc/hexane (10–30%) afforded **3p** as a colorless sticky solid (0.071 g, 34%). **R$_f$** = 0.33 (3:7 EtOAc: hexane); **IR** (film, ν_{max}/cm^{-1}); 1726, 1705; **^{1}H NMR** (400 MHz, $CDCl_3$) δ 9.91 (9.99, 9.94, 9.83) (s, 1H), 7.49 – 7.30 (m, 3H), 7.24 – 7.15 (m, 2H), 6.36 (6.30, 4.72, 4.01) (s, 1H), 6.06 – 5.67 (m, 1H), 5.34 – 4.87 (m, 2H), 3.27 (3.69, 3.30, 3.29, 3.24) (s, 3H), 2.96 – 2.12 (m, 2H), 1.86 – 1.36 (2 x s, 18H); **^{13}C NMR** (101 MHz, $CDCl_3$) δ 193.1 (197.4, 194.3, 193.3, 192.3), 172.4 (174.9, 168.2), 154.7, 154.2, (156.2, 155.9, 155.7), 144.2 (142.5), 137.6, 130.0, 129.9, 127.4, (131.9, 129.4, 128.9, 127.7), 118.8 (115.1), 83.0, 81.5, (82.5, 82.1), 72.3, 39.9 (38.6, 37.4, 36.7, 33.5, 28.2, 28.0, (29.5, 28.2); **HRMS** (ESI$^+$) *m/z*: [M+Na]$^+$ calculated for $C_{23}H_{33}N_3NaO_6^+$ 470.2267; found 470.2262.

$[\alpha]_D^{21}$ = +119.1 (*c* = 1.2, $CHCl_3$).

HPLC: The enantiomeric excess (98%) was determined by HPLC analysis using a Daicel Chiralpak IA column: *n*-hexane: isopropanol = 90:10, flow rate = 1.0 mL/min, λ = 254 nm, R_t (minor) = 13.022 min, R_t (major) = 20.034 min.

Di-*tert*-butyl (*R*)-1-(2-formyl-1-(methyl(phenyl)amino)-1-oxohexan-2-yl)hydrazine-1,2-dicarboxylate (3q)

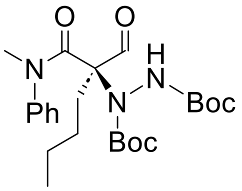

With **2q** (0.100 g, 0.429 mmol), D^{t}BAD (0.118 g, 0.513 mmol) and **cat3** (0.002 g, 0.005 mmol). Chromatography on silica gel with EtOAc/hexane (10–30%) afforded **3q** as a colorless sticky solid (0.162 g, 81%). **R$_f$** = 0.34 (3:7 EtOAc: hexane); **IR** (film, ν_{max}/cm^{-1}) 1727, 1703; **^{1}H NMR** (400 MHz, $CDCl_3$) δ 9.95 (10.01, 9.99, 9.91) (s, 1H), 7.53 – 7.30 (m, 3H), 7.24 – 7.04 (m, 2H), 6.33 (6.04, 4.48, 3.88) (s, 1H), 3.25 (3.67, 3.22) (s, 3H), 1.76 – 1.20 (m, 24H), 0.89 (t, *J* = 7.3 Hz, 3H); **^{13}C NMR** (101 MHz, $CDCl_3$) δ 193.8 (198.3, 194.7, 193.1), 168.7 (173.3), 154.7, 153.7,

(156.0, 152.8), 142.3 (144.4), 129.88, 128.7, 127.5, (129.3, 128.3, 126.5), 82.8, 82.3, (83.8, 81.4), 72.1 (72.7, 72.4), 39.7 (38.3, 37.2), 31.6 (34.0, 32.4), 28.2, 28.1 (28.0), 26.6 (26.4), 23.2, 13.9 (13.8); **HRMS** (ESI^+) *m/z*: $[M+Na]^+$ calculated for $C_{24}H_{37}N_3NaO_6^+$ 486.2580; found 486.2575.

$[\alpha]_D^{21}$ = +78.0 (*c* =1.0, $CHCl_3$).

HPLC: The enantiomeric excess (99%) was determined by HPLC analysis using a Daicel Chiralpak IA column: *n*-hexane: isopropanol = 90:10, flow rate = 1.0 mL/min, λ = 254 nm, R_t (minor) = 13.369 min, R_t (major) = 17.054 min.

Di-*tert*-butyl (*R*)-1-(4-(benzyloxy)-2-formyl-1-(methyl(phenyl)amino)-1-oxobutan-2-yl)hydrazine-1,2-dicarboxylate (3r)

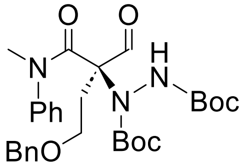

With **2r** (0.100 g, 0.321 mmol), D^{t}BAD (0.088 g, 0.382 mmol) and **cat3** (0.001 mg, 0.002 mmol). Chromatography on silica gel with EtOAc/hexane (10–30%) afforded **3r** as a colorless sticky solid (132 mg, 76%). **R_f** = 0.36 (3:7 EtOAc: hexane); **IR** (film, ν_{max}/cm^{-1}) 1727, 1716; **^{1}H NMR** (400 MHz, $CDCl_3$) δ 9.97 (10.02, 10.06, 9.93) (s, 1H), 7.57 – 7.06 (m, 10H), 4.45 (4.52, 4.39) (s, 2H), 3.87 – 3.58 (m, 2H), 3.13 (3.49, 3.24) (s, 3H), 2.47 – 1.88 (m, 2H), 1.46, 1.35 (1.52, 1.43, 1.32) (2 x s, 18H); **^{13}C NMR** (101 MHz, $CDCl_3$) δ 193.1 (194.2), 168.2 (172.7, 169.5), 154.5, 154.1, (156.0, 155.5, 152.4), 142.4, 138.3 (138.5), 129.7, 129.6, 128.5, 128.3, 127.9, 127.6, (130.0, 127.5), 82.8, 81.4 (83.9, 82.3), 73.3 (73.4), 70.7, 65.8 (66.1, 65.7), 39.5 (38.7), 32.1 (33.4), 28.1, 27.9, (28.2, 28.0); **HRMS** (ESI^+) *m/z*: $[M+Na]^+$ calculated for $C_{29}H_{39}N_3NaO_7^+$ 564.2686; found 564.2680.

$[\alpha]_D^{21}$ = +89.8 (*c* =1.0, $CHCl_3$).

HPLC: The enantiomeric excess (98%) was determined by HPLC analysis using a Daicel Chiralpak IA column: *n*-hexane: isopropanol = 90:10, flow rate 1.0 mL/min, λ = 254 nm, R_t (minor) = 14.084 min, R_t (major) = 17.009 min.

Di-*tert*-butyl (*R*)-1-(2-benzyl-1-(methyl(phenyl)amino)-1,3-dioxopropan-2-yl)hydrazine-1,2-dicarboxylate (3s)

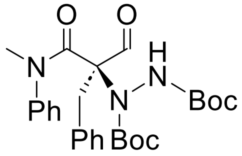

With **2s** (0.100 g, 0.374 mmol), D^{t}BAD (0.103 g, 0.447 mmol) and **cat3** (0.002 g, 0.005 mmol). Chromatography on silica gel with EtOAc/hexane (5–10%) afforded **3s** as a white solid (0.164 g, 89%). **R_f** = 0.36 (3:7 EtOAc: hexane); **IR** (film, ν_{max}/cm^{-1}) 1727, 1706; **^{1}H NMR** (300 MHz,

$CDCl_3$) δ 10.06 (9.76, 9.58) (s, 1H), 7.58 – 6.62 (m, 10H), 6.49 (5.28, 4.35) (s, 1H), 3.82 – 2.90 (m, 5H), 1.46 (1.52) (2 x s, 18H); **^{13}C NMR** (101 MHz, $CDCl_3$) δ 198.8 (196.9, 193.6, 191.7), 167.7 (168.0), 156.1, 154.9, (154.3 152.3), 142.2 (144.4, 142.7, 142.5), 134.6 (134.9, 133.8, 133.4), 131.5, 130.8, 128.6, 128.2, 127.8, 127.4, (129.8, 129.5, 129.2, 126.8, 126.3), 82.8, 81.7, (84.0, 83.4, 82.4, 81.5), 75.0 (74.3), 40.3 (39.3), 38.5 (37.3), 28.2, 28.0; **HRMS** (ESI^+) *m/z*: $[M+H]^+$ calculated for $C_{27}H_{36}N_3O_6^+$ 498.2599; found 498.2602.

The ^{1}H VT NMR at 353K is provided in the appendix.

$[\alpha]_D^{20}$ = +124.8 (*c* =1.0, $CHCl_3$).

HPLC: The enantiomeric excess (99%) was determined by HPLC analysis using a Daicel Chiralpak AD column: *n*-hexane: isopropanol = 98:2, flow rate = 0.5 mL/min, λ = 254 nm, R_t (major) = 84.029 min, R_t (minor) = 93.620 min.

Di-*tert*-butyl (*R*)-1-(3-formyl-1-(4-methoxybenzyl)-2-oxopiperidin-3-yl)hydrazine-1,2-dicarboxylate (3t)

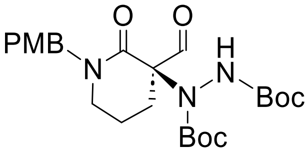

With **2t** (0.100 g, 0.404 mmol), D^{t}BAD (0.112 g, 0.486 mmol) and **cat3** (0.002 g, 0.005 mmol). Chromatography on silica gel with EtOAc/hexane (10–30%) afforded **3t** as a colorless sticky solid (0.036 g, 19%). **R_f** = 0.11 (1:4 EtOAc: hexane); **IR** (film, ν_{max}/cm^{-1}) 1687, 1636; **^{1}H NMR** (400 MHz, $CDCl_3$) δ 9.51 (s, 1H), 7.25 – 7.10 (brs, 2H), 6.96 (6.46) (s, 1H), 6.82 (d, *J* = 8.6 Hz, 2H), 4.73 – 4.28 (m, 2H), 3.76 (s, 3H), 3.30 – 3.12 (m, 2H), 2.59 – 2.23 (m, 2H), 1.87 – 1.77 (m, 2H), 1.44 (1.59) (2 x s, 18H); **^{13}C NMR** (101 MHz, $CDCl_3$) δ 192.7, 163.8, 158.8, 154.9, 154.8, (155.7, 155.5, 155.3, 154.6), 128.9, 128.0, 113.8, 82.8, 81.1, 74.2, 55.0, 50.0, 46.6, 27.9, 27.8, 25.9 (26.7), 19.1; **HRMS** (ESI^+) *m/z*: $[M+Na]^+$ calculated for $C_{24}H_{35}N_3NaO_7^+$ 500.2373; found 500.2367.

$[\alpha]_D^{21}$ = -6.7 (*c* =1.0, $CHCl_3$).

HPLC: The enantiomeric excess (90%) was determined by HPLC analysis using a Daicel Chiralpak IA column: *n*-hexane: isopropanol = 95:5, flow rate = 1.0 mL/min, λ = 254 nm, R_t (major) = 16.833 min, R_t (minor) = 30.438 min.

Di-*tert*-butyl (*R*)-1-(1-((3-bromophenyl)(methyl)amino)-2-formyl-3-(4-nitrophenyl)-1-oxopropan-2-yl)hydrazine-1,2-dicarboxylate (3u)

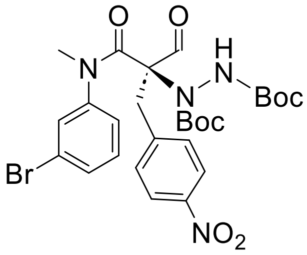

With **2u** (0.100 g, 0.256 mmol), D^{t}BAD (0.071 g, 0.308 mmol) and **cat3** (0.001 g, 0.002 mmol). Chromatography on silica gel with EtOAc/hexane (10–20%) afforded **3u** as a yellow solid (0.118 g, 71%). **R$_f$** = 0.27 (3:7 EtOAc: hexane); **IR** (film, ν_{max}/cm^{-1}) 1648, 1636; **^{1}H NMR** (300 MHz, $CDCl_3$) δ 9.97 (9.46, 9.30) (s, 1H), 8.19 (d, *J* = 8.3 Hz, 2H), 7.65 – 6.73 (m, 6H), 6.43 (5.89, 5.00) (s, 1H), 3.84 – 2.89 (m, 5H), 1.48, 1.45, (1.67) (2 x s, 18H); **^{13}C NMR** (101 MHz, $CDCl_3$) δ 195.9 (193.1, 191.2, 189.4), 167.7 – 166.9, 156.7 – 154.3, 147.3, 143.2 – 142.1, 132.6, 131.8, 130.8, 130.5, 129.5, 126.9, 123.7, 123.4, 83.7, 82.3, (84.2, 83.1), 74.1 (75.6), 40.5 – 39.5, 38.9 – 38.0, 28.6 – 27.7; **HRMS** (ESI$^+$) *m/z*: [M+H]$^+$ calculated for $C_{27}H_{34}BrN_4O_8^+$ 621.1555; found 621.1560.

[α]$_D^{20}$ = +19.3 (*c* =1.0, $CHCl_3$).

HPLC: The enantiomeric excess (99%) was determined by HPLC analysis using a Daicel Chiralpak IA column: *n*-hexane: isopropanol = 90:10, flow rate = 1.0 mL/min, λ = 254 nm, R_t (minor) = 12.478 min, R_t (major) = 18.090 min.

....

6. N-N Bond cleavage to quaternized oxazolidinones

Step 1: Reductive cyclization to Boc-protected oxazolidinone hydrazides 4a, 4c, 4j, 4n and 4o

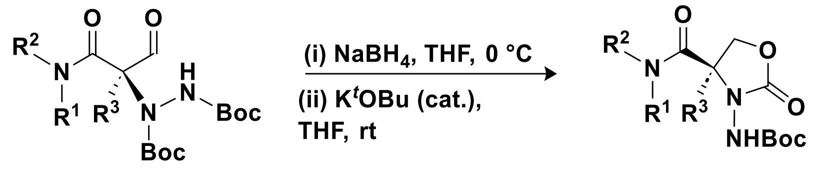

3a, 3c, 3j, 3n and 3o

4a: R^1 = Ph; R^2 = R^3 = Me
4c: R^1 = *p*-ClPh; R^2 = R^3 = Me
4j: R^1 = R^2 = PMB; R^3 = Me
4n: R^1 = Ph; R^2 = Me; R^3 = Et
4o: R^1 = Ph; R^2 = Me; R^3 = CH_2-cyclopentyl

To a solution of **3** (1.0 eq) in THF (3 mL/mmol) was added $NaBH_4$ (1.2 eq) and the solution stirred at room temperature for 20 min. The reaction mixture was then quenched with sat. NH_4Cl and the organic layer separated. The aqueous phase was extracted with ethyl acetate (x 3) and the combined organic extracts were dried over $MgSO_4$ and filtered. The solvent was removed under reduced pressure and the alcohol product was used without further purification. The crude alcohol was dissolved in THF (3 mL per mmol), K^{t}OBu (10 mol%) was added and the solution was stirred at room temperature overnight. The reaction mixture was then quenched with water and the organic layer separated. The aqueous phase was extracted with ethyl acetate (x 3) and the combined organic extracts were dried over $MgSO_4$ and filtered. The solvent was removed under reduced pressure and the crude product was purified by flash column chromatography using ethyl acetate/hexane mixtures as eluent. Yields are based on the two steps. The NMR spectra for compounds **4n** and **4o** revealed that they exist as rotamers due to the hydrazide moiety. For the NMR data, minor rotamers are reported in brackets.

Characterization data for 4a, 4c, 4j, 4n and 4o.

***tert*-Butyl (*R*)-(4-methyl-4-(methyl(phenyl)carbamoyl)-2-oxooxazolidin-3-yl)carbamate (4a)**

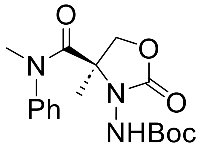

With **3a** (0.410 g, 0.973 mmol), $NaBH_4$ (0.045 g, 1.190 mmol), and K^{t}OBu (0.010 g, 0.089 mmol). Chromatography on silica gel with EtOAc/hexane (30–50%) afforded **4a** as a white solid (0.335 g, 95%). **R$_f$** = 0.15 (3:7 EtOAc: hexane); **IR** (film, ν_{max}/cm^{-1}) 1783, 1733, 1643; **^{1}H NMR** (300 MHz, $CDCl_3$) δ 7.53 – 7.39 (m, 3H), 7.21 (d, *J* = 7.3 Hz, 2H), 6.51 (5.88) (s, 1H), 4.58 (d, *J* = 9.1 Hz, 1H), 3.84 (d, *J* = 9.1 Hz, 1H), 3.27 (s, 3H), 1.43 (s, 9H), 1.32 (s, 3H); **^{13}C NMR** (151 MHz, $CDCl_3$) δ 171.2, 155.5, 154.7, 142.6, 130.4, 129.2, 128.1, 81.9, 71.9, 67.1, 41.0, 28.2, 21.7; **HRMS** (ESI$^+$) *m/z*: [M+H]$^+$ calculated for $C_{17}H_{24}N_3O_5^+$ 350.1710; found 350.1716.

[α]$_D^{20}$ = +15.7 (*c* =1.0, $CHCl_3$).

HPLC: The enantiomeric excess (98%) was determined by HPLC analysis using a Daicel Chiralpak AD column: *n*-hexane: isopropanol = 70:30, flow rate = 1.0 mL/min, λ = 254 nm, R_t (minor) = 13.863 min, R_t (major) = 35.921 min.

***tert*-Butyl (*R*)-(4-((4-chlorophenyl)(methyl)carbamoyl)-4-methyl-2-oxooxazolidin-3-yl)carbamate (4c)**

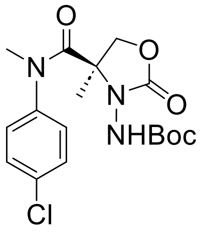

With **3c** (0.100 g, 0.219 mmol), $NaBH_4$ (0.010 g, 0.264 mmol), and K^{t}OBu (0.002 g, 0.018 mmol). Chromatography on silica gel with EtOAc/hexane (10–30%) afforded **4c** as a white solid (0.072 g, 86%). **R$_f$** = 0.16 (3:7 EtOAc: hexane); **IR** (film, ν_{max}/cm^{-1}) 1783, 1733, 1645; **^{1}H NMR** (300 MHz, $CDCl_3$) δ 7.44 (d, *J* = 8.2 Hz, 2H), 7.17 (d, *J* = 8.2 Hz, 2H), 6.69 (6.38) (s, 1H), 4.53 (d, *J* = 9.1 Hz, 1H), 3.87 (s, 1H), 3.24 (s, 3H), 1.43 (s, 9H), 1.34 (s, 3H); **^{13}C NMR** (101 MHz, $CDCl_3$) δ 171.3, 155.8, 155.0, 141.3, 135.3, 130.8, 129.9, 82.2, 71.6, 67.3, 41.1, 28.5, 22.0; **HRMS** (ESI$^+$) *m/z*: [M+H]$^+$ calculated for $C_{17}H_{22}ClN_3O_5^+$ 384.1321; found 384.1326.

[α]$_D^{20}$ = +16.0 (*c* =1.0, $CHCl_3$).

HPLC: The enantiomeric excess (98%) was determined by HPLC analysis using a Daicel Chiralpak AD column: *n*-hexane: isopropanol = 70:30, flow rate = 1.0 mL/min, λ = 254 nm, R_t (minor) = 12.701 min, R_t (major) = 24.511 min.

***tert*-Butyl (*R*)-(4-(bis(4-methoxybenzyl)carbamoyl)-4-methyl-2-oxooxazolidin-3-yl)carbamate (4j)**

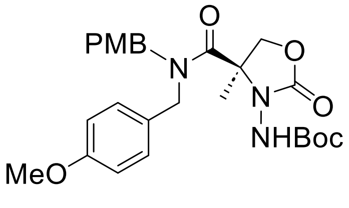

With **3j** (0.100 g, 0.175 mmol), $NaBH_4$ (0.008 g, 0.211 mmol), and K^{t}OBu (0.002 g, 0.018 mmol). Chromatography on silica gel with EtOAc/hexane (10-30%) afforded **4j** as a white solid (0.084 g, 93%). **R$_f$** = 0.24 (1:1 EtOAc: hexane); **IR** (film, ν_{max}/cm^{-1}) 1732, 1701; **^{1}H NMR** (400 MHz, $CDCl_3$) δ 7.26 (s, 1H), 7.09 – 7.01 (m, 4H), 6.97 – 6.79 (m, 4H), 4.62 – 4.57 (m, 1H), 4.48 (d, *J* = 9.1 Hz, 1H), 4.35 – 4.14 (m, 4H), 3.81 - 3.78 (s, 6H), 1.72 (s, 3H), 1.49 (s, 9H); **^{13}C NMR** (101 MHz, $CDCl_3$) δ 172.5, 160.0, 159.7, 155.5, 155.2, 130.1, 128.5, 128.2, 126.4, 115.2, 114.7, (114.5, 114.4), 82.2, 70.9, 66.4, 55.8, 55.8, 49.2, 47.7, 28.6, 21.5; **HRMS** (ESI$^+$) *m/z*: [M+Na]$^+$ calculated for $C_{26}H_{33}N_3NaO_7^+$ 522.2216; found 522.2211.

$[\alpha]_D^{21}$ = +33.4 (c =1.0, $CHCl_3$).

HPLC: The enantiomeric excess (96%) was determined by HPLC analysis using a Daicel Chiralpak IC column: *n*-hexane: isopropanol = 40:60, flow rate = 1.0 mL/min, λ = 254 nm, R_t (minor) = 31.858 min, R_t (major) = 40.842 min.

***tert*-Butyl (*R*)-(4-ethyl-4-(methyl(phenyl)carbamoyl)-2-oxooxazolidin-3-yl)carbamate (4n)**

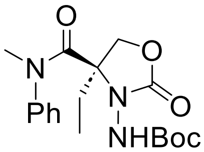

With **3n** (0.100 g, 0.230 mmol), $NaBH_4$ (0.010 g, 0.264 mmol), and K^tOBu (0.003 g, 0.027 mmol). Chromatography on silica gel with EtOAc/hexane (10–30%) afforded **4n** as a white solid (0.068 g, 81%). R_f = 0.15 (3:7 EtOAc: hexane); **IR** (film, ν_{max}/cm^{-1}) 1783, 1736, 1642; **^{1}H NMR** (300 MHz, $CDCl_3$) δ 7.54 – 7.34 (m, 3H), 7.20 (d, J = 6.8 Hz, 2H), 6.71 (6.18) (s, 1H), 4.52 (d, J = 9.2 Hz, 1H), 3.88 – 3.66 (m, 1H), 3.25 (3.19) (s, 3H), 2.11 – 1.56 (m, 2H), 1.40 (1.32) (s, 9H), 0.97 (t, J = 7.4 Hz, 3H); **^{13}C NMR** (151 MHz, $CDCl_3$) δ 170.8, 155.6, 154.5, 142.6, 130.2, 129.0, 127.8, 81.7 (82.3), 70.1, 70.0, 40.8, 28.2, 28.1, 8.5; **HRMS** (ESI$^+$) *m/z*: $[M+H]^+$ calculated for $C_{18}H_{26}N_3O_5^+$ 364.1867; found 364.1872.

$[\alpha]_D^{20}$ = +15.1 (c =1.0, $CHCl_3$).

HPLC: The enantiomeric excess (99%) was determined by HPLC analysis using a Daicel Chiralpak AD column: *n*-hexane: isopropanol = 60:40, flow rate = 1.0 mL/min, λ = 254 nm, R_t (minor) = 12.118 min, R_t (major) = 20.275 min.

***tert*-Butyl (*R*)-(4-(cyclopentylmethyl)-4-(methyl(phenyl)carbamoyl)-2-oxooxazolidin-3-yl)carbamate (4o)**

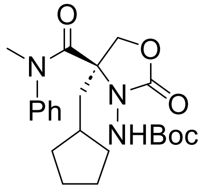

With **3o** (0.340 g, 0.694 mmol), $NaBH_4$ (0.032 g, 0.846 mmol) and K^tOBu (0.008 g, 0.071 mmol). **Note**: For this particular substrate, the reaction mixture was heated to 50 °C overnight for the second step (cyclization). Chromatography on silica gel with EtOAc/hexane (10–30%) afforded **4o** as a white solid (0.280 g, 96%). R_f = 0.15 (3:7 EtOAc: hexane); **IR** (film, ν_{max}/cm^{-1}) 1782, 1708, 1645; **^{1}H NMR** (300 MHz, $CDCl_3$) δ 7.57 – 7.36 (m, 3H), 7.23 (d, J = 7.5 Hz, 2H), 6.07 (4.85) (s, 1H), 4.66 (d, J = 9.0 Hz, 1H), 4.02 (s, 1H), 3.26 (s, 3H), 2.14 – 1.47 (m, 8H), 1.40 (s, 9H), 1.10 – 0.58 (m, 3H); **^{13}C NMR** (101 MHz, $CDCl_3$) δ 171.2, 155.9, 154.3, 142.7, 130.3, 128.9, 128.2, 81.6 (82.5), 71.1 (72.5), 70.0, 41.1, 39.5, 35.1, 34.0, 34.0, 28.1, 25.1, 25.0; **HRMS** (ESI$^+$) *m/z*: $[M+H]^+$ calculated for $C_{22}H_{32}N_3O_5^+$ 418.2336; found 418.2354.

$[\alpha]_D^{20}$ = +105.7 (*c* =1.0, $CHCl_3$).

HPLC: The enantiomeric excess (98%) was determined by HPLC analysis using a Daicel Chiralpak IA column: *n*-hexane: isopropanol = 90:10, flow rate = 1.0 mL/min, λ = 254 nm, R_t (minor) = 25.262 min, R_t (major) = 41.463 min.

....

Step 2: N-N bond cleavage to oxazolidinones 5a and 5j *via* reductive deamination.

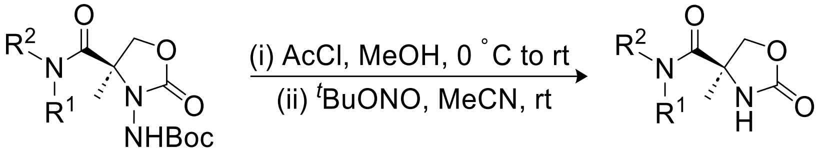

4a: R^1 = Ph; R^2 = Me
4j: R^1 = R^2 = PMB

5a: R^1 = Ph; R^2 = Me
5j: R^1 = R^2 = PMB

To a solution of **4** in methanol at 0 °C under argon was added acetyl chloride and the mixture stirred at this temperature for 15 min (stoichiometries are given below). On completion of Boc group removal (by TLC and the crude ^{1}H NMR spectrum), the reaction was quenched with NaOH (2.5 M) followed by evaporation of volatiles. The resulting mixture was extracted with ethyl acetate (x 3). The combined organic layers were dried over $MgSO_4$ and filtered. The solvent was removed under reduced pressure and the crude hydrazine product was used in the next step without further purification. Extending from the nitrous deamination protocol for hydrazine N-N cleavage, which necessitates using harsh conditions,[10] the crude hydrazine was dissolved in MeCN and *tert*-butyl nitrite (2.0 eq) was added in one portion at room temperature. Once TLC had indicated a satisfactory conversion (~ 15 min), the reaction mixture was quenched with water, ethyl acetate added, and the organic layer separated. The aqueous phase was extracted with ethyl acetate (x 2), and the combined organic extracts were dried over $MgSO_4$ and filtered. The solvent was removed under reduced pressure and the crude product was purified by flash column chromatography using ethyl acetate/hexane mixtures as eluent.

Characterization data for 5a and 5j

(*R*)-*N*,4-Dimethyl-2-oxo-*N*-phenyloxazolidine-4-carboxamide (5a)

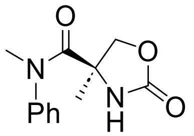

With **4a** (0.200 g, 0.572 mmol), methanol (6 mL) and acetyl chloride (2 mL) followed by the resultant crude hydrazine (0.100 g, 0.401 mmol) in MeCN (2 mL) and *tert*-butyl nitrite (0.14 mL, 1.20 mmol). Chromatography on silica gel with EtOAc/hexane (30–60%) afforded **5a** as a white solid (0.059 g, 63%). **R_f** = 0.36 (9:1 EtOAc: hexane); **IR** (film, ν_{max}/cm^{-1}) 1749, 1636; **^{1}H NMR**

(400 MHz, $CDCl_3$) δ 7.49 – 7.40 (m, 3H), 7.25 – 7.23 (m, 2H), 4.77 (s, 1H), 4.54 (d, *J* = 8.9 Hz, 1H), 3.68 (d, *J* = 8.9 Hz, 1H), 3.24 (s, 3H), 1.53 (s, 3H); **^{13}C NMR** (101 MHz, $CDCl_3$) δ 171.1, 157.0, 142.0, 130.3, 129.3, 128.0, 74.0, 62.8, 40.1, 27.4; **HRMS** (ESI^+) *m/z*: $[M+Na]^+$ calculated for $C_{12}H_{14}N_2NaO_3^+$ 257.0902; found 257.0897.

$[\alpha]_D^{21}$ = +43.3 (*c* =1.4 $CHCl_3$).

HPLC: The enantiomeric excess (98%) was determined by HPLC analysis using a Daicel Chiralpak IC column: *n*-hexane: isopropanol = 60:40, flow rate = 1.0 mL/min, λ = 254 nm, R_t (minor) =38.980 min, R_t (major) = 44.536 min.

(*R*)-*N,N*-Bis(4-methoxybenzyl)-4-methyl-2-oxooxazolidine-4-carboxamide (5j)

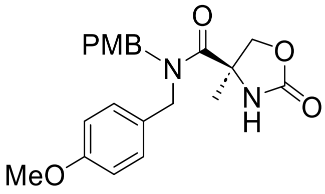

With **4j** (0.150 g, 0.300 mmol), methanol (6 mL) and acetyl chloride (2 mL) followed by the resultant crude hydrazine (0.100 g, 0.250 mmol) in MeCN (2 mL) and *tert*-butyl nitrite (0.06 mL, 0.50 mmol). Chromatography on silica gel with EtOAc/hexane (10–40%) afforded **5j** as a white solid (0.052 g, 54%). **R_f** = 0.31 (7:3 EtOAc: hexane); **IR** (film, ν_{max}/cm^{-1}) 1756, 1632; **^{1}H NMR** (400 MHz, $CDCl_3$) δ 7.13 – 7.00 (m, 4H), 6.93 – 6.82 (m, 4H), 6.14 (s, 1H), 4.65 – 4.13 (m, 6H), 3.81 (brs, 6H), 1.64 (s, 3H); **^{13}C NMR** (101 MHz, $CDCl_3$) δ 171.8, 159.6, 159.6, 157.3, 129.7, 129.7, 127.9, 127.9, 114.8, 114.3, 81.1, 72.9, 62.1, 62.1, 55.5, 55.2, 26.9; **HRMS** (ESI^+) *m/z*: $[M+Na]^+$ calculated for $C_{21}H_{24}N_2NaO_5^+$ 407.1583; found 407.1577.

$[\alpha]_D^{21}$ = +20.1 (*c* =1.0 $CHCl_3$).

HPLC: The enantiomeric excess (96%) was determined by HPLC analysis using a Daicel Chiralpak IC column: *n*-hexane: isopropanol = 40:60, flow rate = 1.0 mL/min, λ = 254 nm, R_t (minor) = 48.660 min, R_t (major) = 53.585 min.

....

Step 3: Deprotection of the PMB groups of 5j to afford

(*R*)-4-Methyl-2-oxooxazolidine-4-carboxamide (6j)[11]

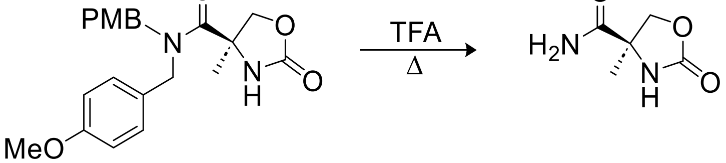

5j 6j

A solution of **5j** (0.050 g) was refluxed in TFA (2 mL) in an oil bath for 17 h. After cooling to room temperature, water (5 mL) was added and the solution extracted once with ethyl acetate (5 mL). Freeze-drying the aqueous layer afforded **6j** as a colorless solid (0.011 g, 61%). **R_f** = 0.50 (9:1 EtOAc: hexane); **^{1}H NMR** (400 MHz, $CDCl_3$) δ 6.66 (s, 1H), 6.28 (s, 1H), 5.64 (s, 1H), 4.59 (d, J = 9.0 Hz, 1H), 4.20 (d, J = 9.0 Hz, 1H), 1.62 (s, 3H); **^{13}C NMR** (101 MHz, $CDCl_3$) δ 174.9, 159.0, 75.0, 61.2, 24.2. **HRMS** (ESI^+) *m/z*: $[M+Na]^+$ calculated for $C_5H_8N_2NaO_3^+$ 167.0433; found 167.0427. All recorded data were in accordance with those previously reported.[11]

$[\alpha]_D^{21}$ = +27.8 (c = 1.0 MeOH).

The *ee* could not be measured by chiral HPLC because compound **6j** is not UV-active.

....

7. Enaminone transition state structures from computer modeling.

Initial guess structures for the transition states (TSs) were generated by scanning along the reaction coordinate in which D^{t}BAD in *cis* and *trans* configurations were placed at the *Re*- and *Si*-faces of the enaminone model compound **m**, shown below in **Figure S1**.

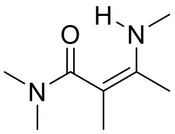

m

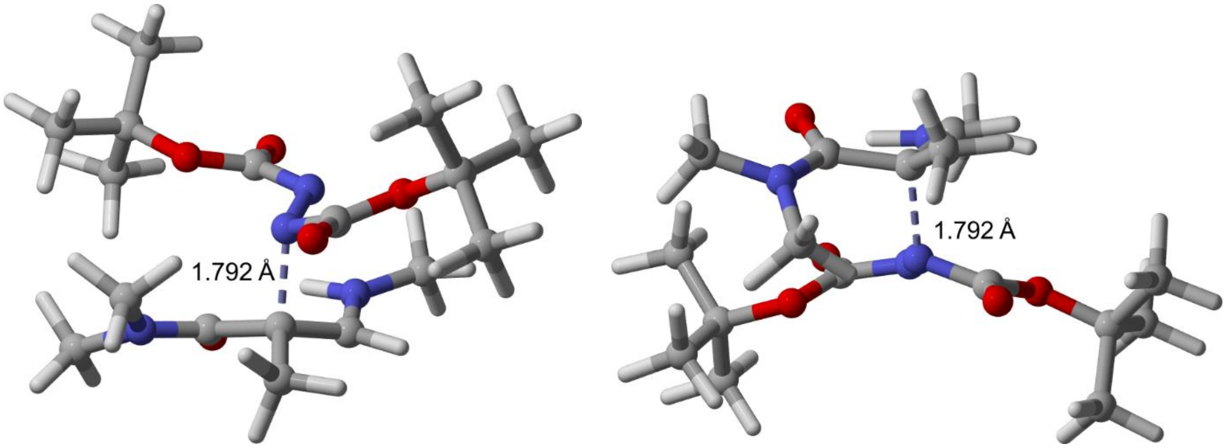

***trans*-D^{t}BAD + *Re*-face attack** ***trans*-D^{t}BAD + *Si*-face attack**

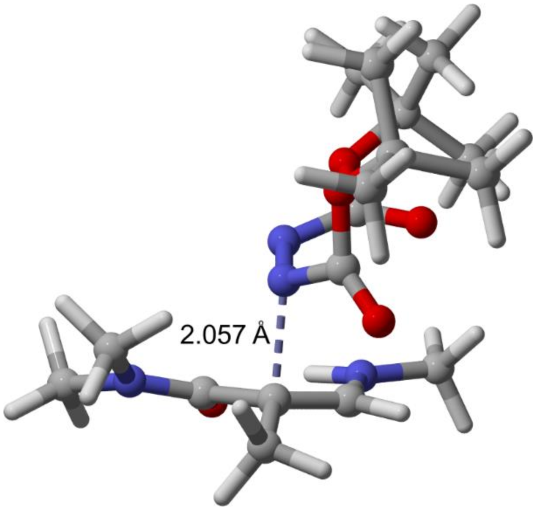

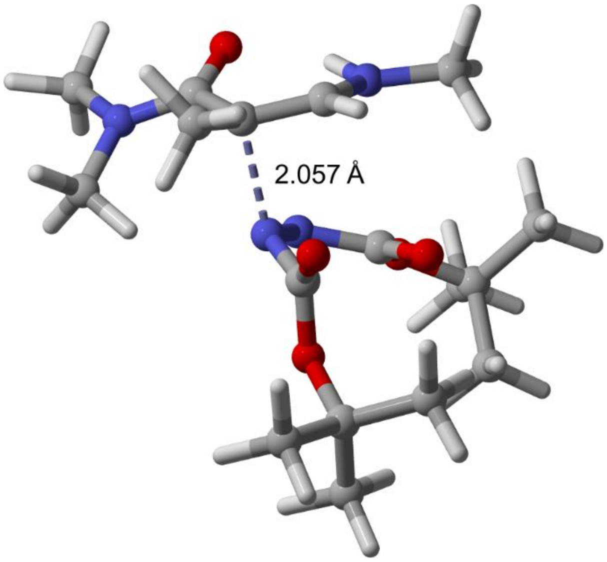

***cis*-D*t*BAD + *Re*-face attack** ***cis*-D*t*BAD + *Si*-face attack**

Figure S1: *cis*- and *trans*-D*t*BAD with *Re* and *Si* faces of enaminone.

The corresponding full enaminone TS structures with a total charge of +2 were then built from these model structures and the conformational space was explored using the Conformer-Rotamer Ensemble Sampling Tool (CREST 2.12),[12,13] in which the C···N forming bond distance was constrained to its value in the model structure, the quinuclidinium N-H bond length was constrained to avoid transfer of the proton to D*t*BAD, and the D*t*BAD was constrained to either a *cis*- or *trans*-configuration on the *Re* or *Si* face. The default metadynamics-based iMTD-GC workflow was used with an energy window of 30 kcal/mol. The GFN2-xTB semi-empirical Hamiltonian[14] was used, as implemented in the xtb 6.6.0 code.[15] The ethyl acetate solvent was included implicitly using the analytical linearized Poisson-Boltzmann (ALPB) model.[16] Using the resulting lowest energy structure of each as an initial guess, the final TS structures were then computed using density functional theory (DFT) at the ωB97X-D/def2-SVP[17,18] level of theory. The conductor-like polarizable continuum (CPCM) model was used to describe the ethyl acetate solvent.[19,20] For calculation of activation energies, the structures of reactants (the enaminone, *cis*-D*t*BAD and *trans*-D*t*BAD) were optimized using a similar procedure starting with generation of a CREST conformer ensemble.

The nature of each stationary point as a TS was confirmed by computing harmonic vibrational frequencies at the ωB97X-D/def2-SVP level of theory and assuring that the normal mode corresponding to the single imaginary frequency corresponds to the reaction coordinate. Note that the reaction coordinate in each case is a combination of the C···N forming bond and H atom transfer from the quinuclidinum to D*t*BAD (see Figure S2). This was confirmed by calculating the intrinsic reaction coordinate (IRC) path. The computed frequencies were used to also calculate zero-point energies as well as thermal and entropic corrections to the enthalpy and Gibbs free energy, at 298.15 K. Vibrational entropy was calculated using the quasi-RRHO (rigid rotor harmonic oscillator) model of Grimme in which vibrational frequencies below 100 cm^{-1} are treated as free rotors and for those above, the RRHO expression is retained; a damping function is used to

interpolate between these two expressions.[21] All DFT calculations were done using Gaussian 16 C.01.[22]

Figure S2 shows the TS structures for attack of *trans*-D^{t}BAD and *cis*-D^{t}BAD on the *Re*-face, leading to the *R*-hydrazide (**TS1** and **TS2**, respectively), and attack of *cis*-D^{t}BAD on the *Si*-face, leading to the *S*-hydrazide (**TS3**). Structure **TS1 (*trans/Re*)** is **48.1 kJ mol^{-1}** lower in enthalpy (or **42.6 kJ mol^{-1}** lower in Gibbs free energy) than structure **TS2 (*cis/Re*)**, confirming that the *trans*-D^{t}BAD configuration is preferred. Unfortunately, the geometry optimization of the TS structure for attack of *trans*-D^{t}BAD on the *Si*-face did not converge despite multiple attempts at refining the initial structure and redoing the optimization at different levels of theory, so we are not able to directly compare the corresponding *Re* and *Si*-face TS structures. However, structure **TS3 (*cis*/*Si*)** came out as **14.7 kJ mol^{-1}** higher in enthalpy than structure **TS2 (*cis*/*Re*;** or 15.1 **kJ mol^{-1}** higher in Gibbs free energy) and **62.8 kJ mol^{-1}** higher in enthalpy (or **57.7 kJ mol^{-1}** higher in Gibbs free energy) than structure **TS1 (*trans*/*Re*)** and. Although the *trans*/*Si* structure could not be computed, based on these results, it is likely that it would have come out higher in enthalpy compared to **TS1** (*trans*/*Re*), corroborating our empirical findings regarding preference for the *R*-enantiomer.

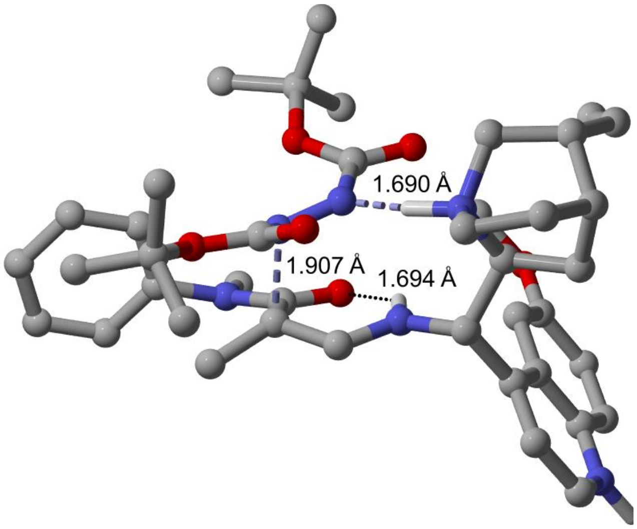

TS1 (*Re*-face) for *trans*-D^{t}BAD

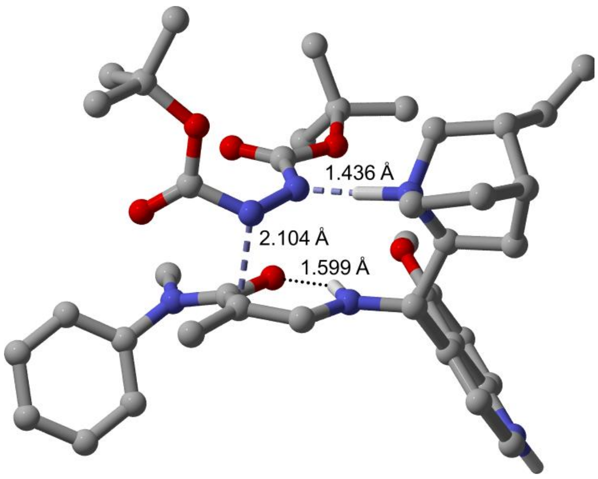

TS2 (*Re*-face) for *cis*-D^{t}BAD

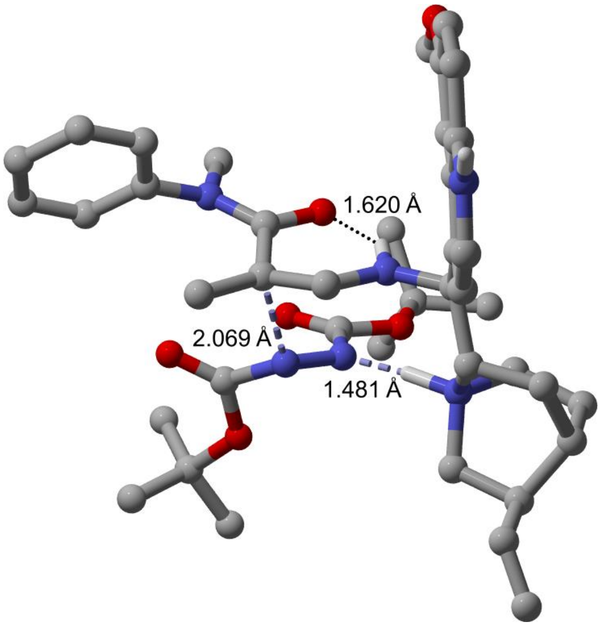

TS3 (*Si*-face) for *cis*-D*ᵗ*BAD

Figure S2: Transition state structures computed at the ωB97X-D/def2-SVP level of theory. Structures TS1 and TS2 lead to the *R*-hydrazide while structure TS3 leads to the *S*-hydrazide.

Gibbs activation free energies ($\Delta_r G^{\ddagger}$) were calculated as the difference between the Gibbs energy of the TS structure and that of the corresponding reactants consisting of the enaminone and D^{t}BAD, in either *cis*- or *trans*-configuration; note that the *trans*-configuration is 7.8 kJ mol^{-1} lower in enthalpy (or 10.5 kJ mol^{-1} lower in Gibbs free energy) than the *cis*-configuration. The resulting activation free energies are 8.1 kJ mol^{-1}, 40.2 kJ mol^{-1} and 55.3 kJ mol^{-1} for the *trans*/*Re*, c*is*/*Re* and *cis*/*Si* reactions, respectively. This is illustrated on the next page:

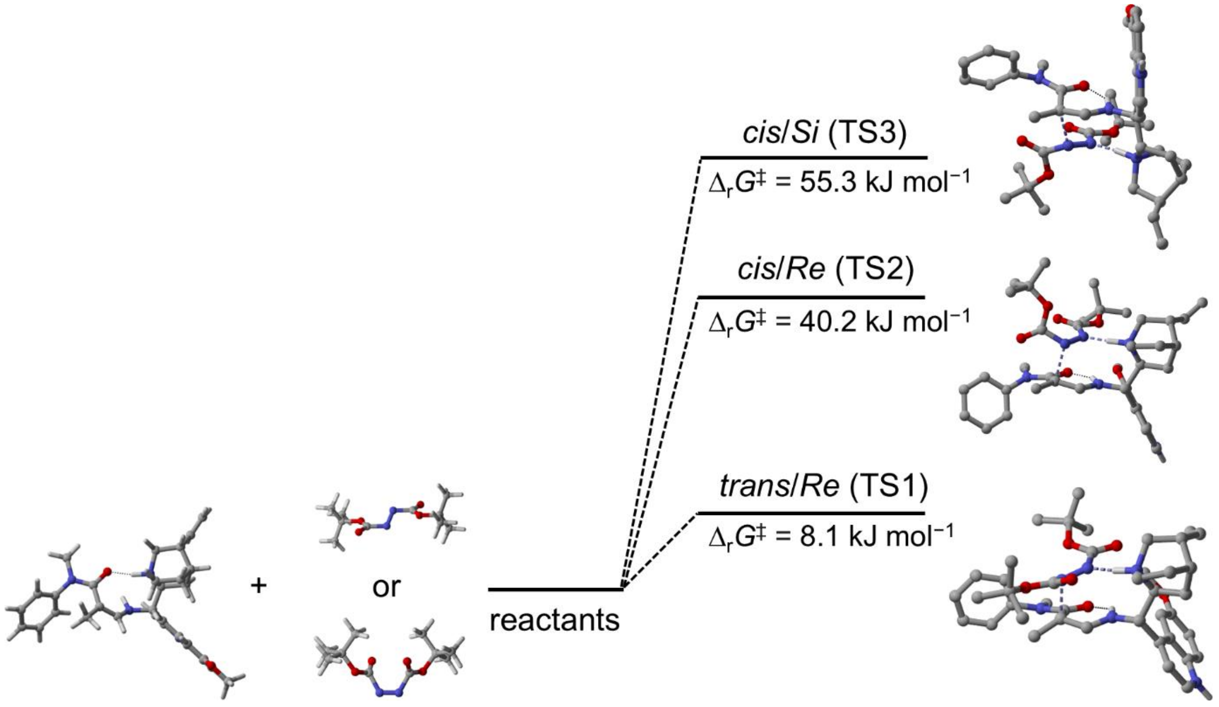

A mechanism for the hydrazination step of the reaction *via* **TS1** is shown in the mechanistic **Scheme** below together with a Dreiding model interpretation.

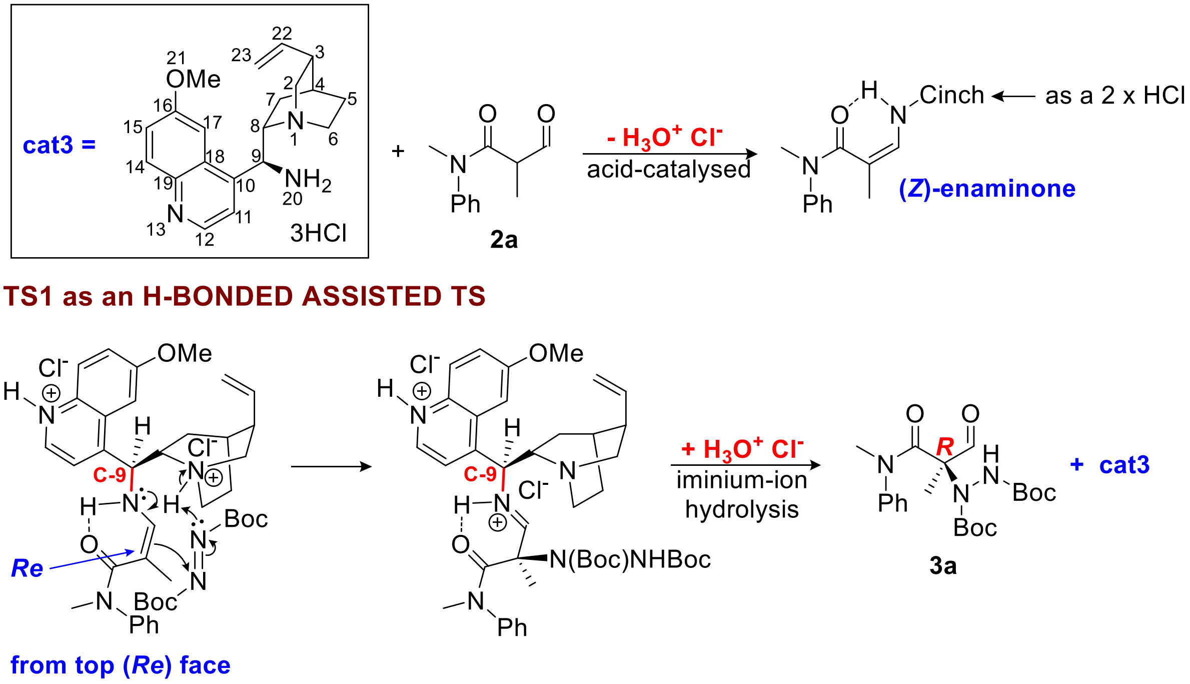

Mechanistic Scheme: Proposed hydrazination transition state using a Dreiding model.

....

8. X-Ray Crystallographic Data for compound 3h and 3m

Crystal-growth of **3h** and **3m** was performed via slow evaporation of a chloroform/hexane mixture. Single-crystal X-ray diffraction data were collected on a Bruker D8 Venture diffractometer using graphite-monochromated Cu-Kα radiation (χ = 1.54178 Å). Data collection was carried out at 100(2) K. Temperature was controlled by an Oxford Cryostream cooling system (Oxford Cryostat). Cell refinement and data reduction were performed using the program SAINT.[23] The data were scaled and absorption correction performed using SADABS.[23] The structure was solved by direct methods using SHELXS-97 and refined by full-matrix least-squares methods based on F^2 using SHELXL-2014 and using the graphics interface program X-Seed.[24, 25] The programs X-Seed and POV-Ray were used to prepare molecular graphic images.

For compound **3h**, all non-hydrogen atoms were refined anisotropically. All hydrogen atoms, except H3 on N3, were placed in idealized positions and refined in riding models with U_{iso} assigned 1.2 or 1.5 times U_{eq} of their parent atoms and the C-H bond distances were constrained range from 0.95 Å to 0.99 Å. The hydrogen H3 was located in the difference density maps and refined independently. The structure was refined to R factor of 0.0228. The Flack x parameter is 0.019(121) by classical fit to all intensities and -0.022(26) from 2274 selected quotients (Parsons' method). The absolute configuration could be determined reliably. Numbering Scheme: The ellipsoidal models were drawn at the probability level of 50%. The structure of **3h** is shown in **Figure S5**.

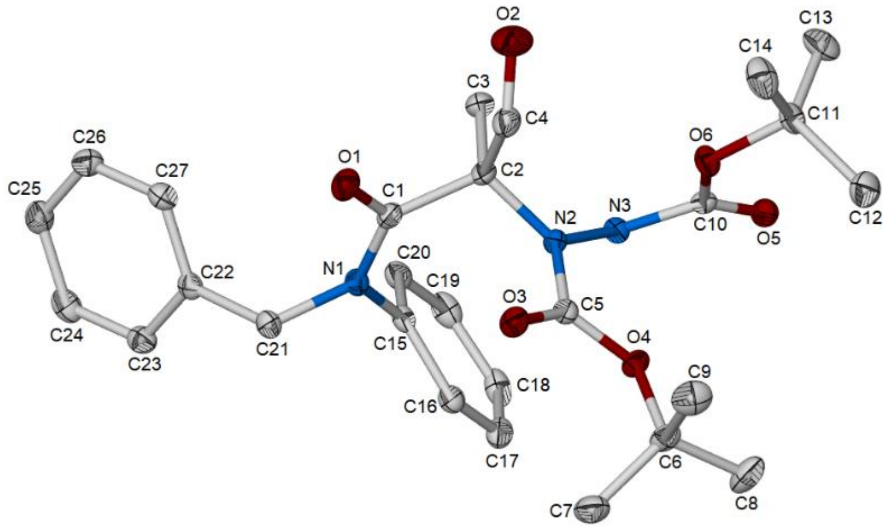

Figure S5: X-Ray structure for **3h**.

For compound **3m**, all non-hydrogen atoms were refined anisotropically. All hydrogen atoms, except the amino hydrogens, were placed in idealized positions and refined in riding models with U_{iso} assigned 1.2 or 1.5 times U_{eq} of their parent atoms and the C-H bond distances were constrained range from 0.95 Å to 0.99 Å. The hydrogen H3A and H3B were located in the difference density maps and refined independently. The structure was refined to R factor of 0.0361. The Flack x parameter is -0.038(144) by classical fit to all intensities and -0.007(104) from 3792 selected quotients (Parsons' method). The absolute configuration could be determined reliably. Numbering Scheme: The ellipsoidal models were drawn at the probability level of 50%. The structure of **3m** is shown in **Figure S6**.

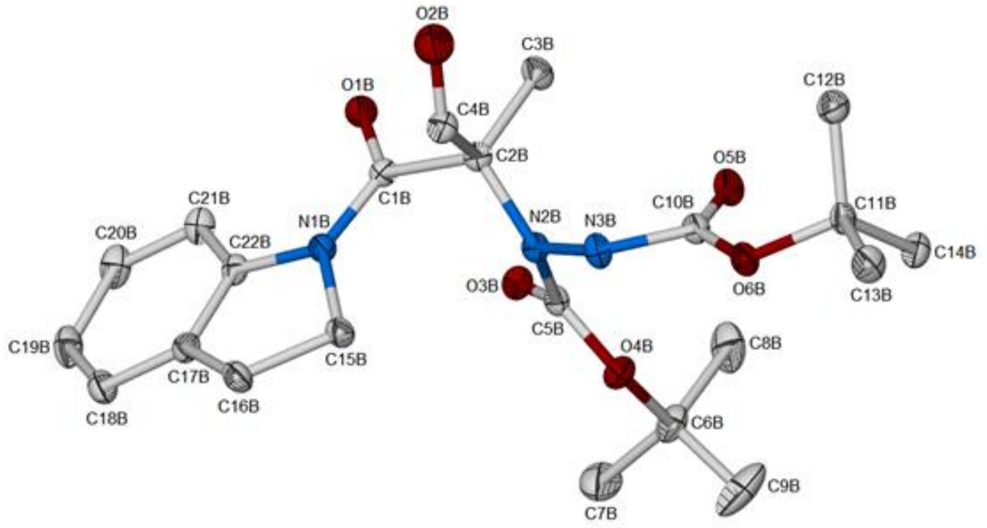

Figure S6: X-Ray structure for **3m**.

Table S2: Crystallographic data parameters of 3h and 3m compounds

**Compound**	**3h**	**3m**
Structural formula	$C_{27}H_{35}N_3O_6$	$C_{22}H_{31}N_3O_6$
Molecular mass ($g \cdot mol^{-1}$)	497.58	433.50
Data collection temp. (K)	100(2)	100(2)
Radiation (Å)	CuKa (1.54178)	CuKa (1.54178)
Crystal system	Orthorhombic	Monoclinic
Space group	$P2_12_12_1$	$P2_1$
a (Å)	11.4640(3)	10.0704(2)

b (Å)	14.3109(4)	20.1752(4)
c (Å)	16.3496(4)	11.3748(2)
α (°)	90	90
β (°)	90	90.196(1)
γ (°)	90	90
Volume ($Å^3$)	2682.32(12)	2311.03(8)
Z	4	4
Calc. density ($g \cdot cm^{-1}$)	1.232	1.246
μ (CuKα) (mm^{-1})	0.715	0.751
F(000)	1064	928
θ range (°)	4.9 - 74.4	4.5 - 75.0
HKL Index Range	-14: 14; -17: 17; -20: 20	-12: 12; -25: 25; -13: 13
Reflections collected	53064	55301
R_{int}	0.042	0.047
Data / Parameter	5435 / 336	9199 / 581
Final R indices ($I > 2\sigma(I)$)	0.0228	0.0361
R indices (all data)	0.0581	0.1033
Goodness-of-fit on F^2	1.05	1.08
Min. & Max. Resd. Dens. ($e \cdot Å^{-3}$)	-0.13, 0.17	-0.33, 0.83
Flack x parameter	-0.02(3)	-0.01(10)
CCDC accession codes	2283012	2283011

……

9. References

1. Shaffer, C. L.; Harriman, S.; Koen, Y. M.; Hanzlik, R. P., Formation of Cyclopropanone During Cytochrome P450-Catalyzed *N*-Dealkylation of a Cyclopropylamine. *J. Am. Chem. Soc.* **2002,** *124*, 8268-8274.
2. Dobah, F.; Mazodze, C. M.; Petersen, W. F., Cross-Dehydrogenative Cyclization–Dimerization Cascade Sequence for the Synthesis of Symmetrical 3, 3′-Bisoxindoles. *Org. Lett.* **2021,** *23*, 5466-5470.
3. Xu, S.; Zhang, Y.; Li, B.; Liu, S.-Y., Site-Selective and Stereoselective trans-Hydroboration of 1,3-Enynes Catalyzed by 1,4-Azaborine-Based Phosphine–Pd Complex. *J. Am. Chem. Soc.* **2016,** *138*, 14566-14569. .
4. Ghaffarzadeh, M.; Heidarifard, S.; Faraji, F.; Joghan, S. S., Synthesis of Amides From Imines Using Et_3SiH/Zn System. *Appl. Organomet. Chem.* **2012,** *26*, 103-107.
5. Kita, Y.; Akai, S.; Ajimura, N.; Yoshigi, M.; Tsugoshi, T.; Yasuda, H.; Tamura, Y., Facile and Efficient Syntheses of Carboxylic Anhydrides and Amides Using (Trimethylsilyl) Ethoxyacetylene. *J. Org. Chem.* **1986,** *51*, 4150-4158.5.
6. Li, W.-J.; Zhao, F.-F.; Ding, M.-W., Unexpected Synthesis of N-Acyl Indolines via a Consecutive Cyclization of Iminophosphorane. *Synlett* **2011,** *2011*, 265-267.
7. Singh, K.; Singh, K., N1, N3-Diacyl-3, 4-Dihydropyrimidin-2 (1H)-ones: Neutral Acyl Group Transfer Reagents. *Tetrahedron* **2009,** *65*, 10395-10399
8. Jankins, T. C.; Martin-Montero, R.; Cooper, P.; Martin, R.; Engle, K. M. Low-Valent Tungsten Catalysis Enables Site-Selective Isomerization–Hydroboration of Unactivated Alkenes. *J. Am. Chem. Soc.* **2021**, *143*, 14981-14986.
9. Tongdee, S.; Wei, D.; Wu, J.; Netkaew, C.; Darcel, C. Synthesis of Lactams by Reductive Amination of Carbonyl Derivatives with ω-Amino Fatty Acids under Hydrosilylation Conditions. *Eur. J. Org. Chem.* **2021**, *2021*, 5536-5539.
10. Santra, S.; Maji, U.; Guin, J. Enantioselective α-Amination of Acyclic 1, 3-Dicarbonyls Catalyzed by N-Heterocyclic Carbene. *Org. Lett.* **2020**, *22*, 468-473.
11. Anson, M. S.; Clark, H. F.; Evans, P.; Fox, M. E.; Graham, J. P.; Griffiths, N. N.; Meek, G.; Ramsden, J. A.; Roberts, A. J.; Simmonds, S. Complementary Syntheses of *N*, *O*-Protected-(*S*)-2-methylserine on a Multikilogram Scale. *Org. Process Res. Dev.* **2011**, *15*, 389-397.
12. Pracht, P.; Bohle, F.; Grimme, S., Automated exploration of the low-energy chemical space with fast quantum chemical methods. *Phys. Chem. Chem. Phys.* **2020,** *22*, 7169-7192.
13. Grimme, S., Exploration of Chemical Compound, Conformer, and Reaction Space with Meta-Dynamics Simulations Based on Tight-Binding Quantum Chemical Calculations. *J. Chem. Theory Comput.* **2019,** *15*, 2847-2862.
14. Bannwarth, C.; Ehlert, S.; Grimme, S., GFN2-xTB—An Accurate and Broadly Parametrized Self-Consistent Tight-Binding Quantum Chemical Method with Multipole Electrostatics and Density-Dependent Dispersion Contributions. *J. Chem. Theory Comput.* **2019,** *15*, 1652-1671.
15. Bannwarth, C.; Caldeweyher, E.; Ehlert, S.; Hansen, A.; Pracht, P.; Seibert, J.; Spicher, S.; Grimme, S., Extended tight-binding quantum chemistry methods. *WIREs Comput. Mol. Sci.* **2021,** *11*, e1493.
16. Ehlert, S.; Stahn, M.; Spicher, S.; Grimme, S., Robust and Efficient Implicit Solvation Model for Fast Semiempirical Methods. *J. Chem. Theory Comput.* **2021,** *17*, 4250-4261.
17. Chai, J.-D.; Head-Gordon, M., Long-range corrected hybrid density functionals with damped atom-atom dispersion corrections. *Phys. Chem. Chem. Phys.* **2008,** *10*, 6615-6620.
18. Weigend, F.; Ahlrichs, R., Balanced Basis Sets of Split Valence, Triple Zeta Valence and Quadruple Zeta Valence Quality for H to Rn: Design and Assessment of Accuracy. *Phys. Chem. Chem. Phys.* **2005,** *7*, 3297-3305.
19. Cossi, M.; Rega, N.; Scalmani, G.; Barone, V., Energies, structures, and electronic properties of molecules in solution with the C-PCM solvation model. *J. Comput. Chem.* **2003,** *24*, 669-681.
20. Barone, V.; Cossi, M., Quantum Calculation of Molecular Energies and Energy Gradients in Solution by a Conductor Solvent Model. *J. Phys. Chem. A* **1998,** *102*, 1995-2001.
21. Grimme, S., Supramolecular Binding Thermodynamics by Dispersion-Corrected Density Functional Theory. *Chem. Eur. J.* **2012,** *18*, 9955-9964.
22. Frisch, M. J.; Trucks, G. W.; Schlegel, H. B.; Scuseria, G. E.; Robb, M. A.; Cheeseman, J. R.; Scalmani, G.; Barone, V.; Petersson, G. A.; Nakatsuji, H.; Li, X.; Caricato, M.; Marenich, A. V.; Bloino, J.; Janesko, B. G.; Gomperts, R.; Mennucci, B.; Hratchian, H. P.; Ortiz, J. V.; Izmaylov, A. F.; Sonnenberg, J. L.; Williams; Ding, F.; Lipparini, F.; Egidi, F.; Goings, J.; Peng, B.; Petrone, A.; Henderson, T.; Ranasinghe, D.; Zakrzewski, V. G.; Gao, J.; Rega, N.; Zheng, G.; Liang, W.; Hada, M.; Ehara, M.; Toyota, K.; Fukuda, R.; Hasegawa, J.; Ishida, M.; Nakajima, T.; Honda, Y.; Kitao, O.; Nakai, H.; Vreven, T.; Throssell, K.; Montgomery Jr., J. A.; Peralta, J. E.; Ogliaro, F.; Bearpark, M. J.; Heyd, J. J.; Brothers, E. N.; Kudin, K. N.; Staroverov, V. N.; Keith, T. A.; Kobayashi, R.; Normand, J.; Raghavachari, K.; Rendell, A. P.; Burant, J. C.; Iyengar, S. S.; Tomasi, J.; Cossi, M.; Millam, J. M.; Klene, M.; Adamo, C.; Cammi, R.; Ochterski, J. W.; Martin, R. L.; Morokuma, K.; Farkas, O.; Foresman, J. B.; Fox, D. J. *Gaussian 16 Rev. C.01*: Wallingford, CT, 2016.
23. Bruker, A.; Saint, A., Inc., Madison, WI, 2004 Search PubMed;(b) GM Sheldrick. *Acta Crystallogr., Sect. A: Fundam. Crystallogr* **2008,** *64*, 112.
24. Barbour, L. J., X-Seed — A Software Tool for Supramolecular Crystallography. *Supramol Chem* **2001,** *1*, 189-191.
25. Atwood, J. L.; Barbour, L. J., Molecular Graphics: From Science to Art. *Cryst. Growth Des.* **2003,** *3*, 3-8.

……

10. NMR spectra

(i) 1a-u

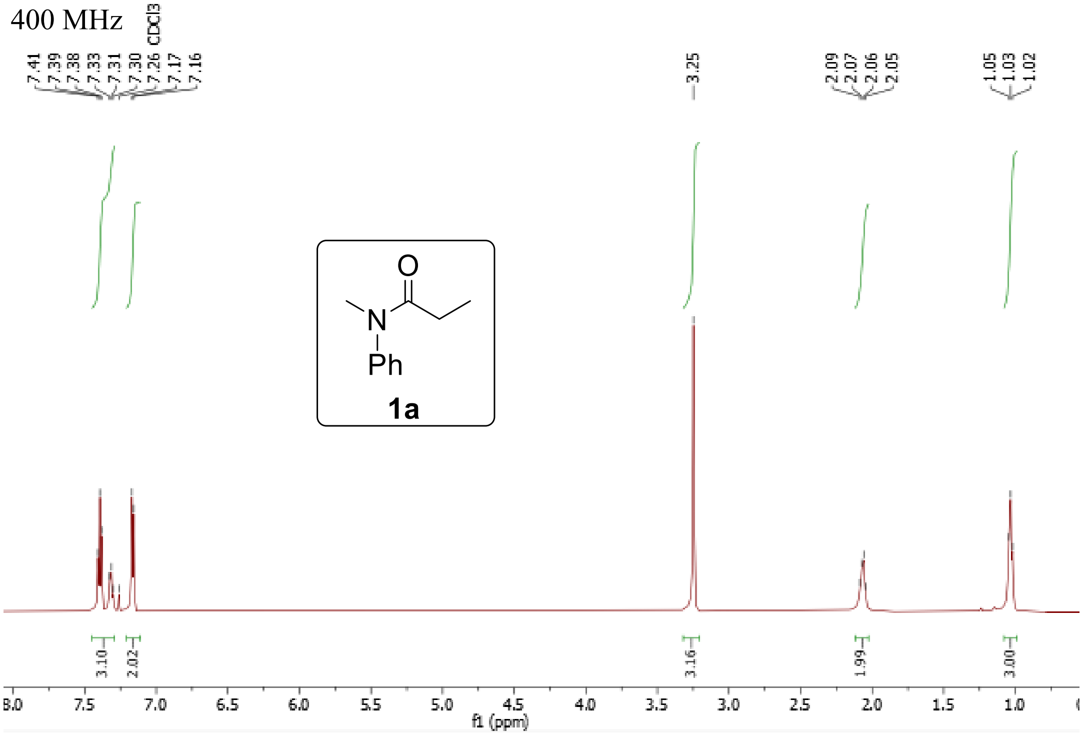

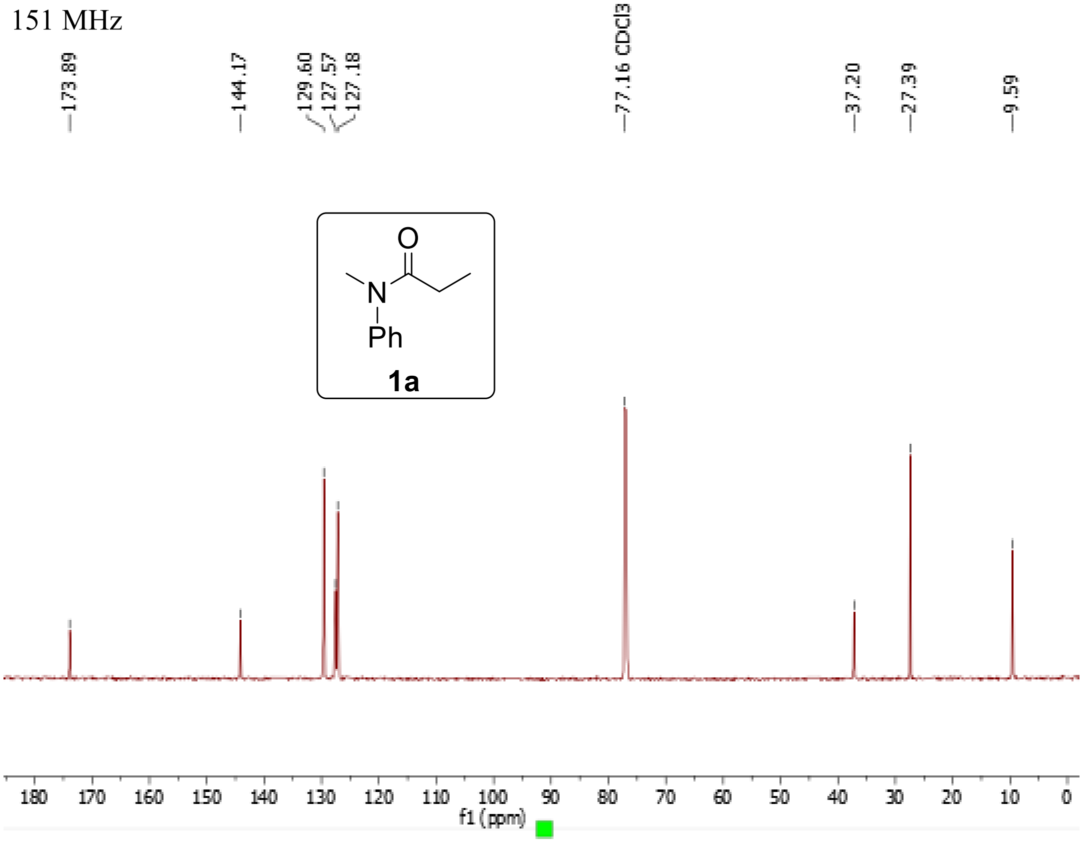

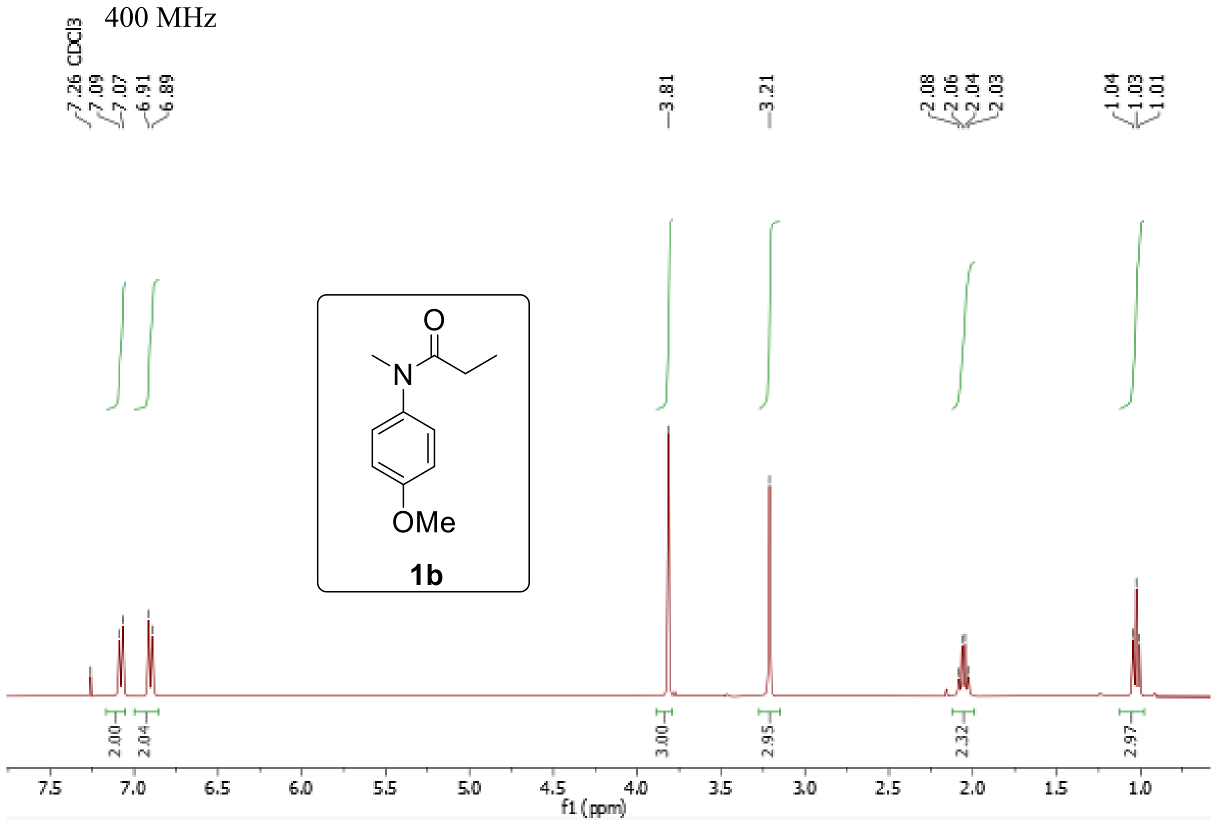
400 MHz
7.26 CDCl3
7.09
7.07
6.91
6.89
3.81
3.21
2.08
2.06
2.04
2.03
1.04
1.03
1.01
N
O
OMe
1b
2.00
2.04
3.00
2.95
2.32
2.97
f1 (ppm)

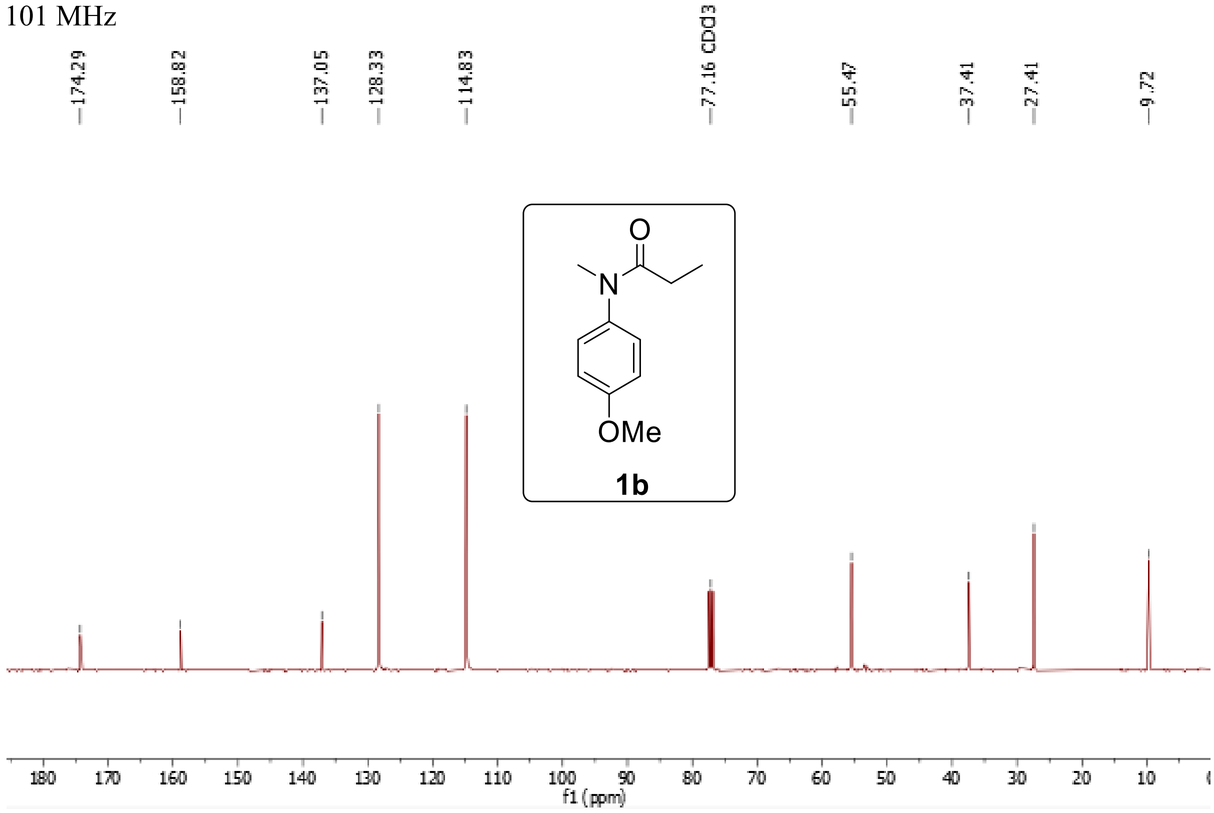
101 MHz
174.29
158.82
137.05
128.33
114.83
77.16 CDCl3
55.47
37.41
27.41
9.72
N
O
OMe
1b
f1 (ppm)

400 MHz

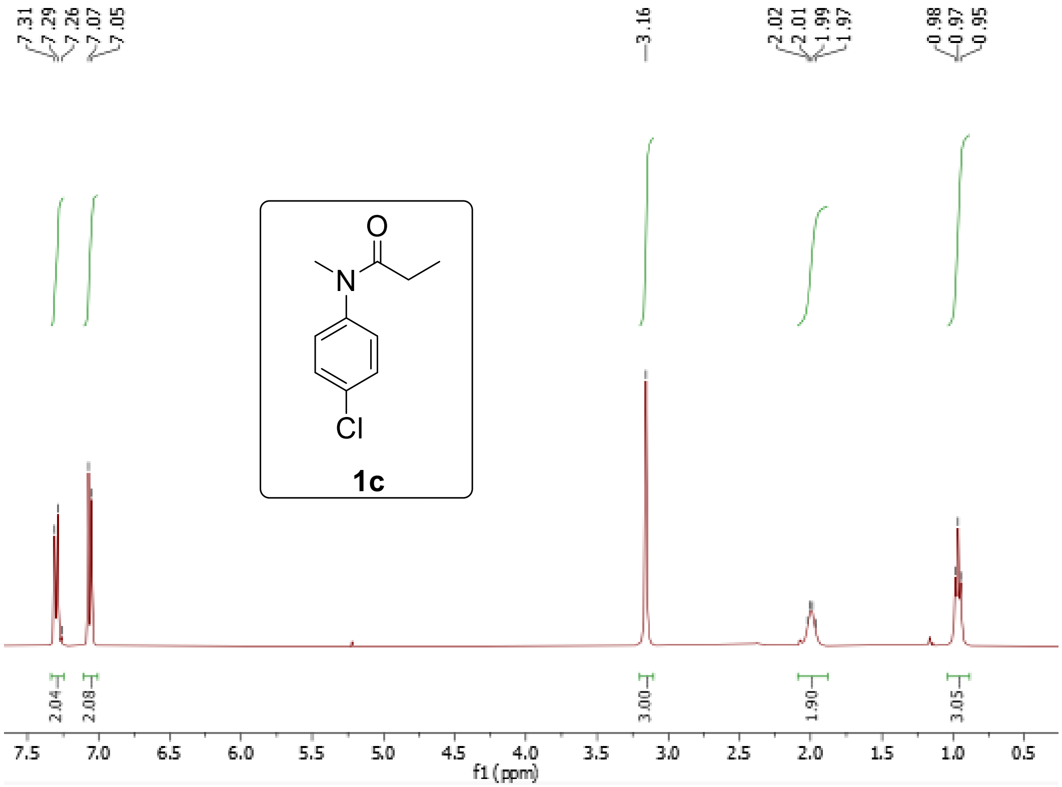

101 MHz

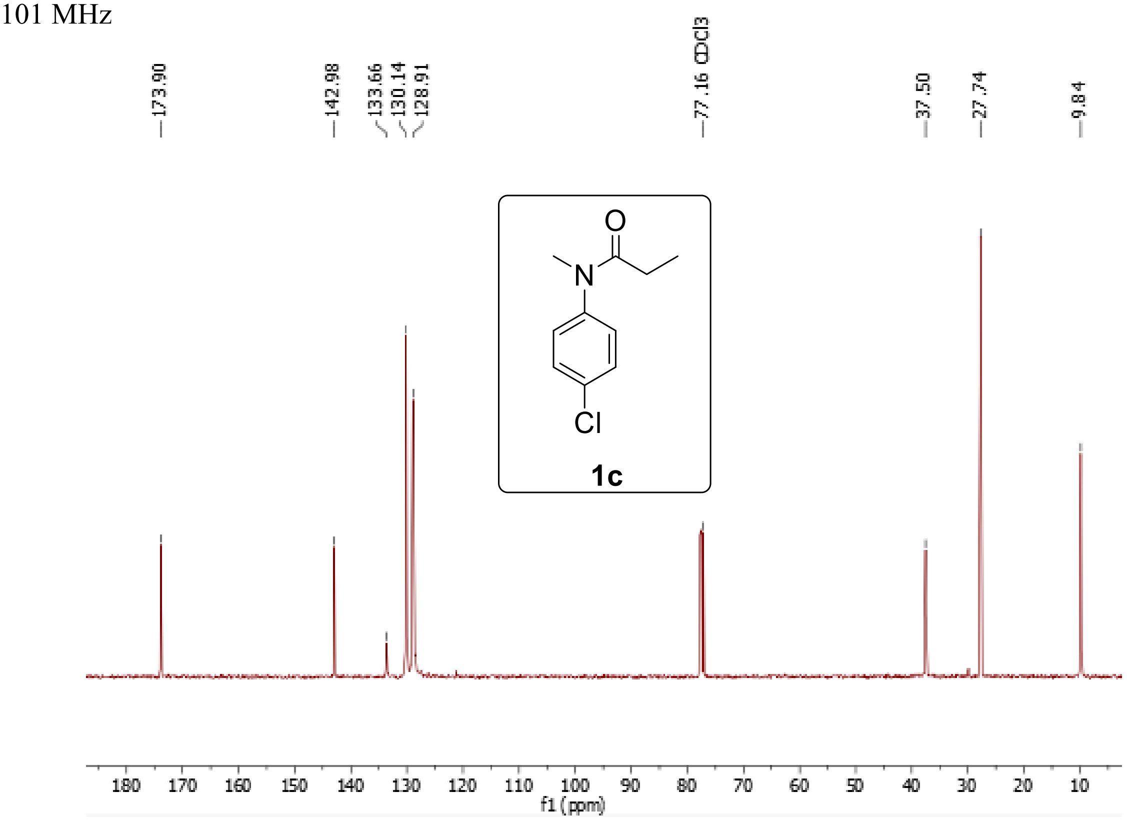

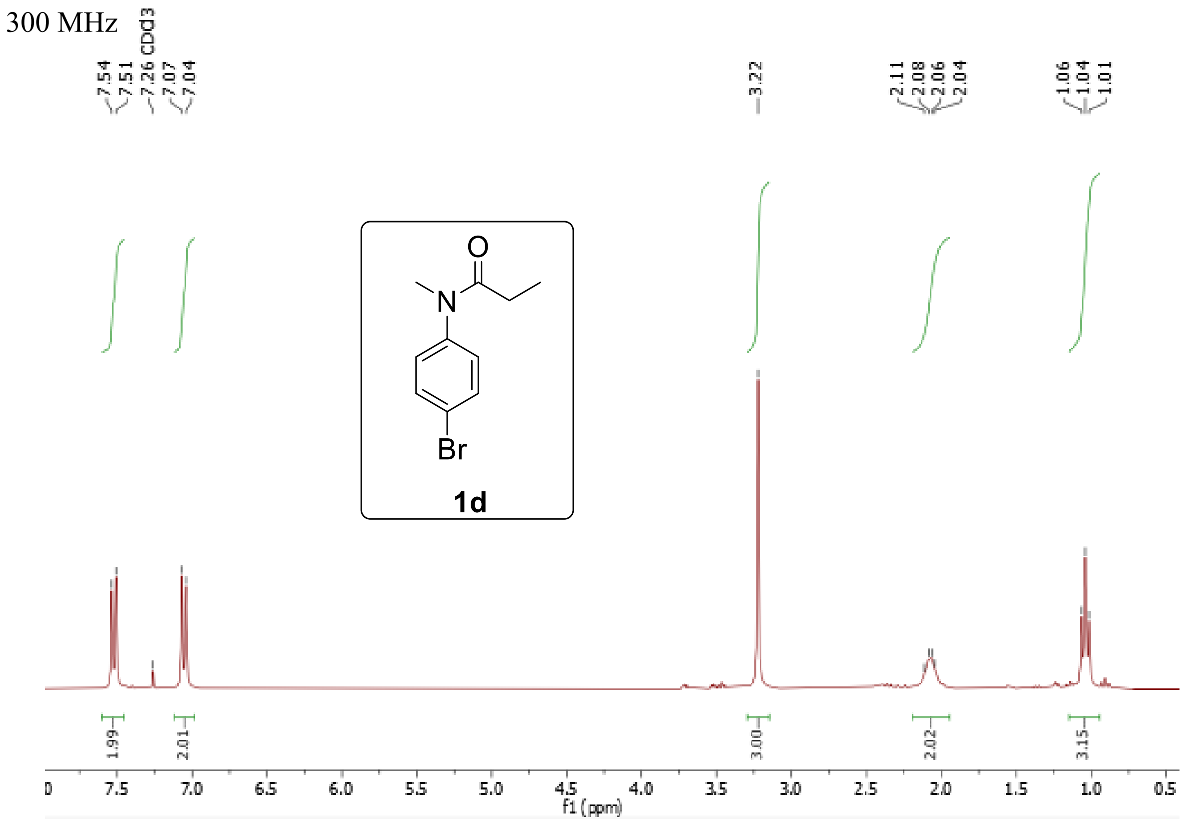
300 MHz
7.54
7.51
7.26 CDCl3
7.07
7.04
3.22
2.11
2.08
2.06
2.04
1.06
1.04
1.01
O
N
Br
1d
1.99
2.01
3.00
2.02
3.15
7.5
7.0
6.5
6.0
5.5
5.0
4.5
4.0
3.5
3.0
2.5
2.0
1.5
1.0
0.5
f1 (ppm)

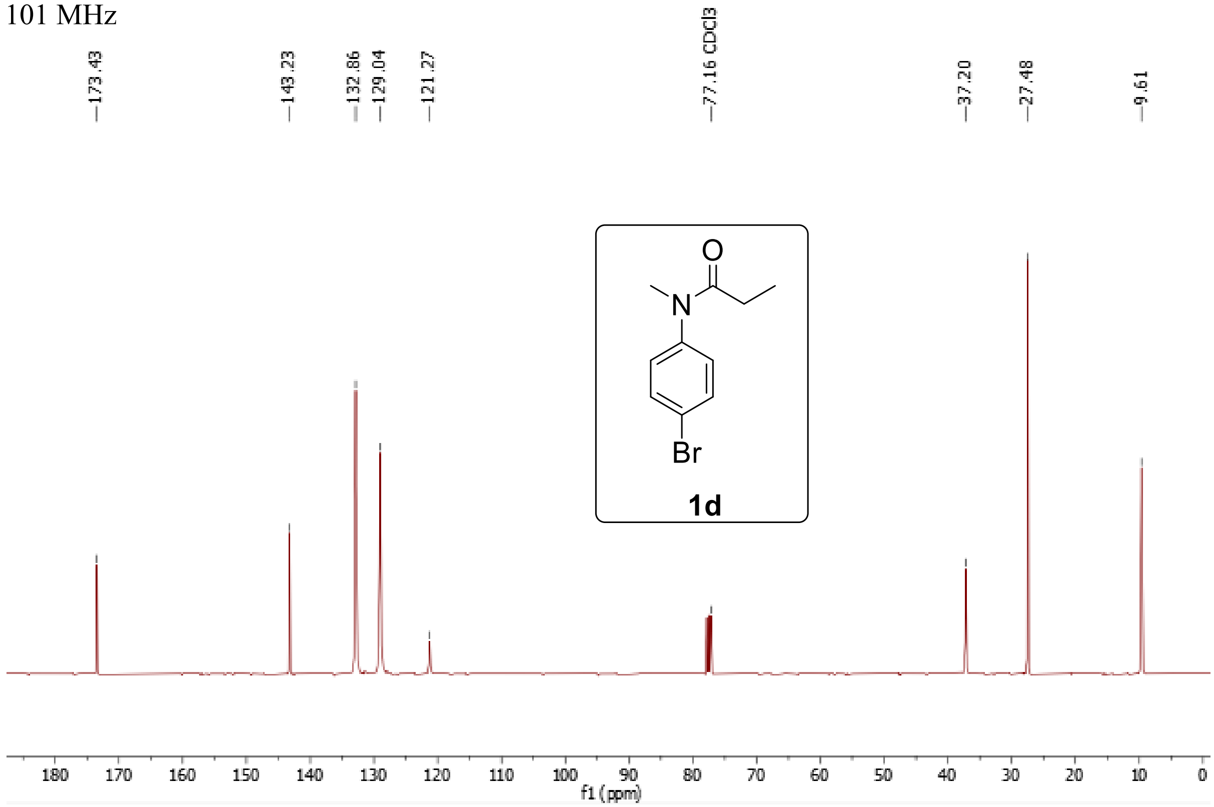
101 MHz
173.43
143.23
132.86
129.04
121.27
77.16 CDCl3
37.20
27.48
9.61
O
N
Br
1d
180
170
160
150
140
130
120
110
100
90
80
70
60
50
40
30
20
10
0
f1 (ppm)

400 MHz

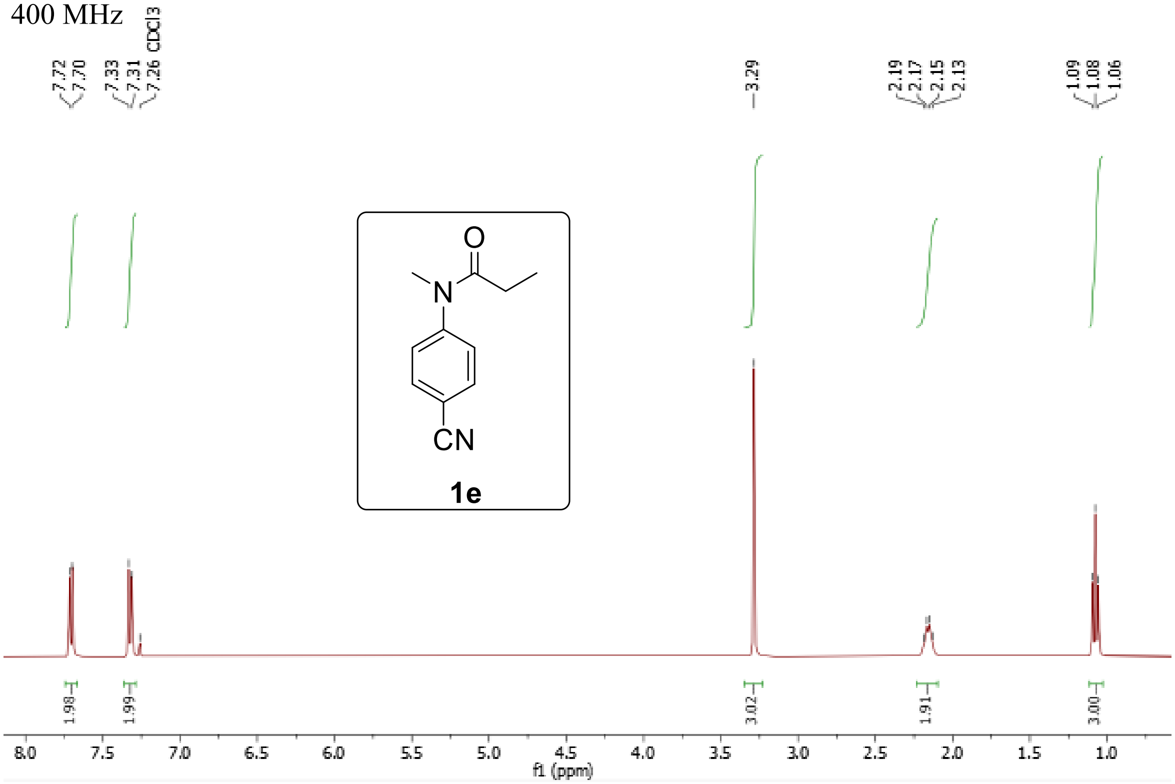

101 MHz

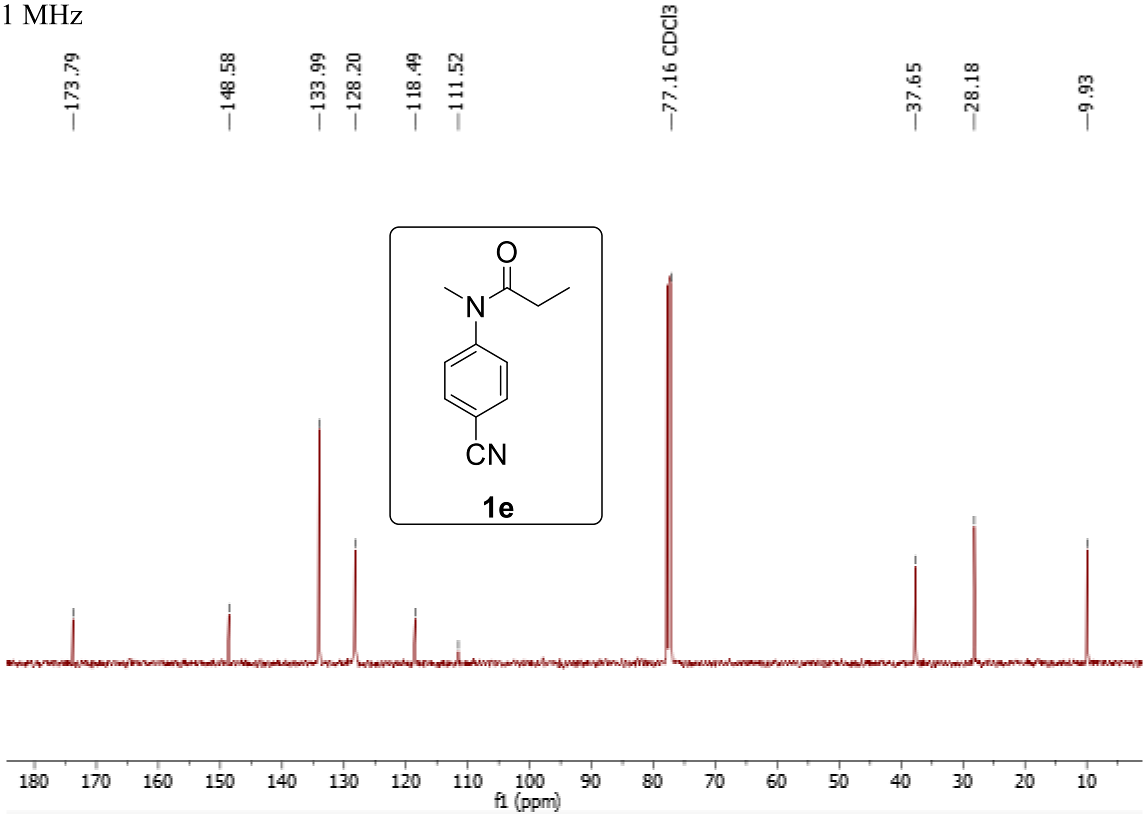

300 MHz

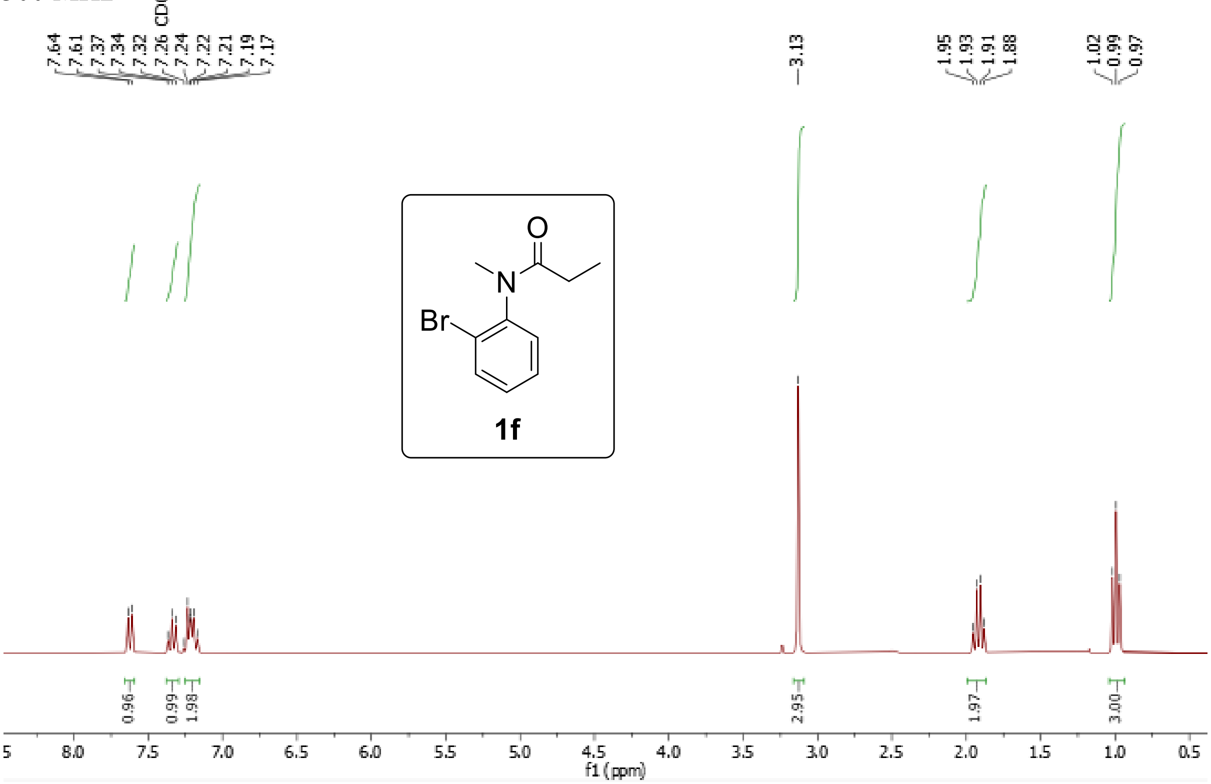

101 MHz

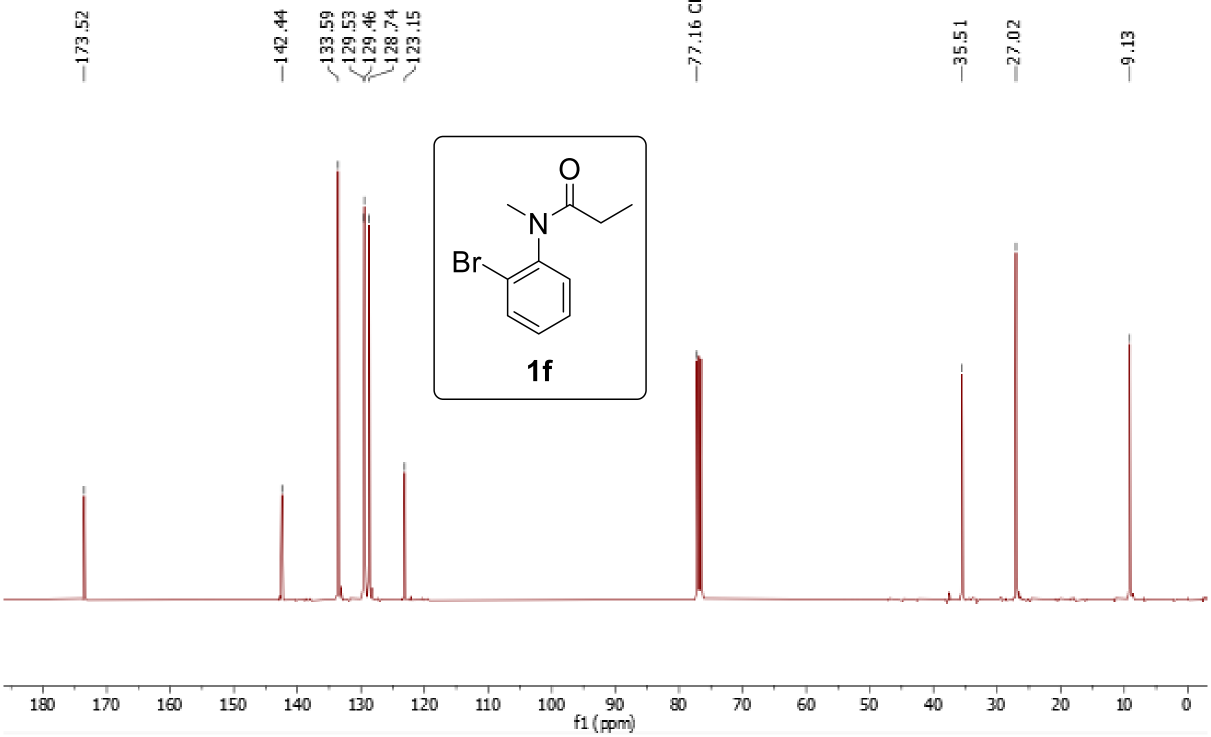

400 MHz

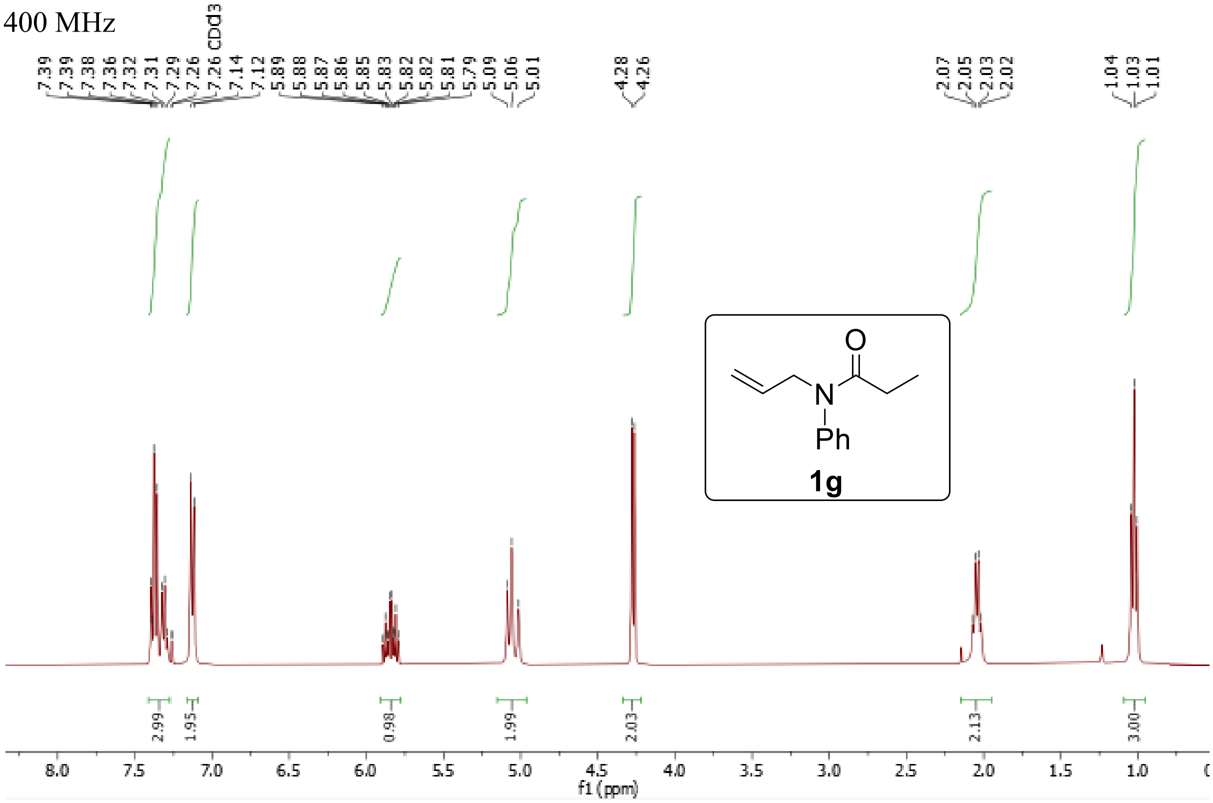

101 MHz

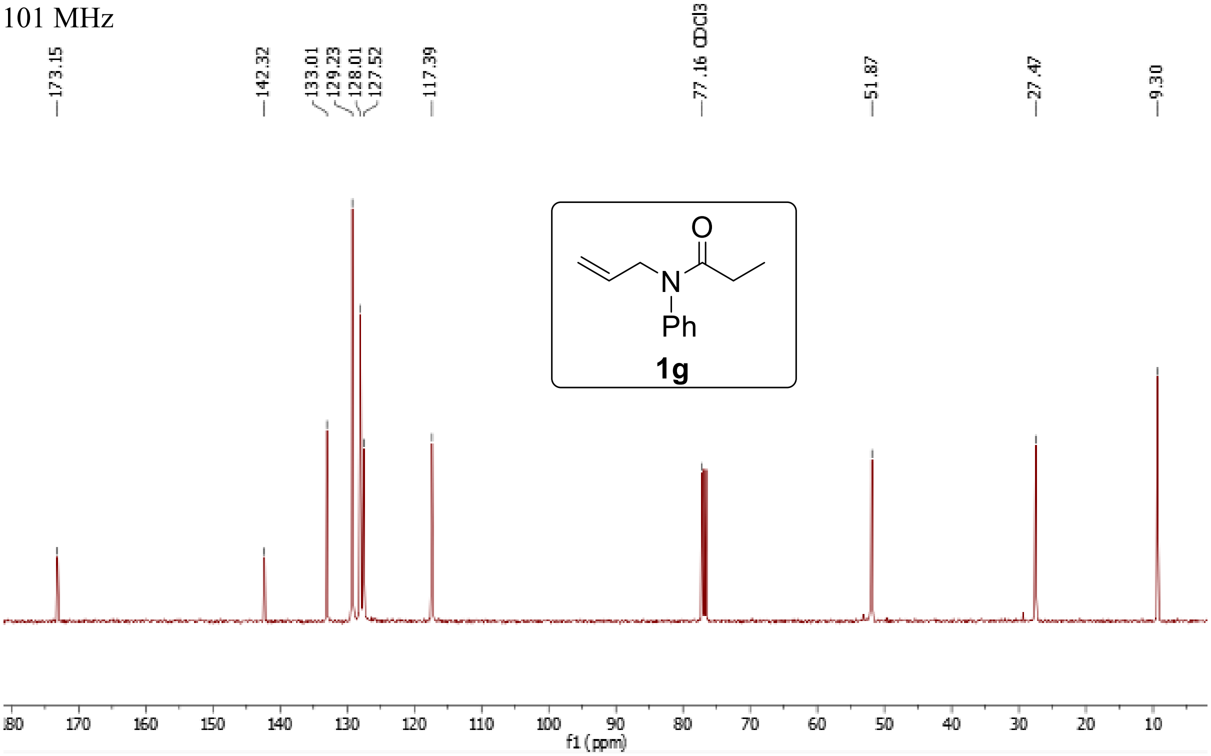

400 MHz

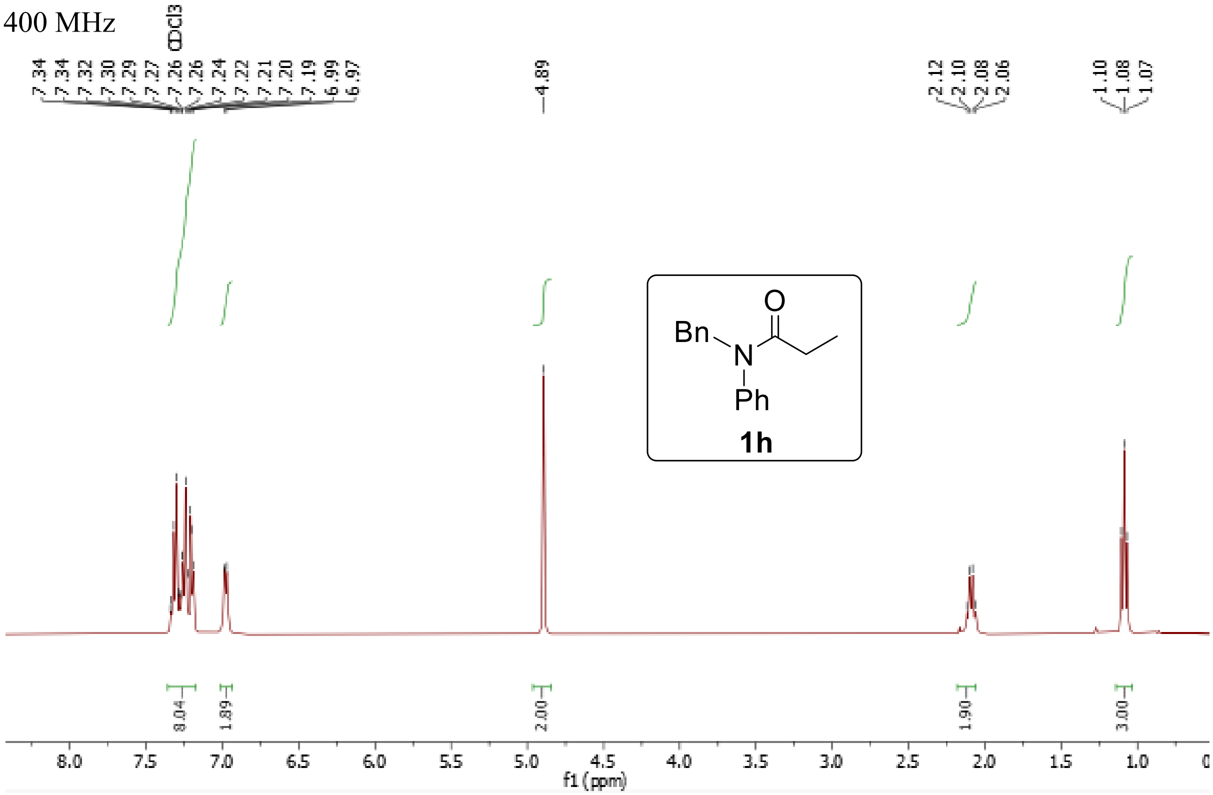

101 MHz

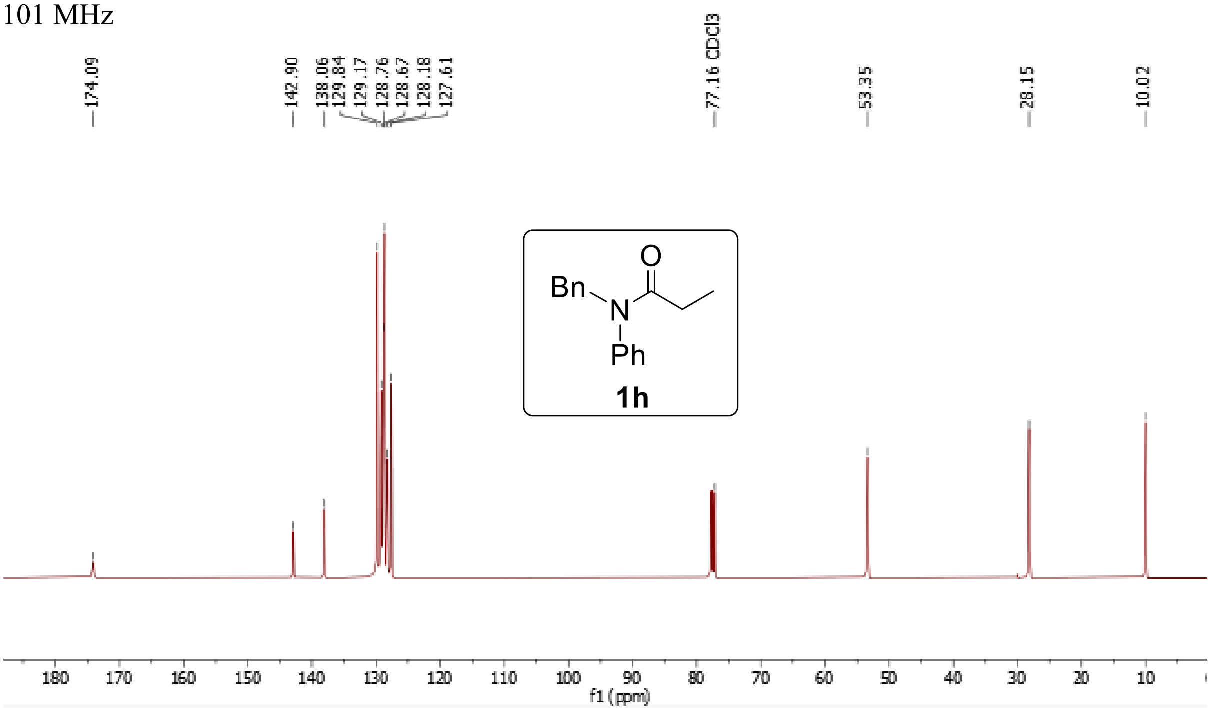

300 MHz

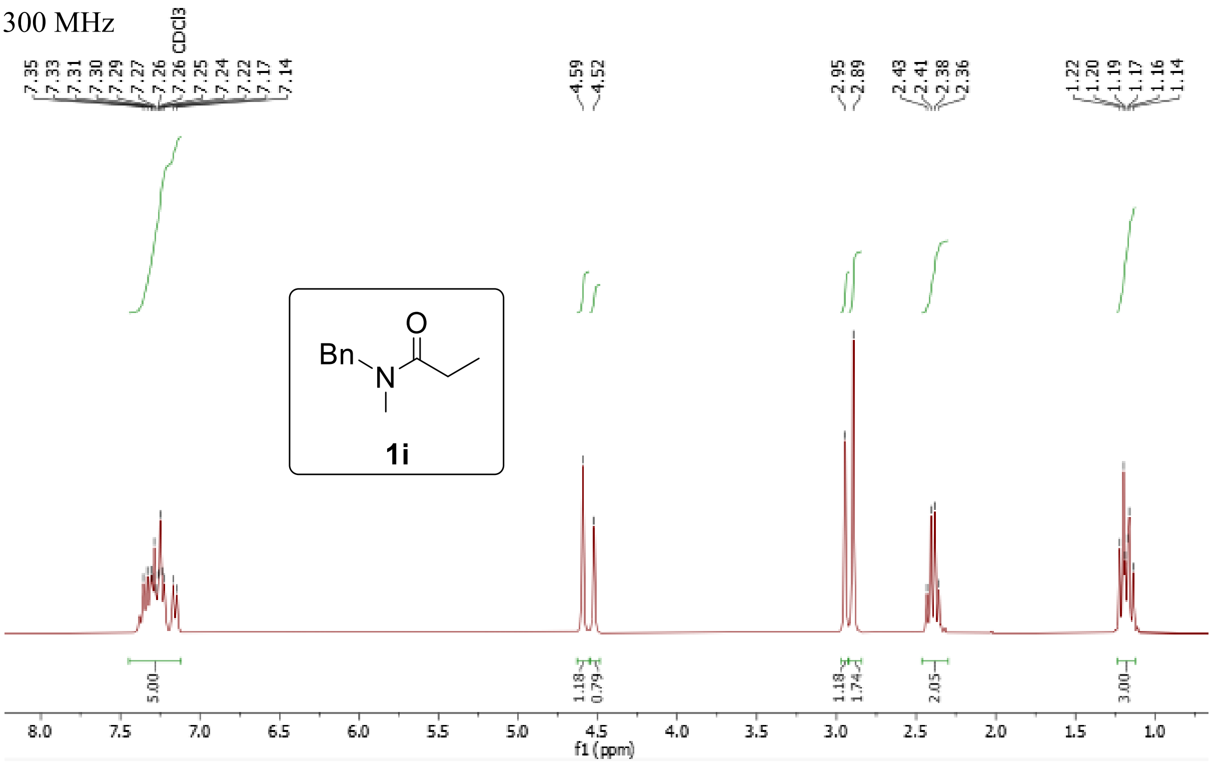

101 MHz

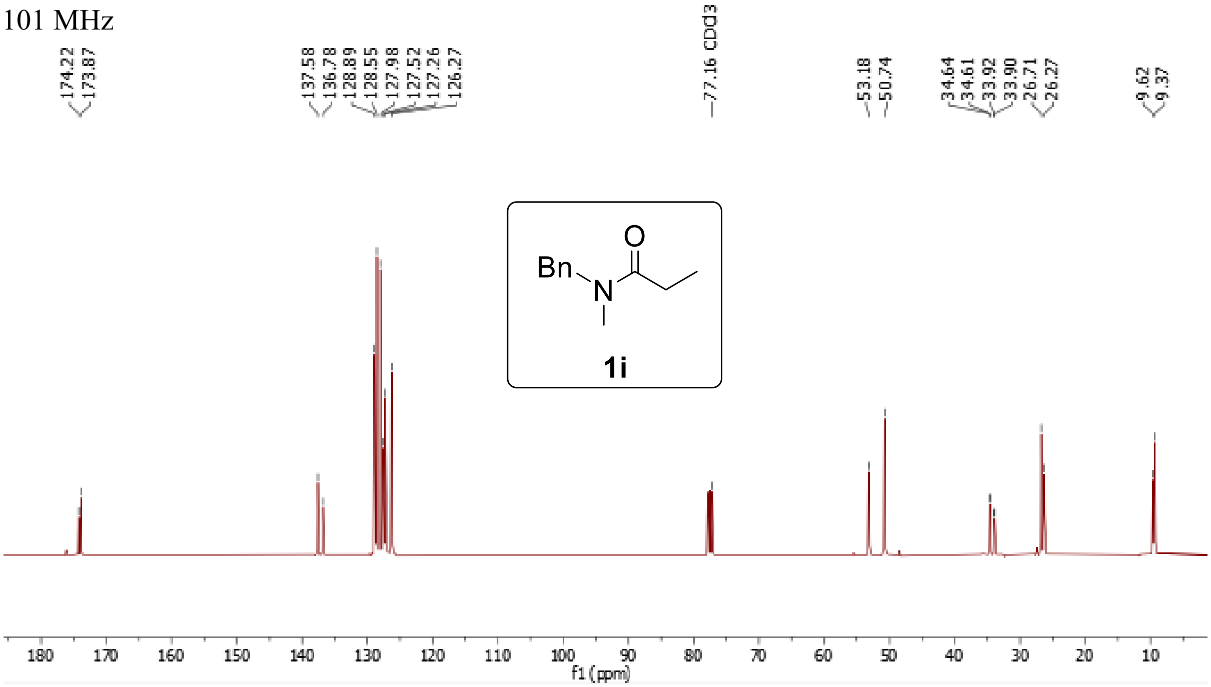

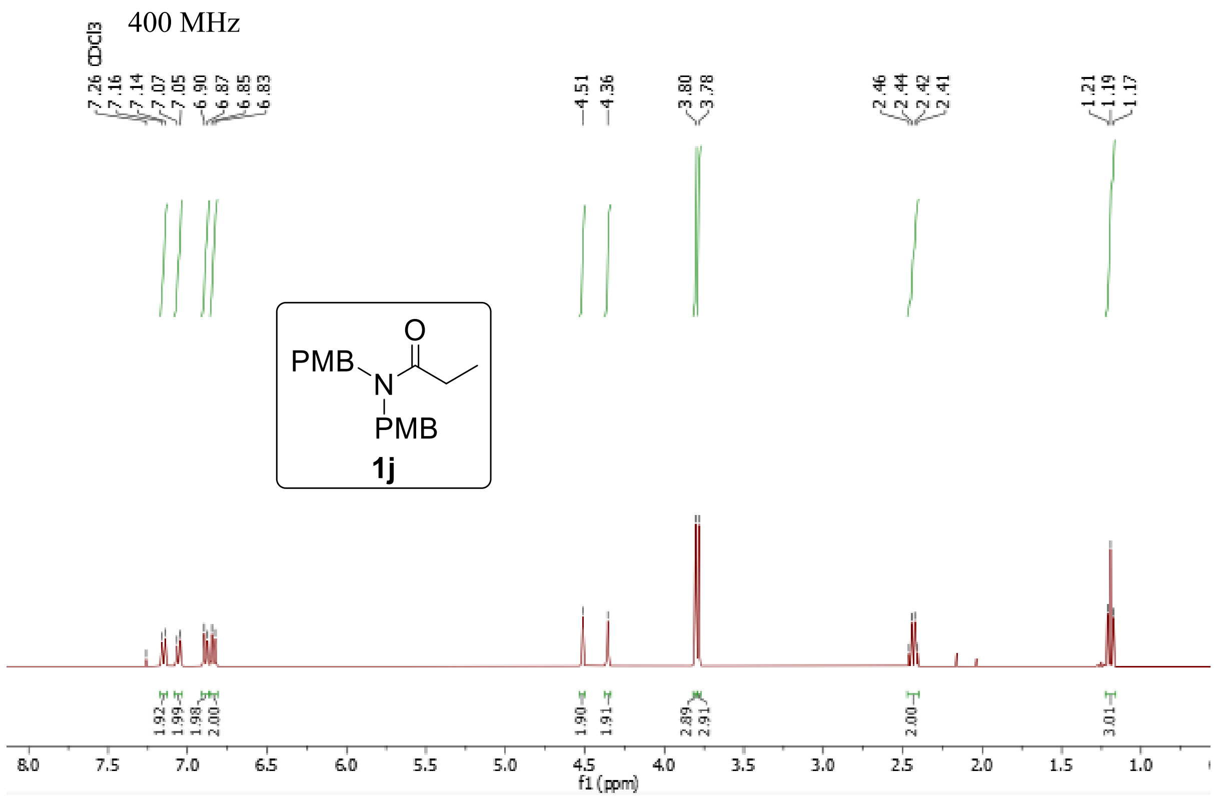
400 MHz
CDCl3
7.26 7.16 7.14 7.07 7.05 6.90 6.87 6.85 6.83
4.51 4.36
3.80 3.78
2.46 2.44 2.42 2.41
1.21 1.19 1.17
PMB
N
O
PMB
1j
1.92 1.99 1.98 2.00
1.90 1.91
2.89 2.91
2.00
3.01
f1 (ppm)

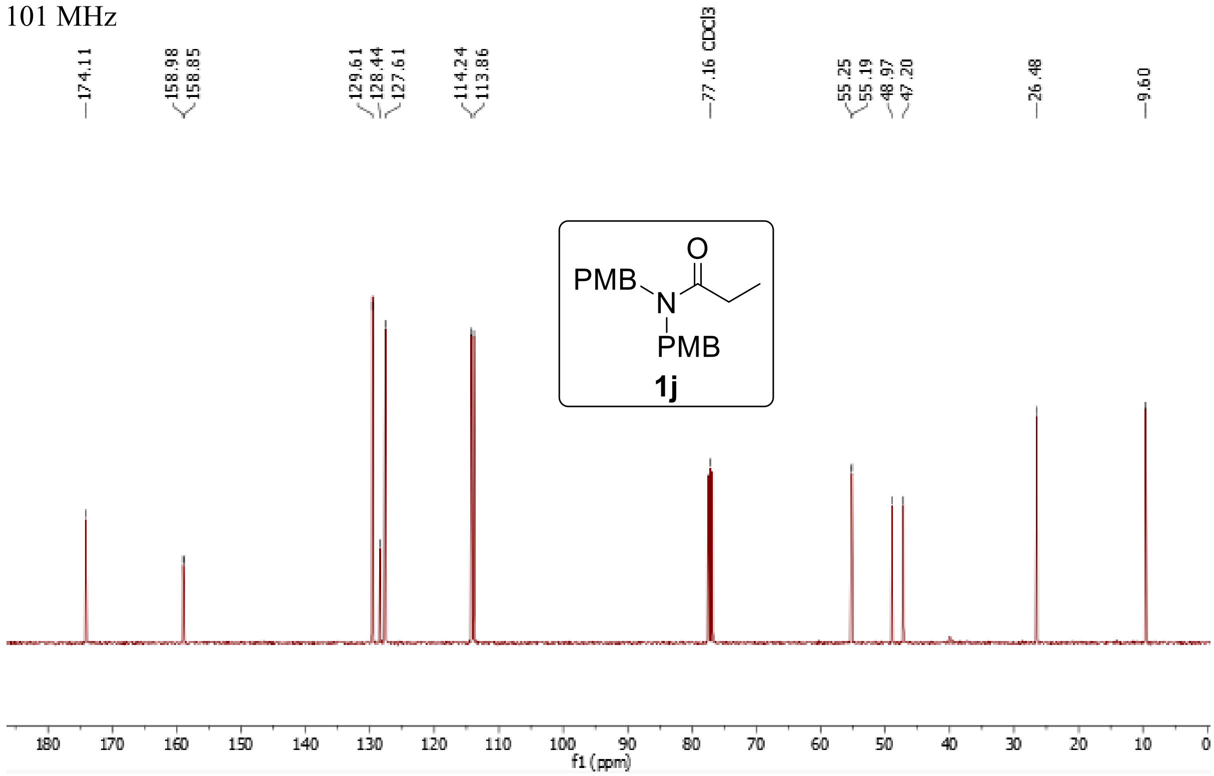
101 MHz
174.11
158.98 158.85
129.61 128.44 127.61
114.24 113.86
77.16 CDCl3
55.25 55.19 48.97 47.20
26.48
9.60
PMB
N
O
PMB
1j
f1 (ppm)

300 MHz

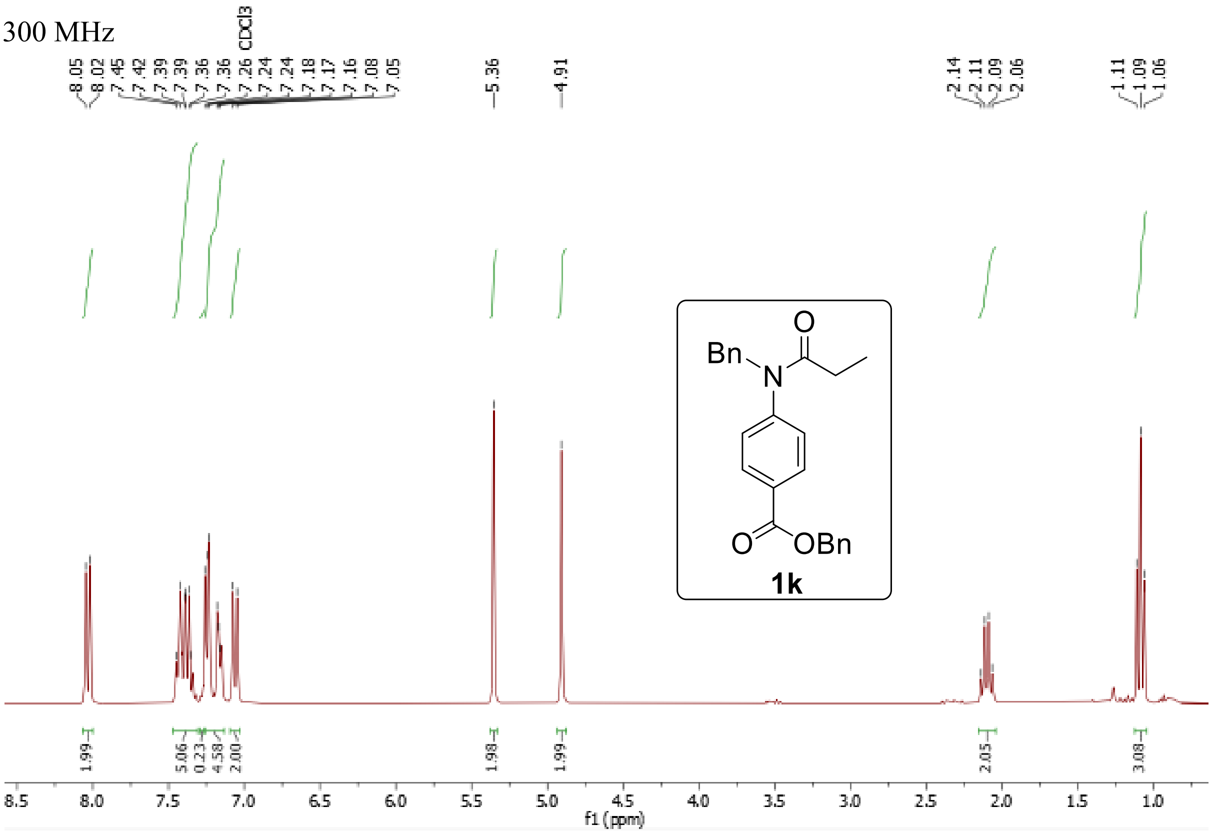

151 MHz

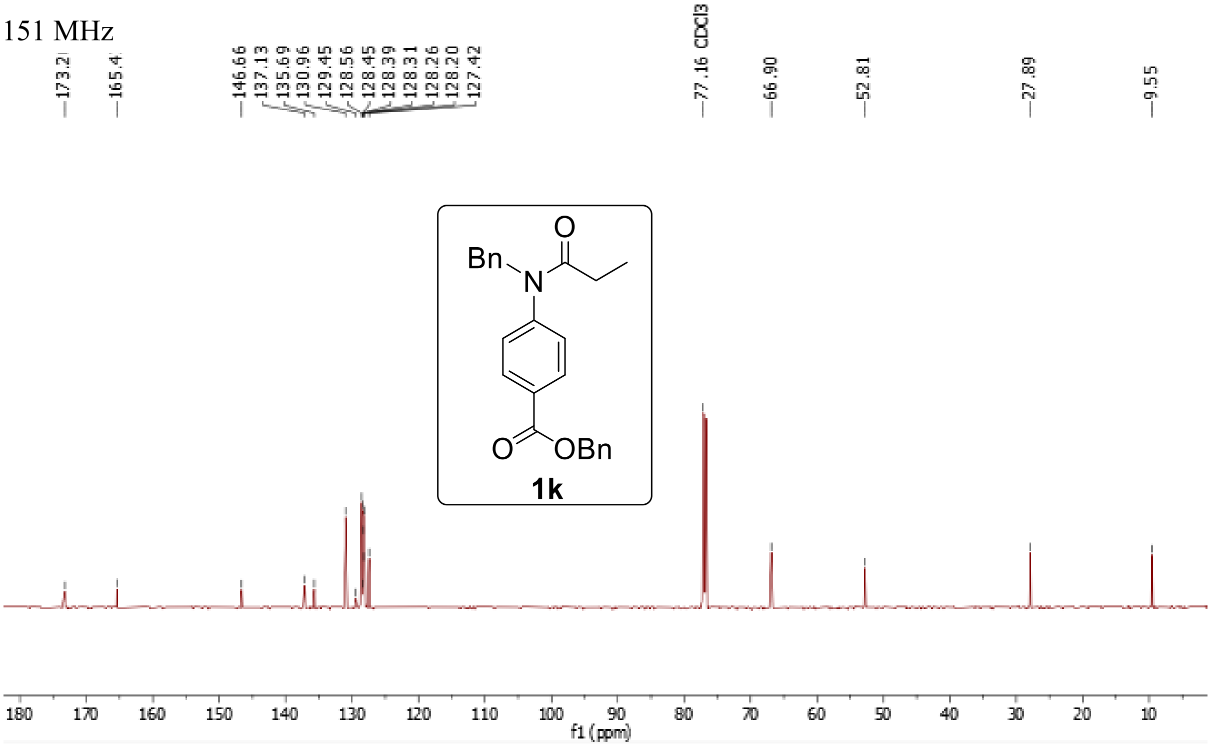

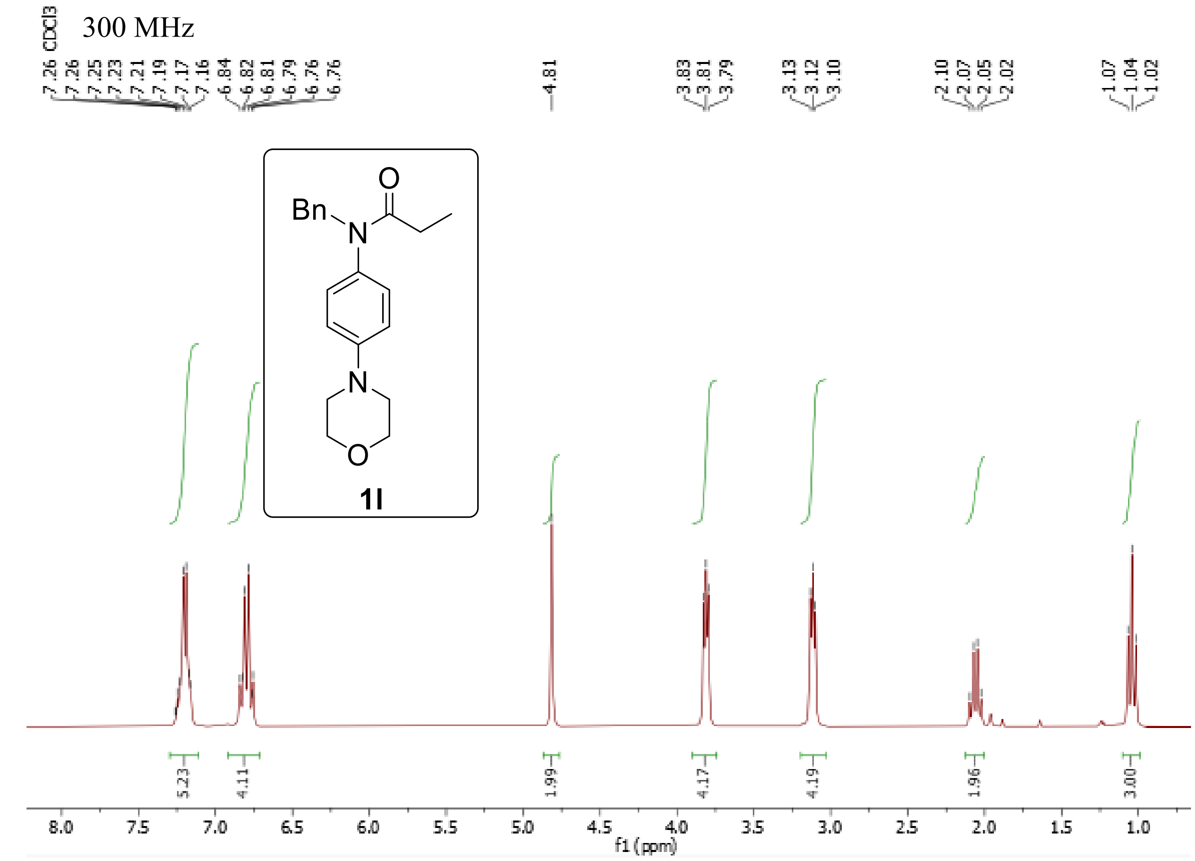
300 MHz
7.26 CDCl3
7.26
7.25
7.23
7.21
7.19
7.17
7.16
6.84
6.82
6.81
6.79
6.76
6.76
4.81
3.83
3.81
3.79
3.13
3.12
3.10
2.10
2.07
2.05
2.02
1.07
1.04
1.02
Bn
N
O
N
O
1l
5.23
4.11
1.99
4.17
4.19
1.96
3.00
8.0
7.5
7.0
6.5
6.0
5.5
5.0
4.5
4.0
3.5
3.0
2.5
2.0
1.5
1.0
f1 (ppm)

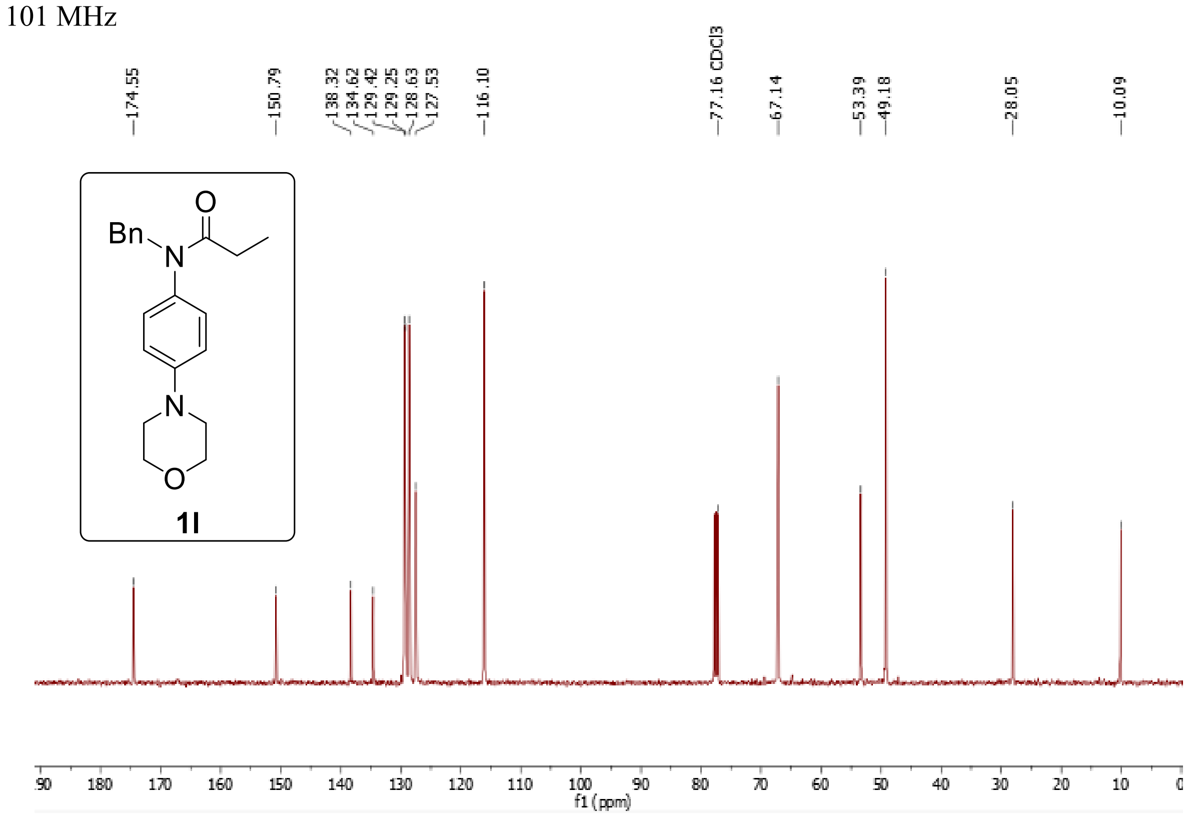
101 MHz
174.55
150.79
138.32
134.62
129.42
129.25
128.63
127.53
116.10
77.16 CDCl3
67.14
53.39
49.18
28.05
10.09
Bn
N
O
N
O
1l
180
170
160
150
140
130
120
110
100
90
80
70
60
50
40
30
20
10
f1 (ppm)

300 MHz

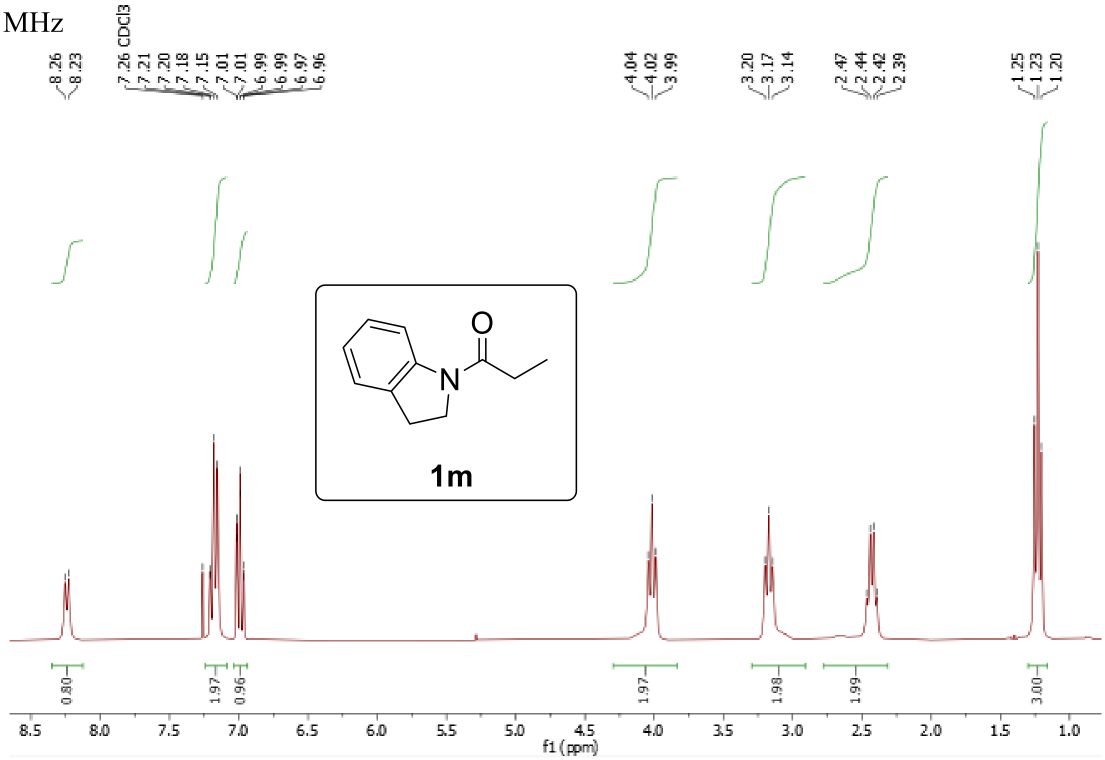

151 MHz

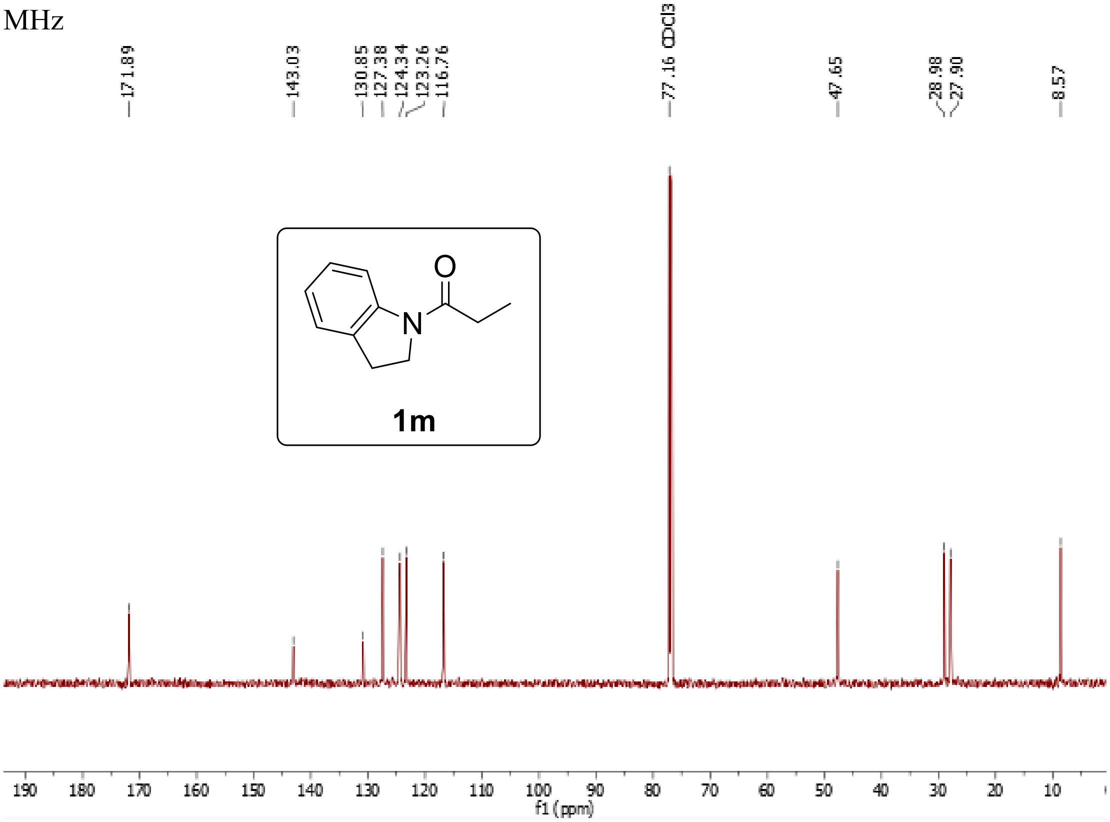

300 MHz

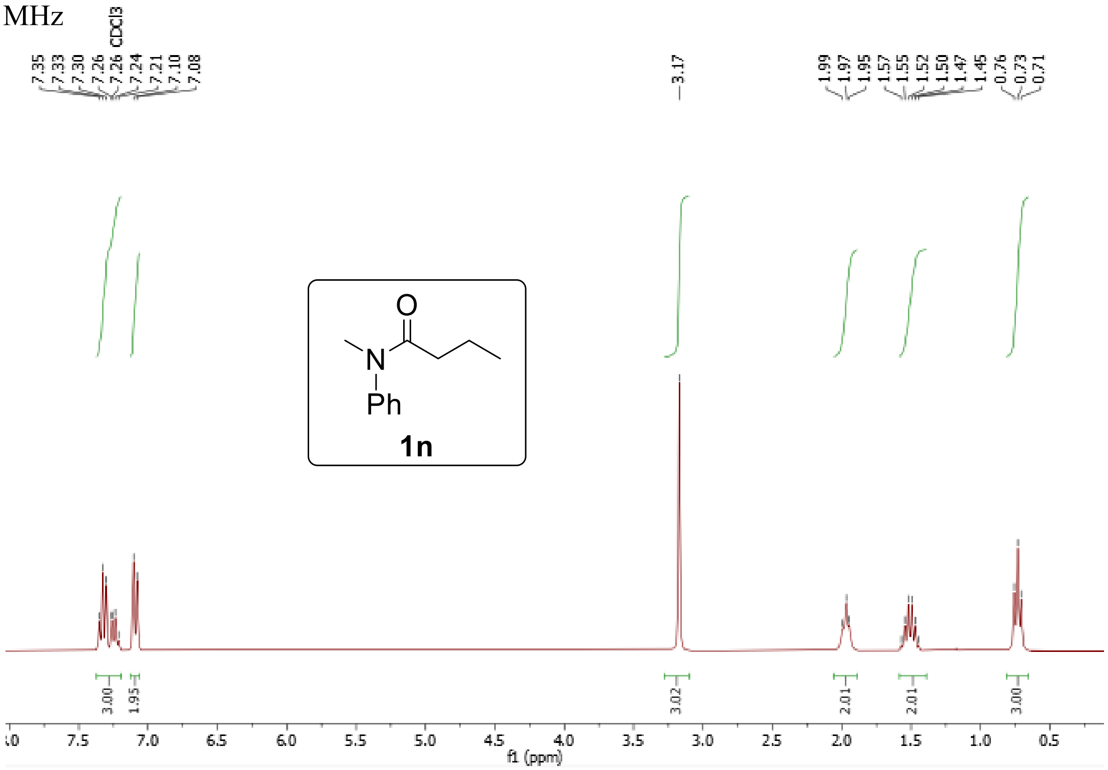

151 MHz

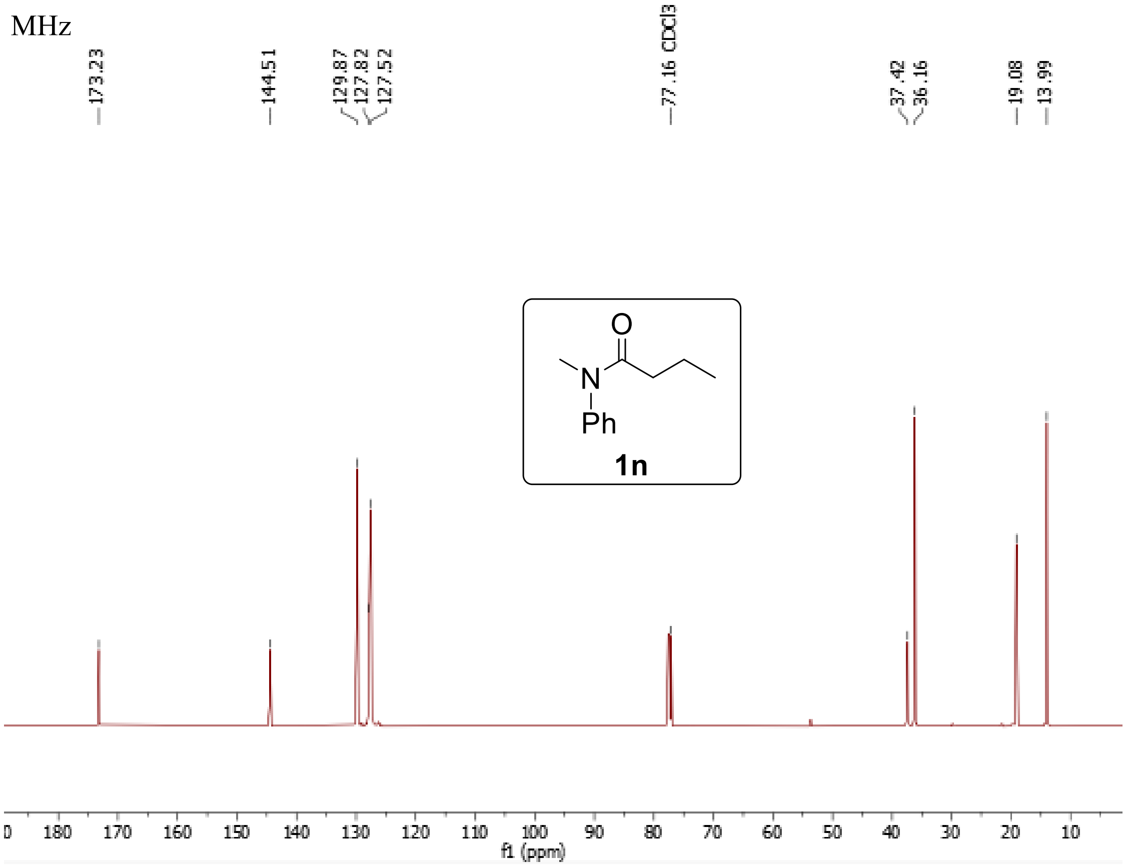

400 MHz

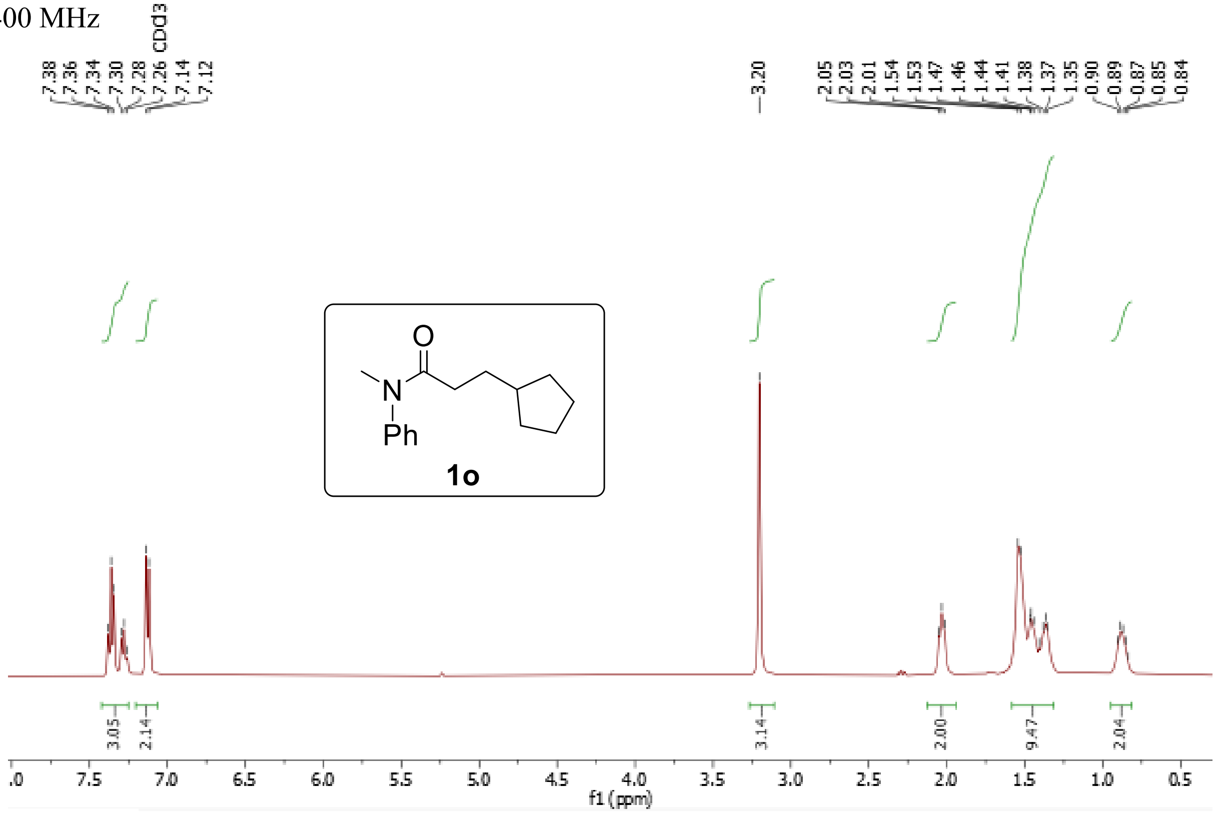

101 MHz

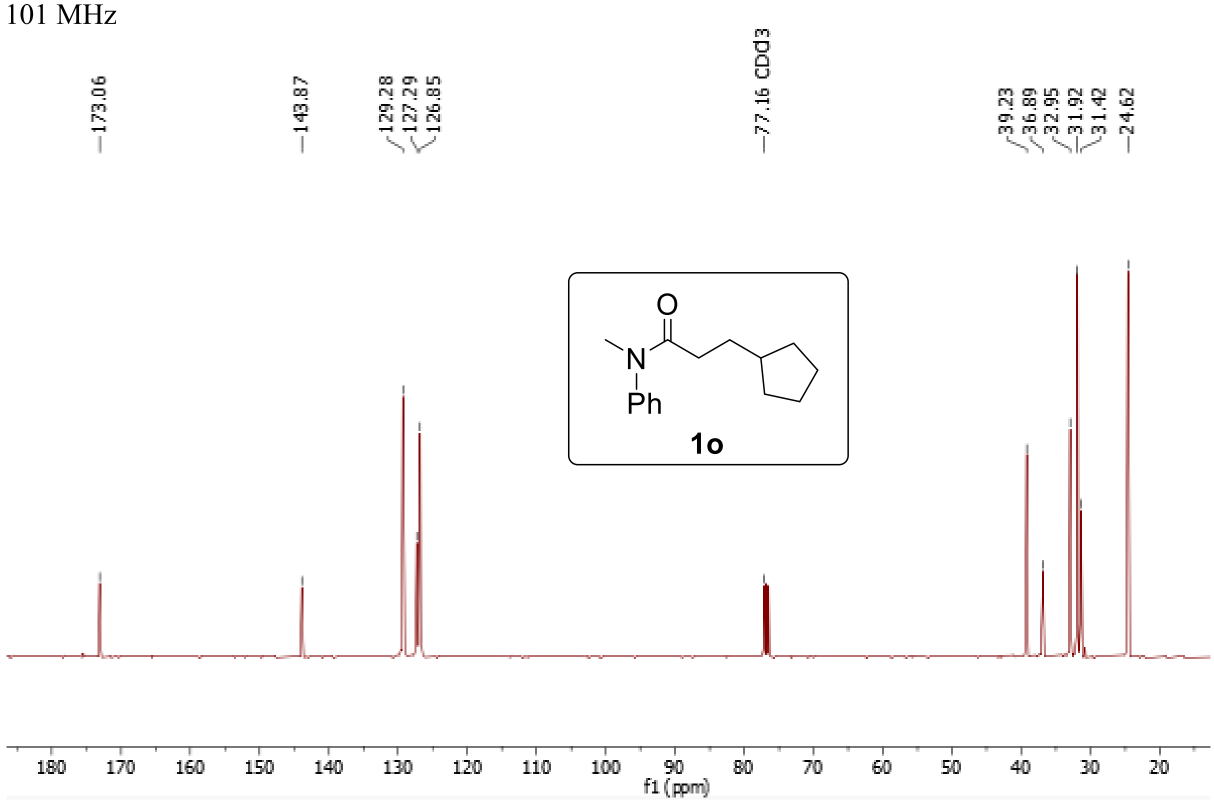

400 MHz

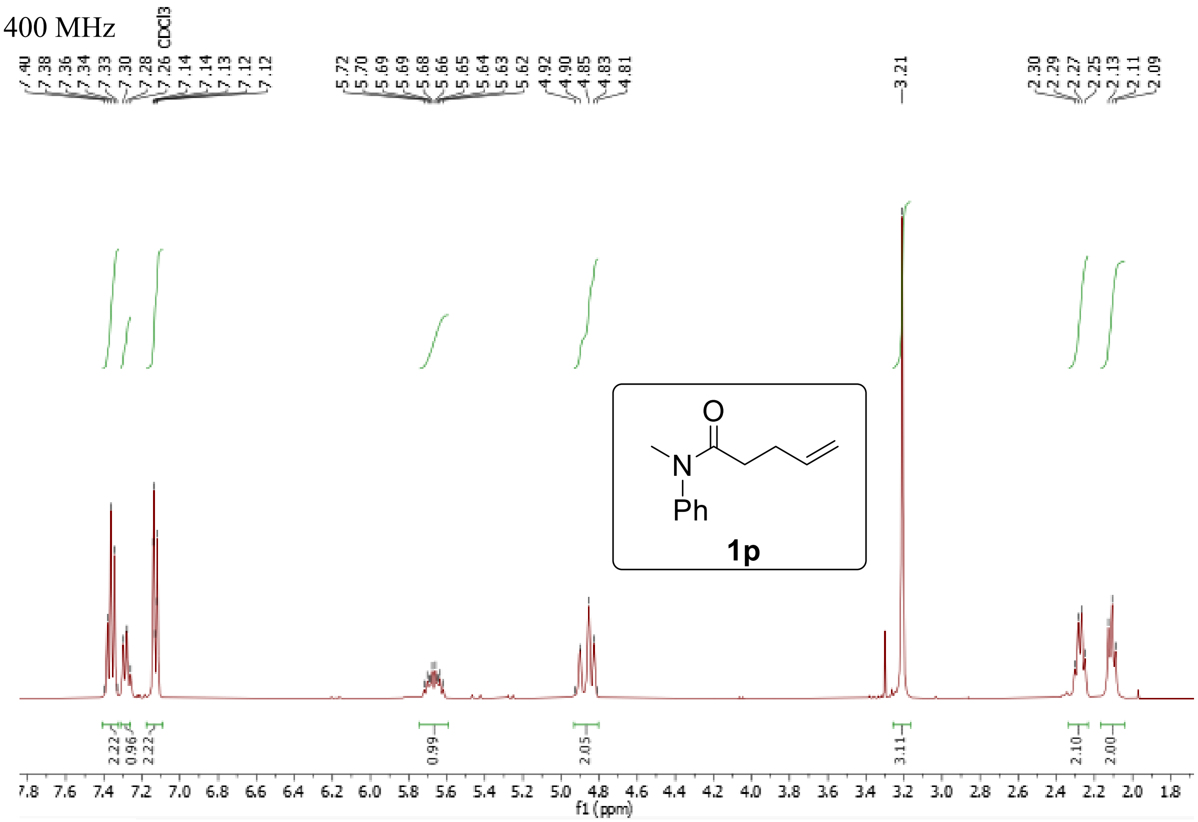

101 MHz

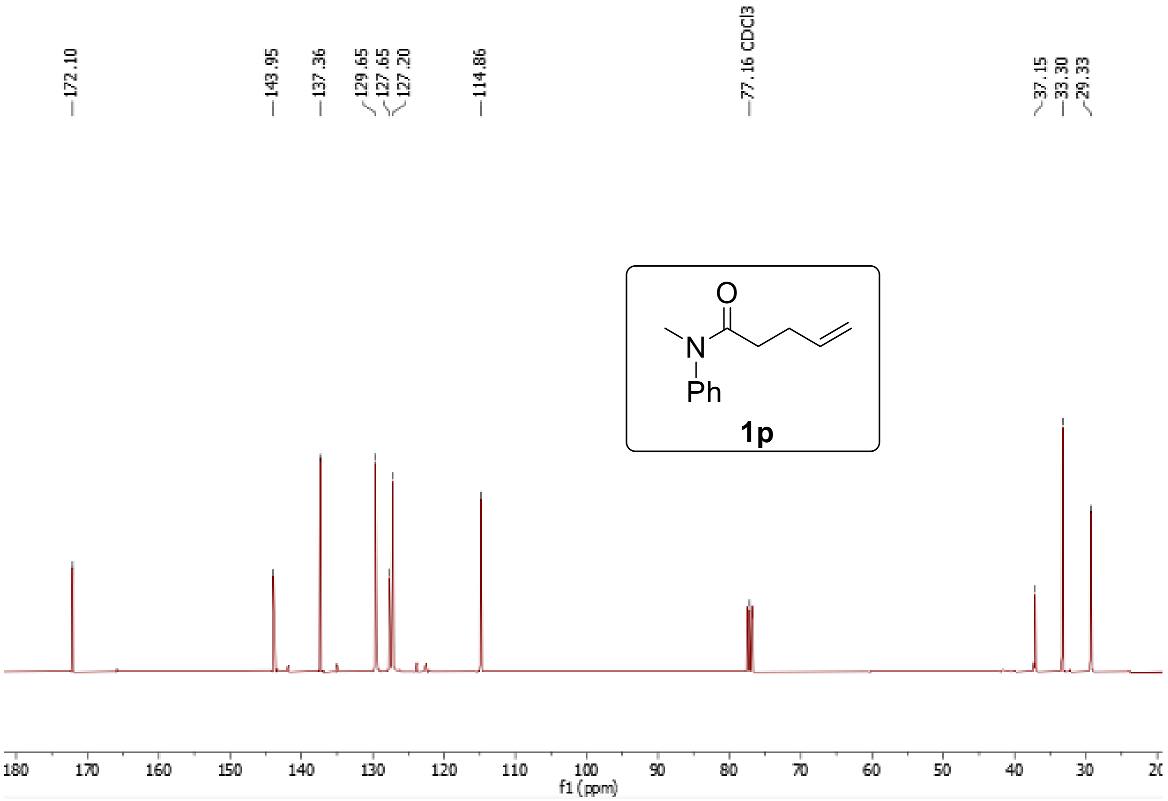

400 MHz

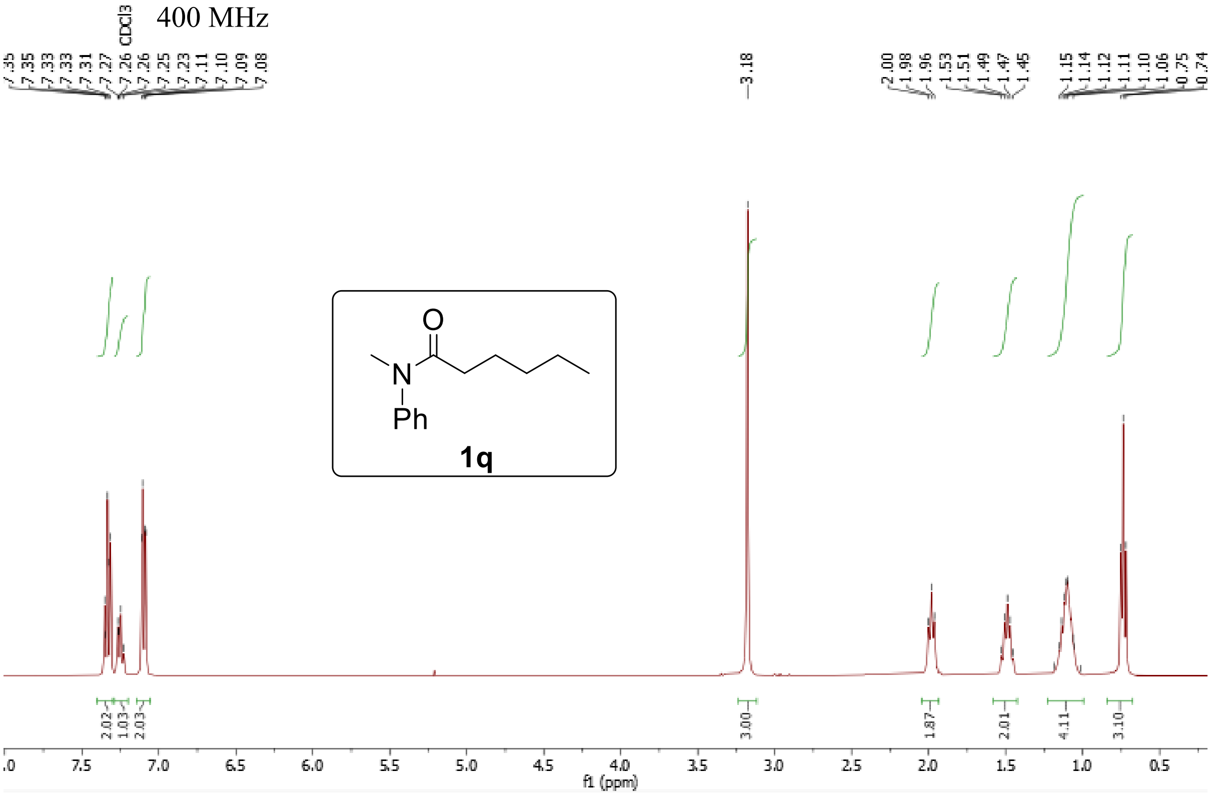

101 MHz

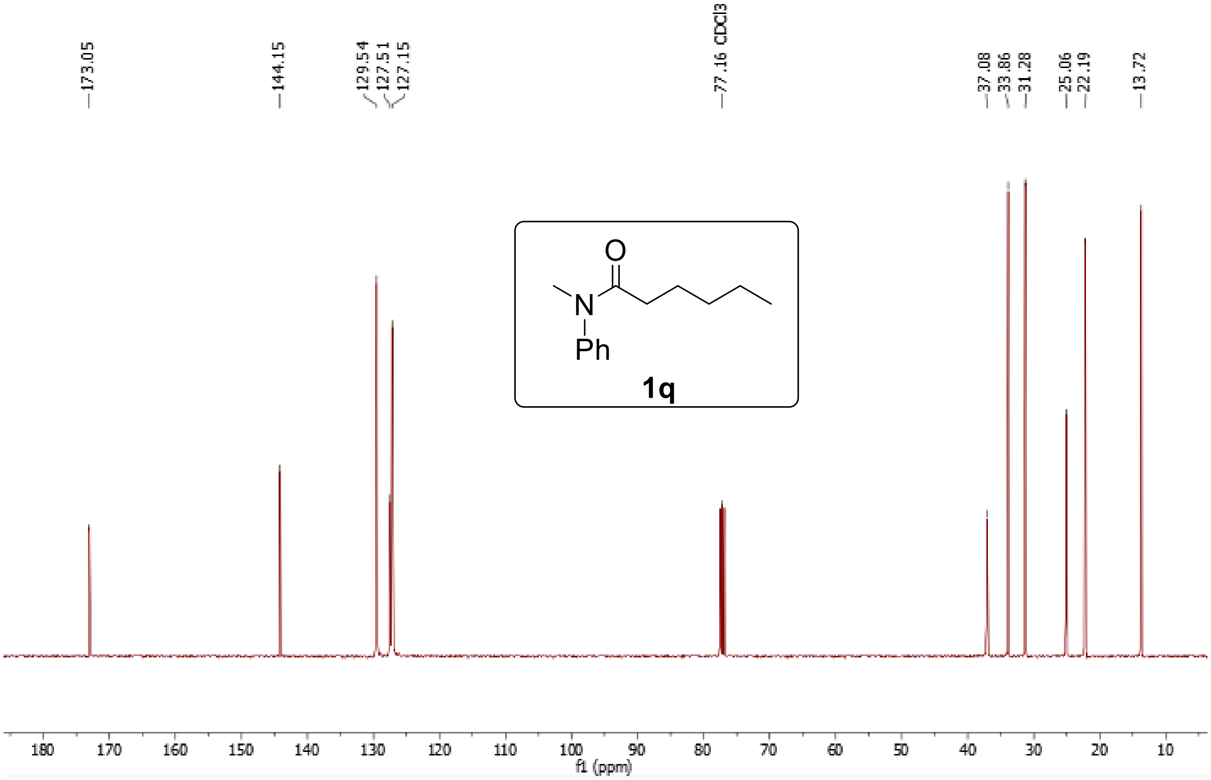

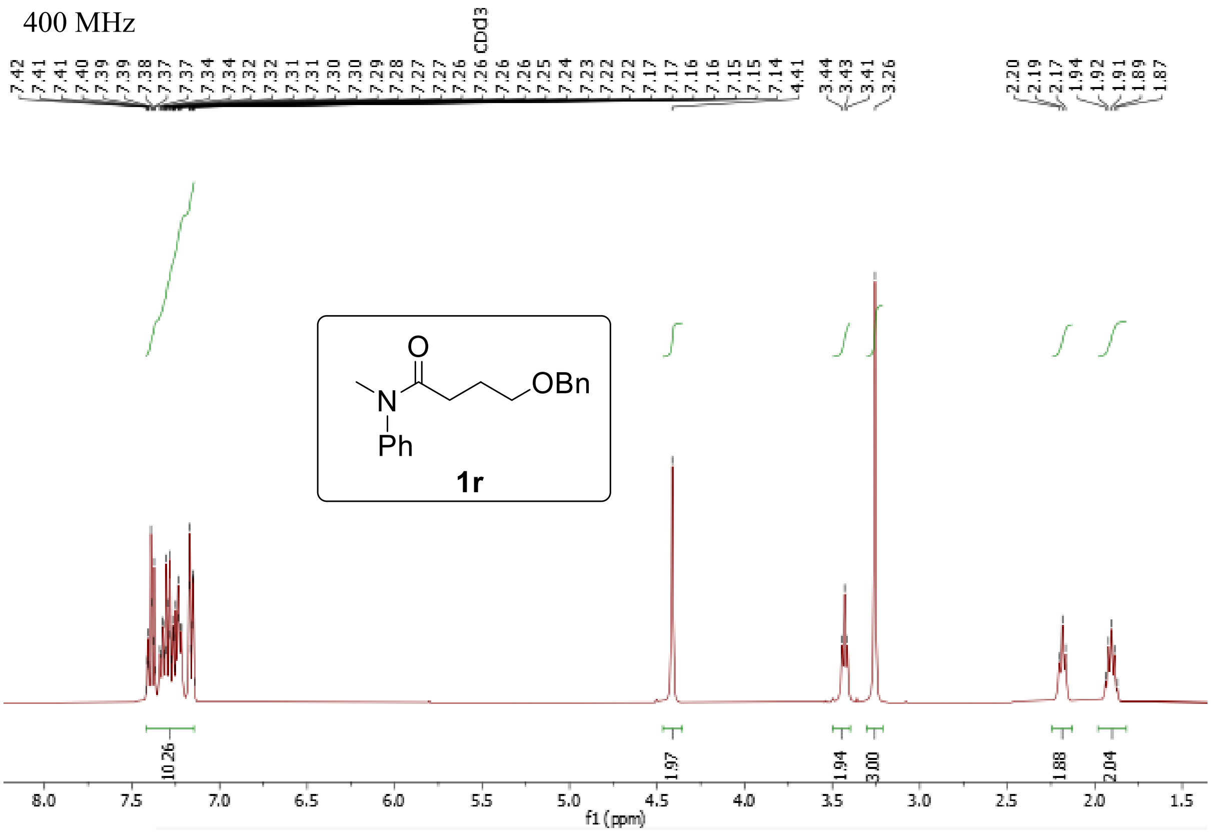
400 MHz
7.42 7.41 7.41 7.40 7.39 7.39 7.38 7.37 7.37 7.37 7.34 7.34 7.32 7.32 7.31 7.31 7.30 7.30 7.29 7.28 7.27 7.27 7.26 7.26 CDCl3 7.26 7.26 7.25 7.24 7.23 7.22 7.22 7.17 7.17 7.16 7.16 7.15 7.15 7.14 4.41 3.44 3.43 3.41 3.26 2.20 2.19 2.17 1.94 1.92 1.91 1.89 1.87
OBn
N
Ph
1r
10.26
1.97
1.94
3.00
1.88
2.04
f1 (ppm)

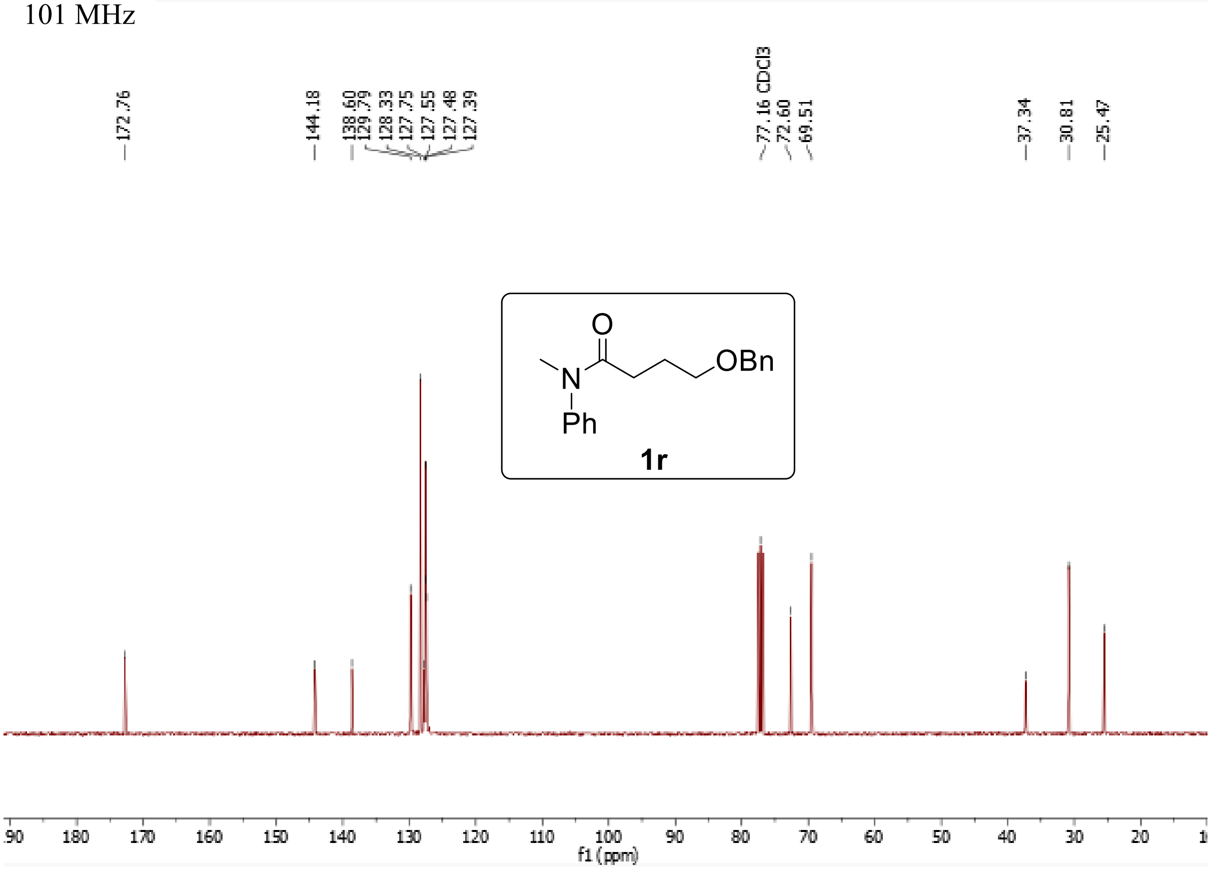
101 MHz
172.76
144.18
138.60 129.79 128.33 127.75 127.55 127.48 127.39
77.16 CDCl3 72.60 69.51
37.34
30.81
25.47
OBn
N
Ph
1r
f1 (ppm)

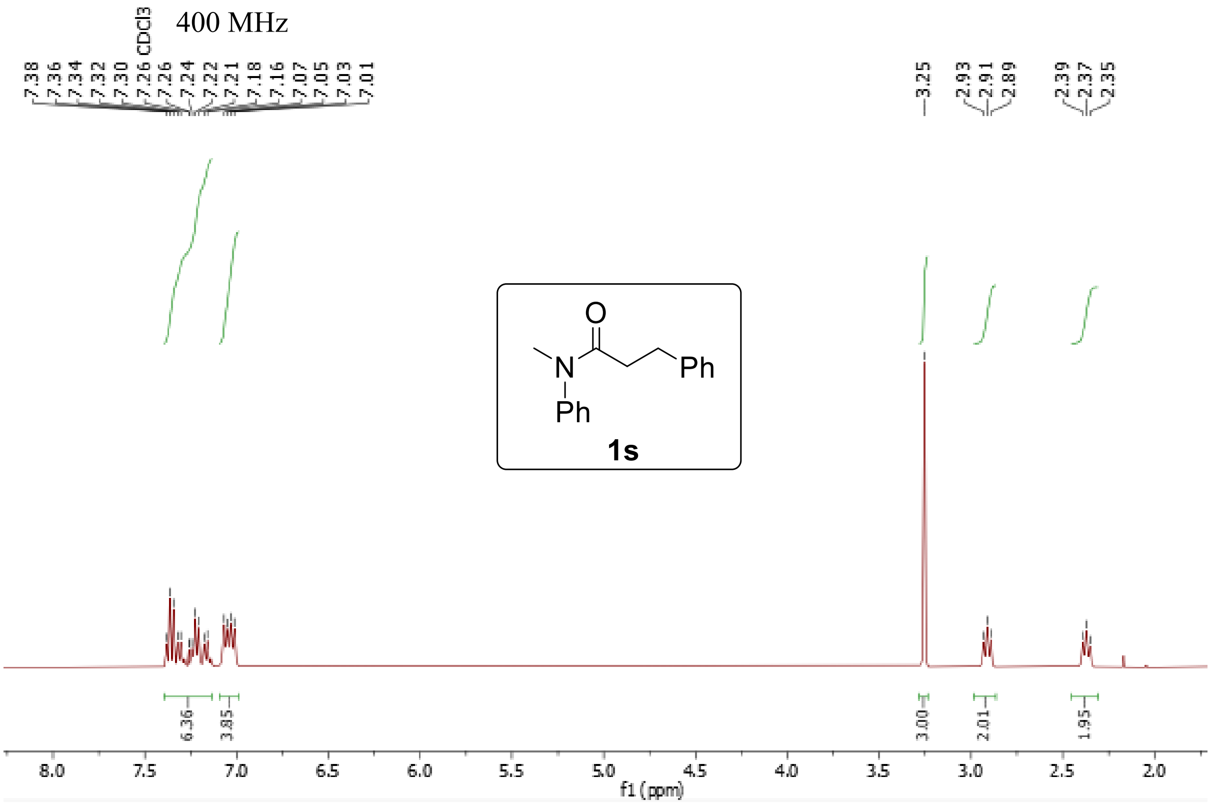
400 MHz
7.38
7.36
7.34
7.32
7.30
7.26 CDCl3
7.26
7.24
7.22
7.21
7.18
7.16
7.07
7.05
7.03
7.01
3.25
2.93
2.91
2.89
2.39
2.37
2.35
O
N
Ph
Ph
1s
6.36
3.85
3.00
2.01
1.95
8.0
7.5
7.0
6.5
6.0
5.5
5.0
4.5
4.0
3.5
3.0
2.5
2.0
f1 (ppm)

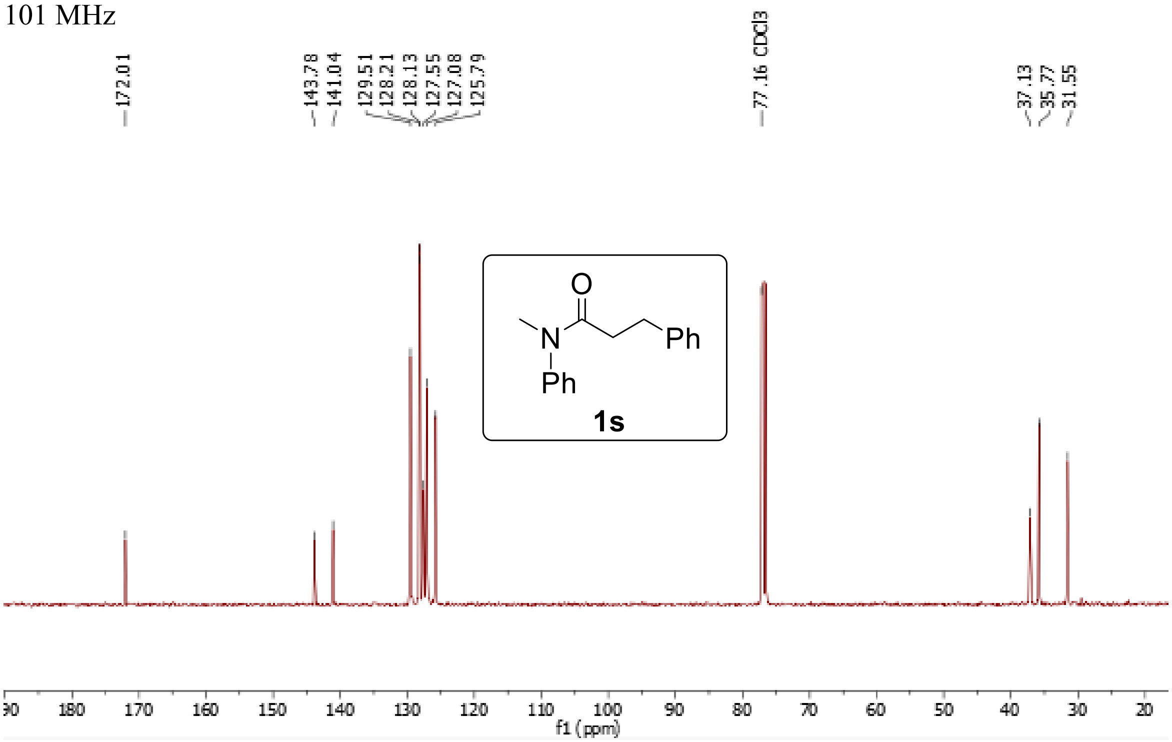
101 MHz
172.01
143.78
141.04
129.51
128.21
128.13
127.55
127.08
125.79
77.16 CDCl3
37.13
35.77
31.55
O
N
Ph
Ph
1s
180
170
160
150
140
130
120
110
100
90
80
70
60
50
40
30
20
f1 (ppm)

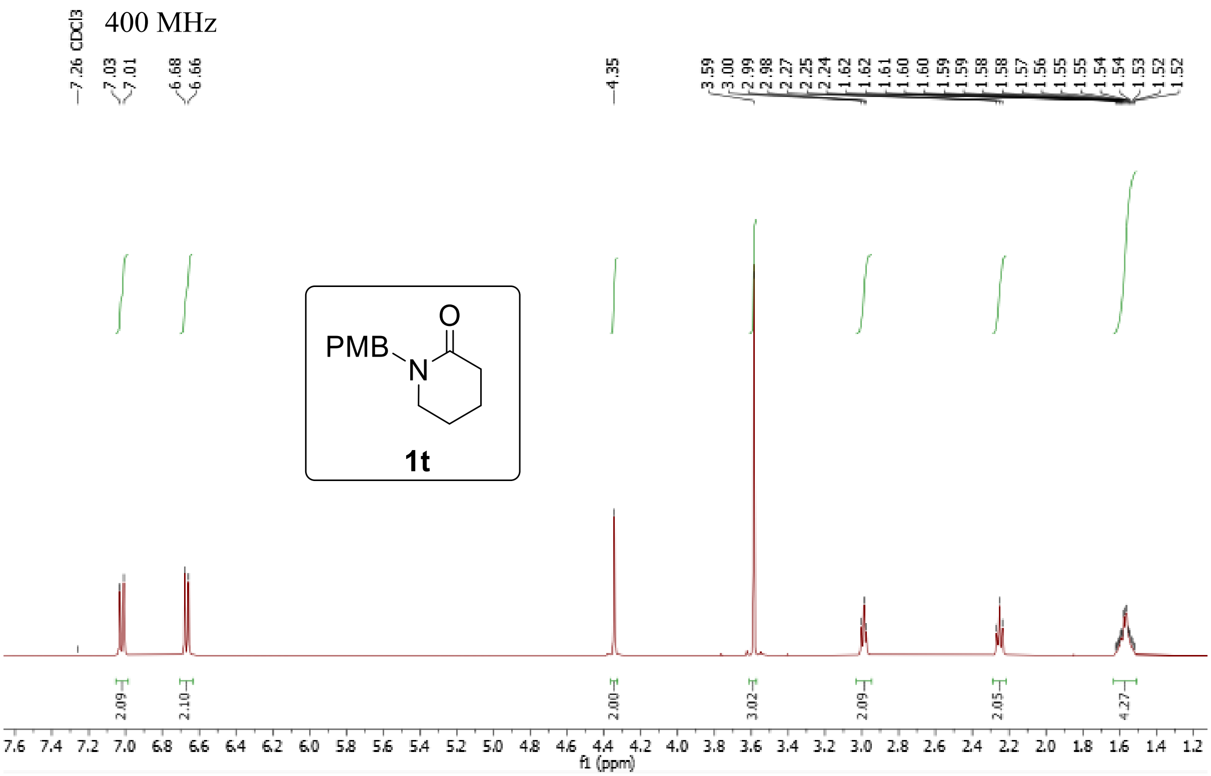
400 MHz
7.26 CDCl3
7.03
7.01
6.68
6.66
4.35
3.59
3.00
2.99
2.98
2.27
2.25
2.24
1.62
1.61
1.60
1.59
1.59
1.58
1.58
1.57
1.56
1.55
1.55
1.54
1.54
1.53
1.52
1.52
PMB
N
O
1t
2.09
2.10
2.00
3.02
2.09
2.05
4.27
f1 (ppm)

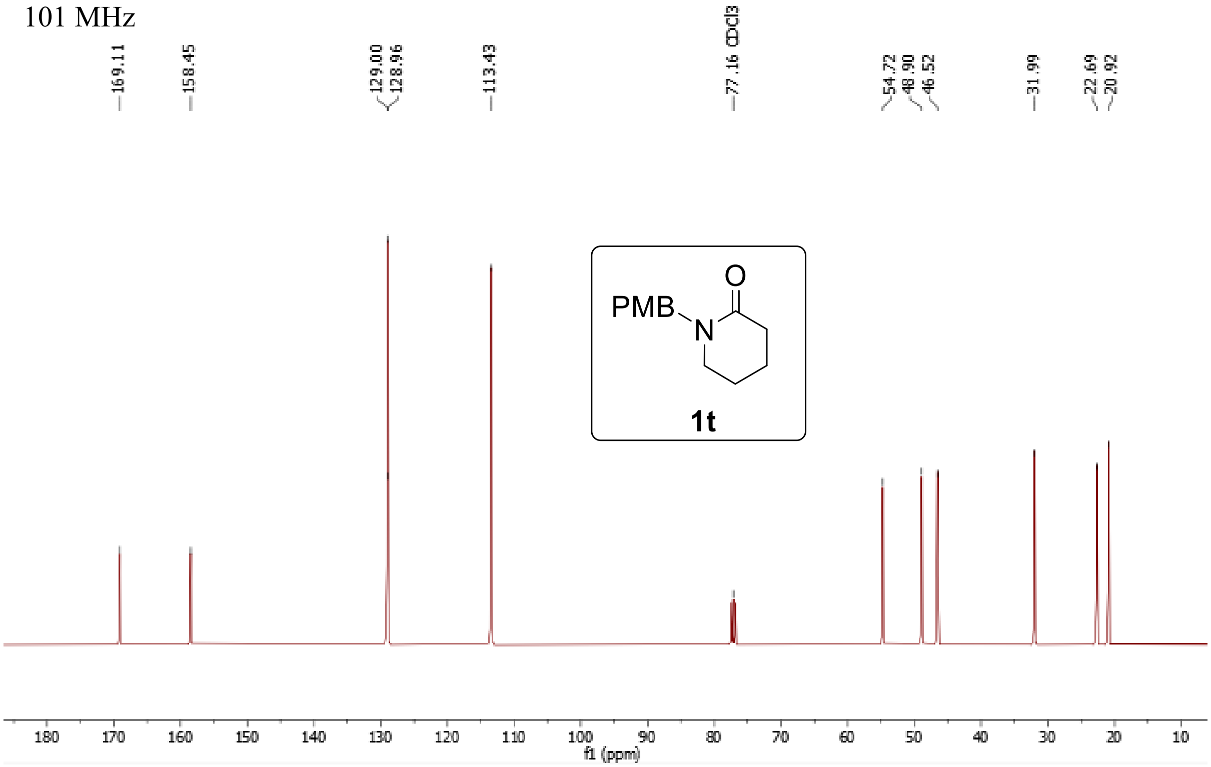
101 MHz
169.11
158.45
129.00
128.96
113.43
77.16 CDCl3
54.72
48.90
46.52
31.99
22.69
20.92
PMB
N
O
1t
f1 (ppm)

300 MHz

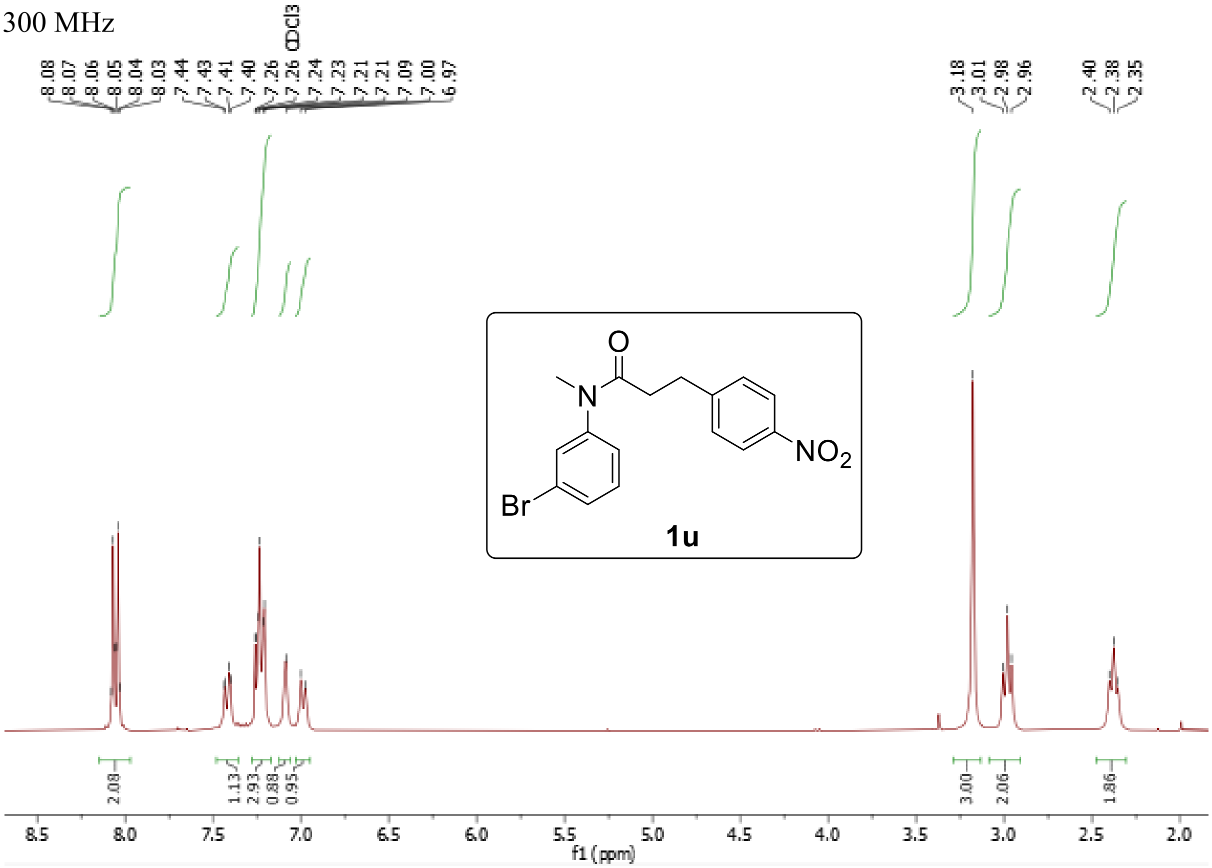

151 MHz

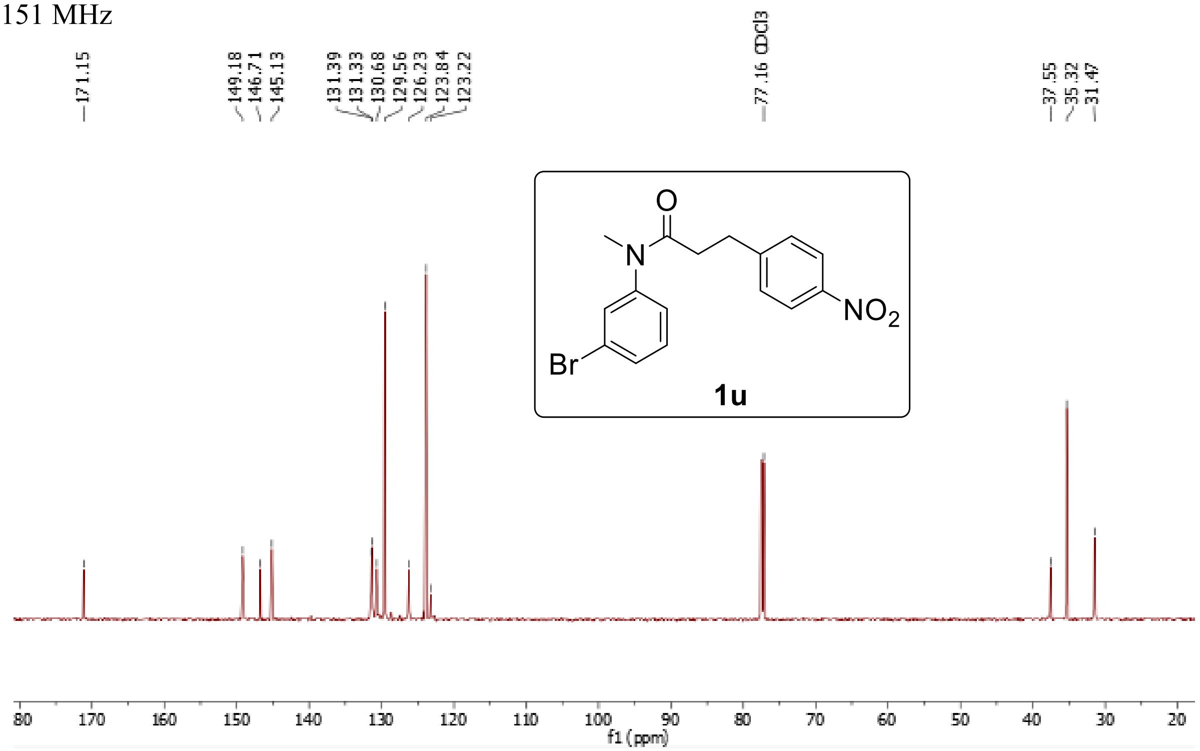

(ii) 2a-u

300 MHz

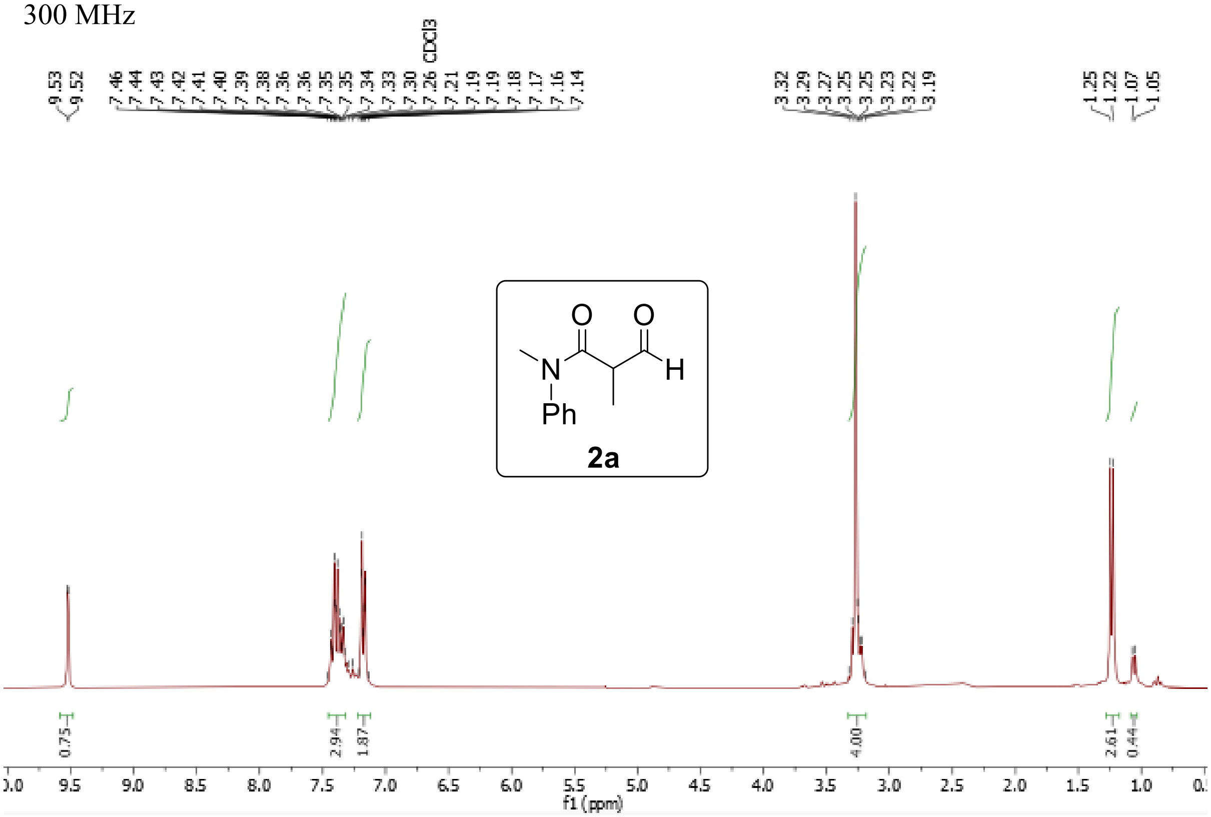

101 MHz

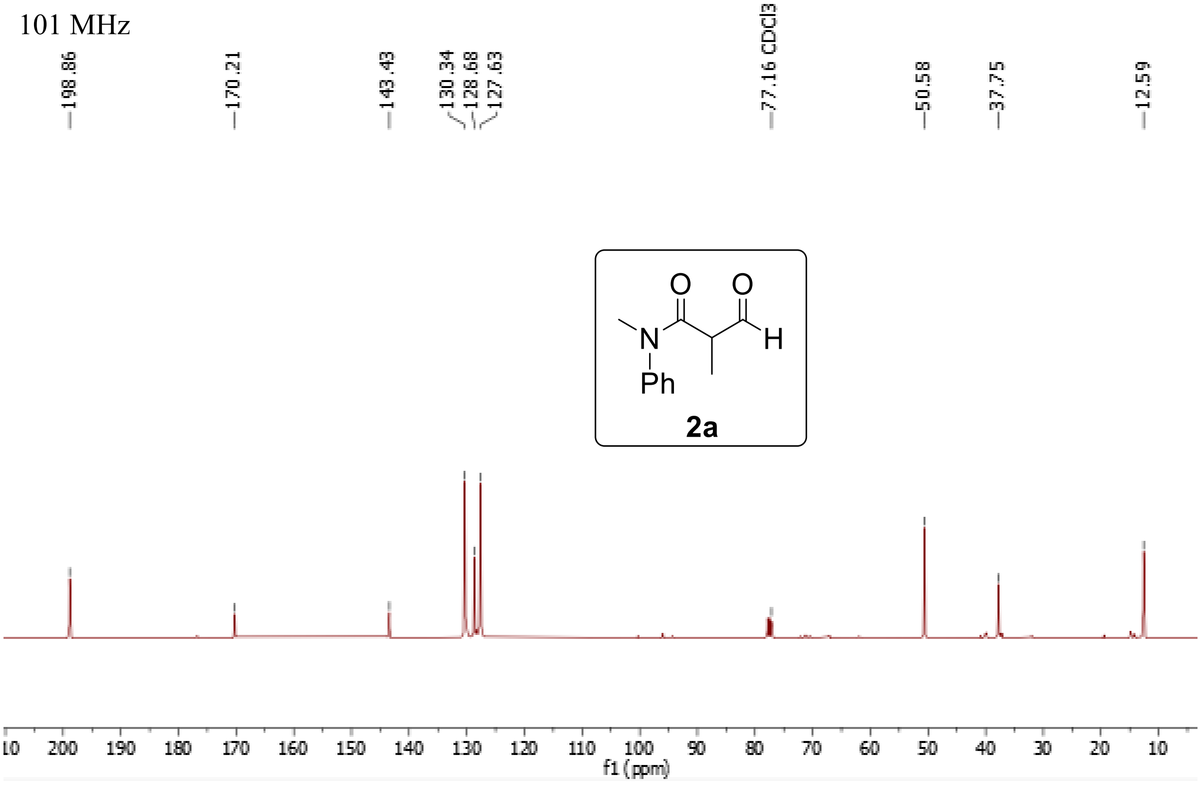

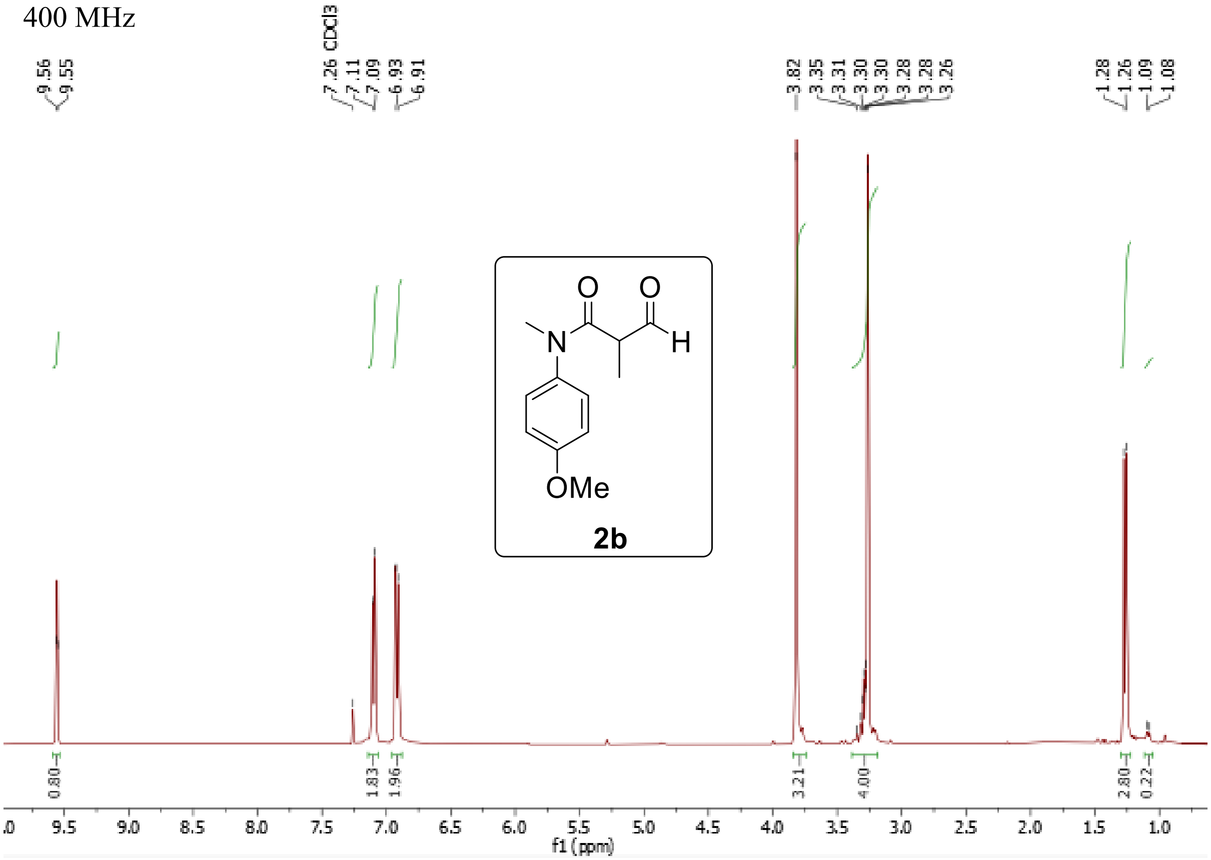
400 MHz
9.56
9.55
7.26 CDCl3
7.11
7.09
6.93
6.91
3.82
3.35
3.31
3.30
3.30
3.28
3.28
3.26
1.28
1.26
1.09
1.08
0.80
1.83
1.96
3.21
4.00
2.80
0.22
O
O
N
H
OMe
2b
f1 (ppm)

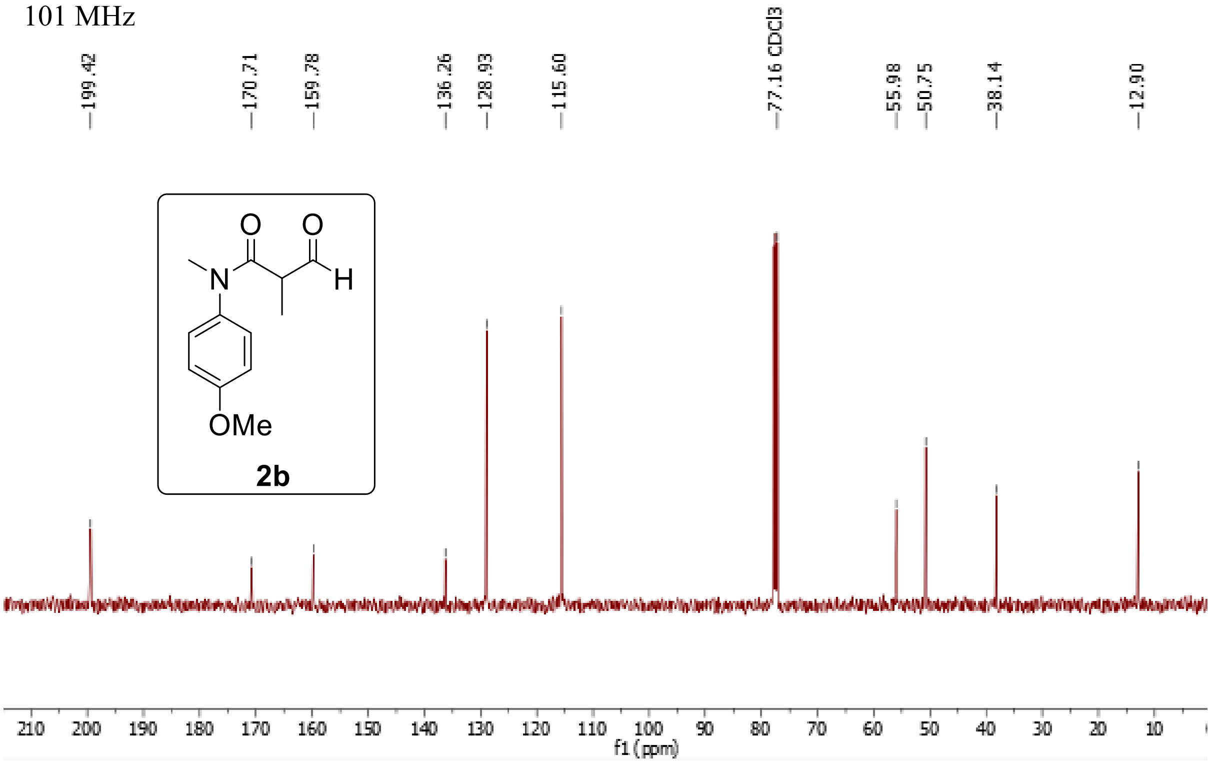
101 MHz
199.42
170.71
159.78
136.26
128.93
115.60
77.16 CDCl3
55.98
50.75
38.14
12.90
O
O
N
H
OMe
2b
f1 (ppm)

300 MHz

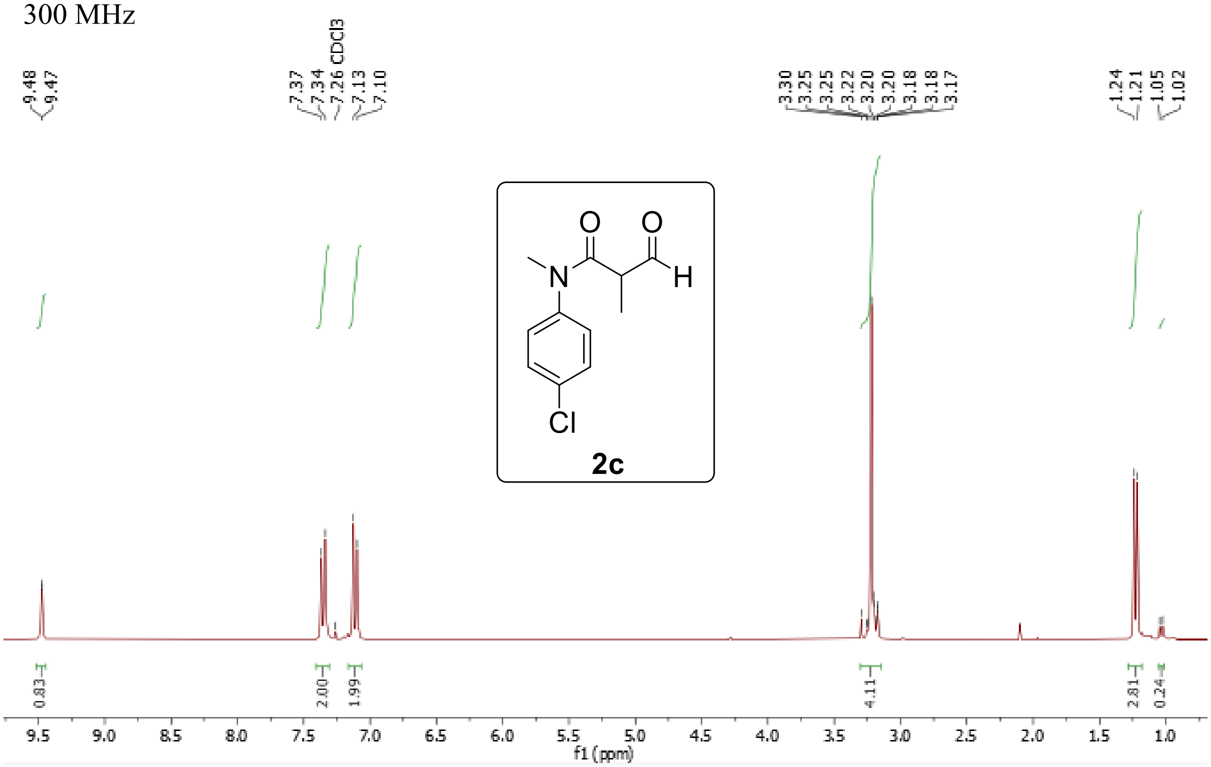

101 MHz

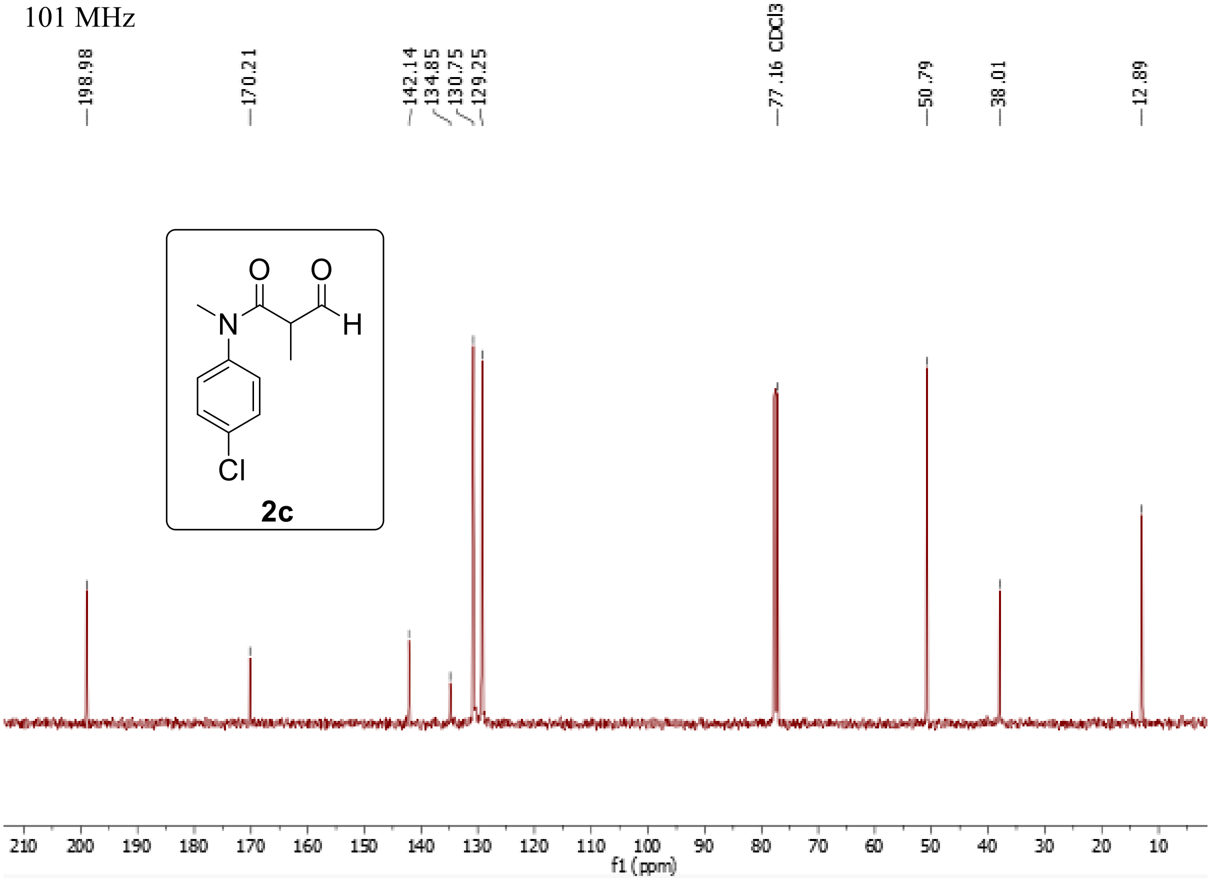

300 MHz

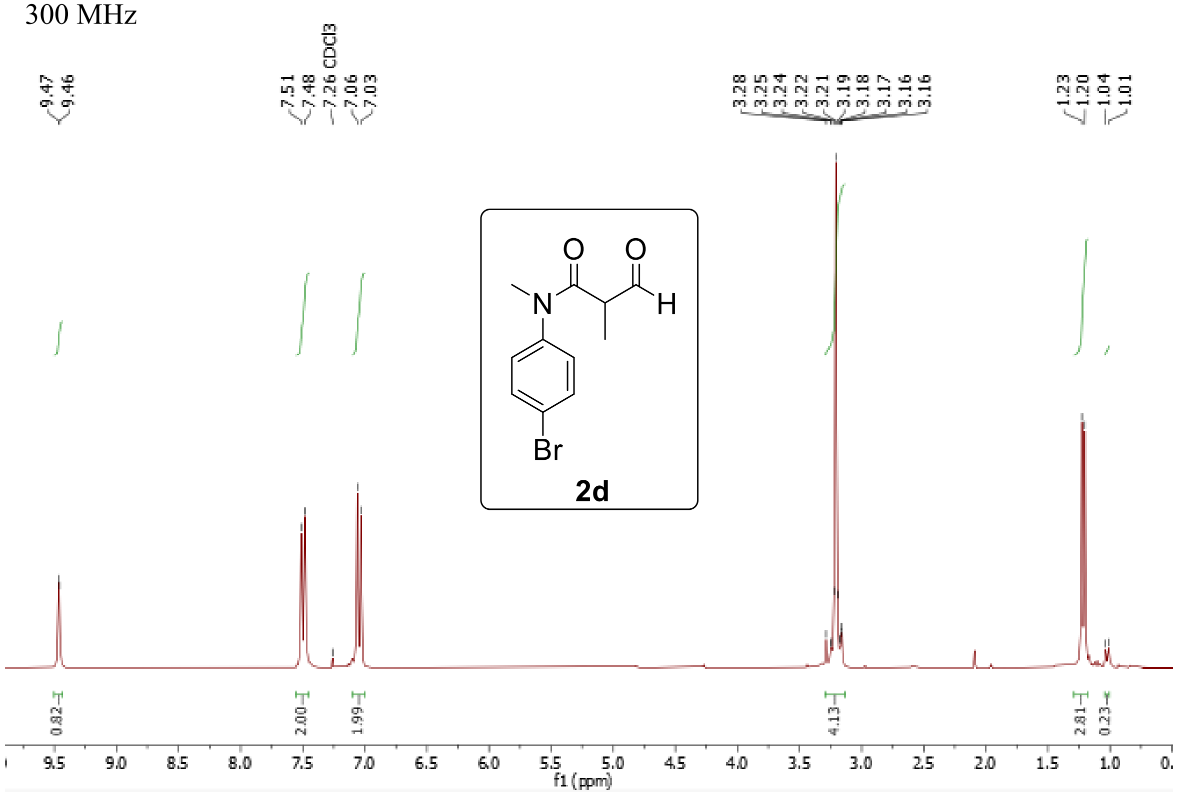

101 MHz

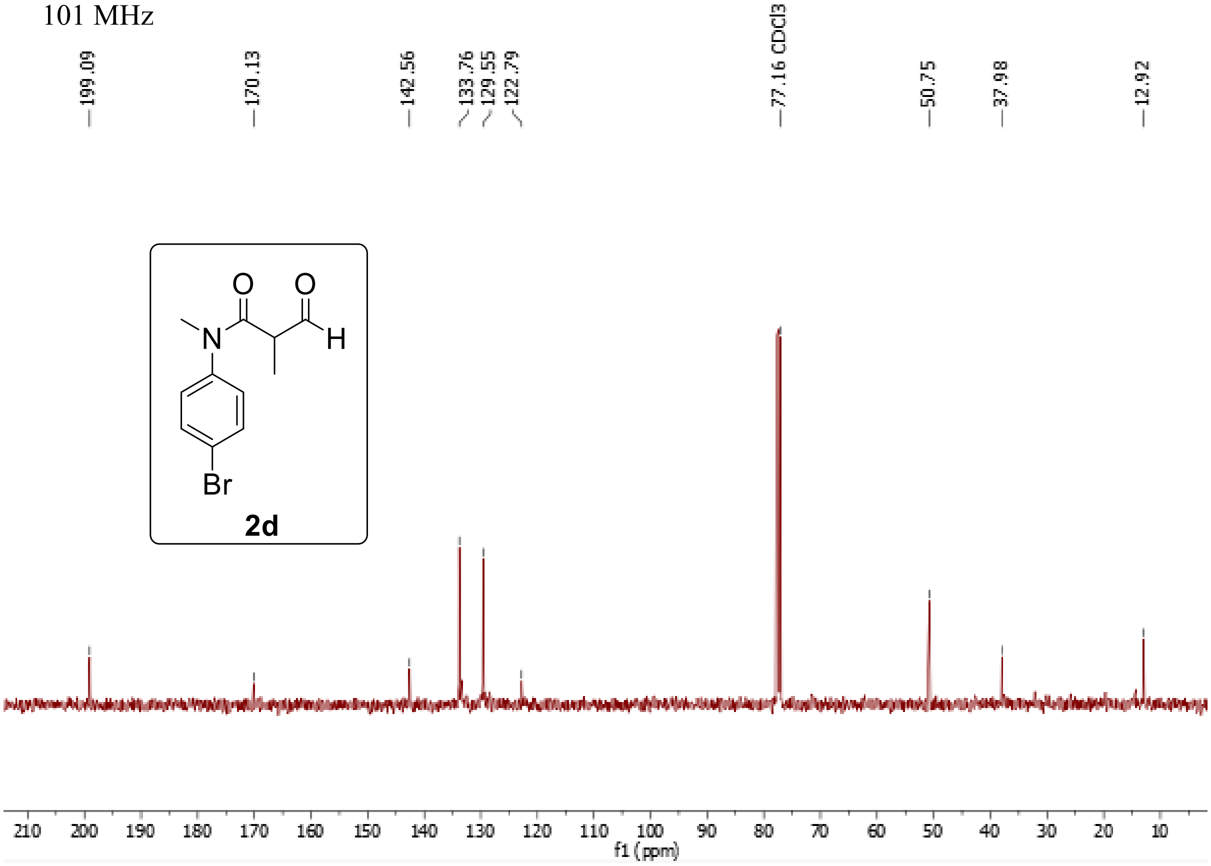

400 MHz

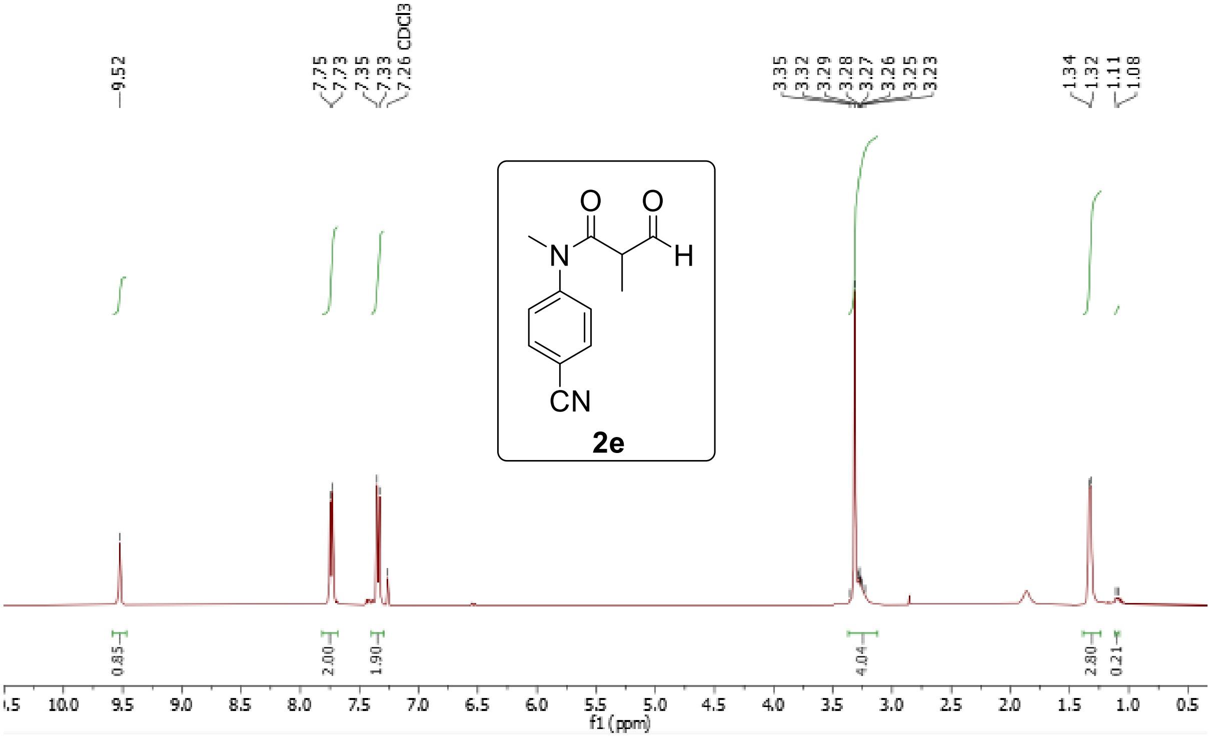

101 MHz

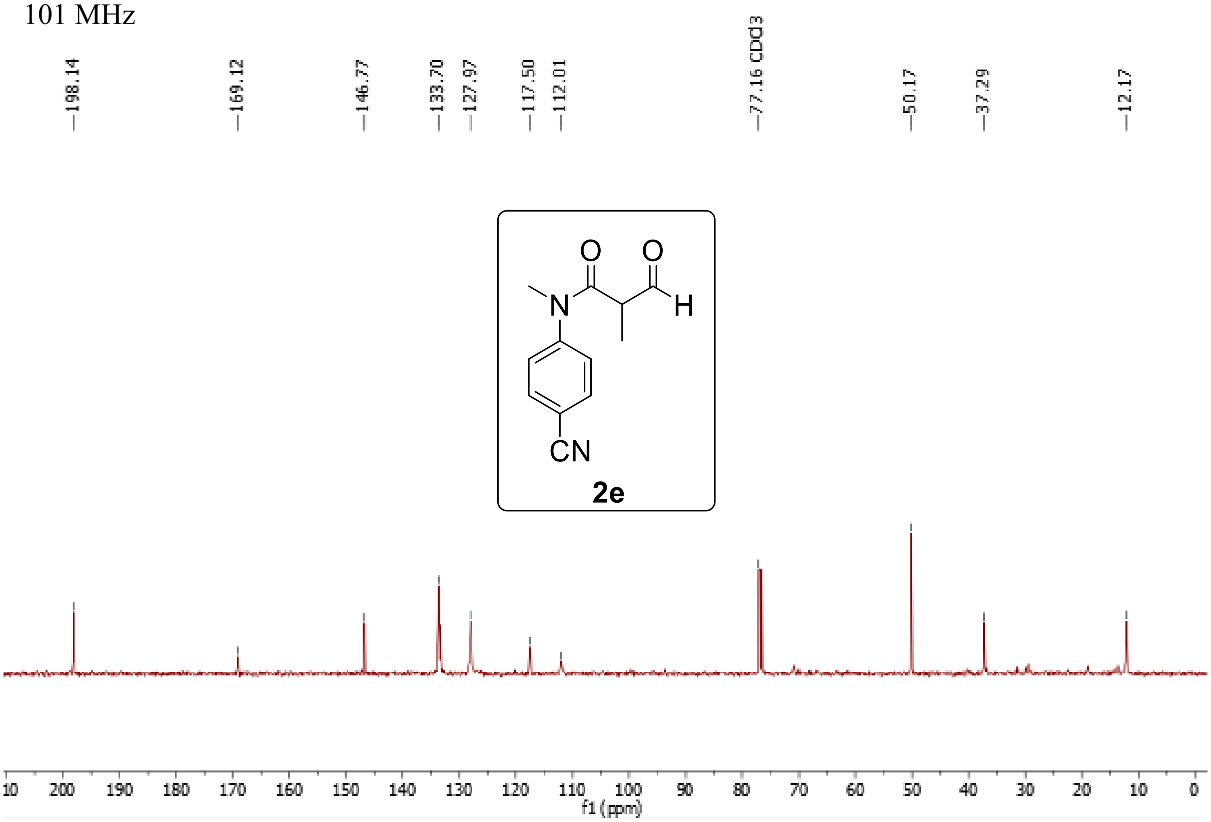

300 MHz

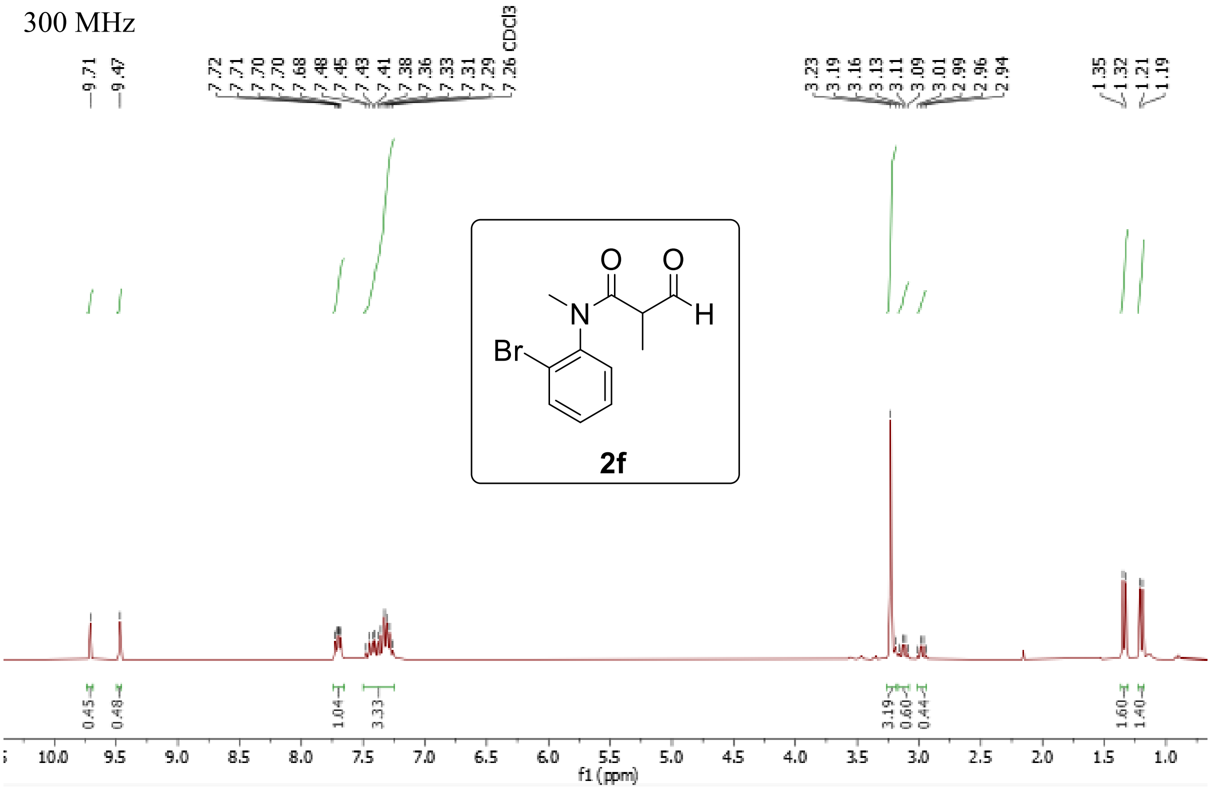

75 MHz

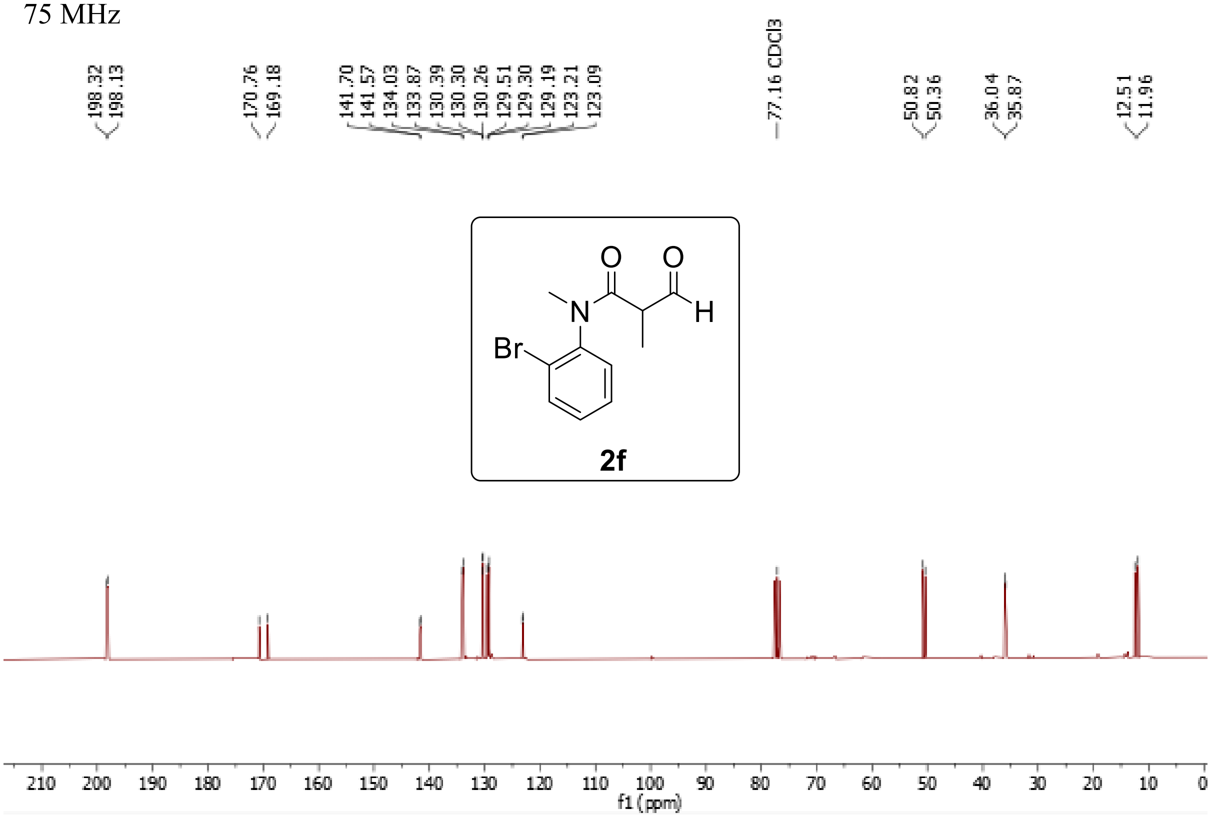

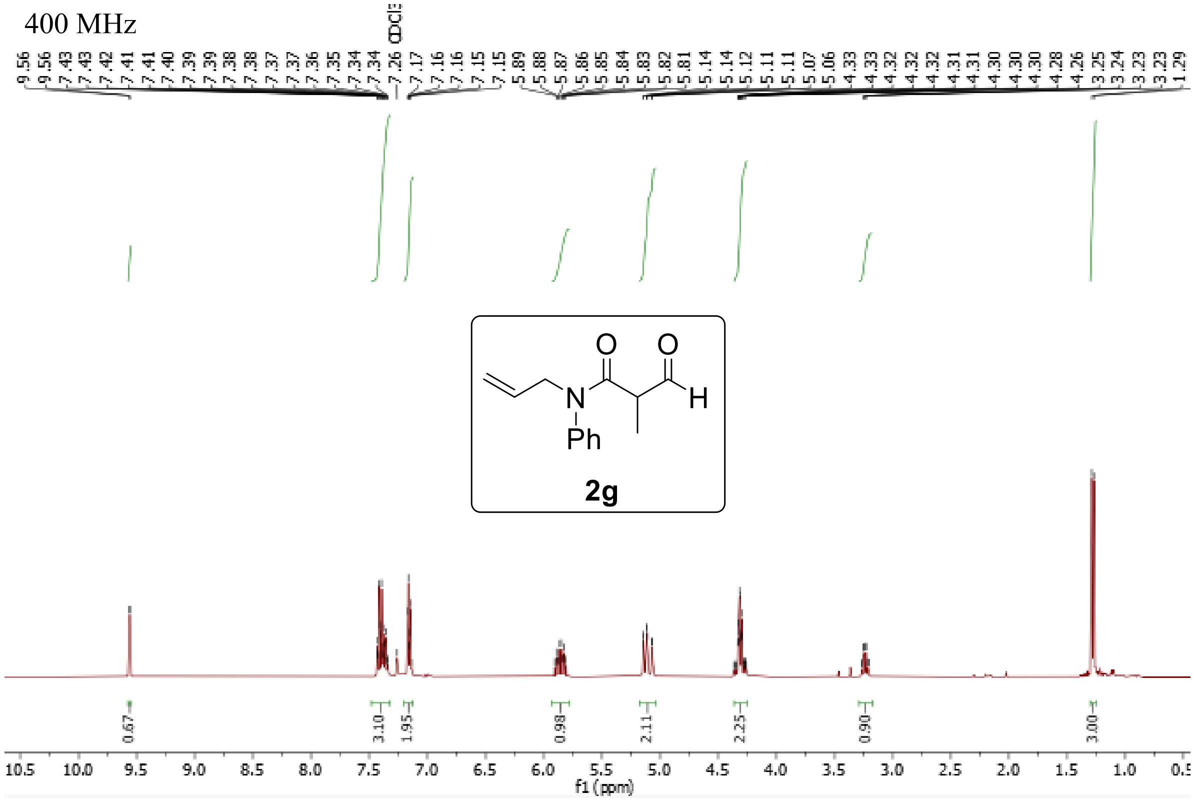
400 MHz
9.56 9.56 7.43 7.43 7.42 7.41 7.41 7.40 7.39 7.39 7.38 7.38 7.37 7.37 7.36 7.35 7.34 7.34 CDCl3 7.26 7.17 7.16 7.16 7.15 7.15 5.89 5.88 5.87 5.86 5.85 5.84 5.83 5.82 5.81 5.14 5.14 5.12 5.11 5.11 5.07 5.06 4.33 4.33 4.32 4.32 4.32 4.31 4.31 4.30 4.30 4.30 4.28 4.26 3.25 3.24 3.23 3.23 1.29
N
O
O
H
Ph
2g
0.67 3.10 1.95 0.98 2.11 2.25 0.90 3.00
10.5 10.0 9.5 9.0 8.5 8.0 7.5 7.0 6.5 6.0 5.5 5.0 4.5 4.0 3.5 3.0 2.5 2.0 1.5 1.0 0.5
f1 (ppm)

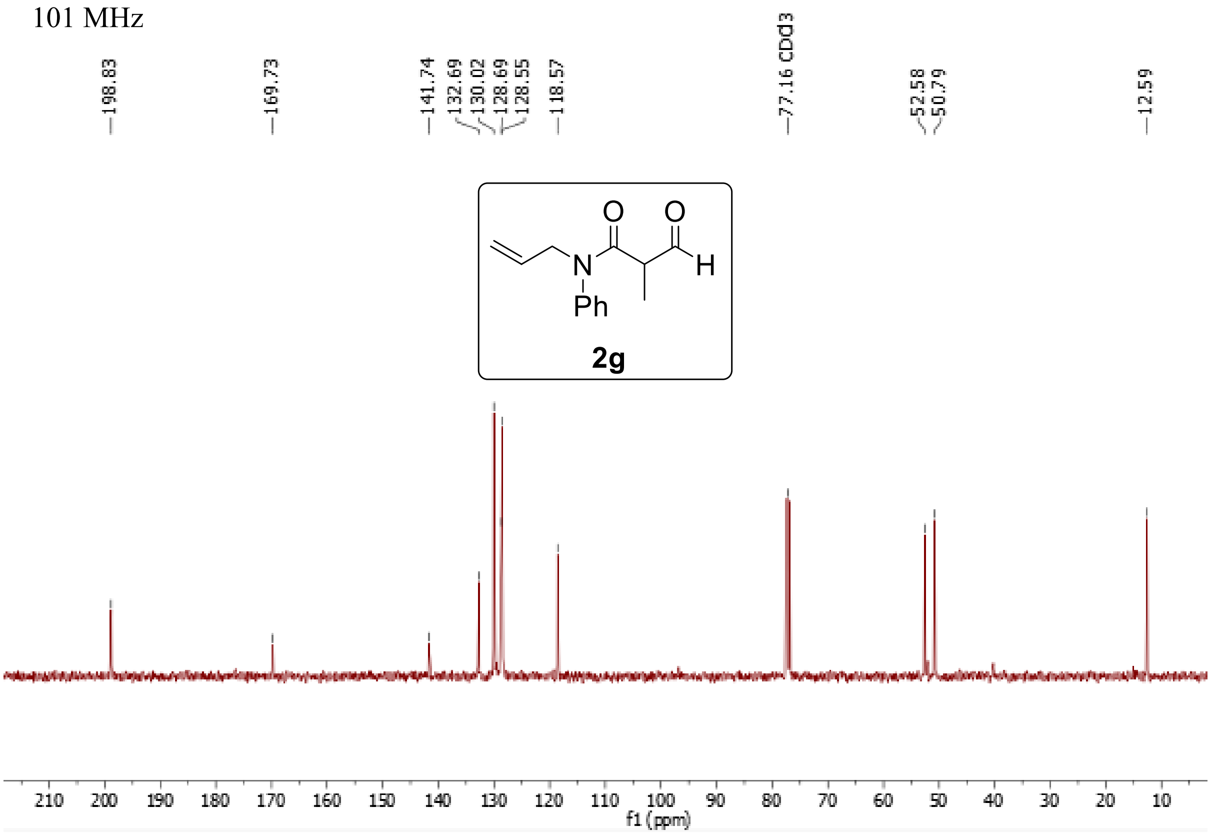
101 MHz
198.83 169.73 141.74 132.69 130.02 128.69 128.55 118.57 77.16 CDCl3 52.58 50.79 12.59
N
O
O
H
Ph
2g
210 200 190 180 170 160 150 140 130 120 110 100 90 80 70 60 50 40 30 20 10
f1 (ppm)

400 MHz

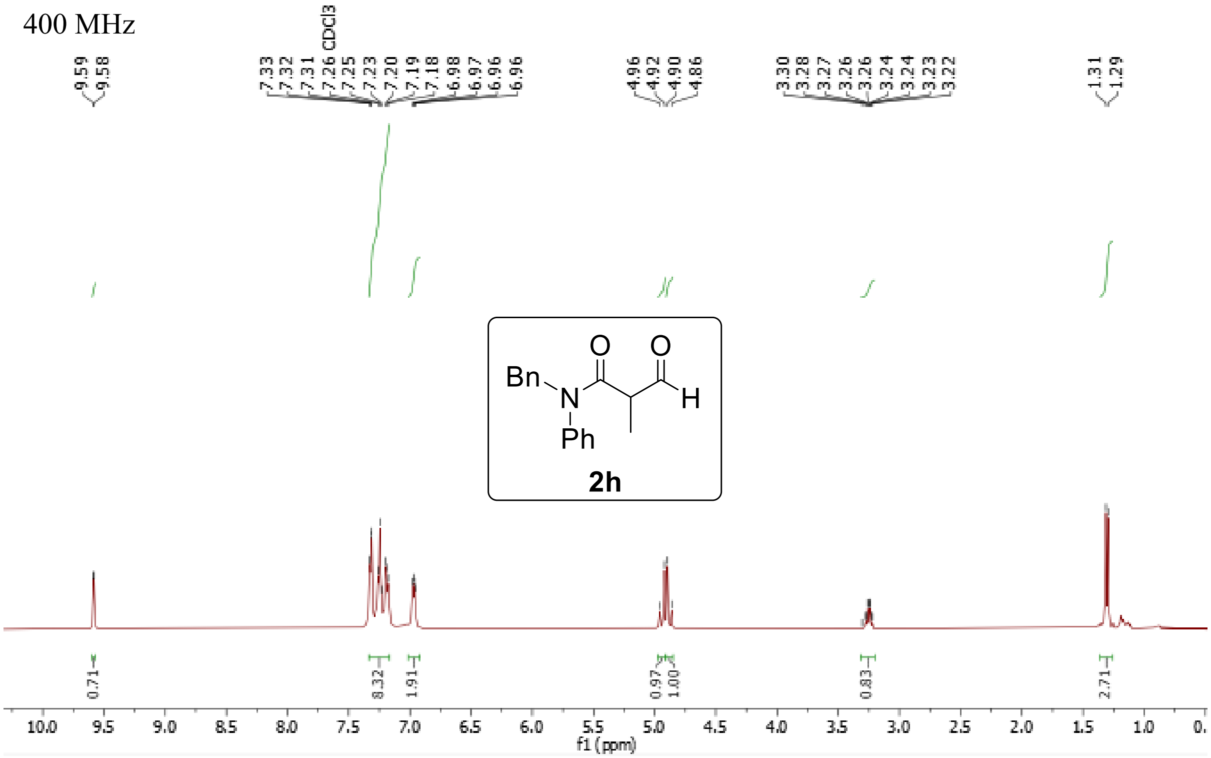

101 MHz

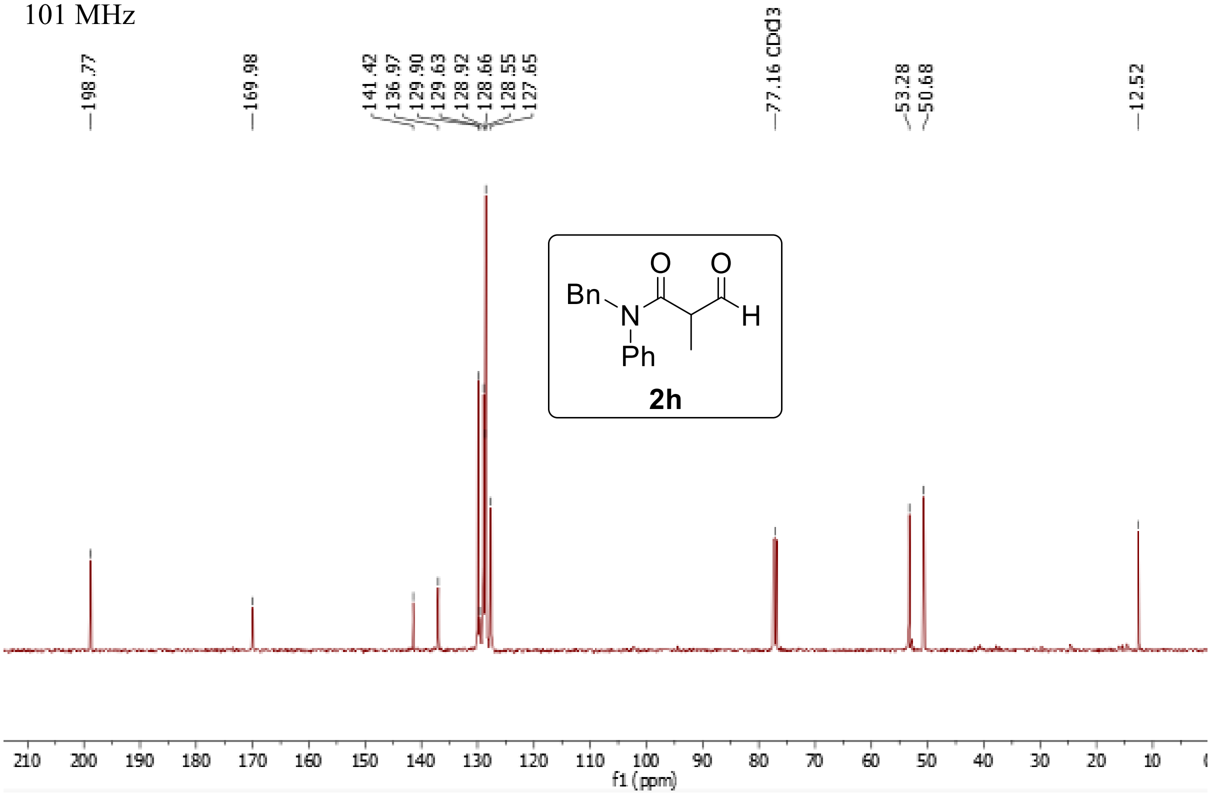

300 MHz

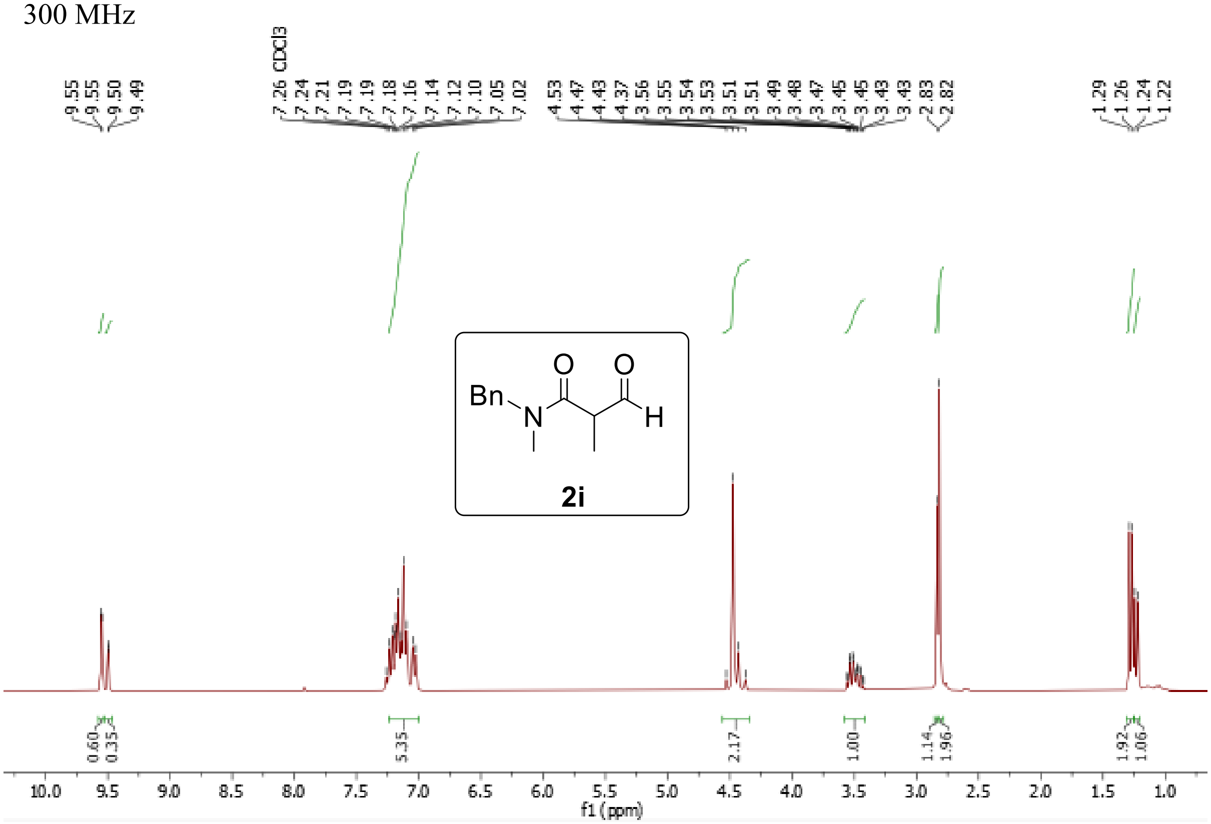

101 MHz

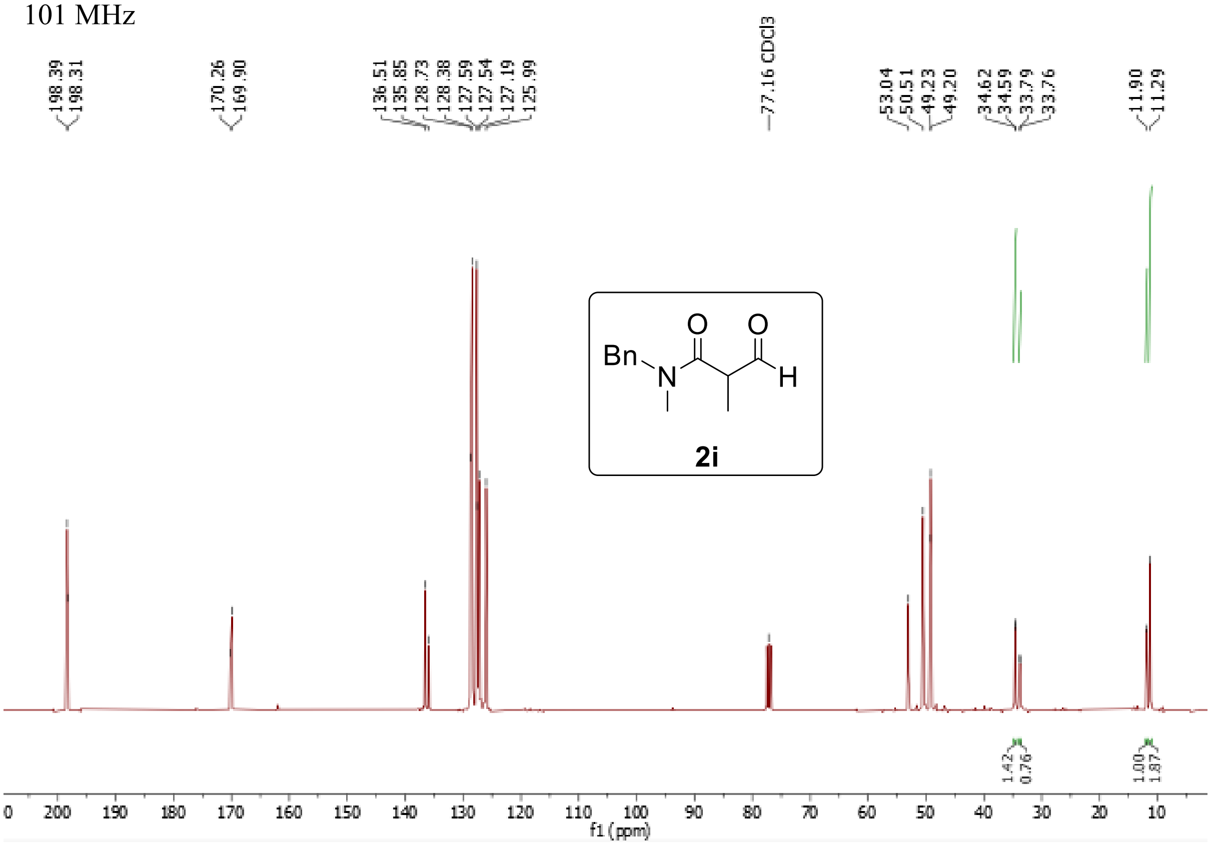

400 MHz

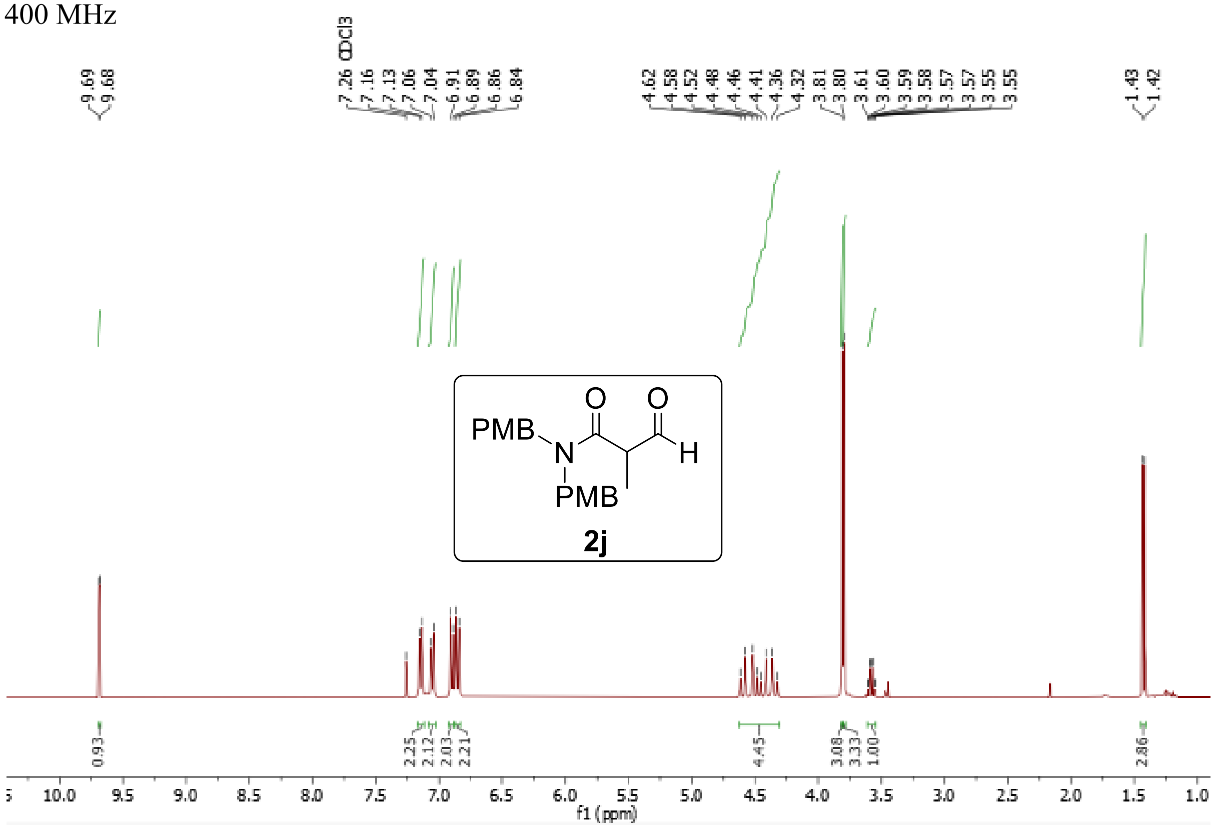

101 MHz

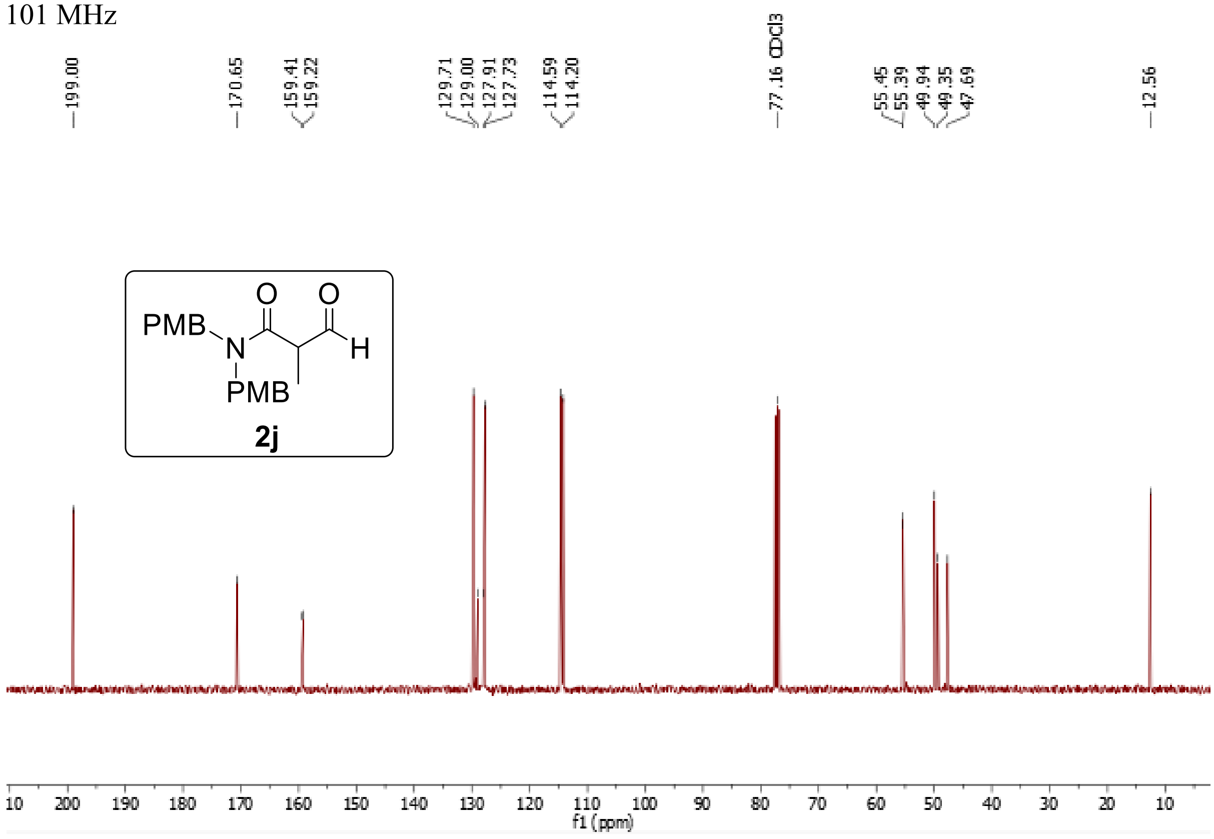

300 MHz

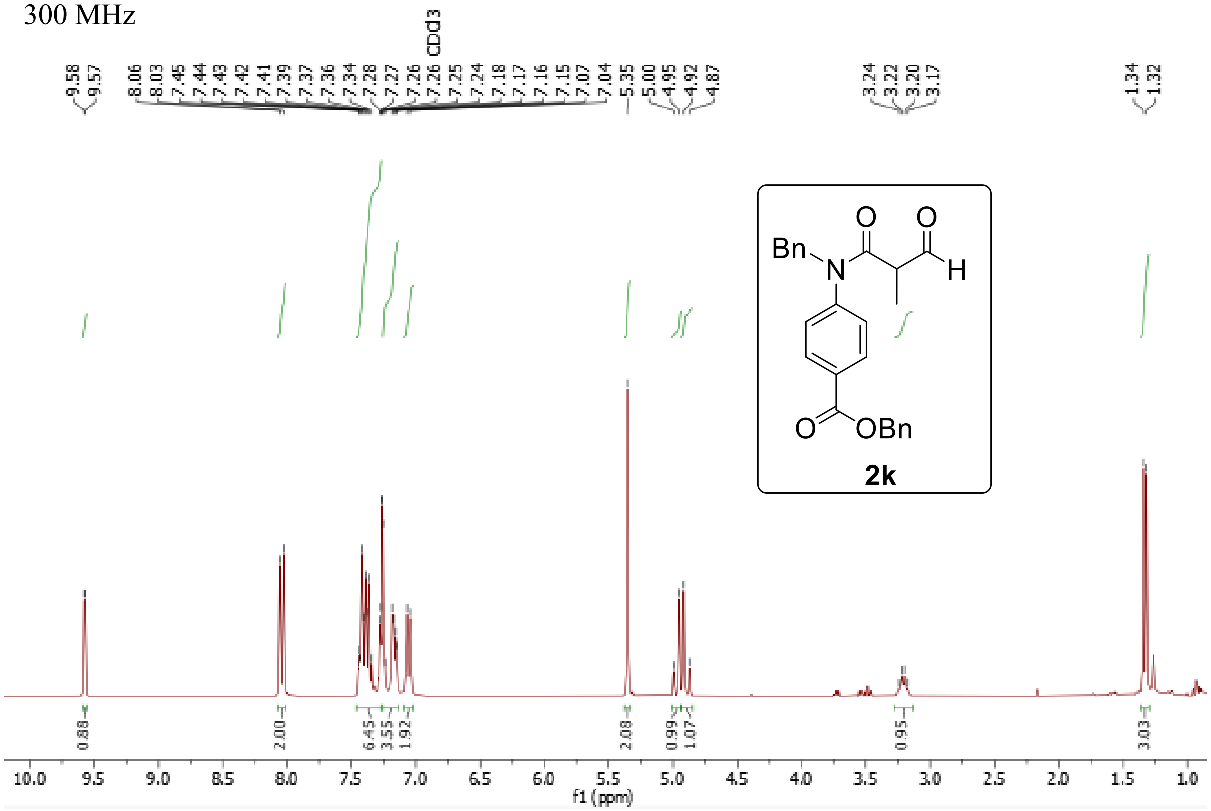

151 MHz

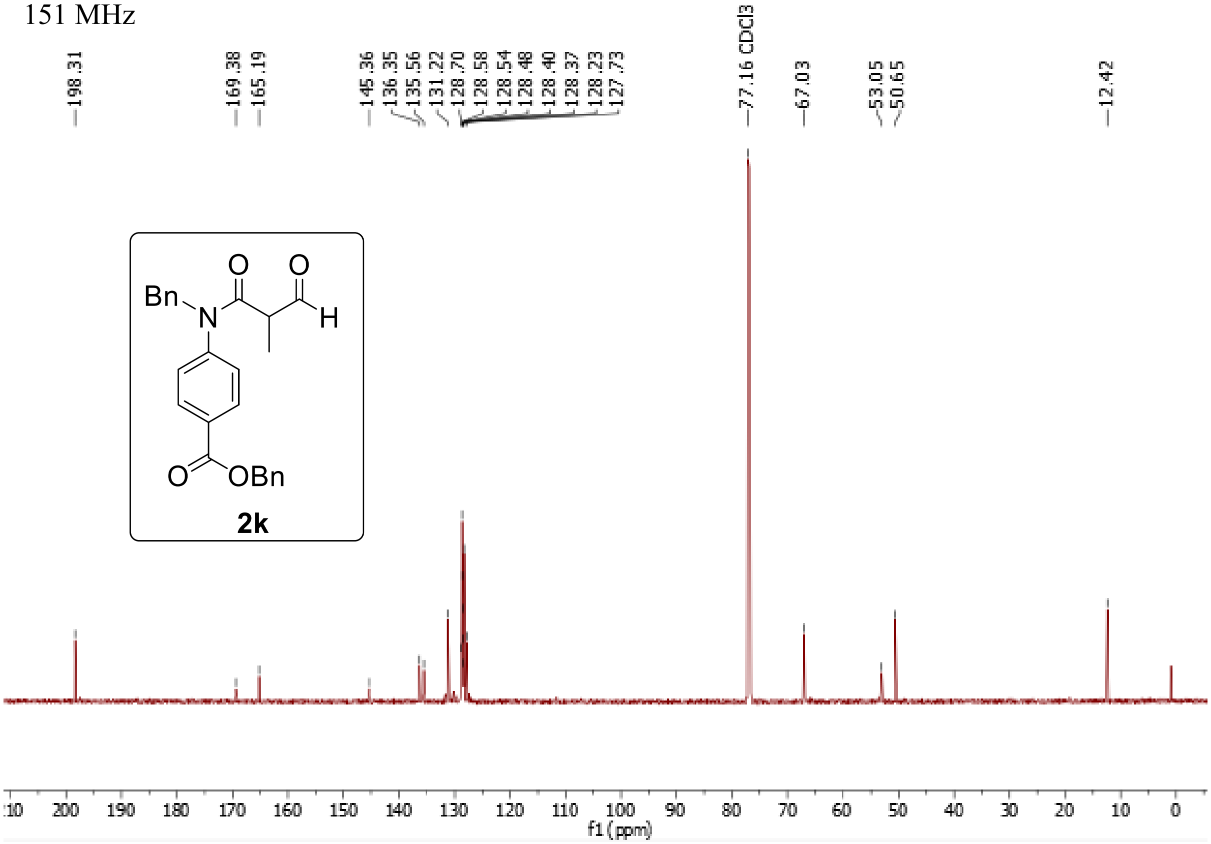

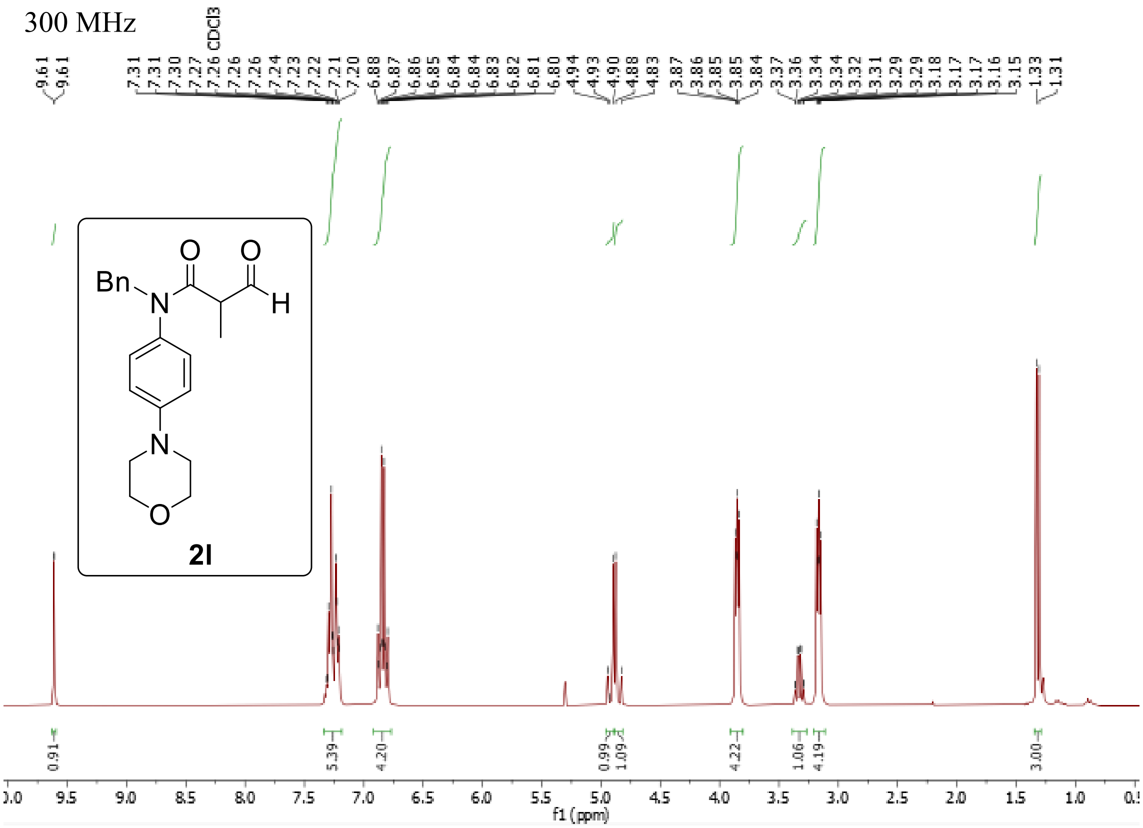
300 MHz
9.61
9.61
7.31
7.31
7.30
7.27
7.26 CDCl3
7.26
7.26
7.24
7.23
7.22
7.21
7.20
6.88
6.87
6.86
6.85
6.84
6.84
6.83
6.82
6.81
6.80
4.94
4.93
4.90
4.88
4.83
3.87
3.86
3.85
3.85
3.84
3.37
3.36
3.34
3.34
3.32
3.31
3.29
3.29
3.18
3.17
3.17
3.16
3.15
1.33
1.31
Bn
N
O
O
H
N
O
2l
0.91
5.39
4.20
0.99
1.09
4.22
1.06
4.19
3.00
f1 (ppm)

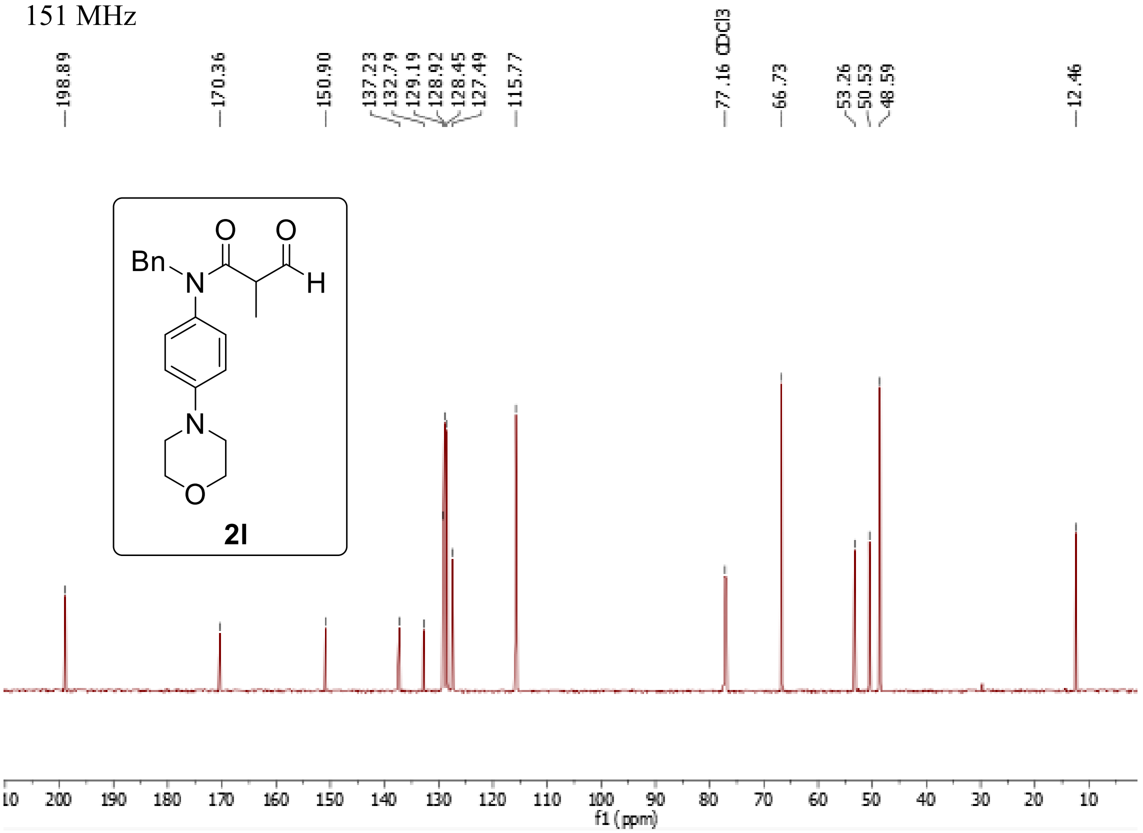
151 MHz
198.89
170.36
150.90
137.23
132.79
129.19
128.92
128.45
127.49
115.77
77.16 CDCl3
66.73
53.26
50.53
48.59
12.46
Bn
N
O
O
H
N
O
2l
f1 (ppm)

300 MHz

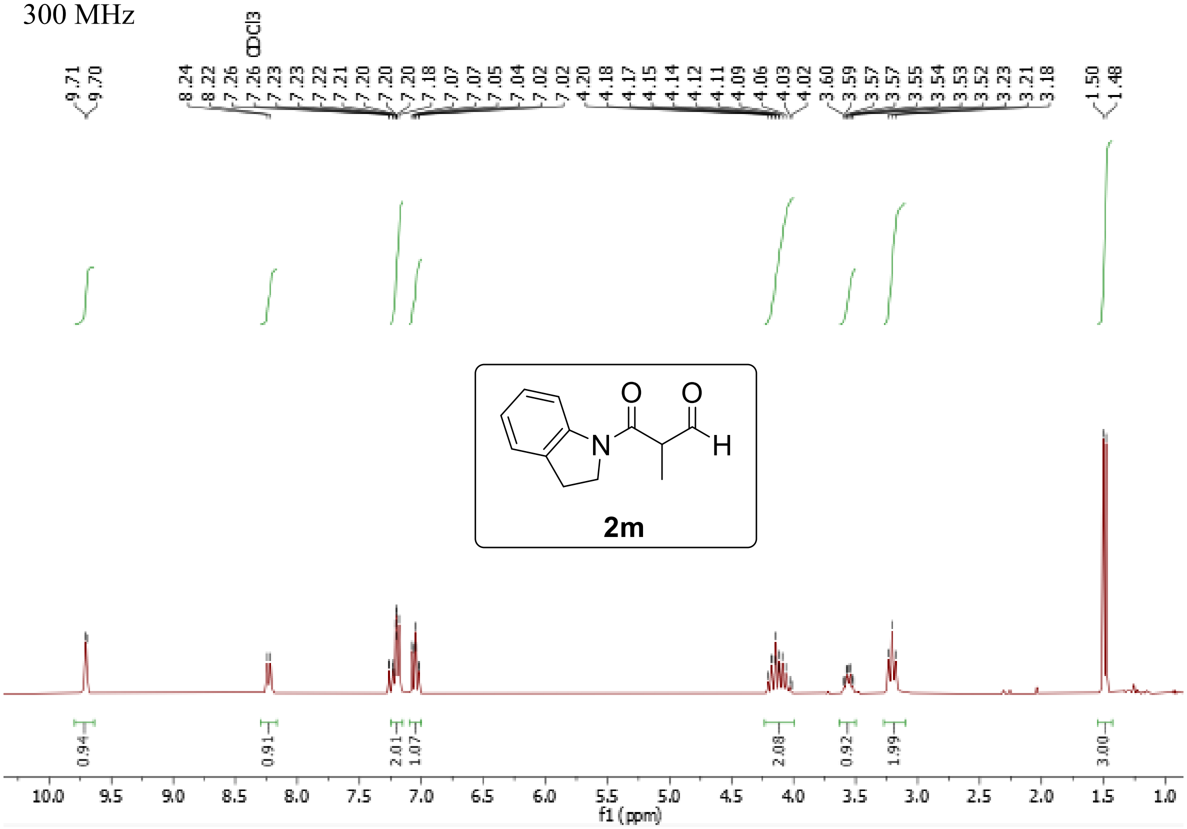

151 MHz

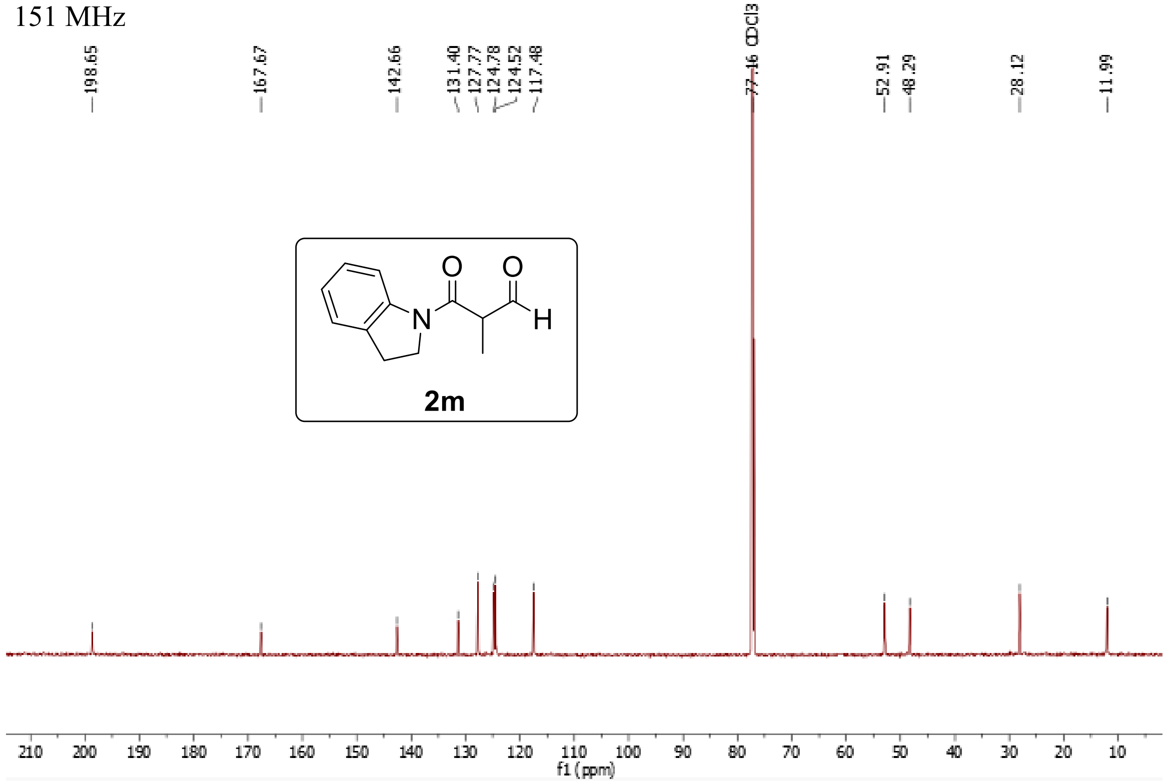

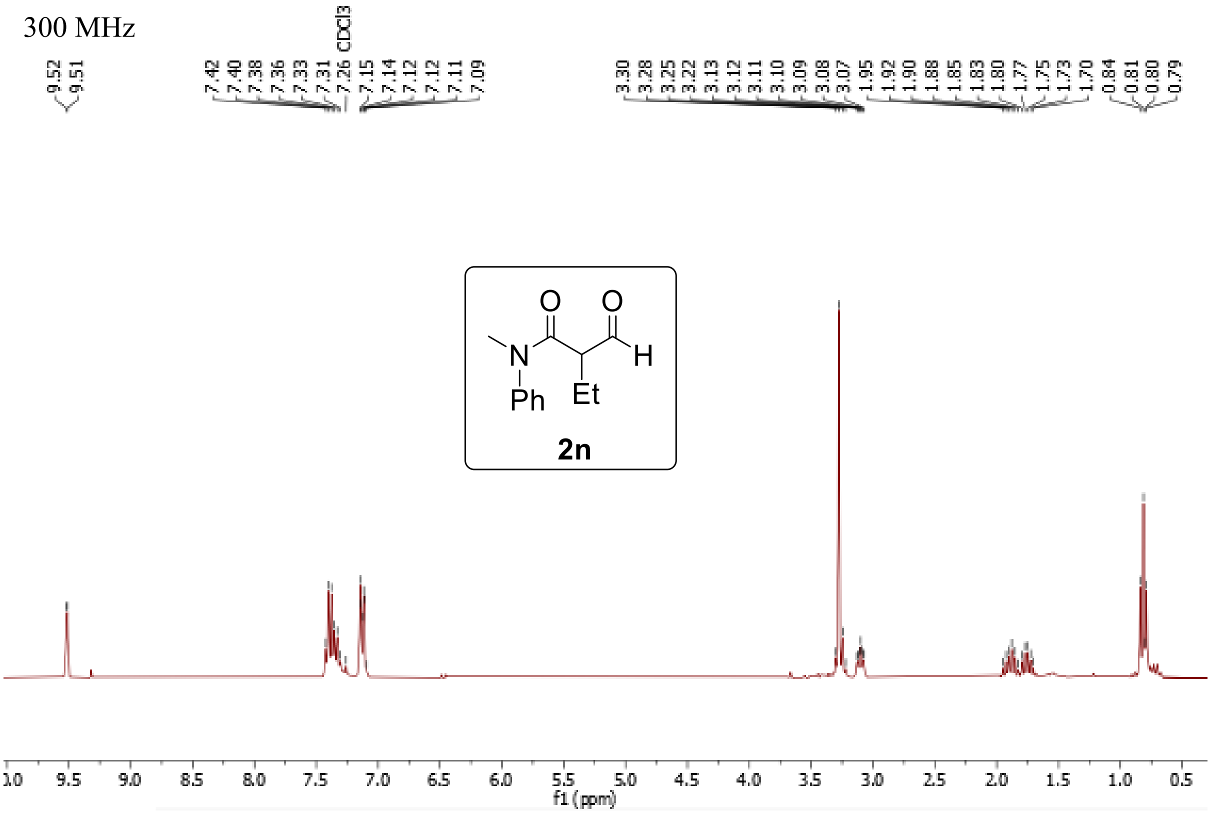

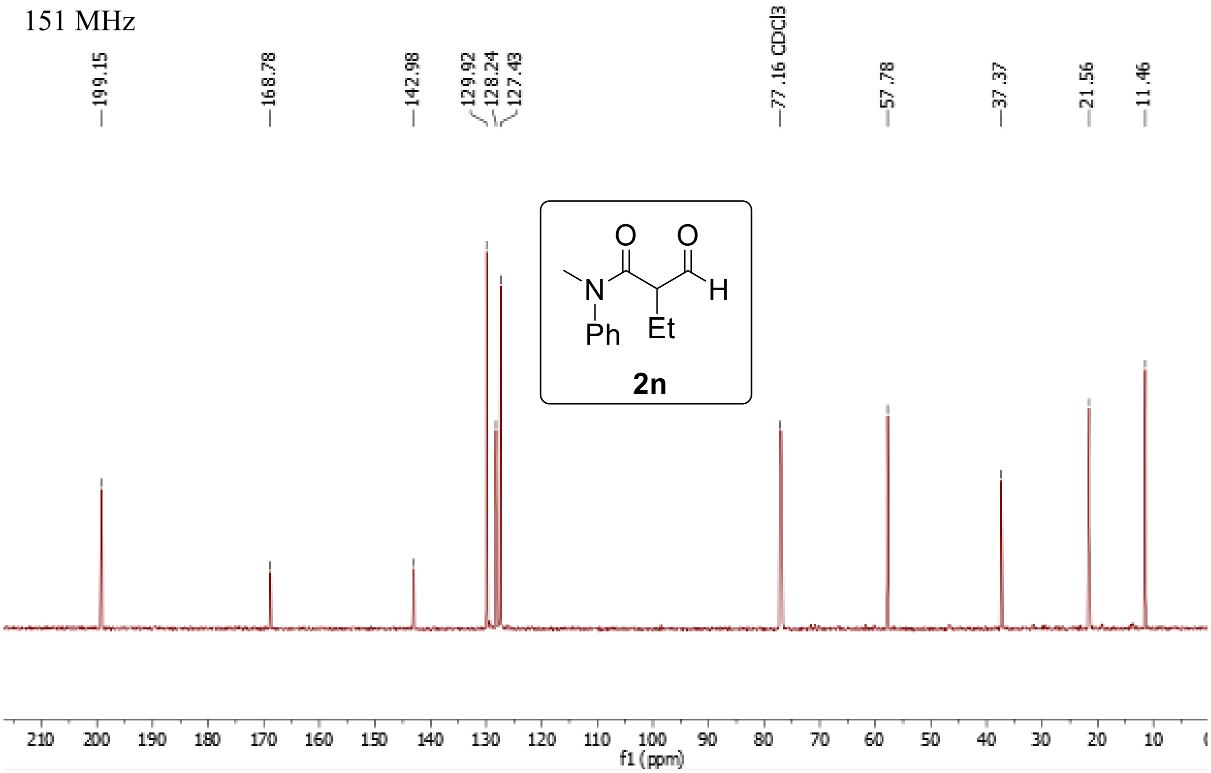

300 MHz

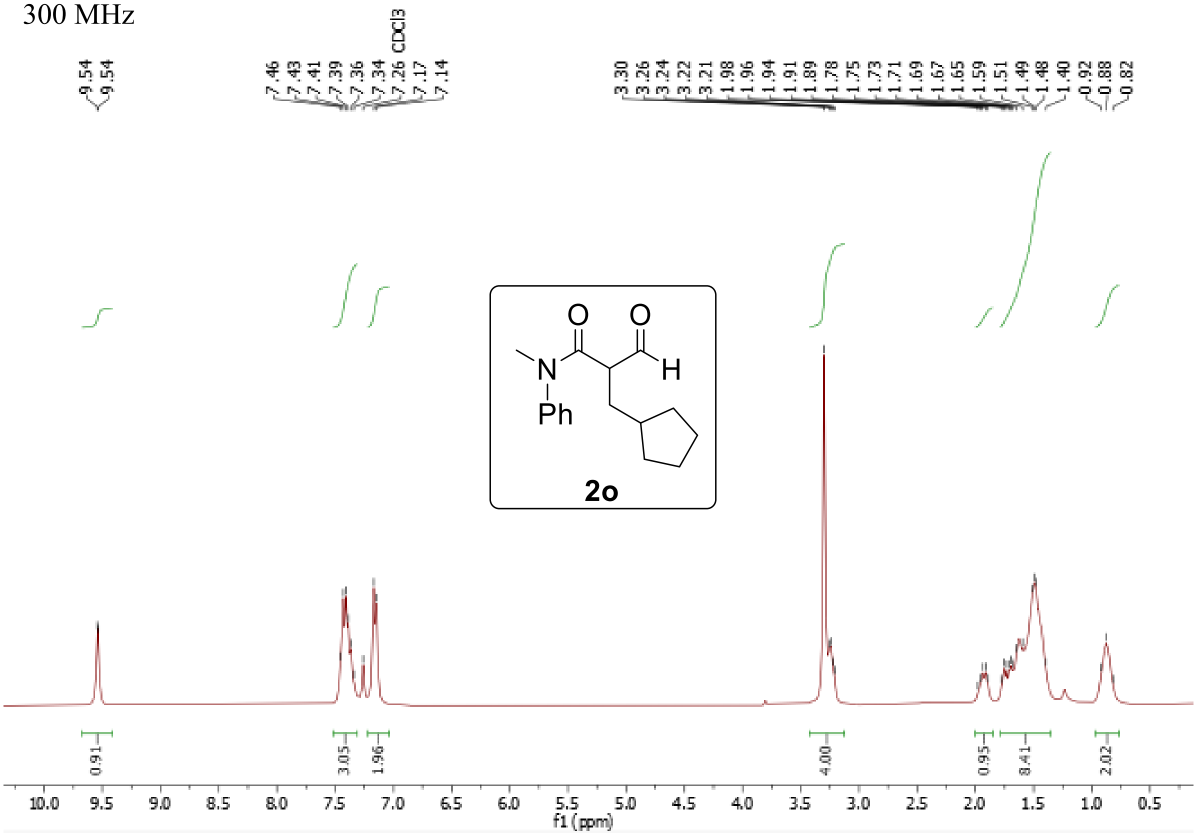

151 MHz

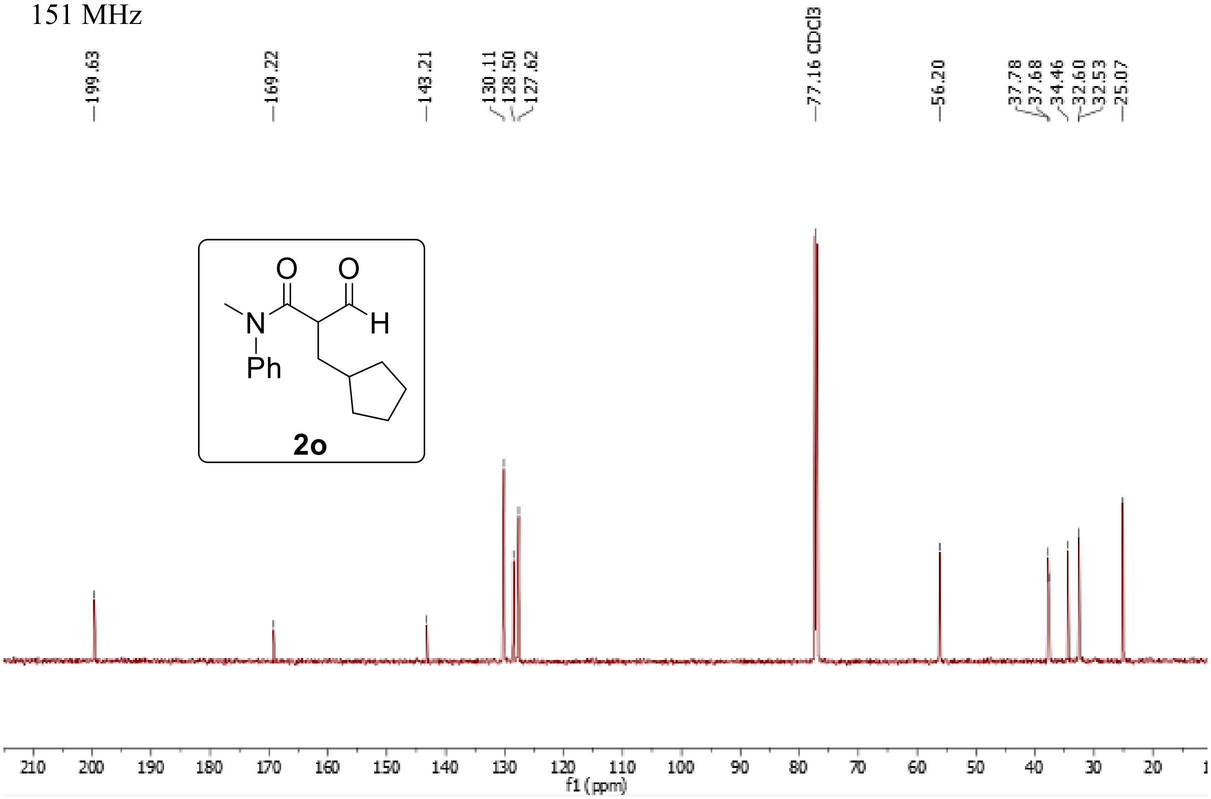

300 MHz

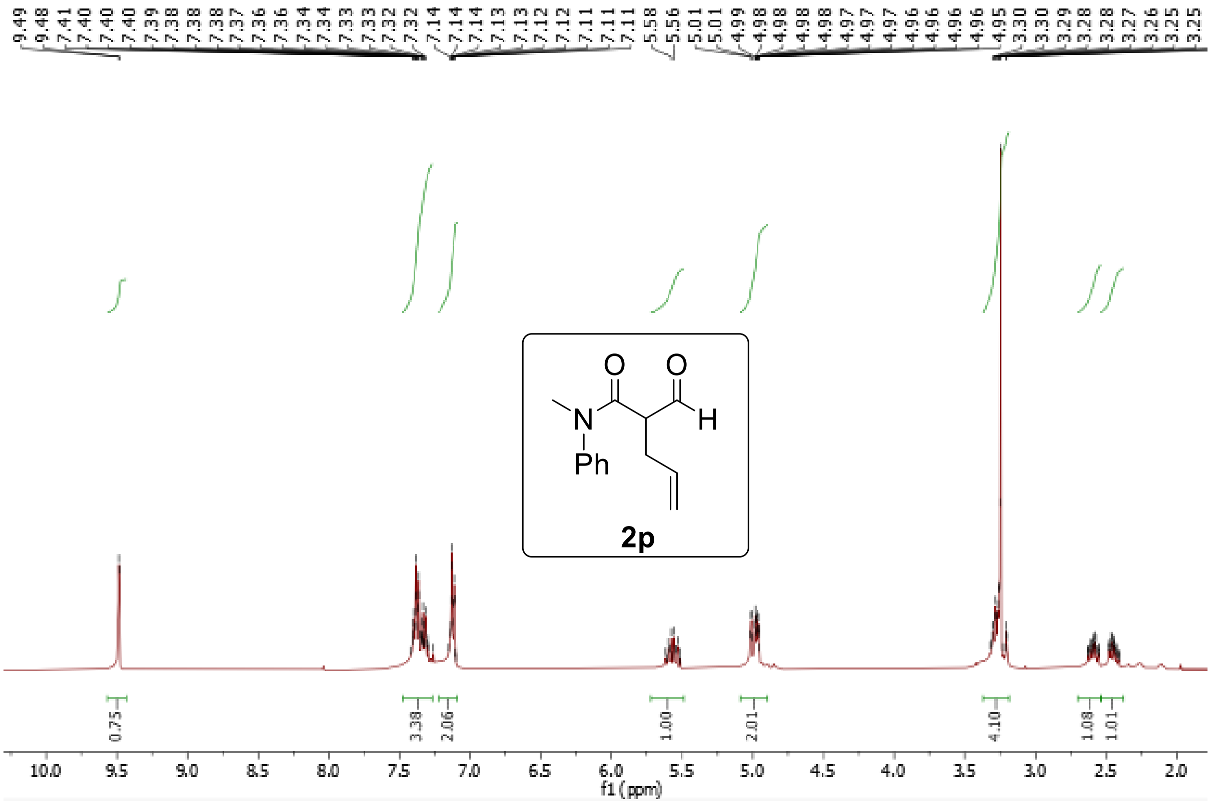

101 MHz

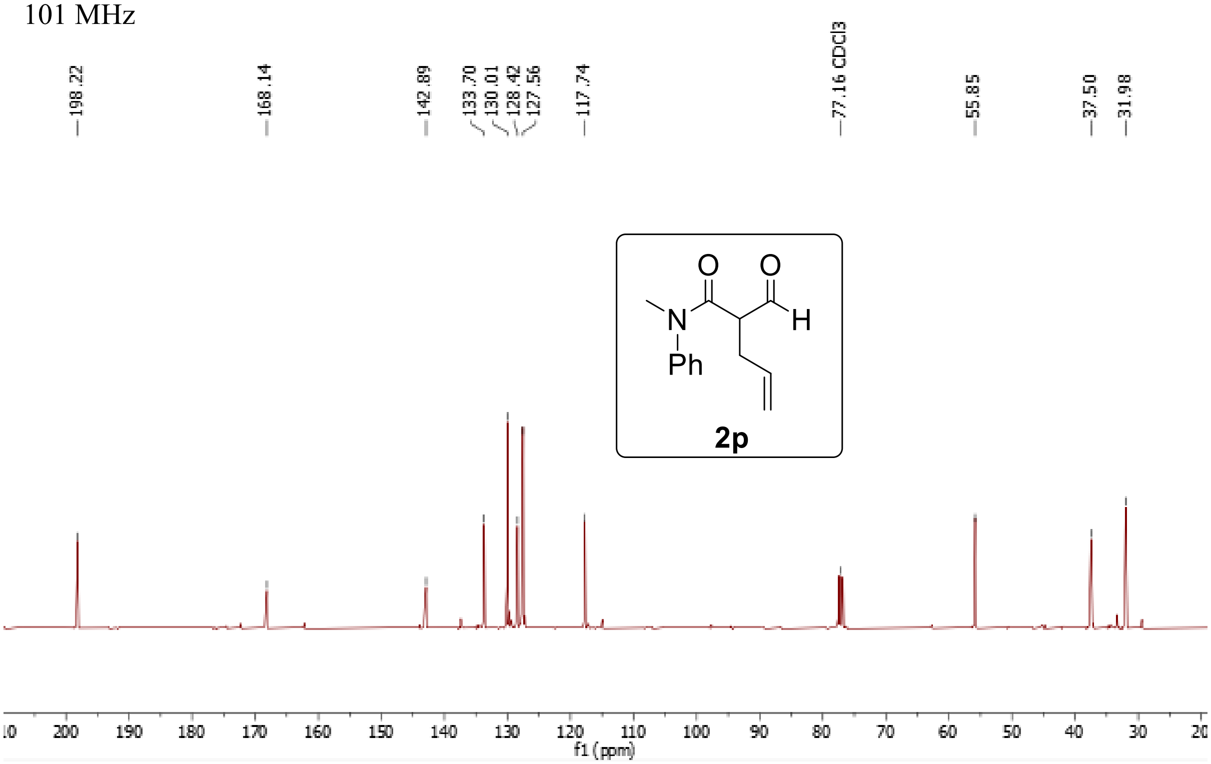

400 MHz

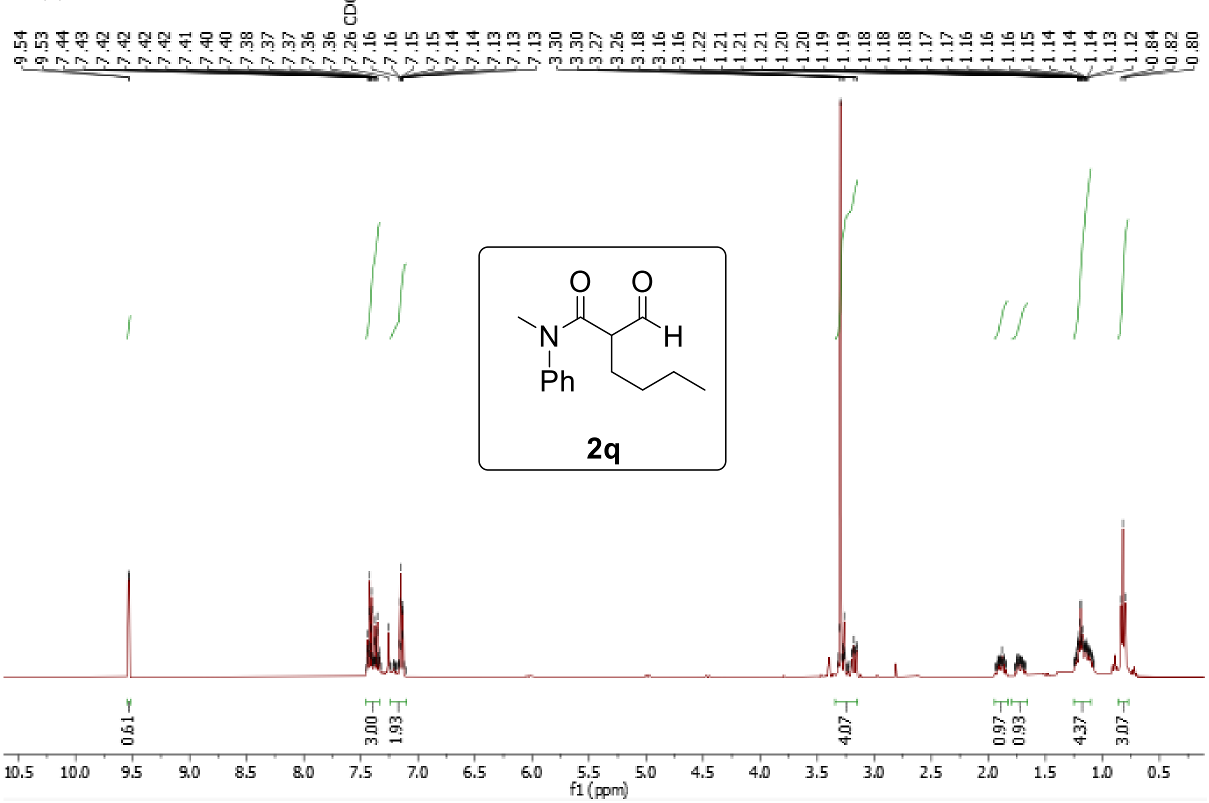

101 MHz

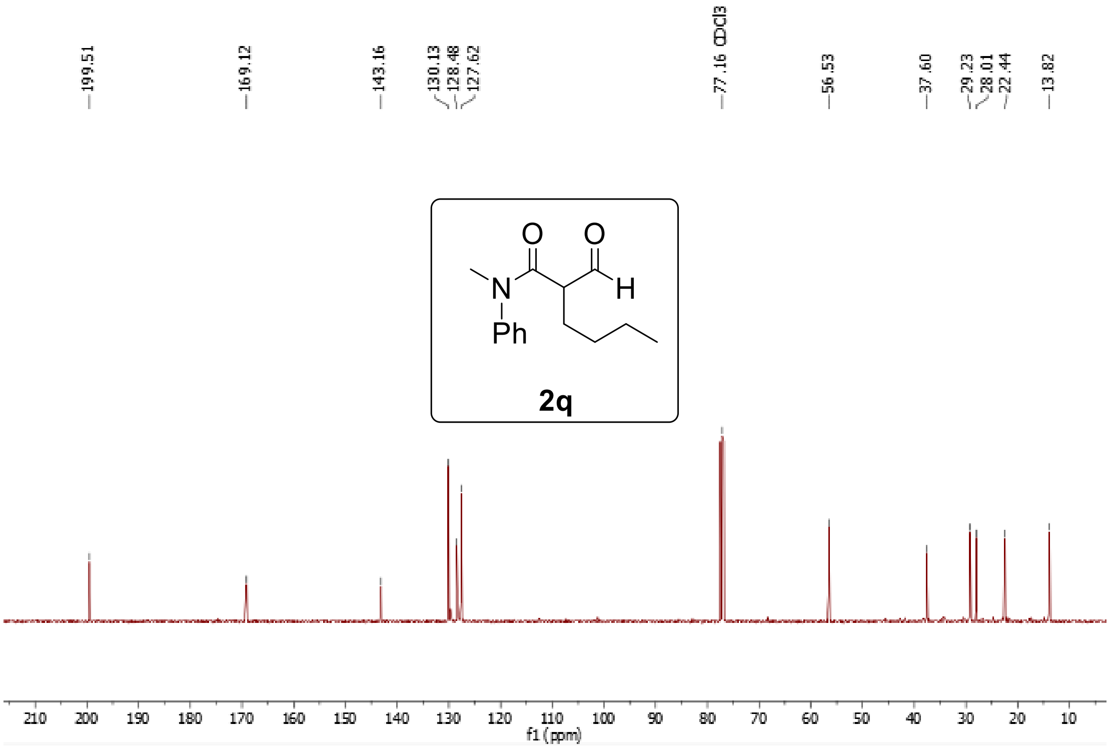

400 MHz

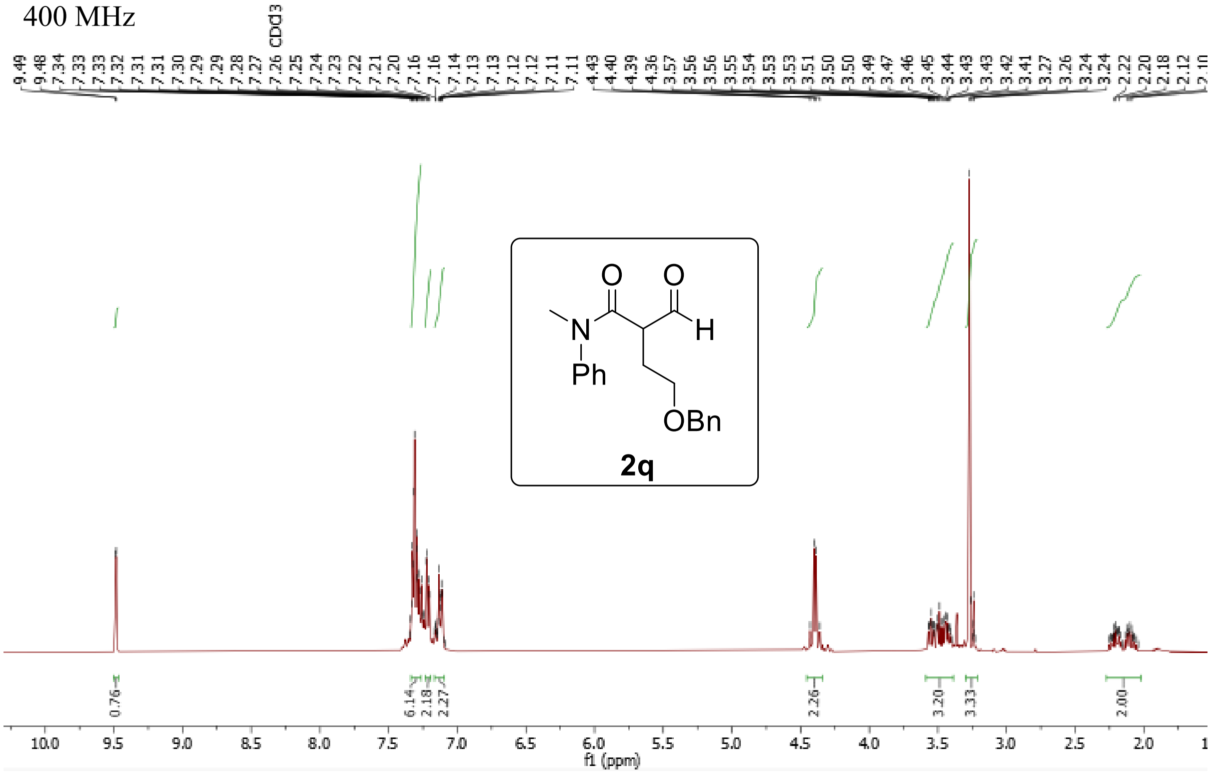

101 MHz

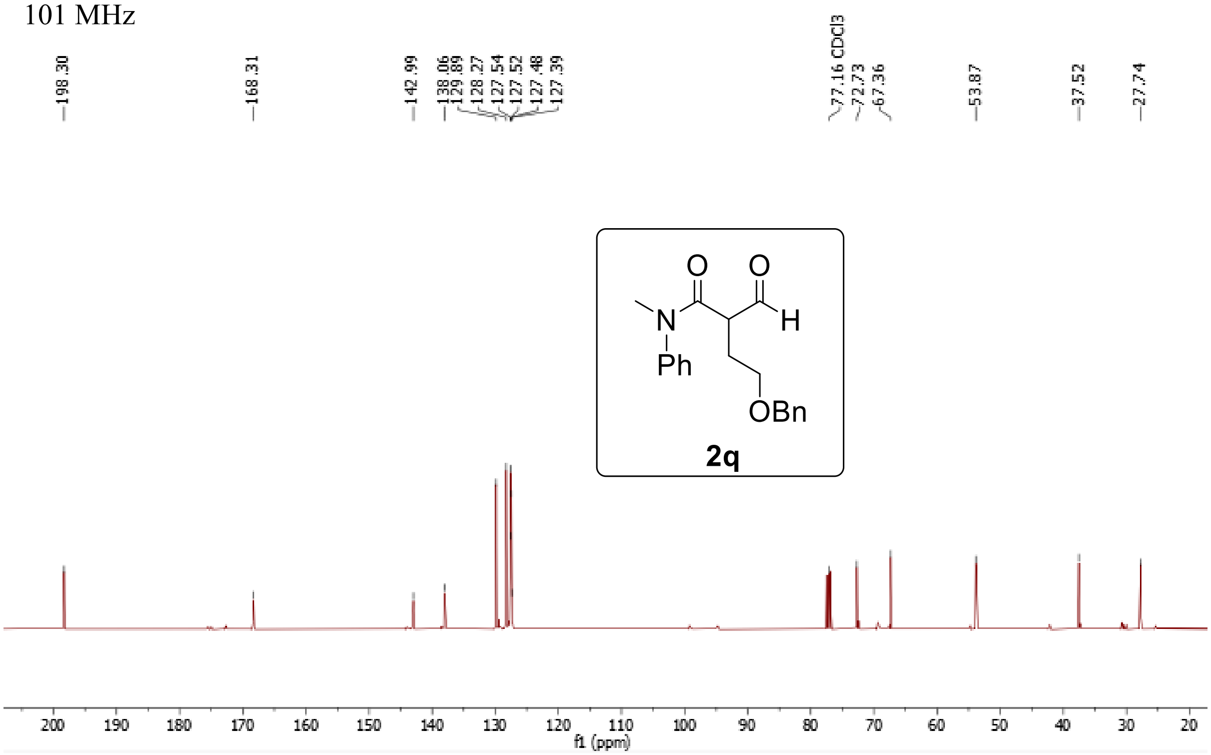

400 MHz

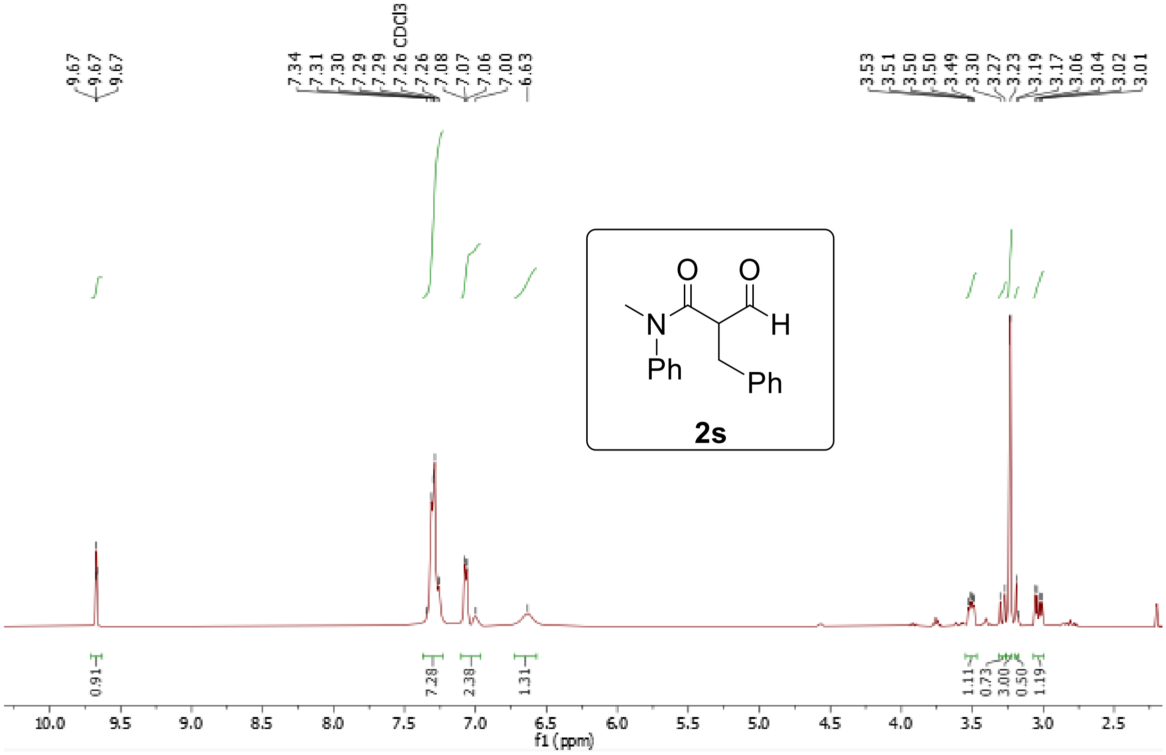

101 MHz

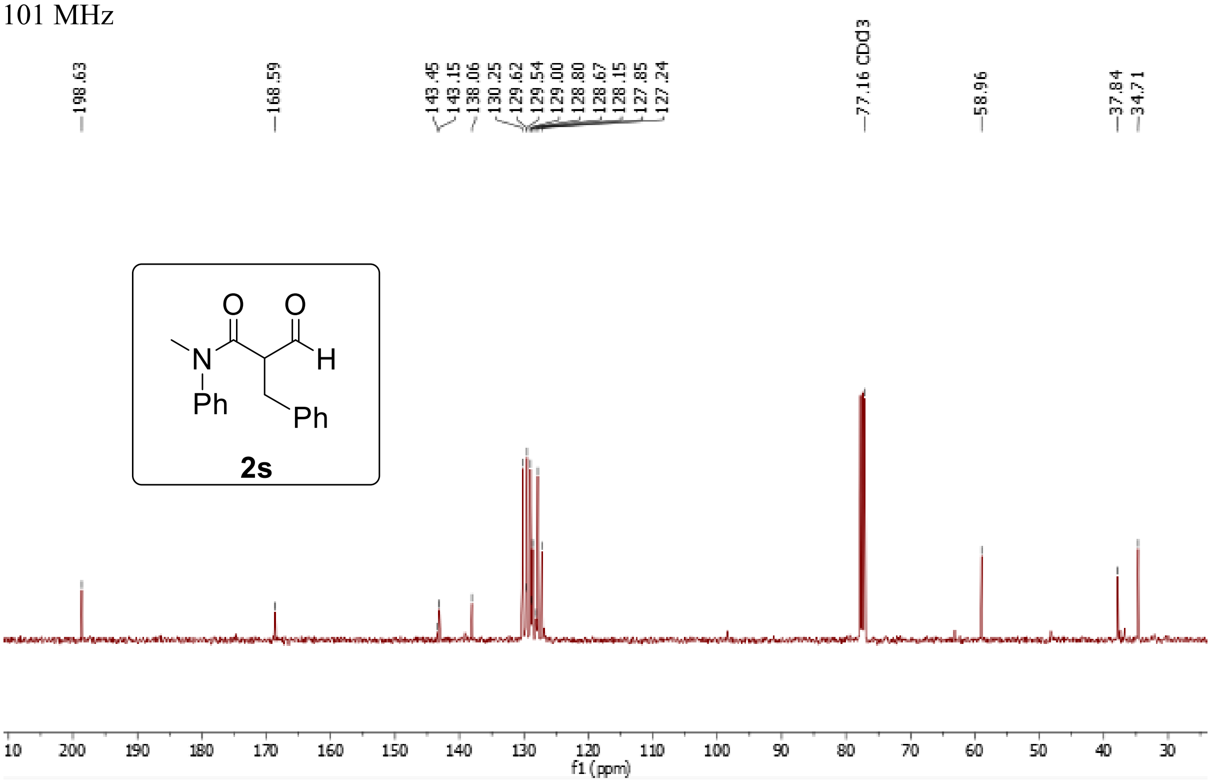

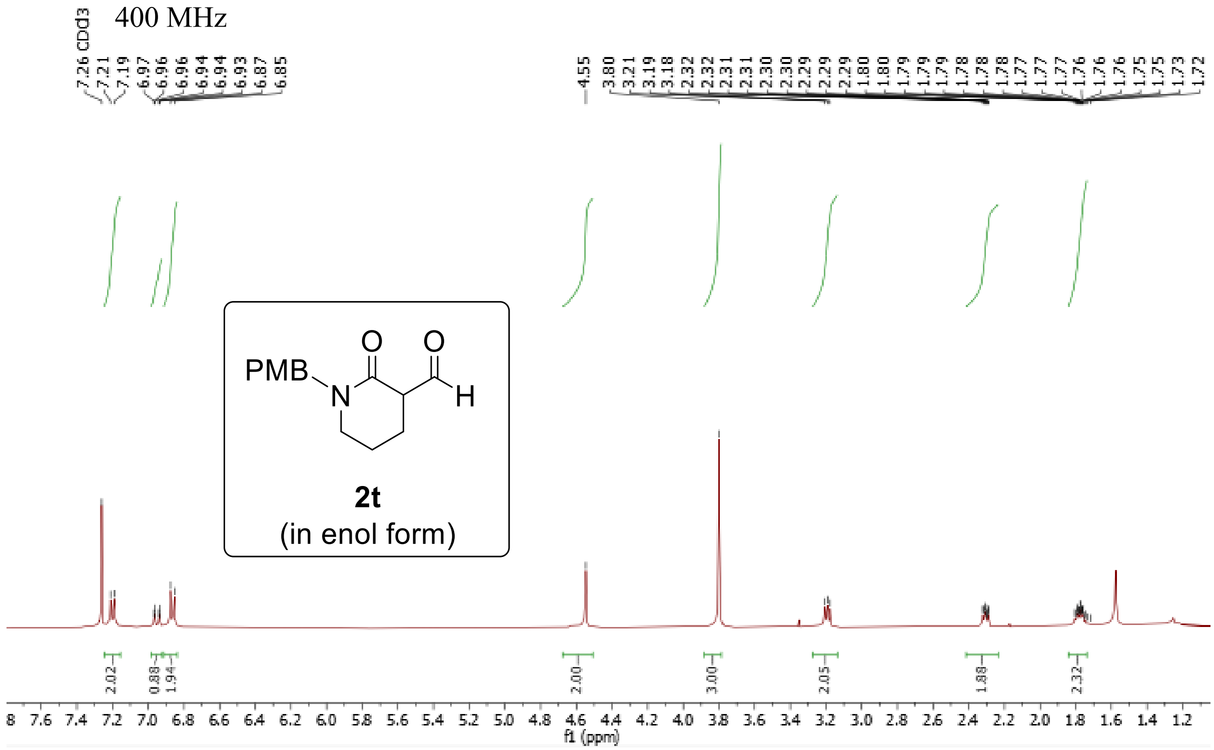
400 MHz
7.26 CDCl3
7.21
7.19
6.97
6.96
6.96
6.94
6.94
6.93
6.87
6.85
4.55
3.80
3.21
3.19
3.18
2.32
2.32
2.31
2.31
2.30
2.30
2.29
2.29
2.29
1.80
1.80
1.79
1.79
1.79
1.78
1.78
1.78
1.77
1.77
1.77
1.76
1.76
1.76
1.75
1.75
1.73
1.72
PMB
N
O
O
H
2t
(in enol form)
2.02
0.88
1.94
2.00
3.00
2.05
1.88
2.32
f1 (ppm)

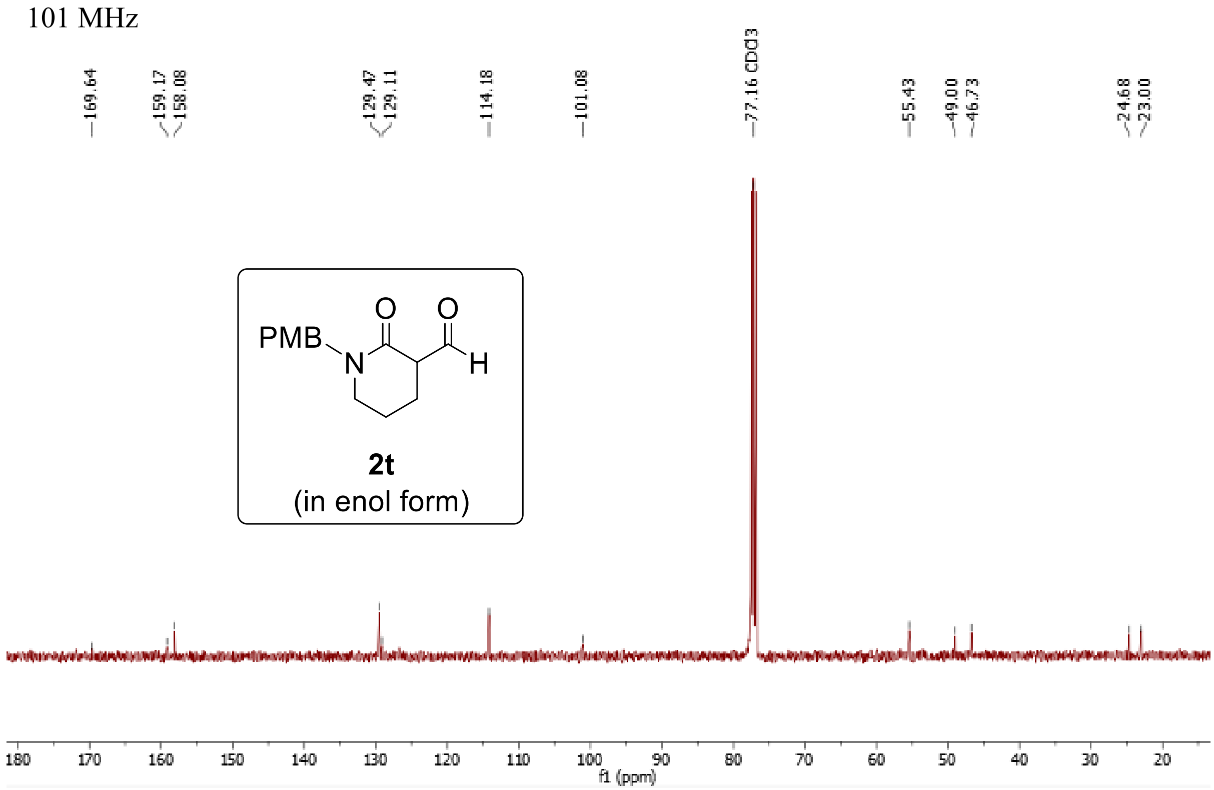
101 MHz
169.64
159.17
158.08
129.47
129.11
114.18
101.08
77.16 CDCl3
55.43
49.00
46.73
24.68
23.00
PMB
N
O
O
H
2t
(in enol form)
f1 (ppm)

400 MHz

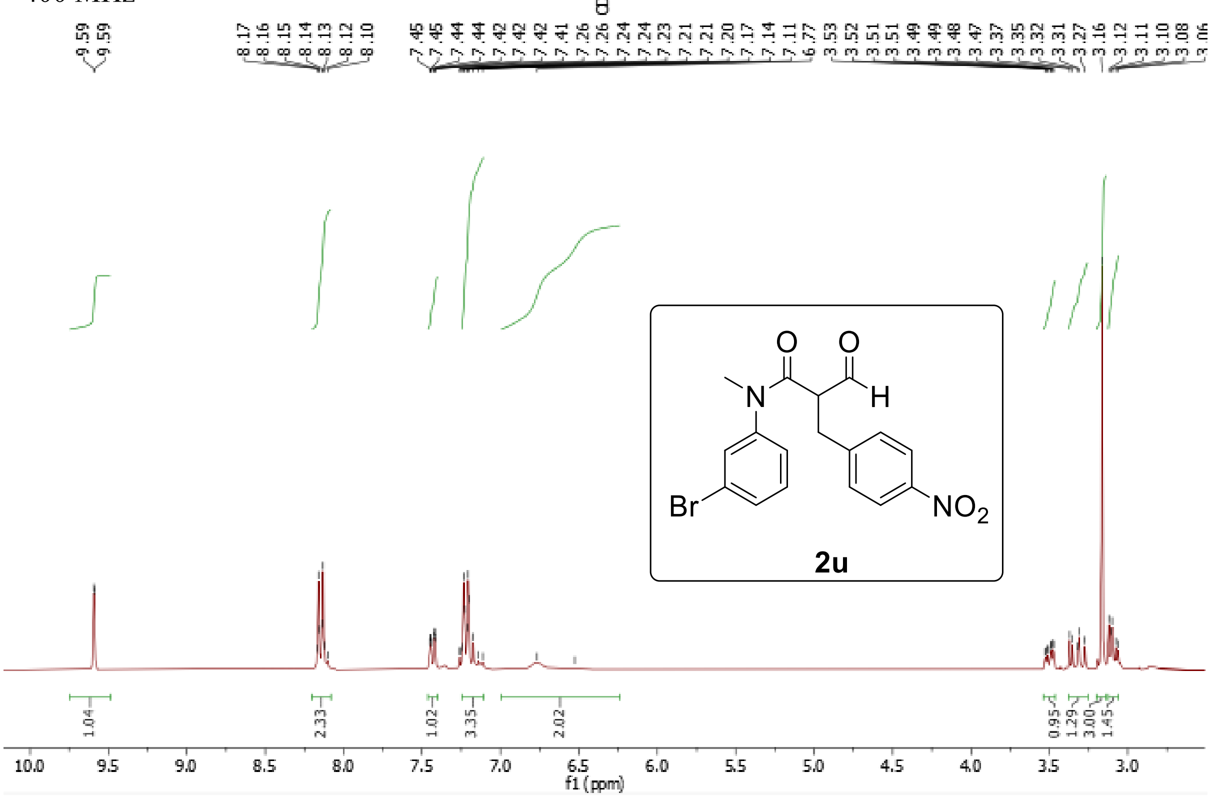

101 MHz

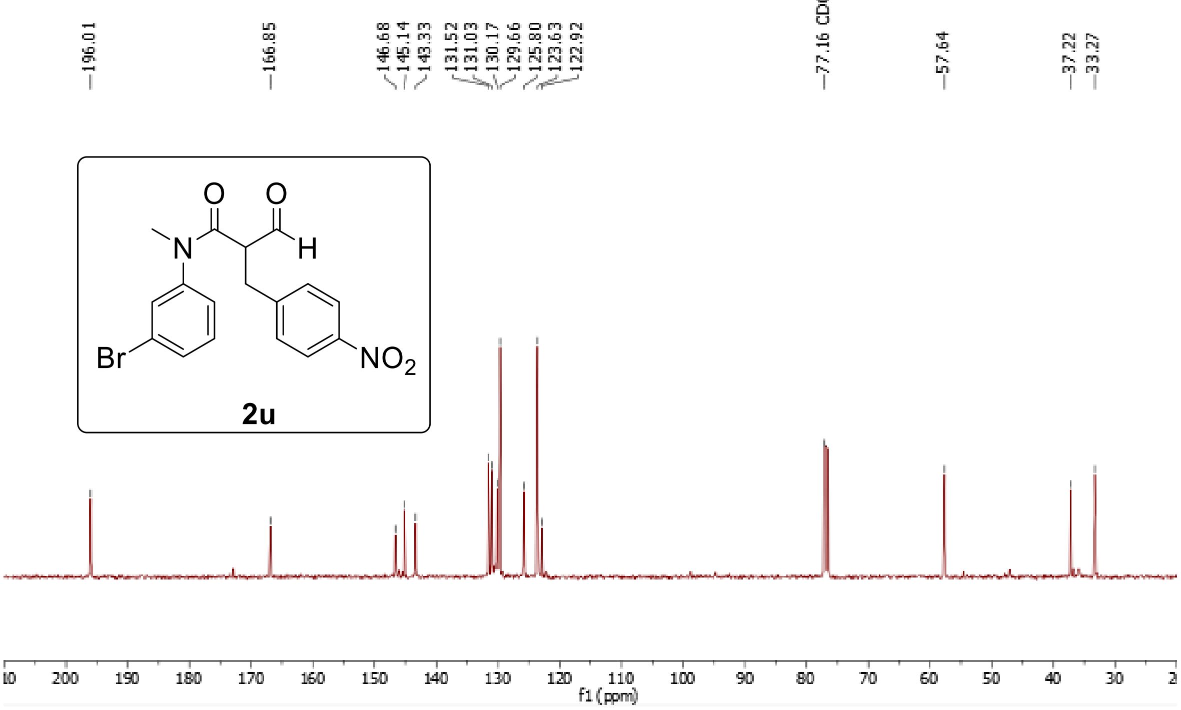

(iii) 3a-u

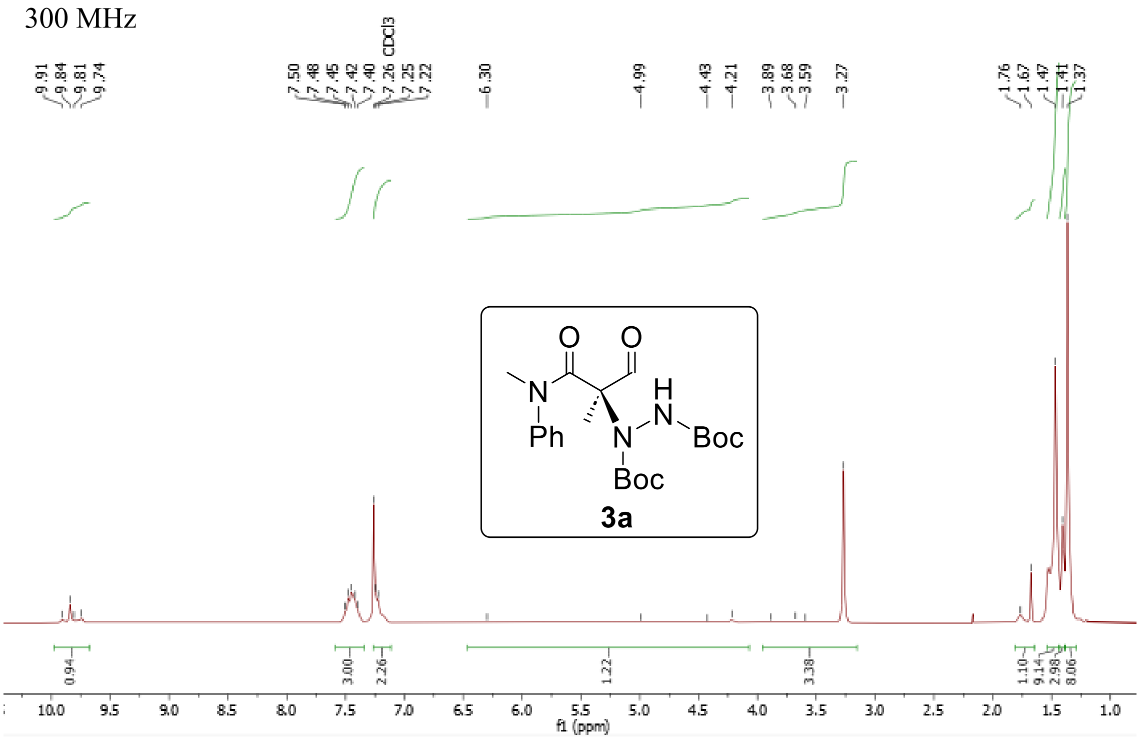

VT [1]H Spectrum for 3a at 353K (600 MHz)

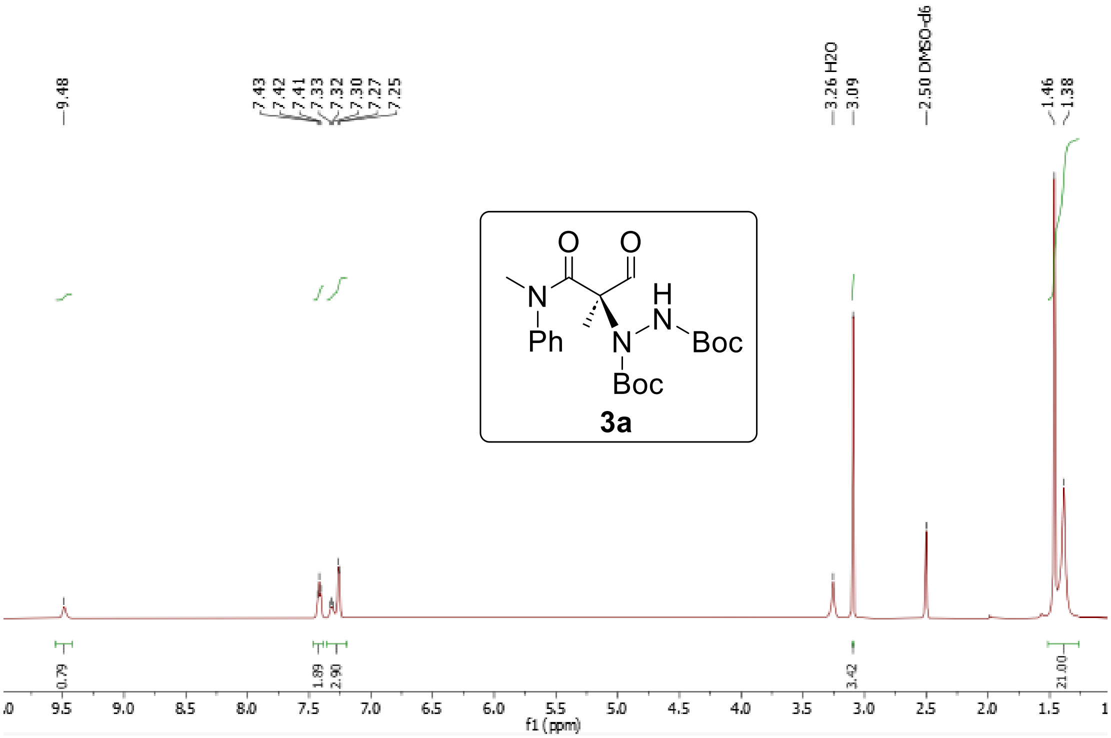

101 MHz

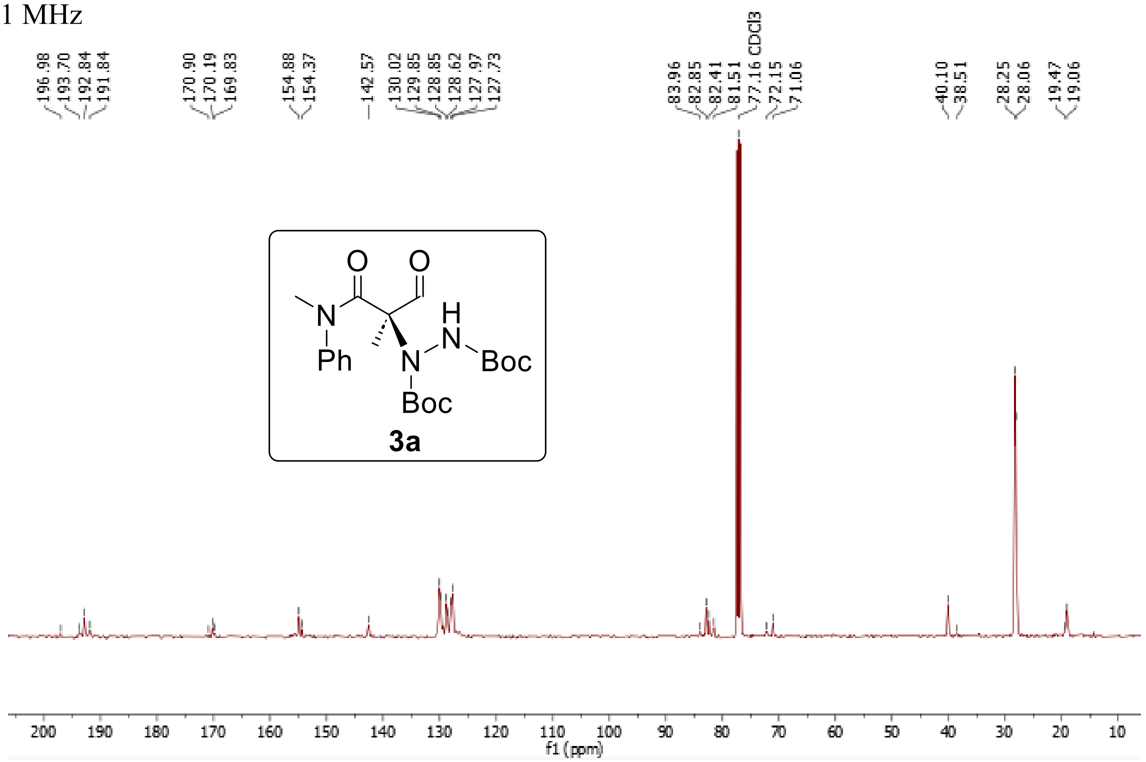

300 MHz

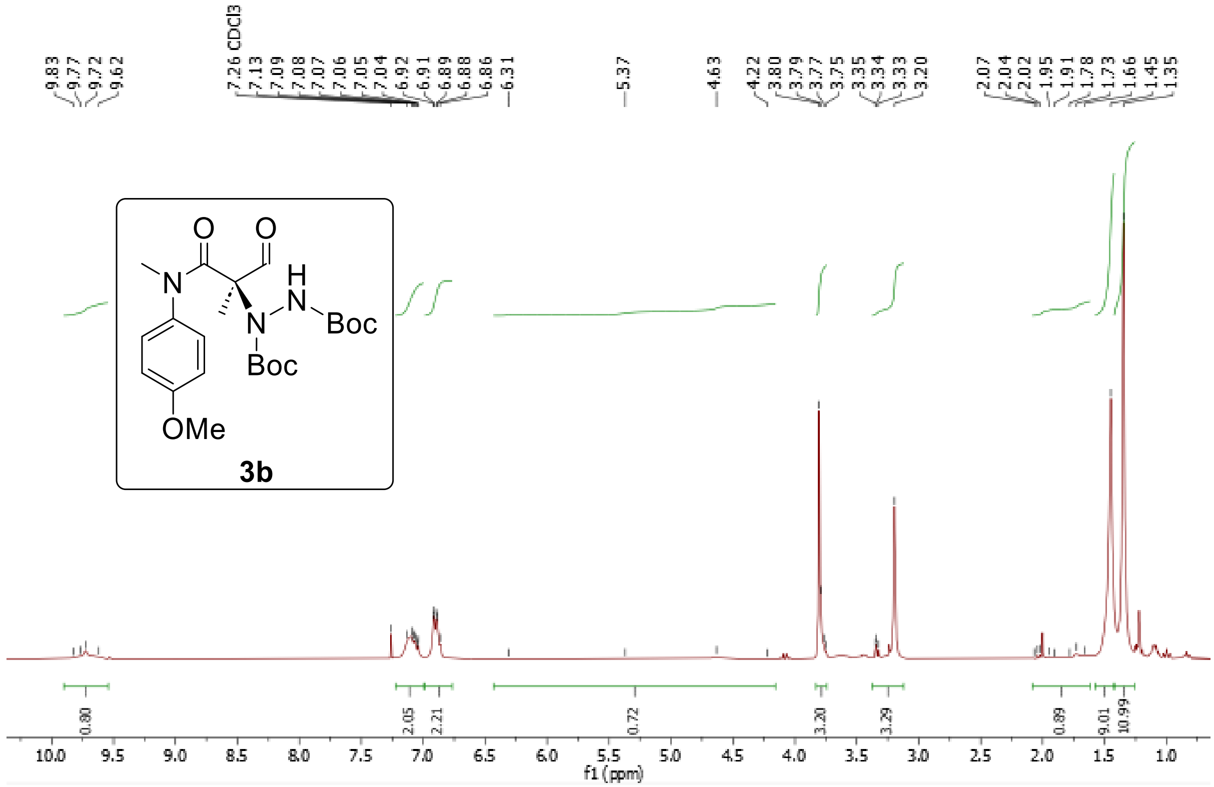

101 MHz

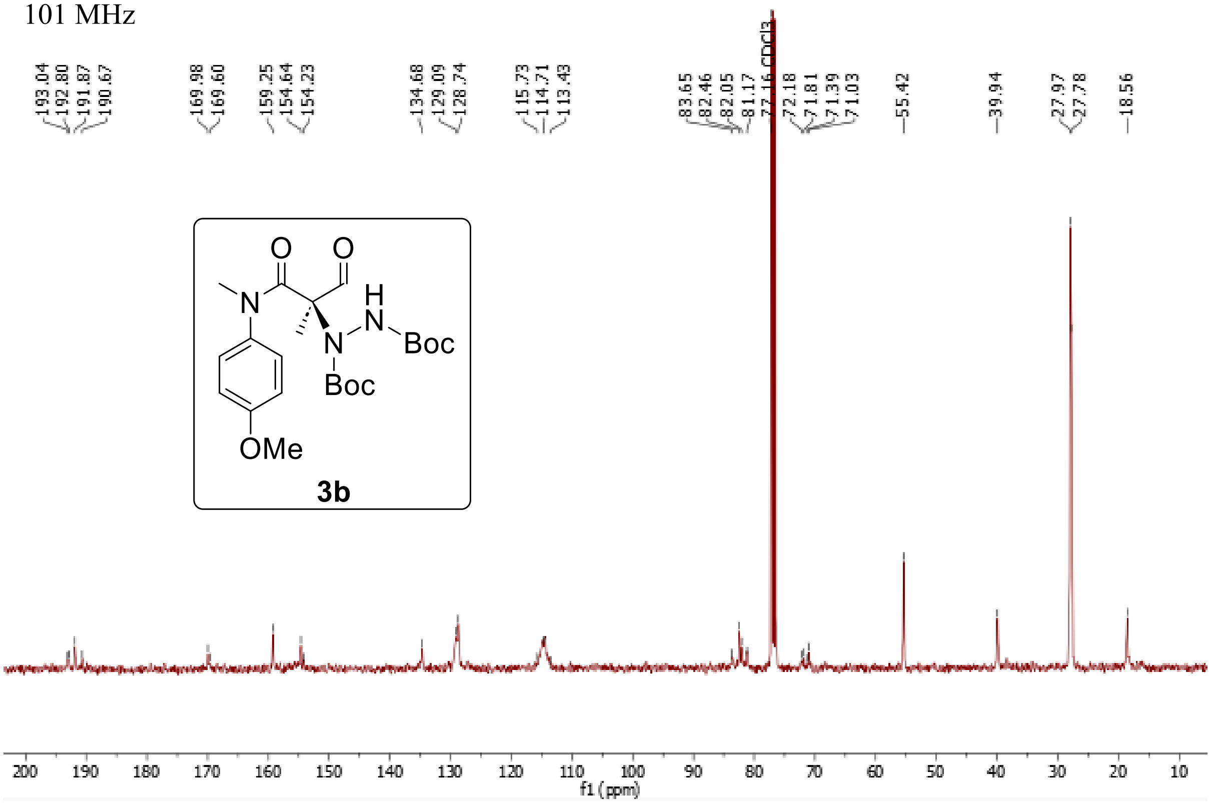

300 MHz

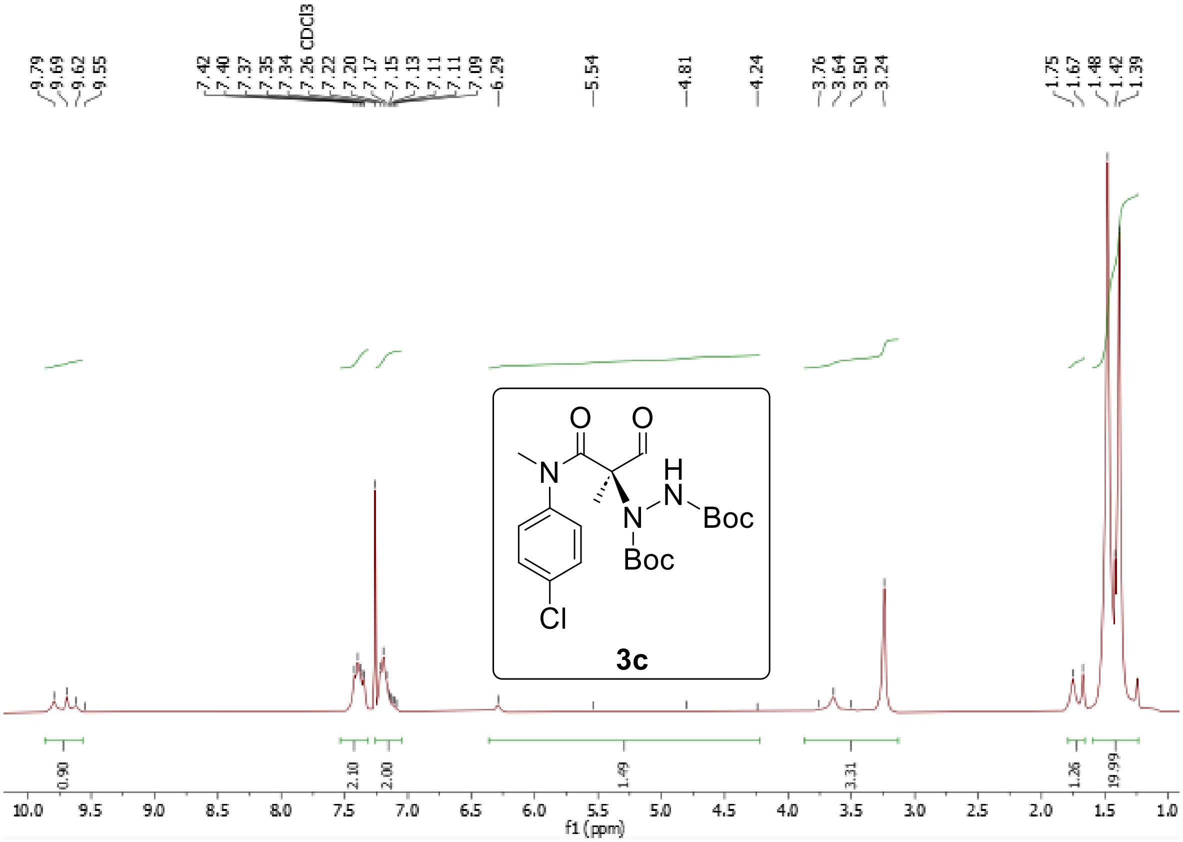

101 MHz

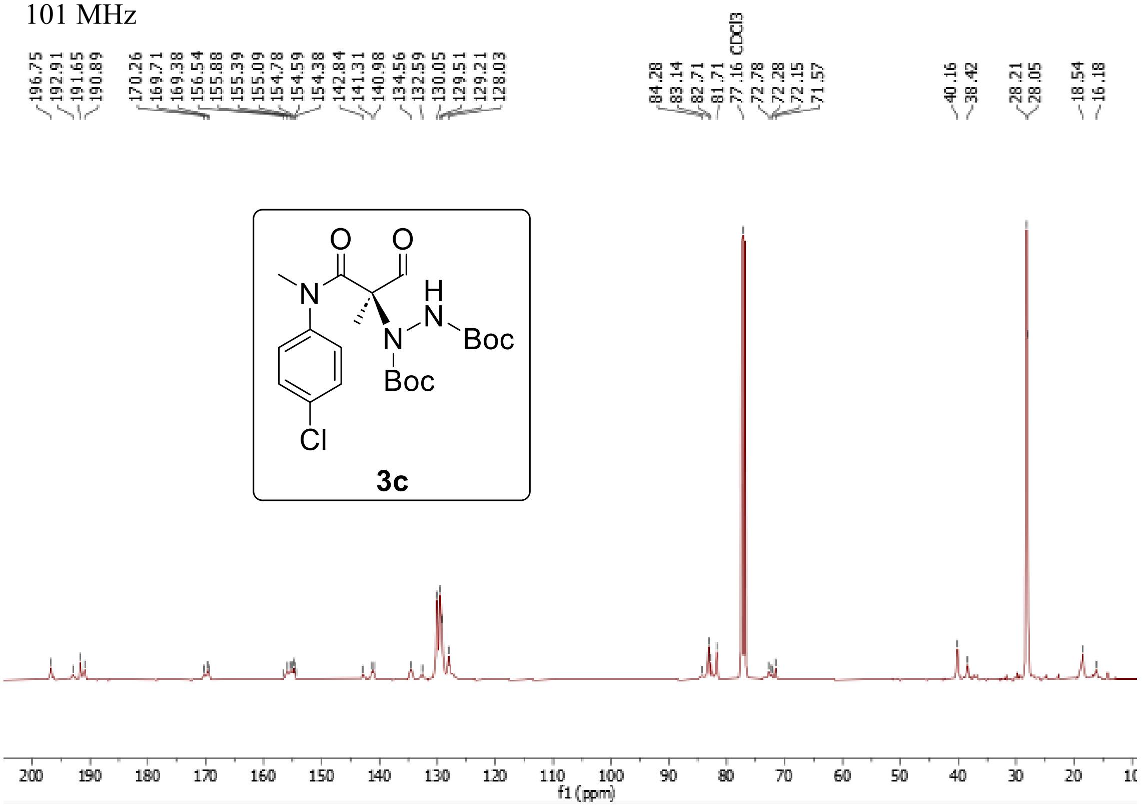

300 MHz

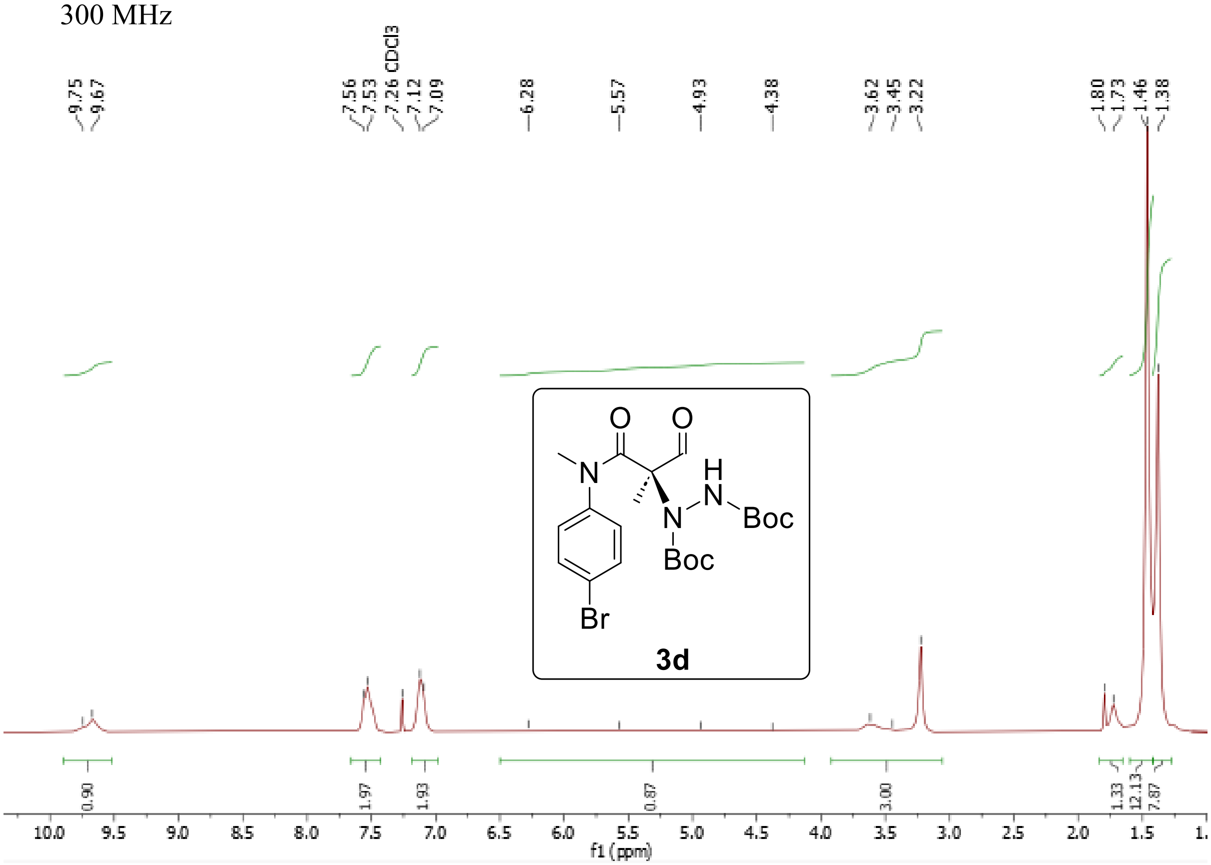

151 MHz

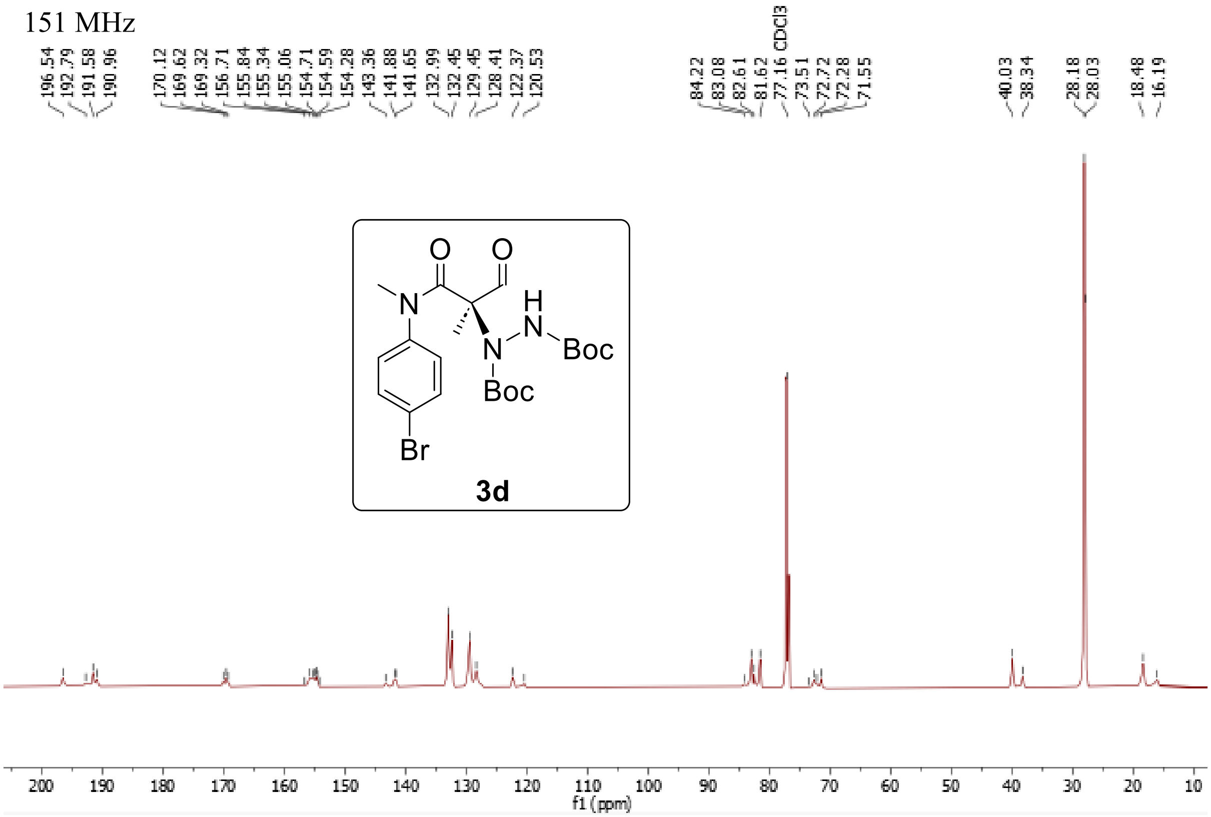

300 MHz

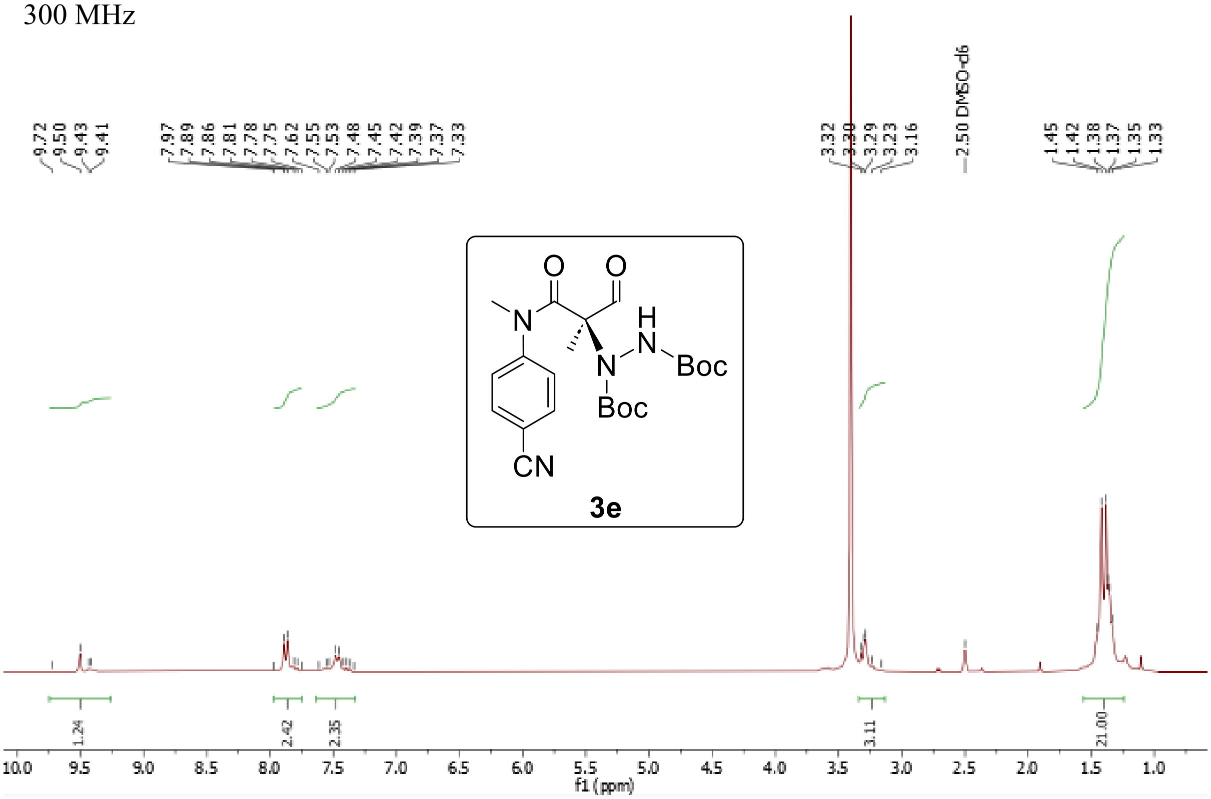

101 MHz

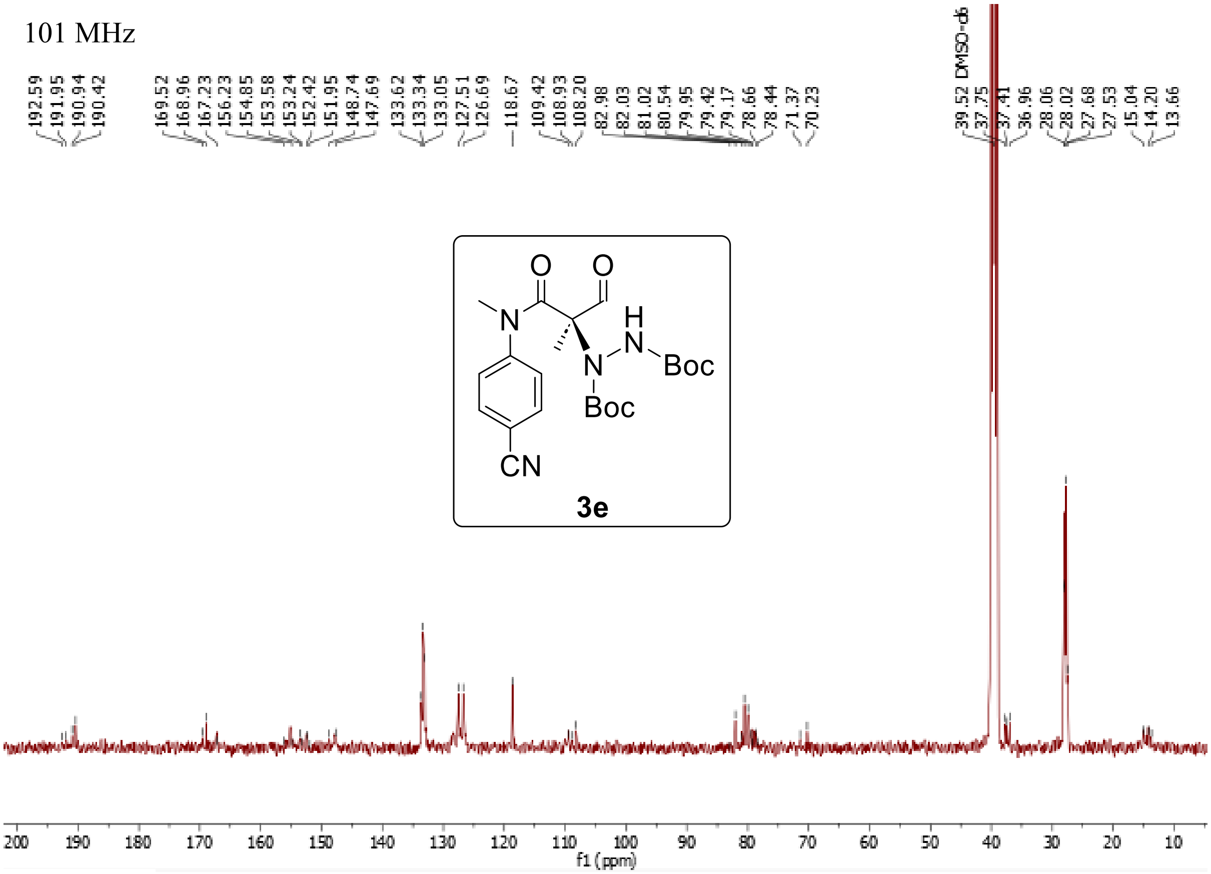

300 MHz

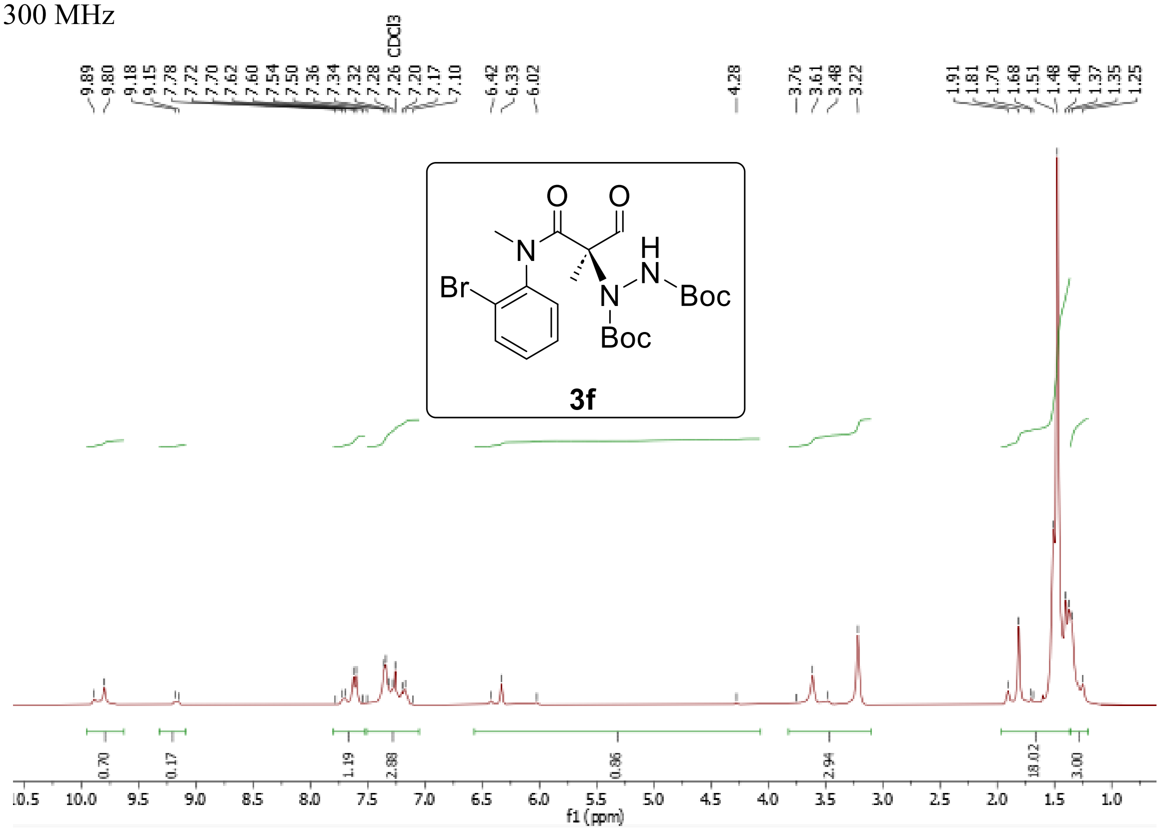

101 MHz

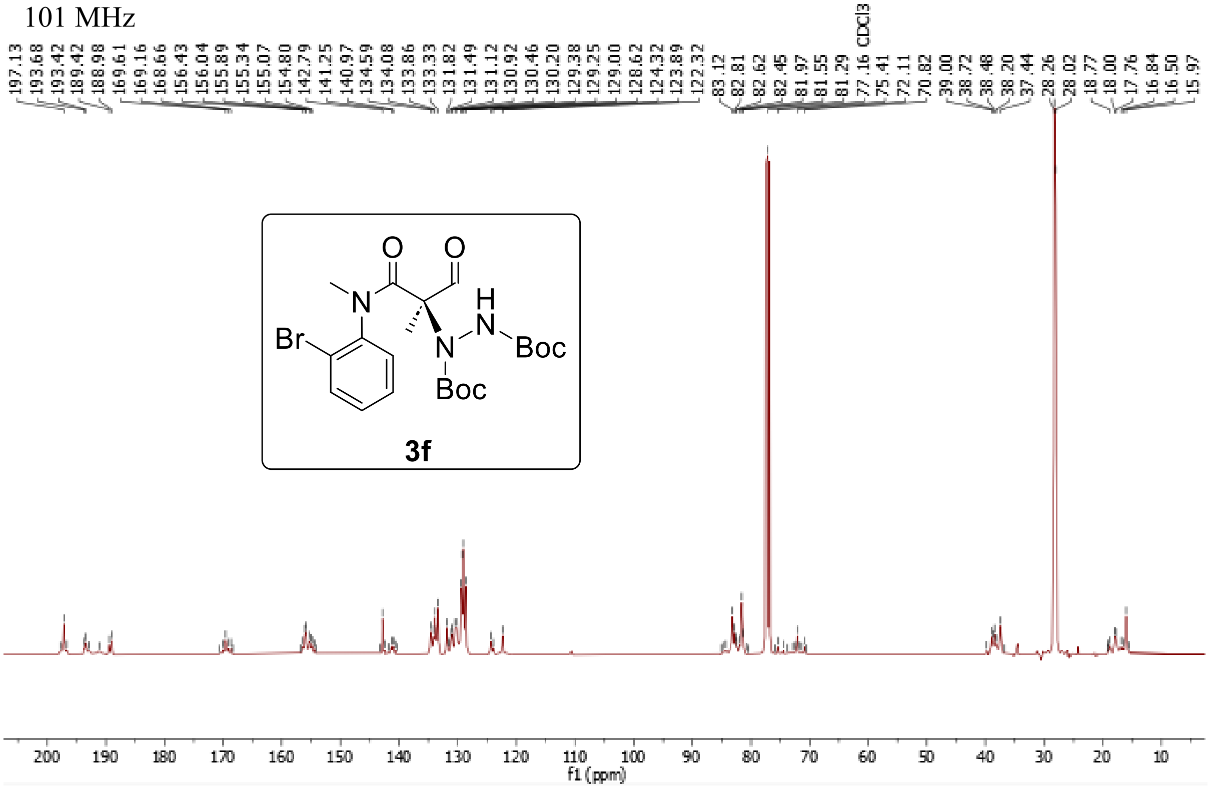

300 MHz

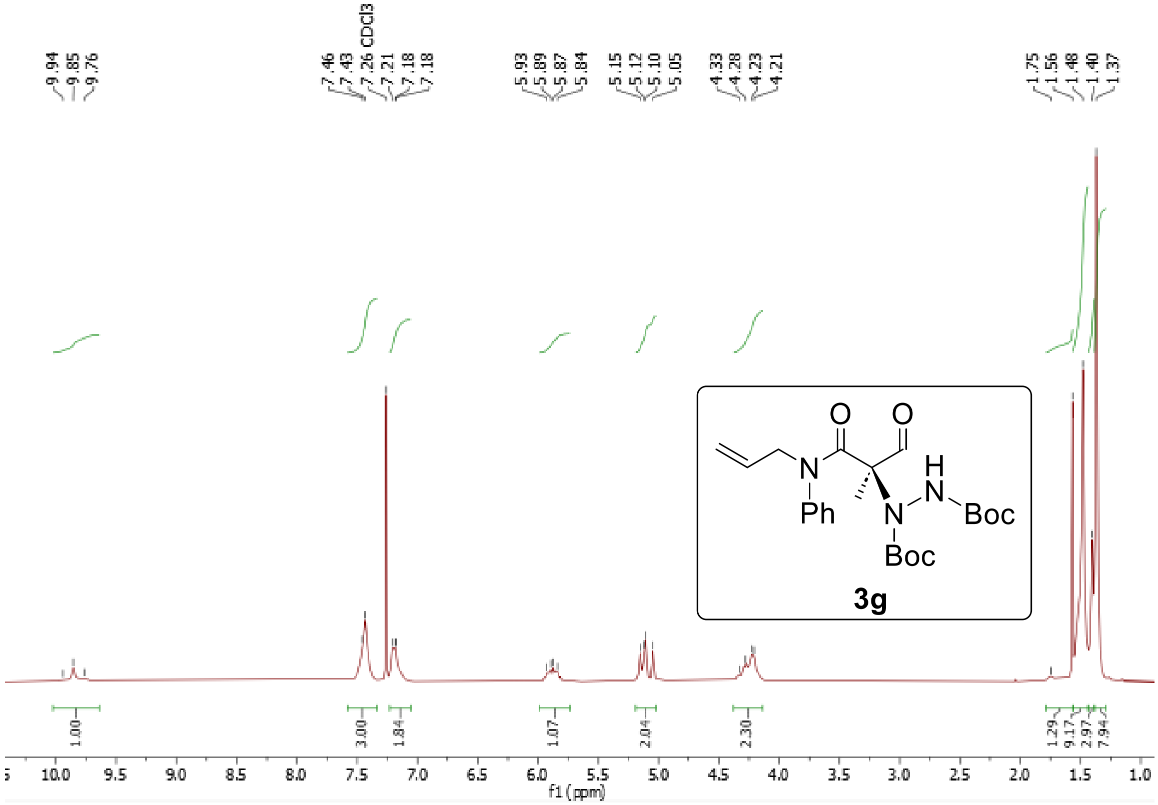

101 MHz

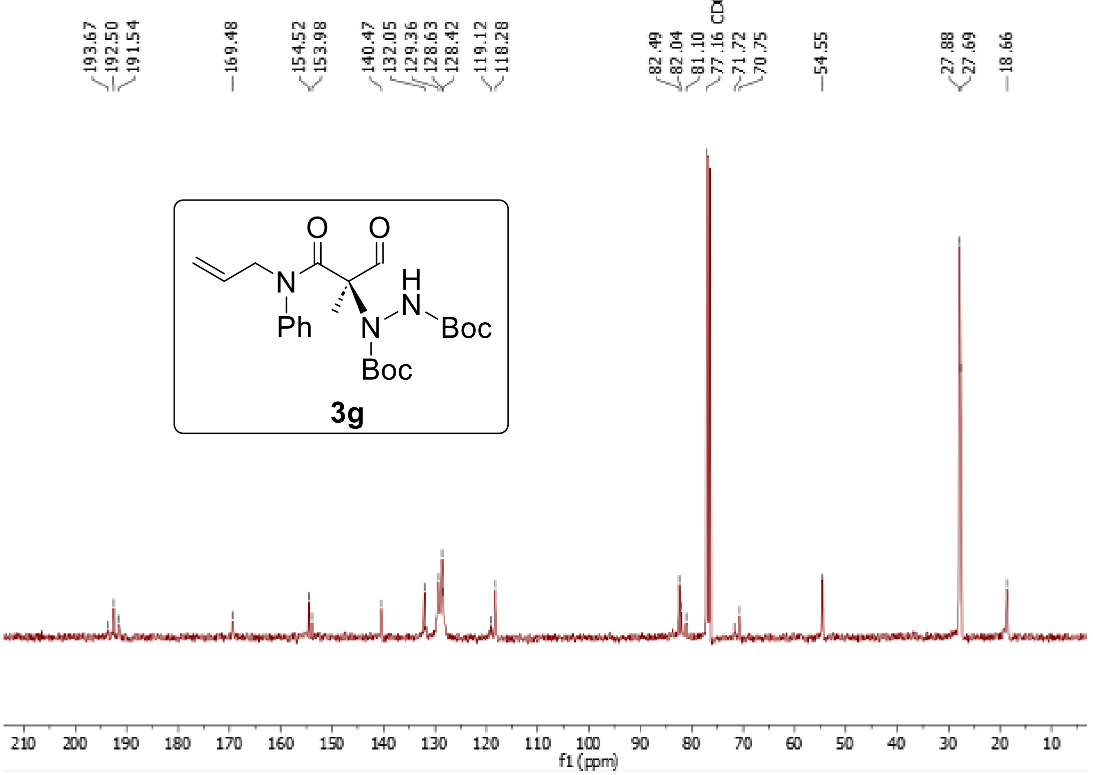

300 MHz

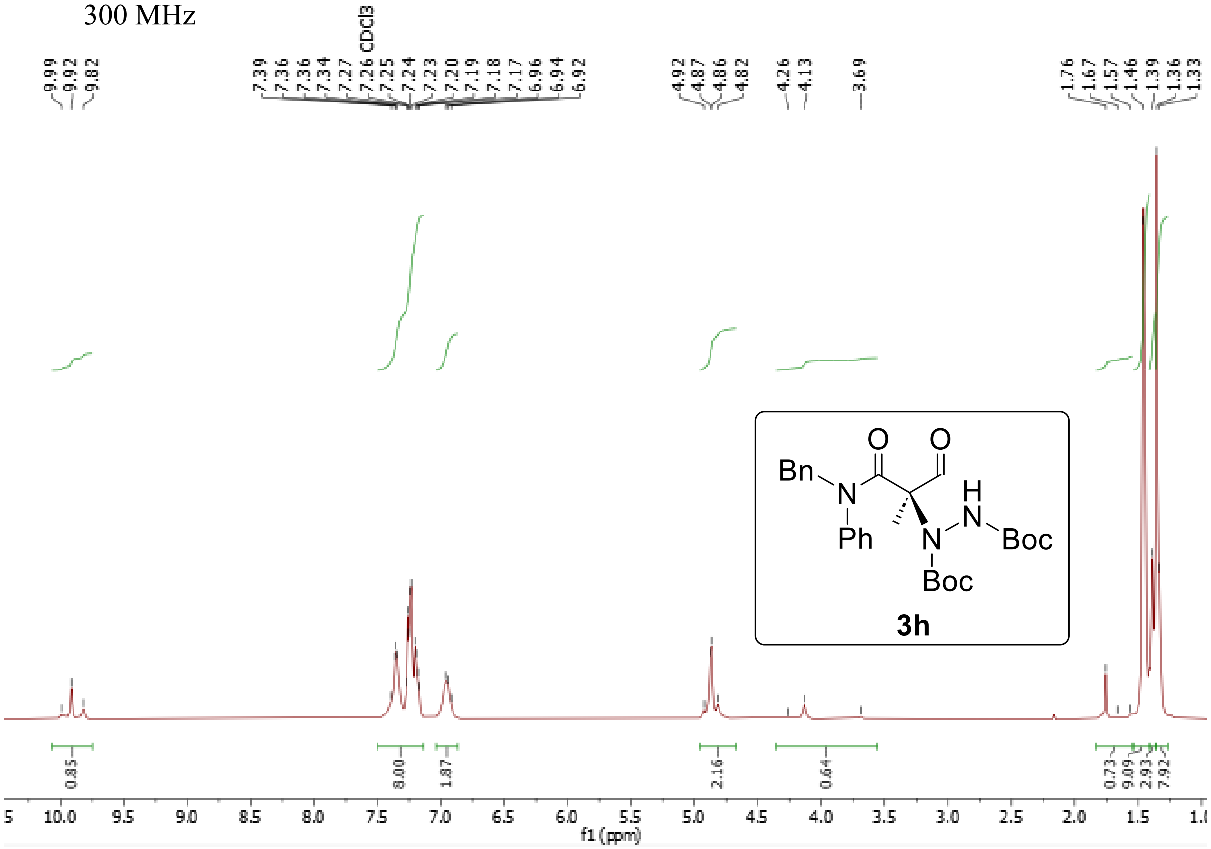

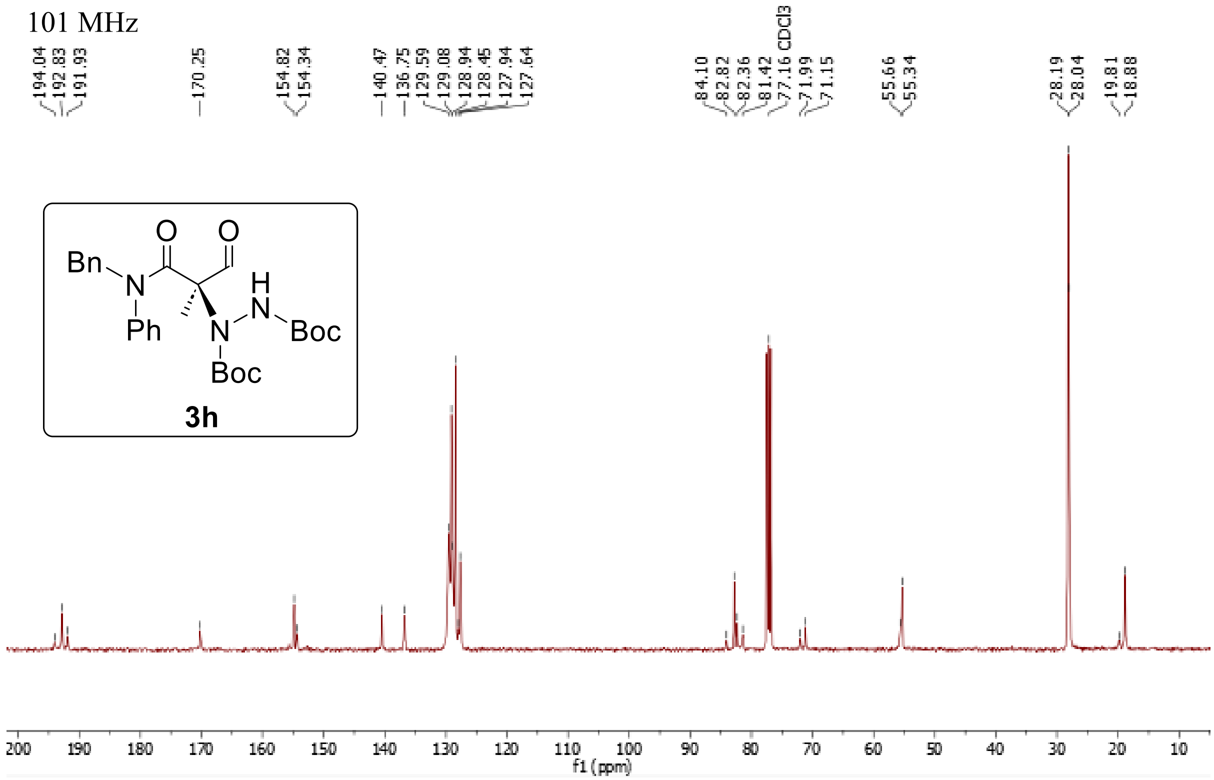
101 MHz
194.04
192.83
191.93
170.25
154.82
154.34
140.47
136.75
129.59
129.08
128.94
128.45
127.94
127.64
84.10
82.82
82.36
81.42
77.16 CDCl3
71.99
71.15
55.66
55.34
28.19
28.04
19.81
18.88
Bn
N
Ph
O
O
H
N
N
Boc
Boc
3h
f1 (ppm)

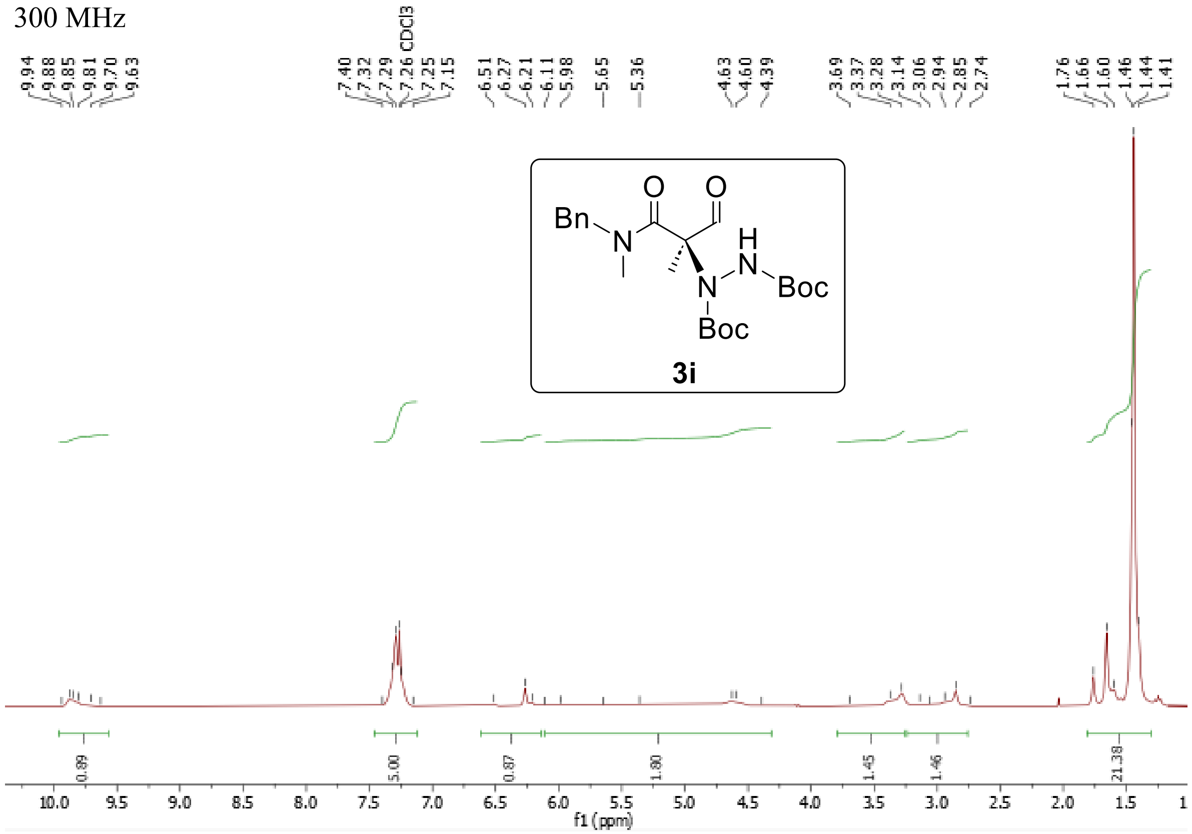
300 MHz
9.94
9.88
9.85
9.81
9.70
9.63
7.40
7.32
7.29
7.26 CDCl3
7.25
7.15
6.51
6.27
6.21
6.11
5.98
5.65
5.36
4.63
4.60
4.39
3.69
3.37
3.28
3.14
3.06
2.94
2.85
2.74
1.76
1.66
1.60
1.46
1.44
1.41
Bn
N
O
O
H
N
N
Boc
Boc
3i
0.89
5.00
0.87
1.80
1.45
1.46
21.38
f1 (ppm)

151 MHz

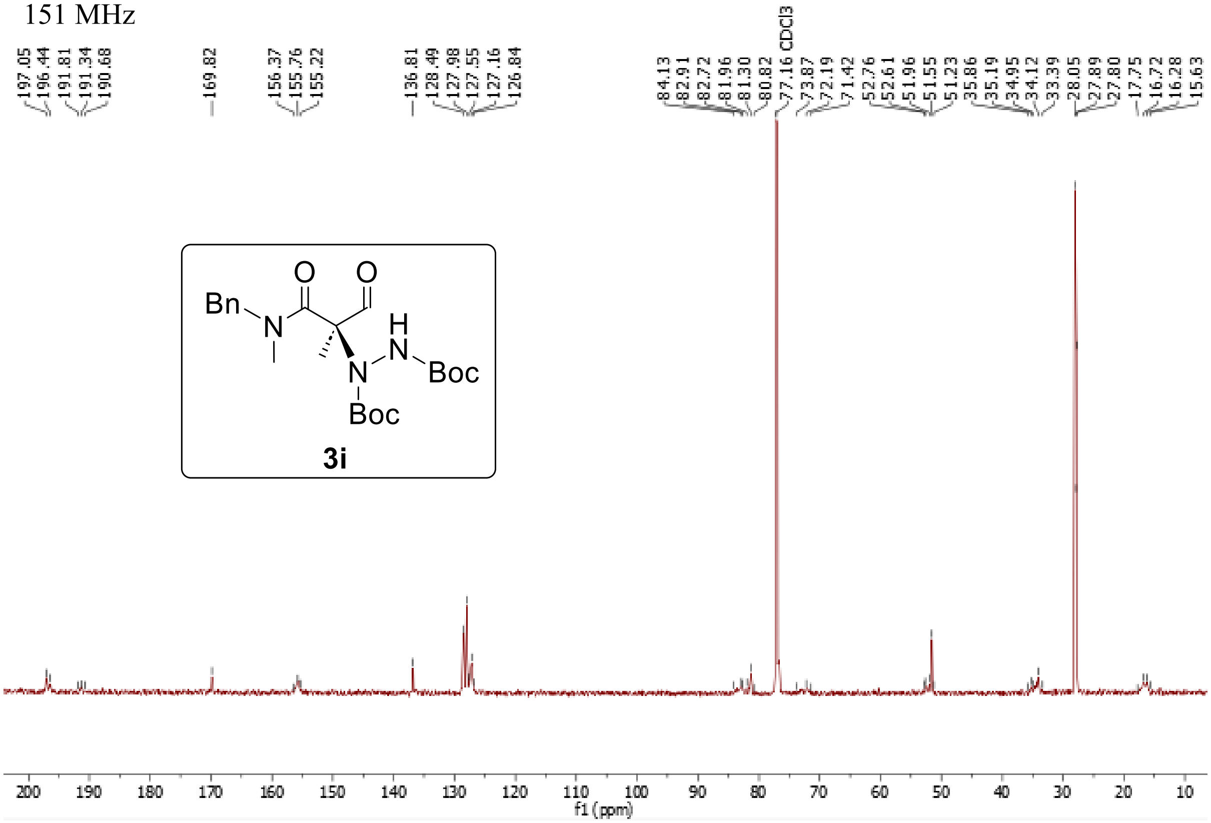

400 MHz

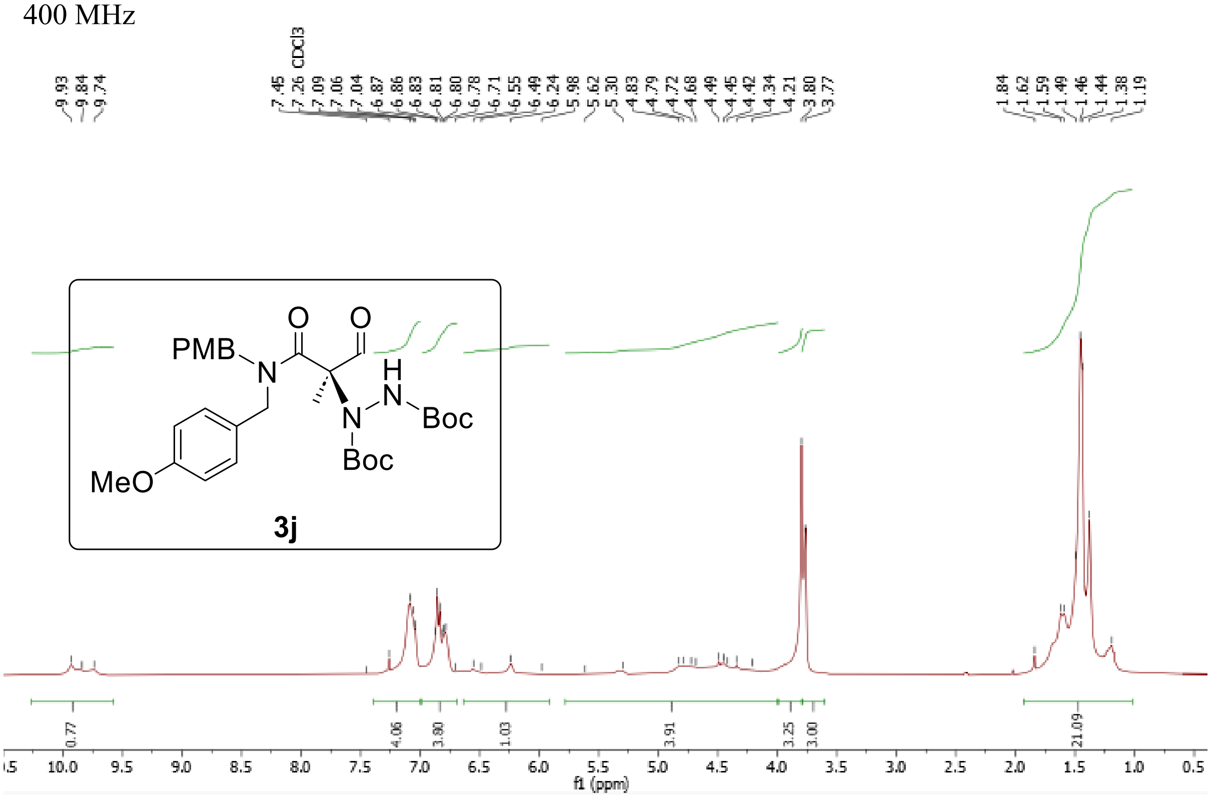

101 MHz

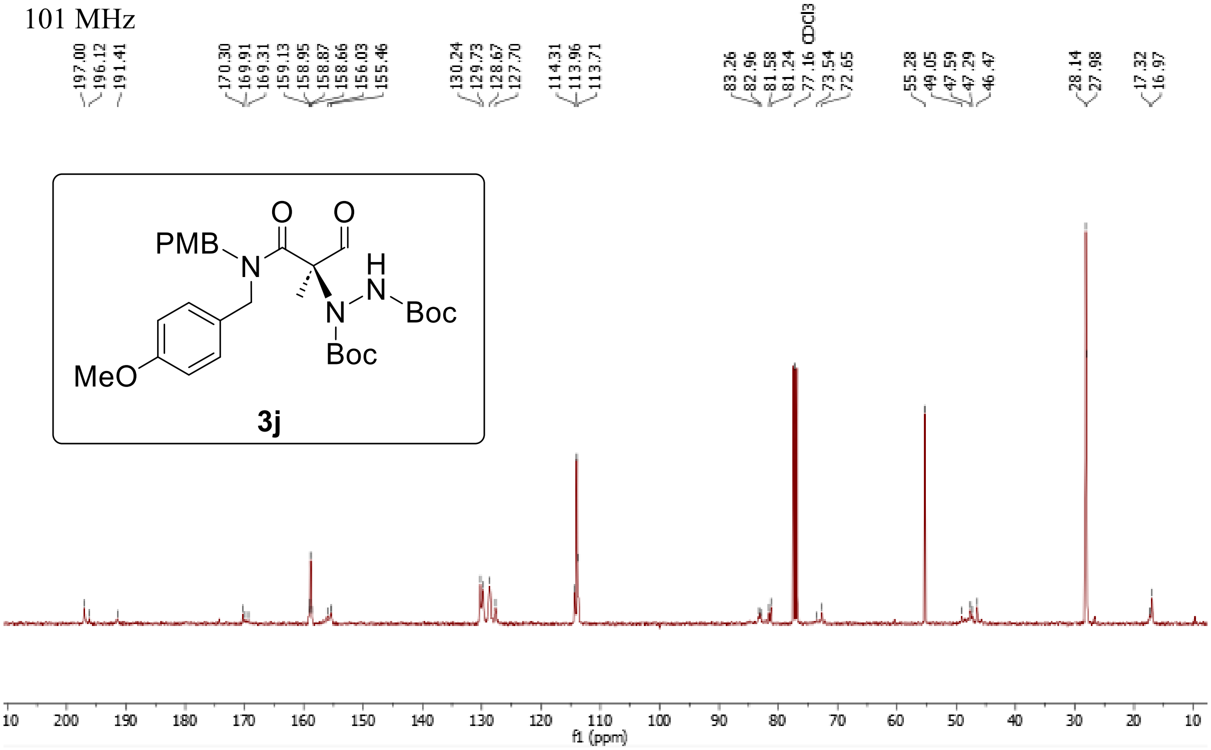

300 MHz

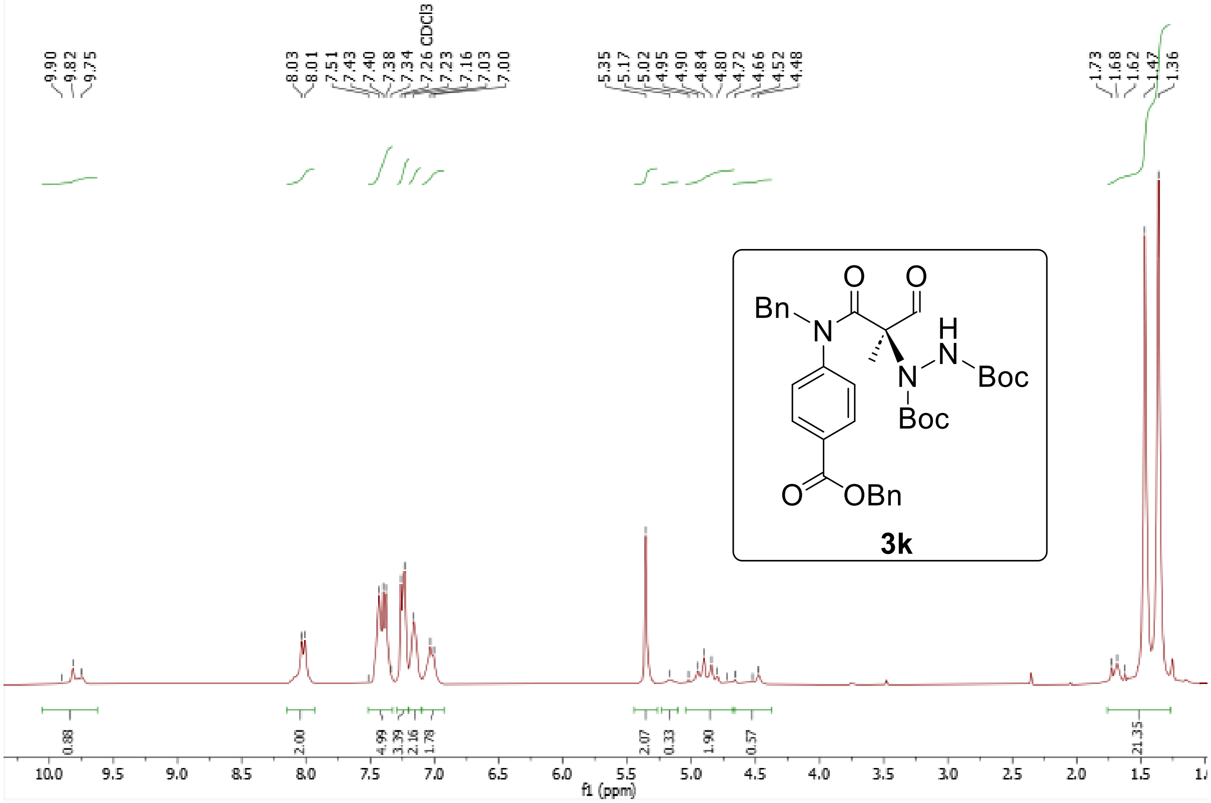

101 MHz

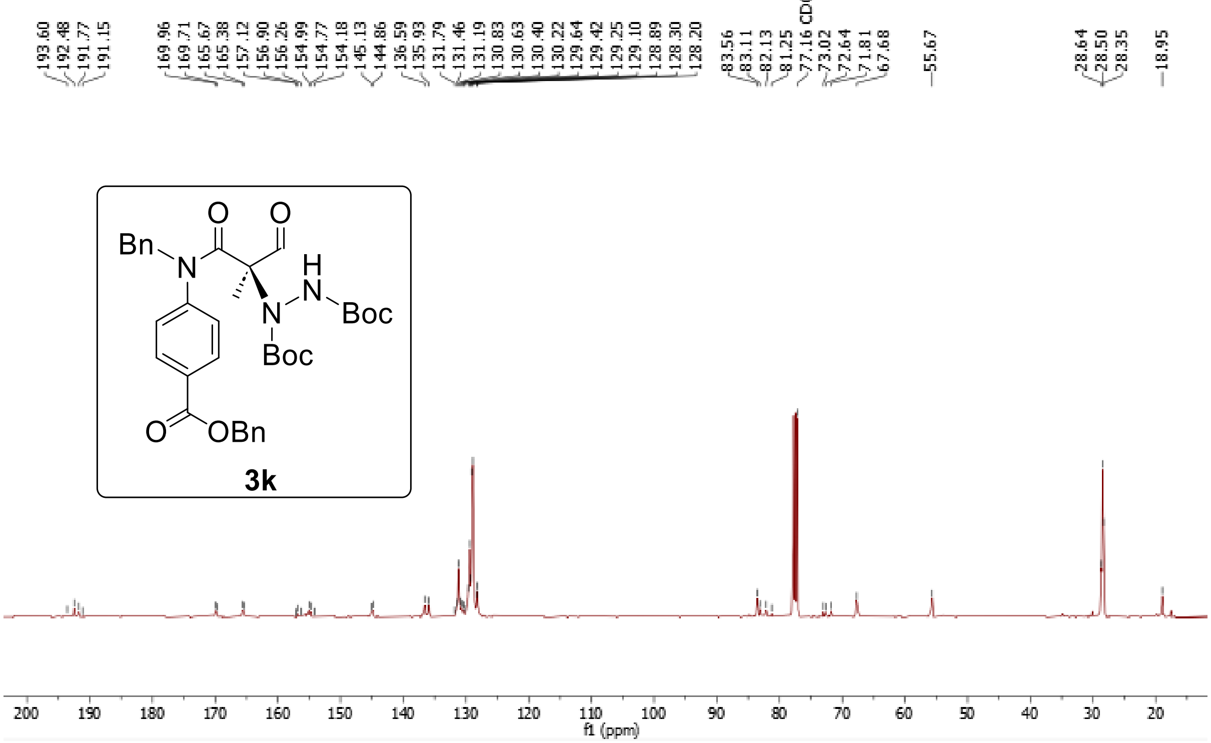

300 MHz

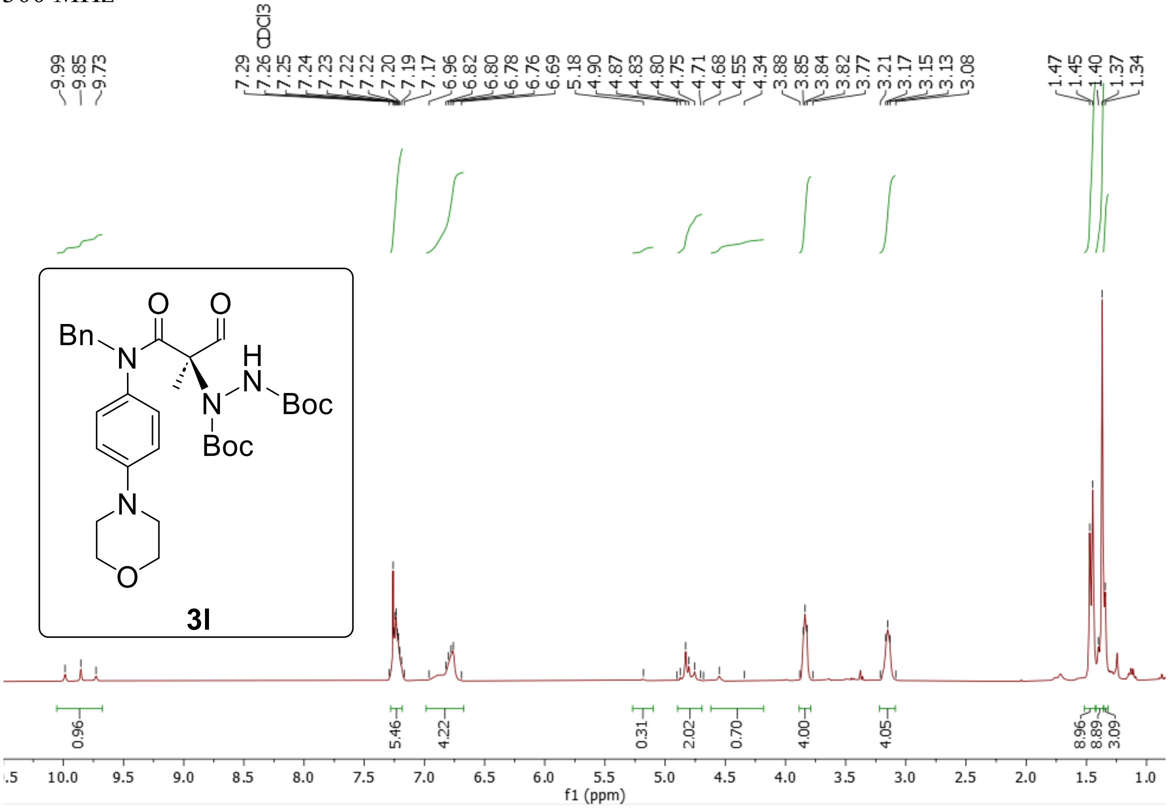

101 MHz

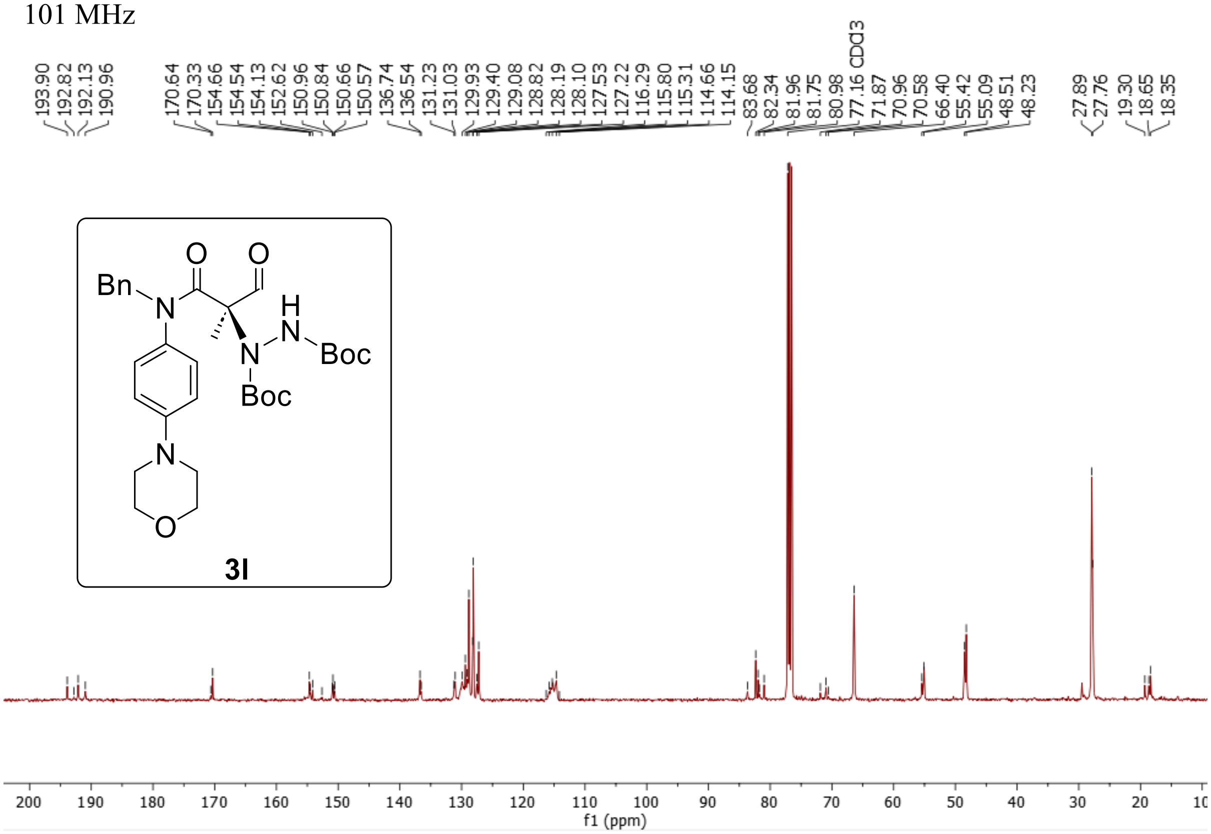

300 MHz

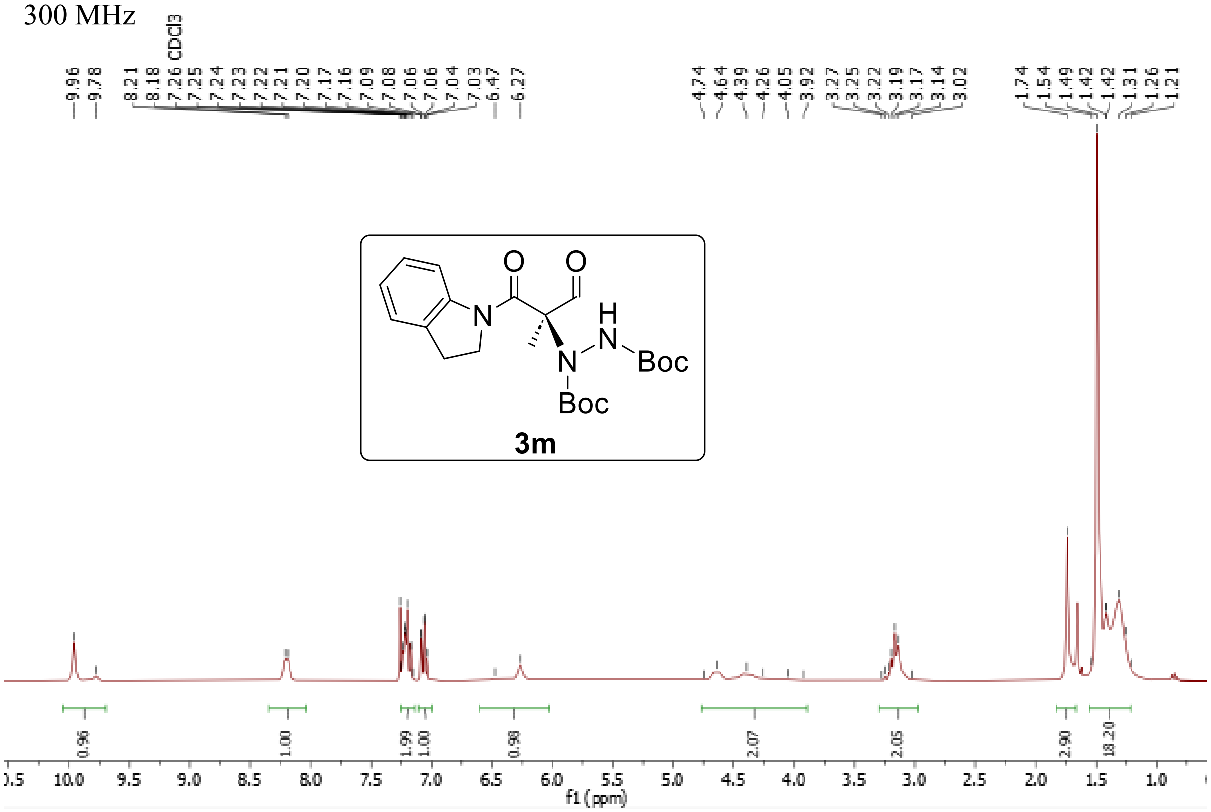

101 MHz

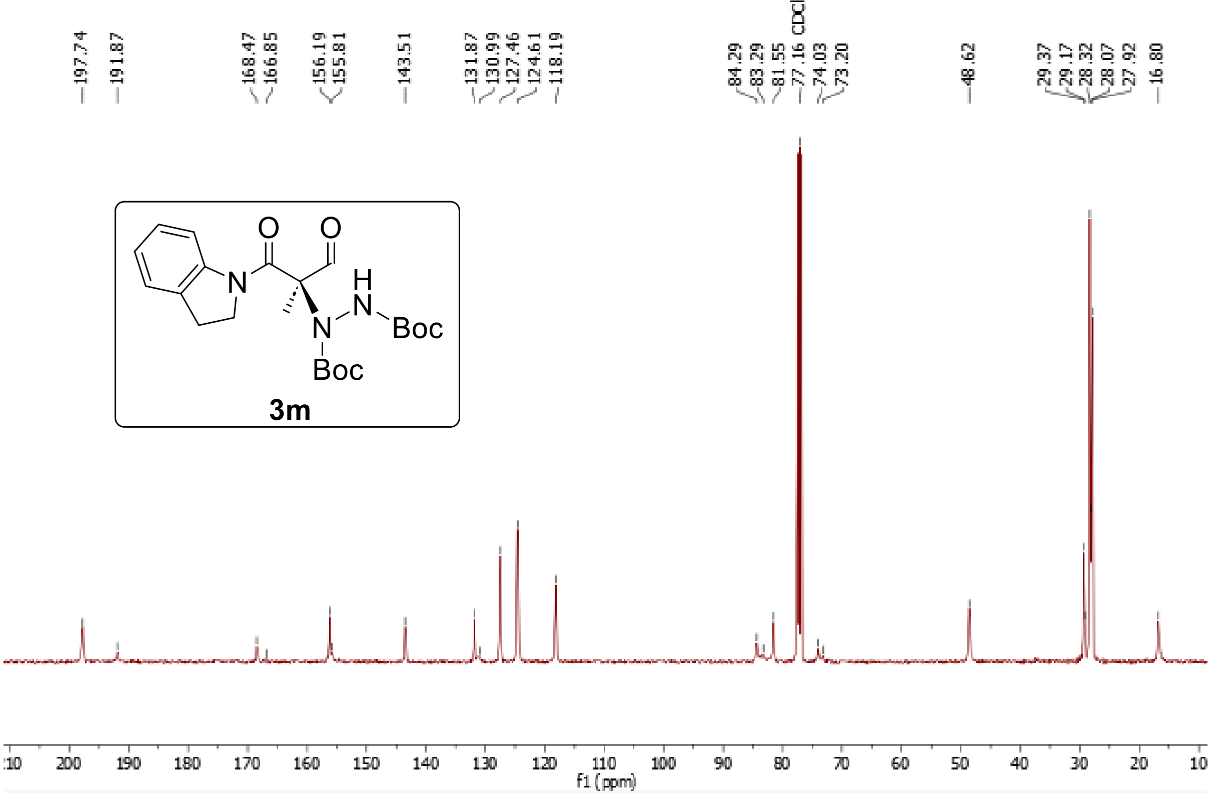

300 MHz

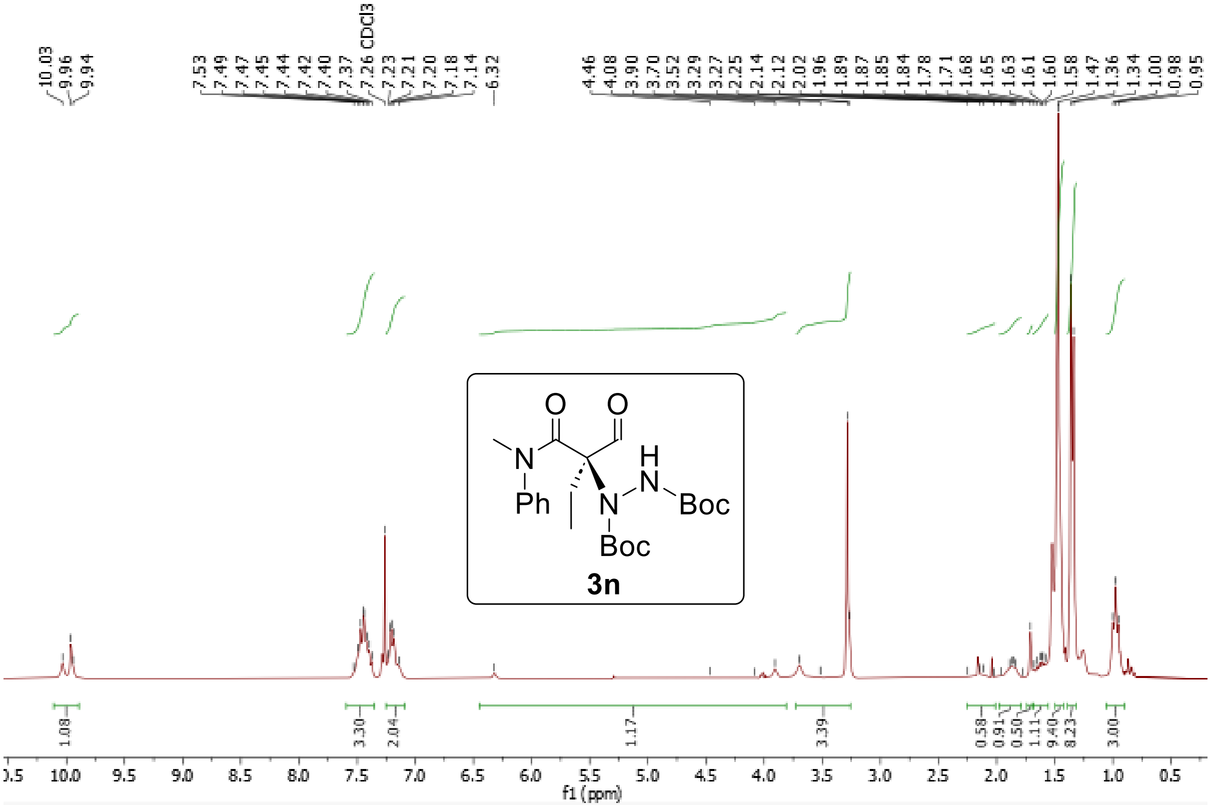

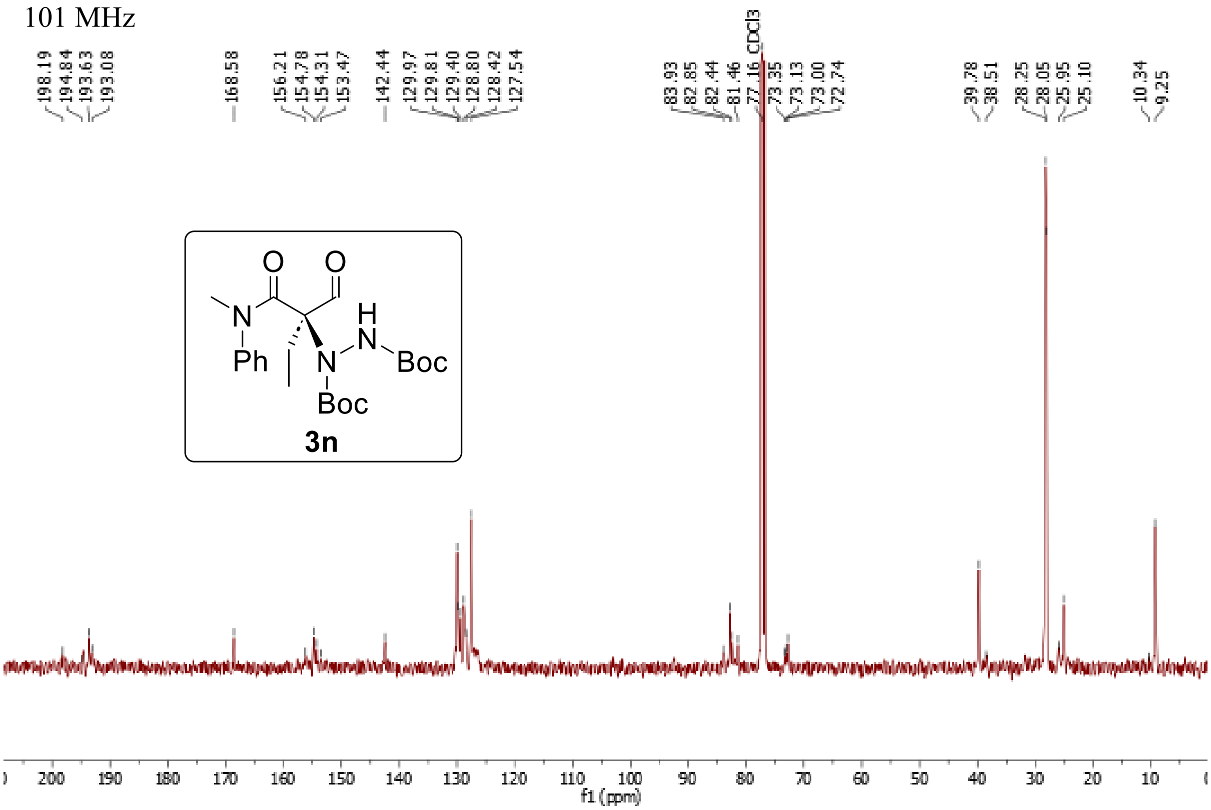
101 MHz
198.19
194.84
193.63
193.08
168.58
156.21
154.78
154.31
153.47
142.44
129.97
129.81
129.40
128.80
128.42
127.54
83.93
82.85
82.44
81.46
77.16 CDCl3
73.35
73.13
73.00
72.74
39.78
38.51
28.25
28.05
25.95
25.10
10.34
9.25
O
O
N
Ph
H
N
N
Boc
Boc
3n
200 190 180 170 160 150 140 130 120 110 100 90 80 70 60 50 40 30 20 10
f1 (ppm)

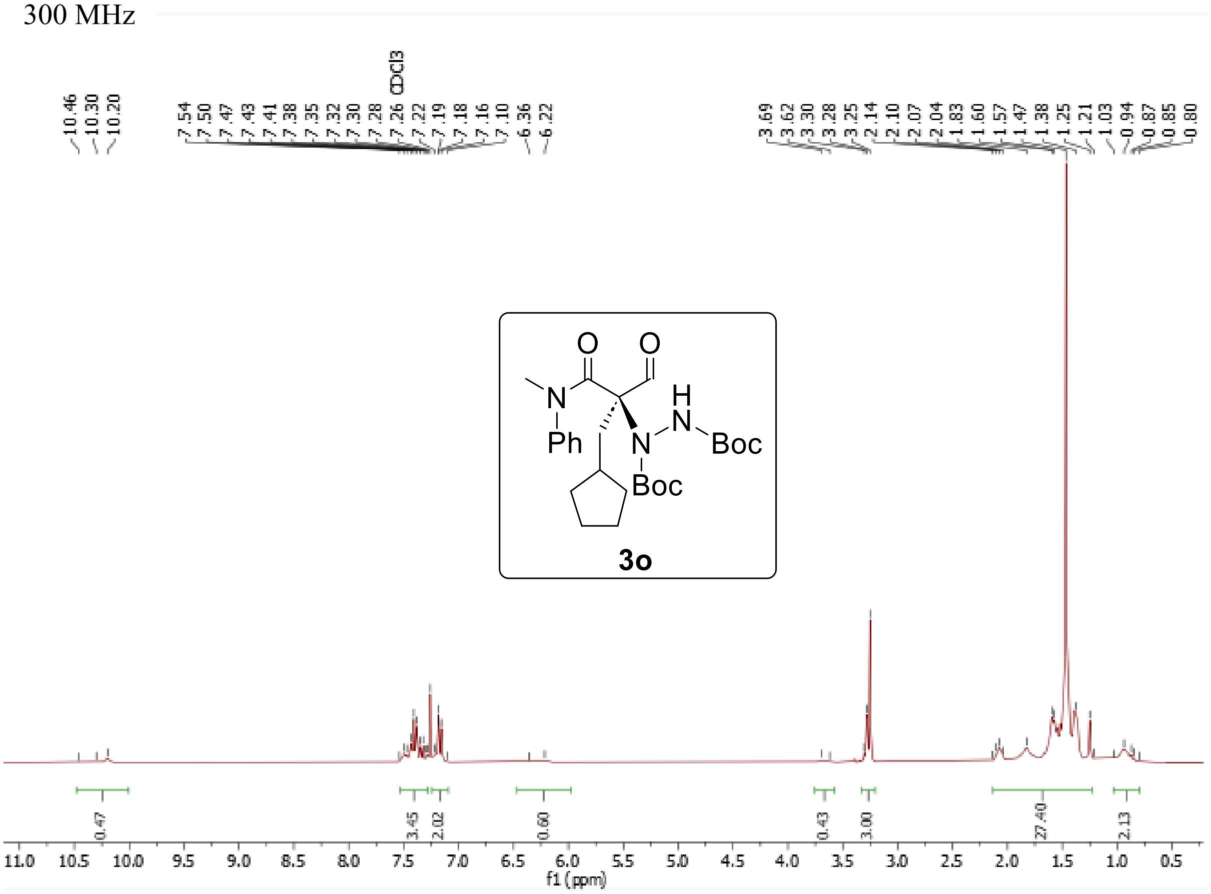
300 MHz
10.46
10.30
10.20
7.54
7.50
7.47
7.43
7.41
7.38
7.35
7.32
7.30
7.28
7.26 CDCl3
7.22
7.19
7.18
7.16
7.10
6.36
6.22
3.69
3.62
3.30
3.28
3.25
2.14
2.10
2.07
2.04
1.83
1.60
1.57
1.47
1.38
1.25
1.21
1.03
0.94
0.87
0.85
0.80
O
O
N
Ph
H
N
N
Boc
Boc
3o
0.47
3.45
2.02
0.60
0.43
3.00
27.40
2.13
11.0 10.5 10.0 9.5 9.0 8.5 8.0 7.5 7.0 6.5 6.0 5.5 5.0 4.5 4.0 3.5 3.0 2.5 2.0 1.5 1.0 0.5
f1 (ppm)

101 MHz

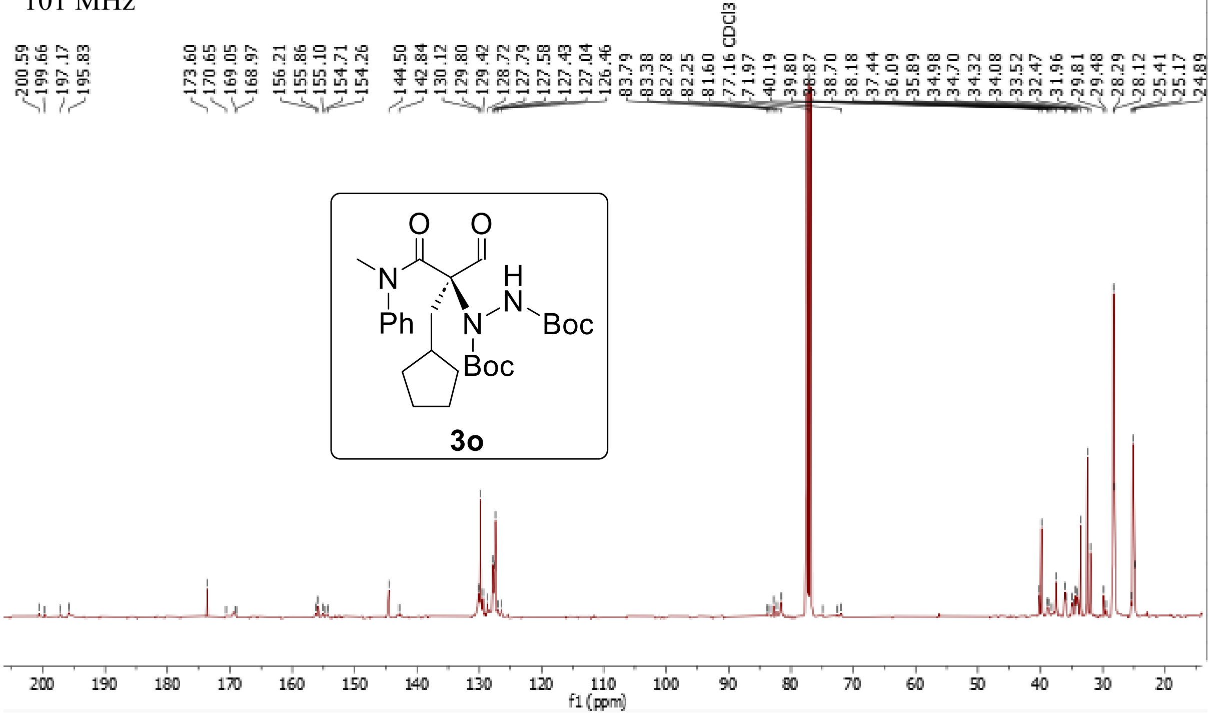

400 MHz

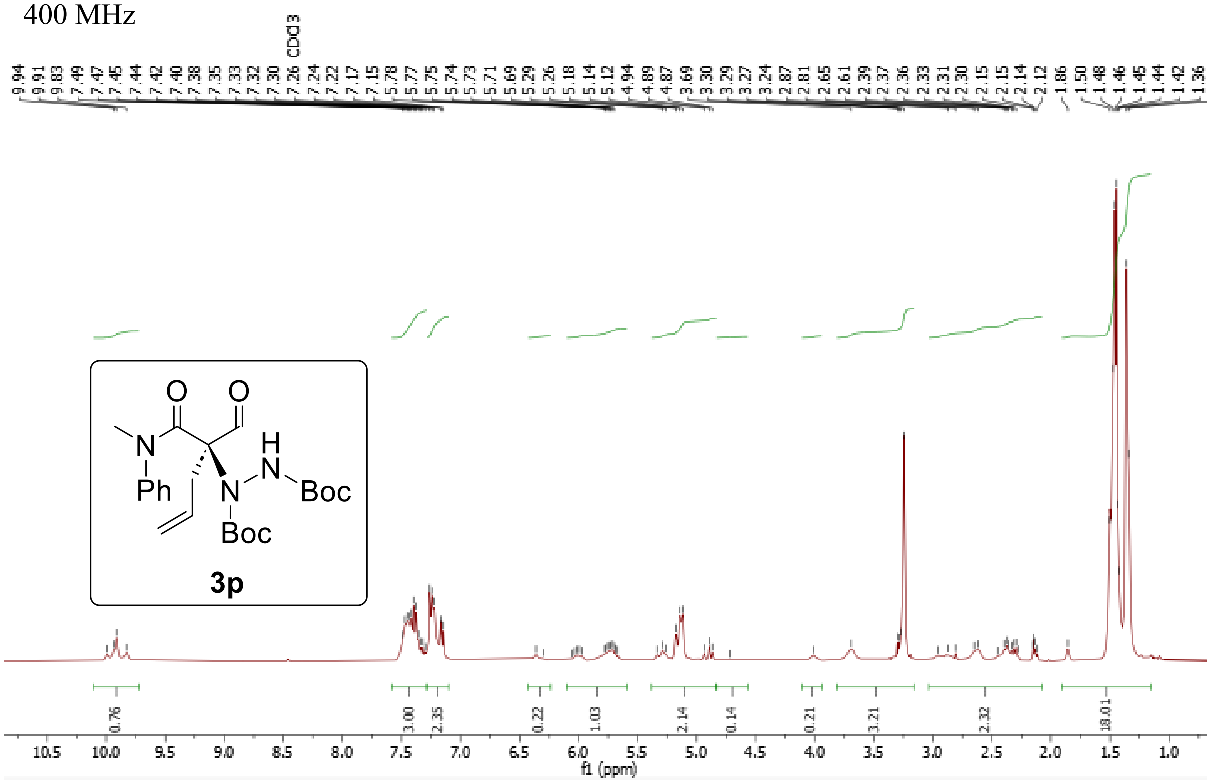

101 MHz

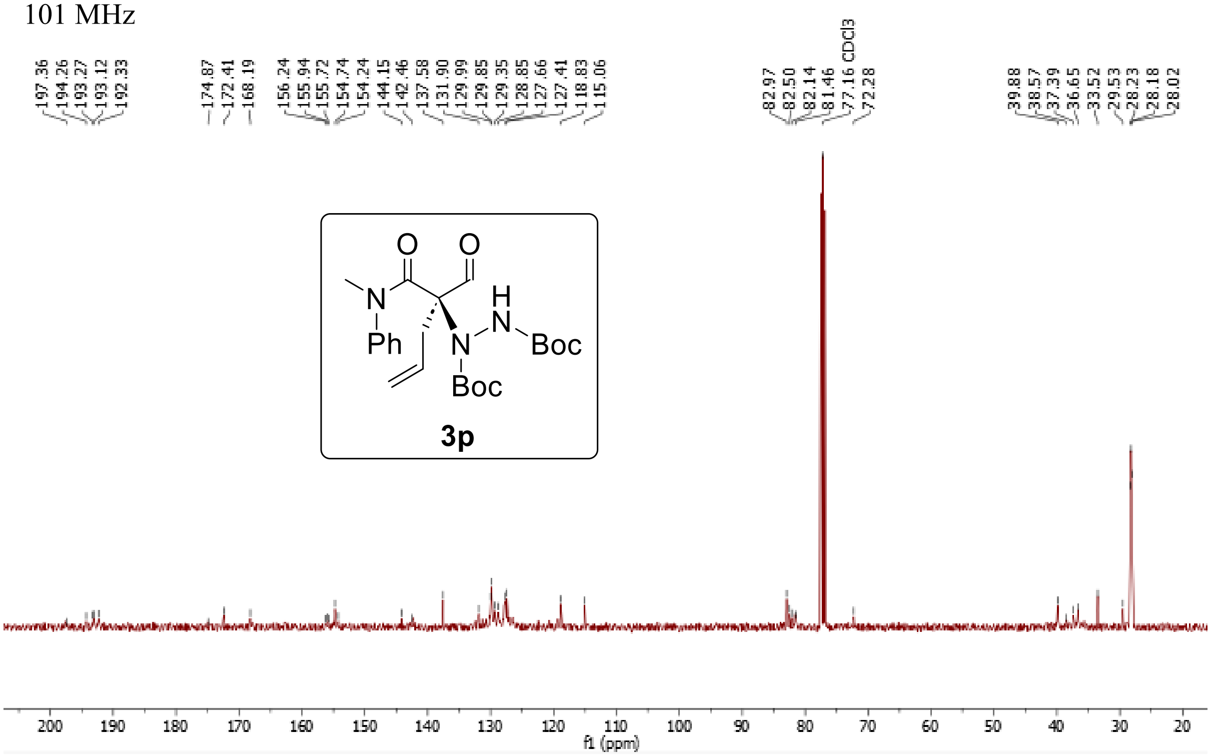

400 MHz

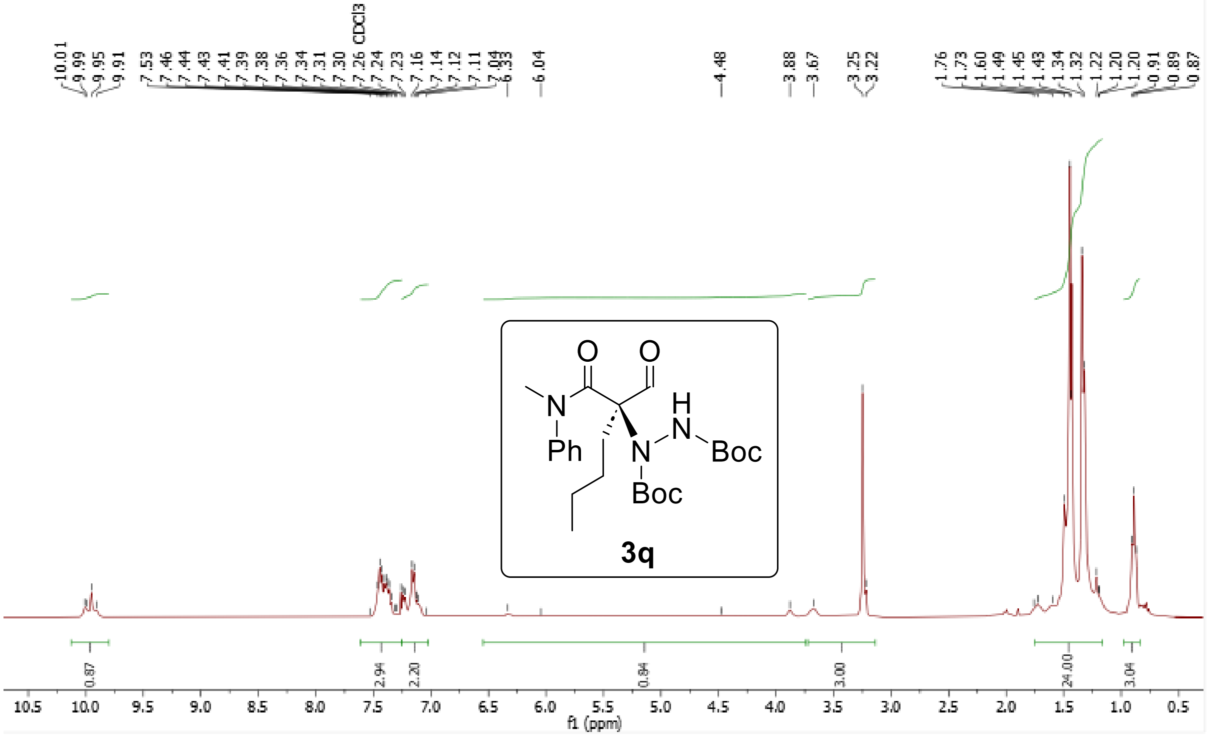

101 MHz

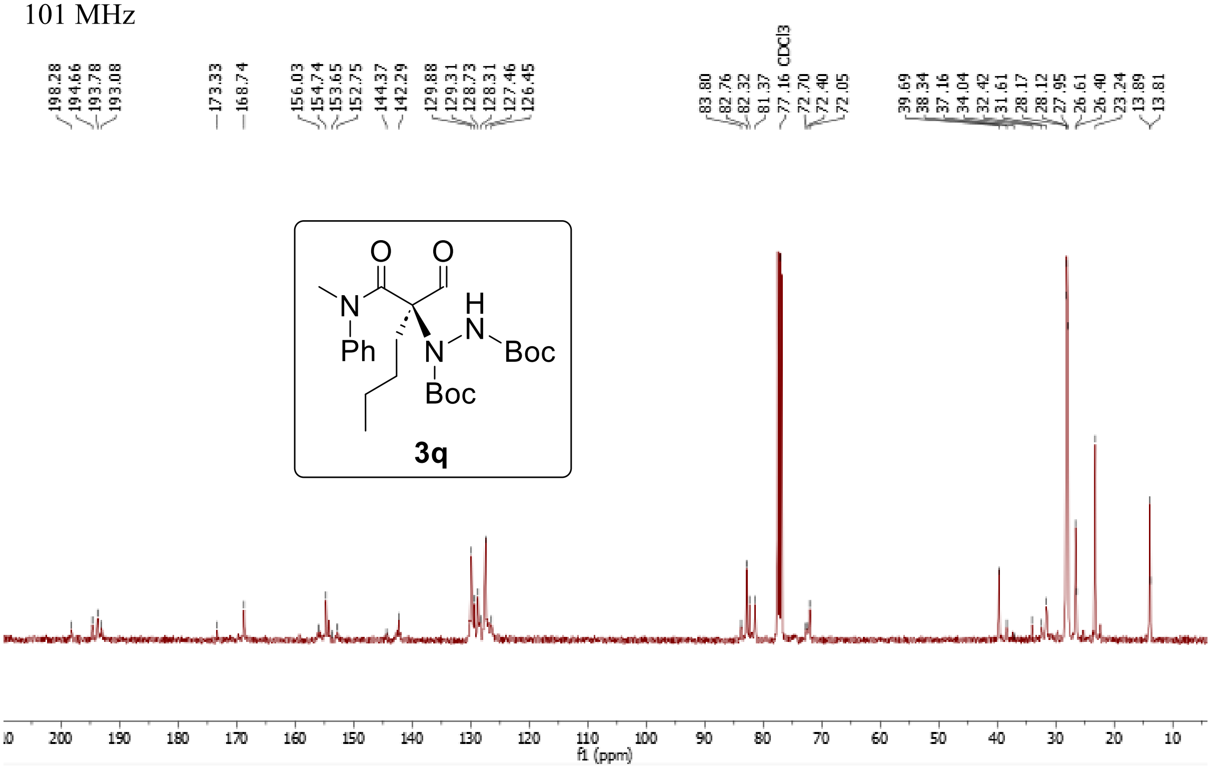

400 MHz

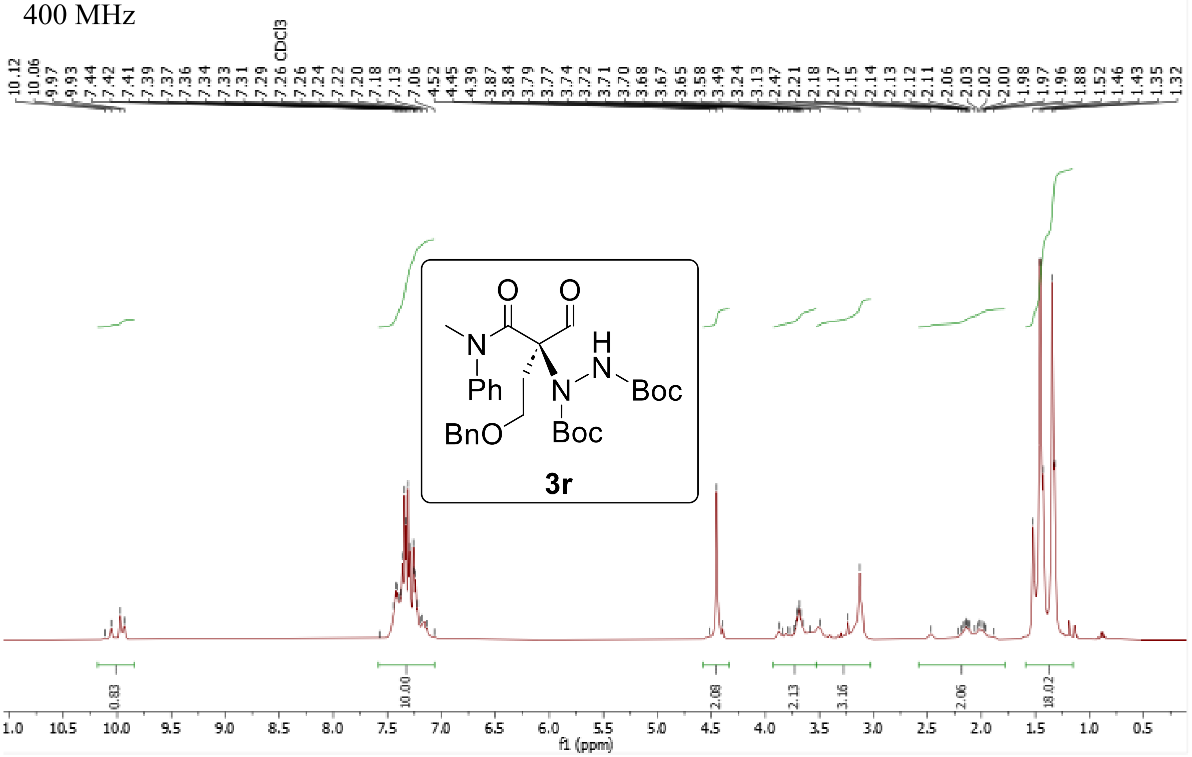

101 MHz

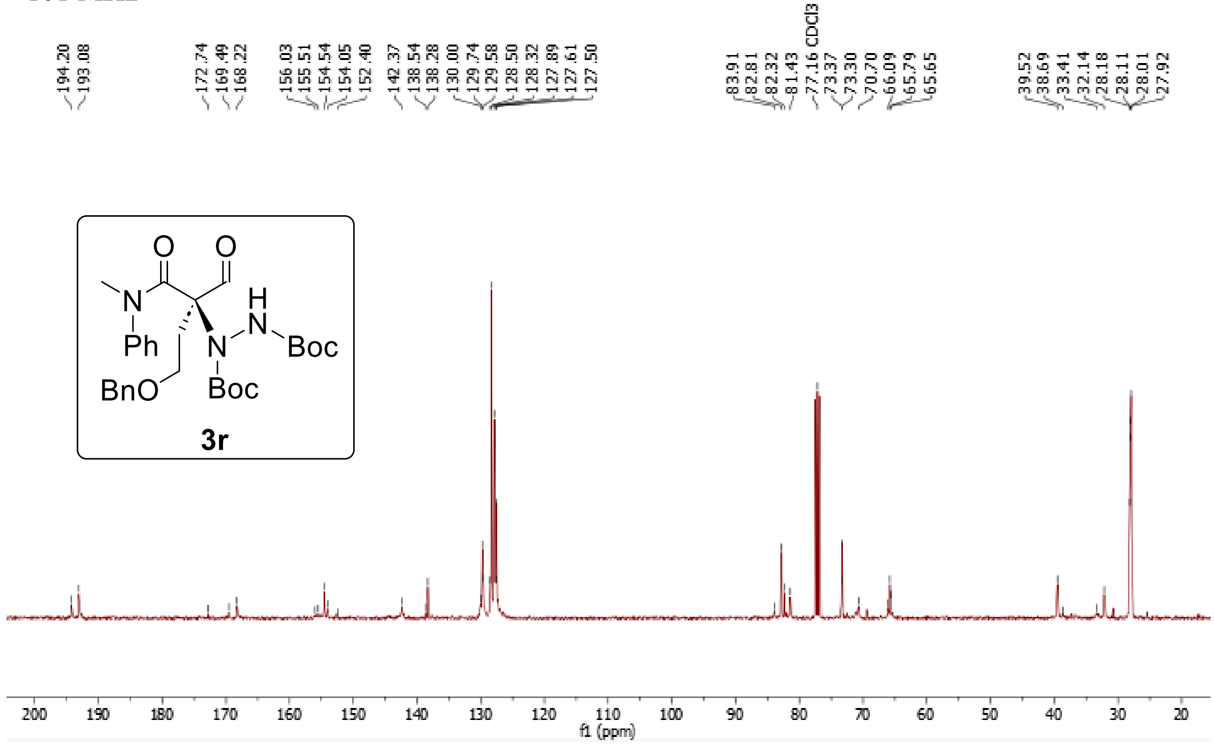

300 MHz

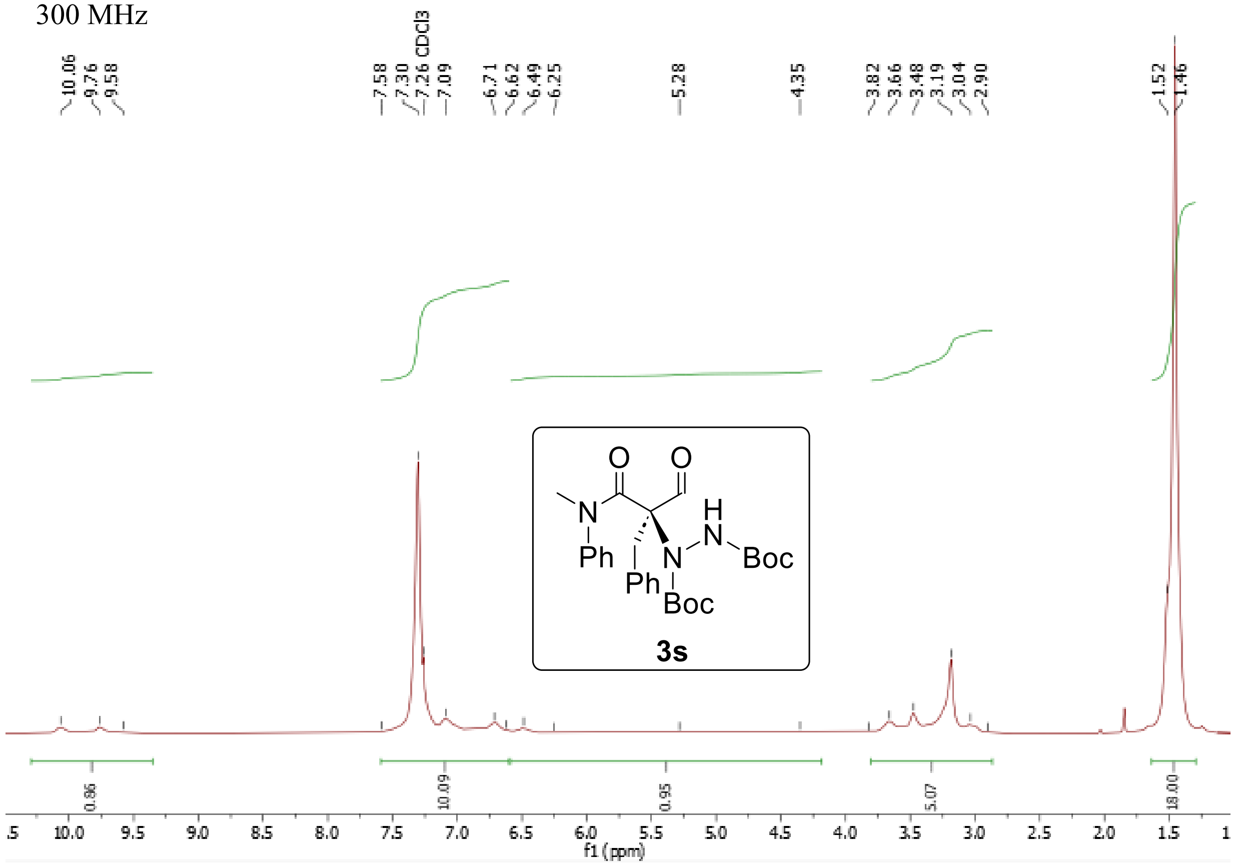

VT [1]H Spectrum for 3s at 353K (600 MHz)

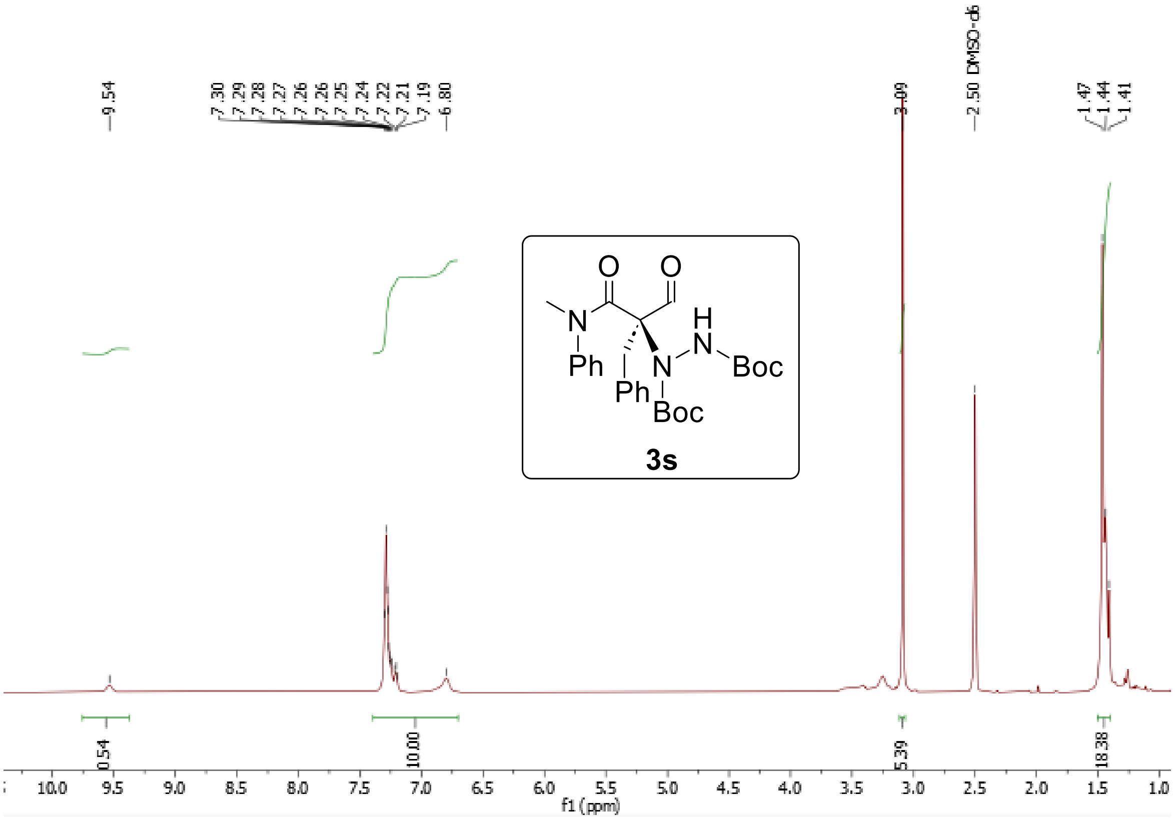

101 MHz

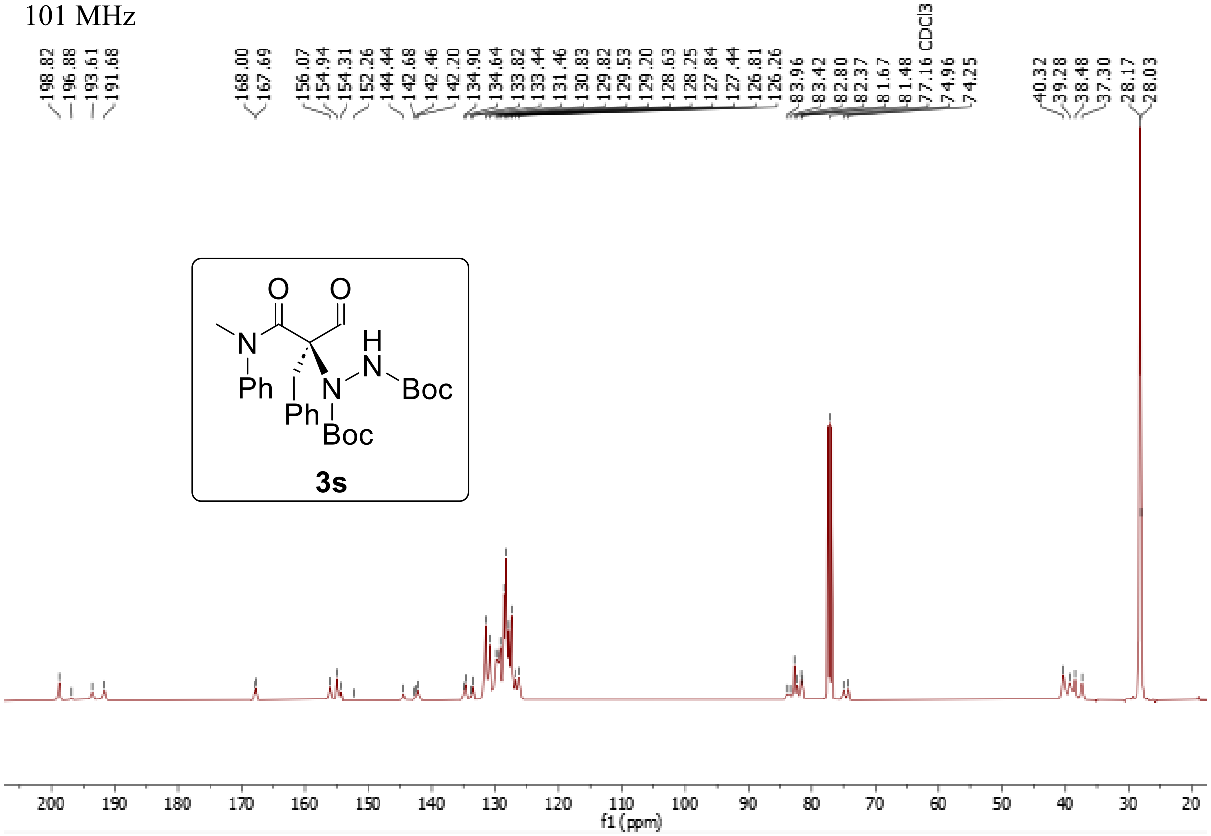

400 MHz

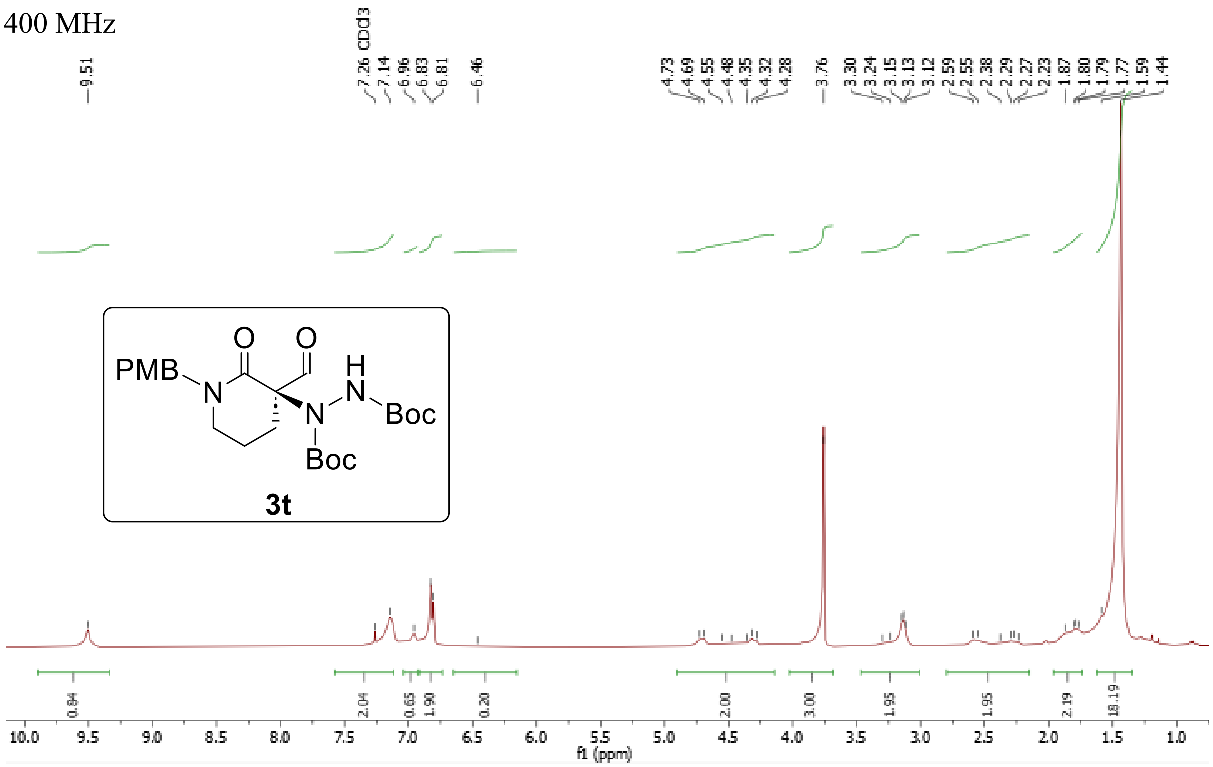

101 MHz

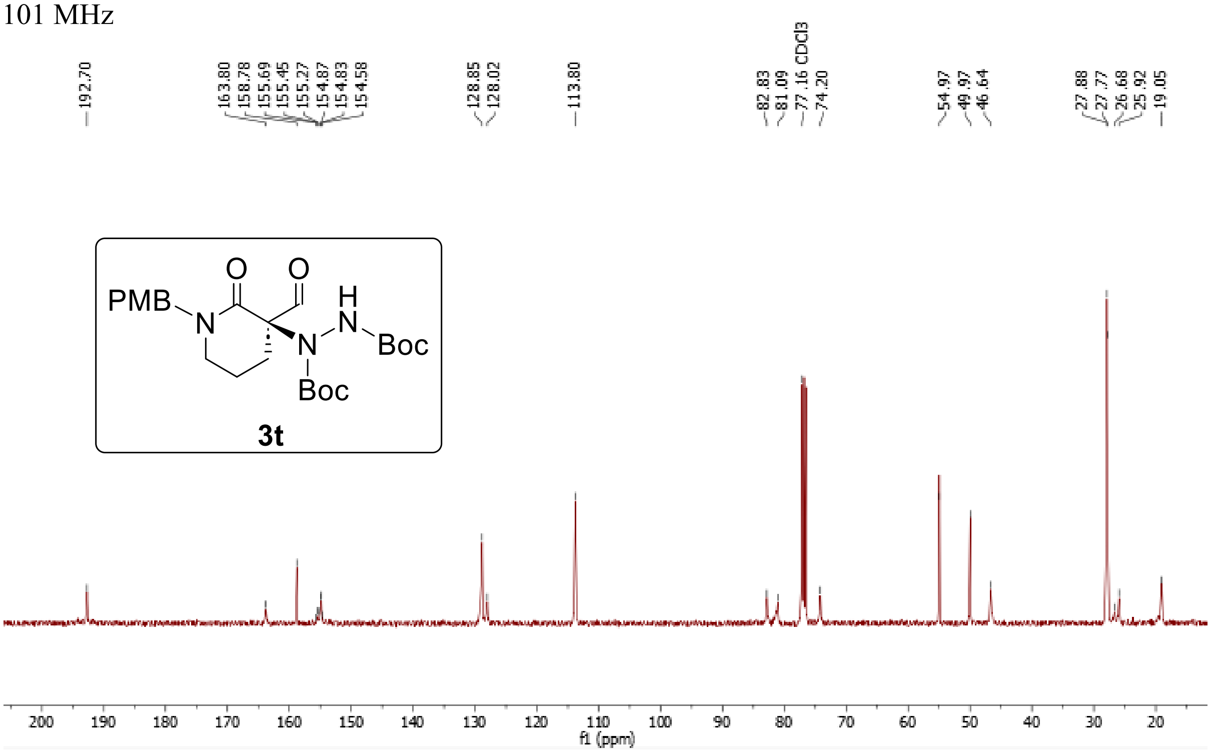

400 MHz

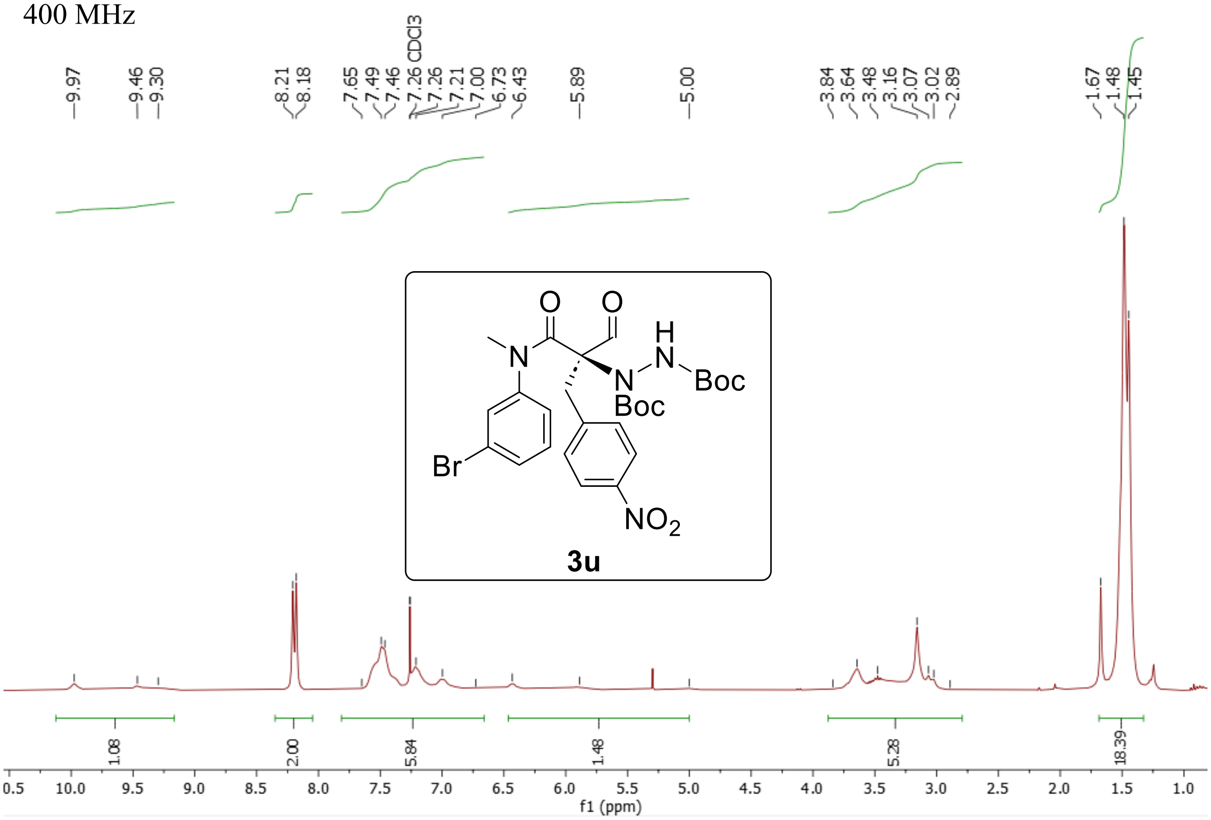

101 MHz

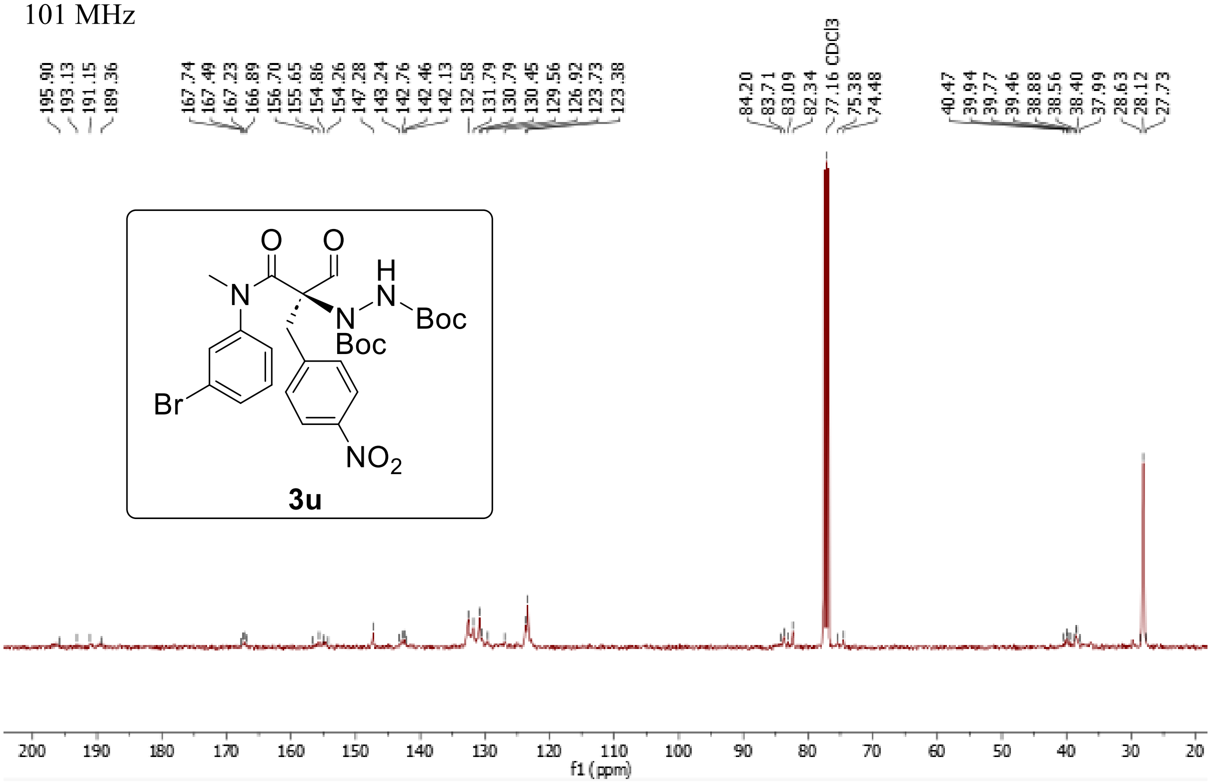

(iv) Oxazolidinone Hydrazides 4a, 4c, 4j, 4n, 4o

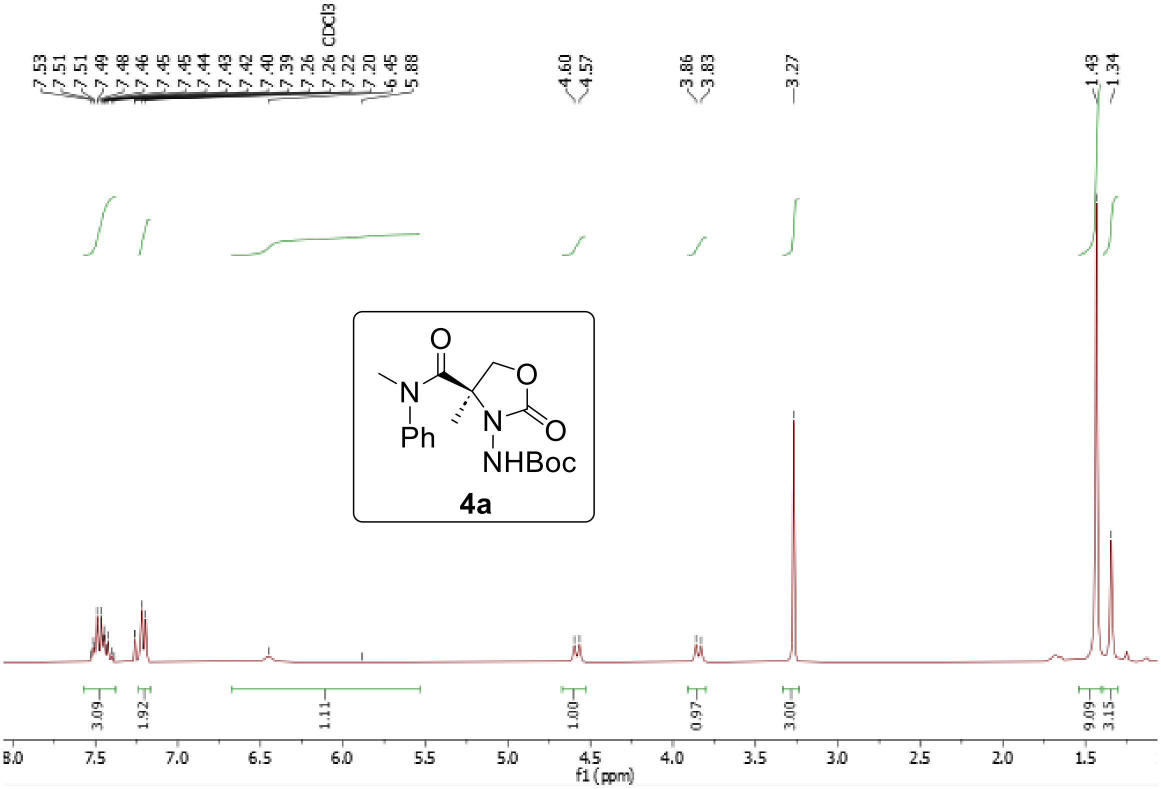

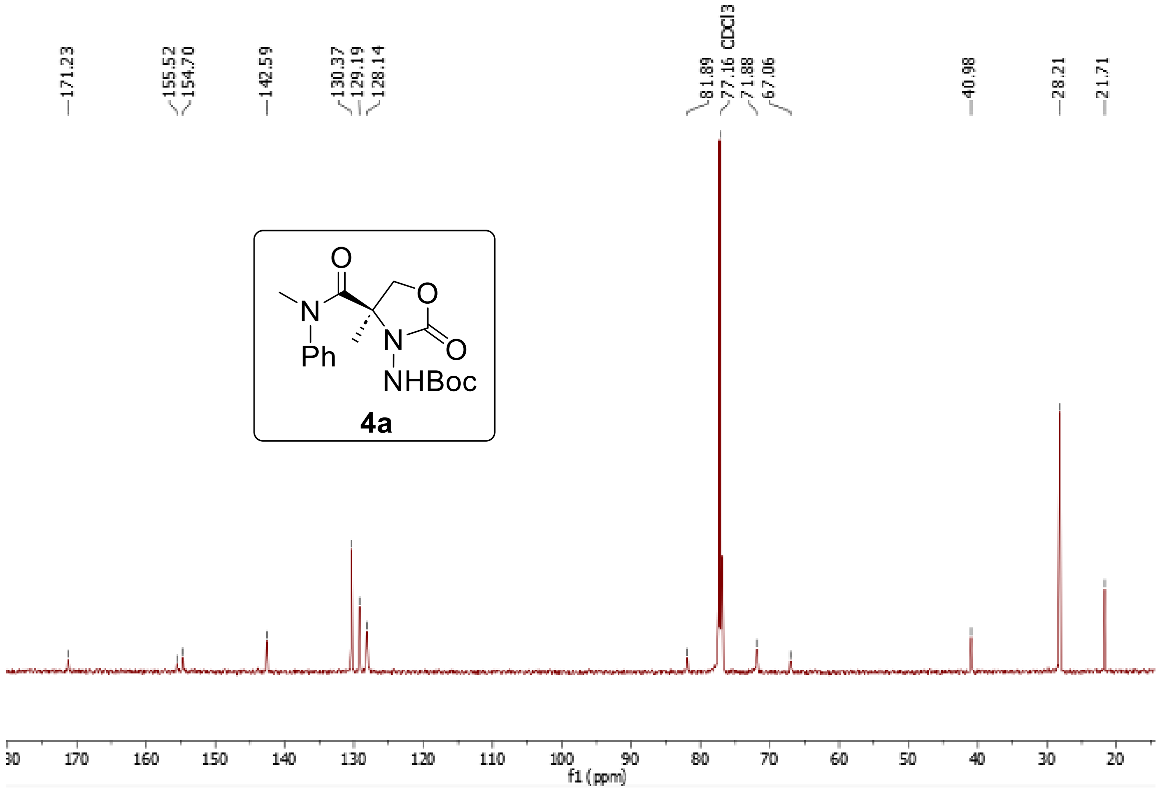

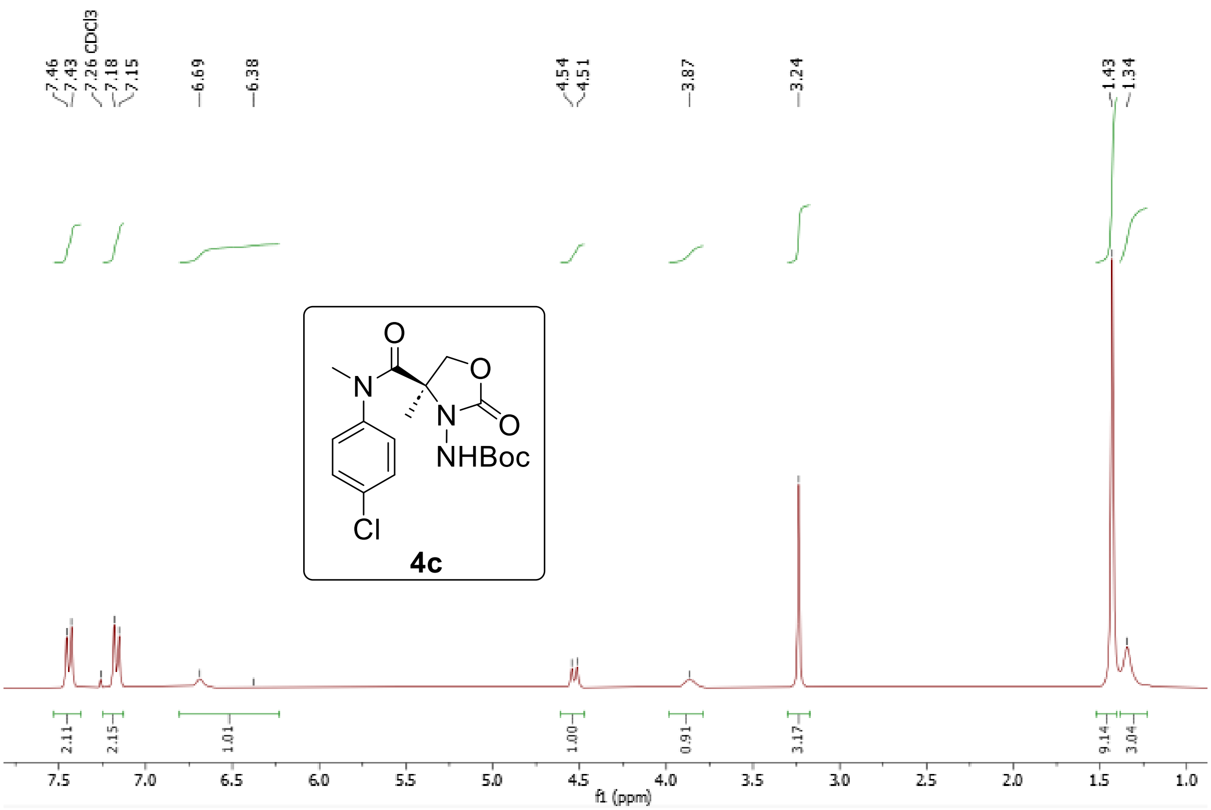

7.46
7.43
7.26 CDCl3
7.18
7.15
6.69
6.38
4.54
4.51
3.87
3.24
1.43
1.34
4c
NHBoc
Cl
2.11
2.15
1.01
1.00
0.91
3.17
9.14
3.04
f1 (ppm)

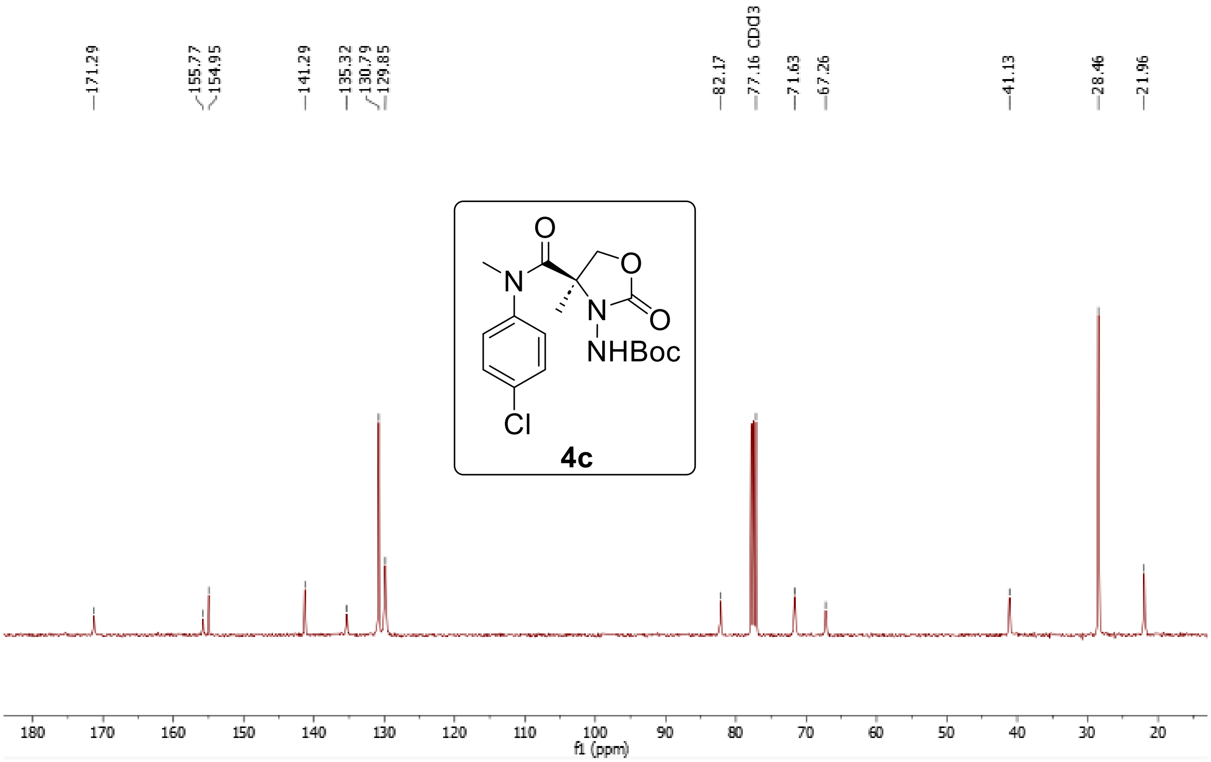

171.29
155.77
154.95
141.29
135.32
130.79
129.85
82.17
77.16 CDCl3
71.63
67.26
41.13
28.46
21.96
4c
NHBoc
Cl
f1 (ppm)

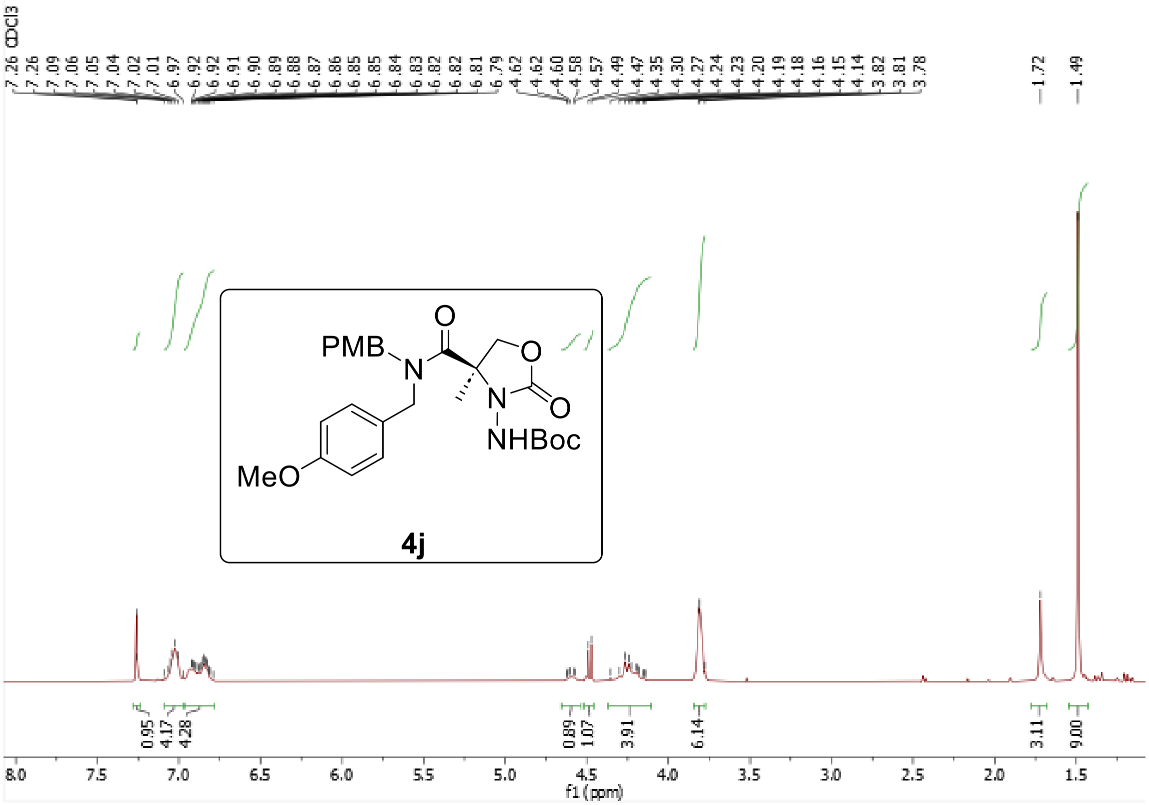

7.26 CDCl3
7.26
7.09
7.06
7.05
7.04
7.02
7.01
6.97
6.92
6.92
6.91
6.90
6.89
6.88
6.87
6.86
6.85
6.85
6.84
6.83
6.82
6.82
6.81
6.79
4.62
4.60
4.58
4.57
4.49
4.47
4.35
4.30
4.27
4.24
4.23
4.20
4.19
4.18
4.16
4.15
4.14
3.82
3.81
3.78
1.72
1.49
PMB
N
O
O
O
N
NHBoc
MeO
4j
0.95
4.17
4.28
0.89
1.07
3.91
6.14
3.11
9.00
8.0
7.5
7.0
6.5
6.0
5.5
5.0
4.5
4.0
3.5
3.0
2.5
2.0
1.5
f1 (ppm)

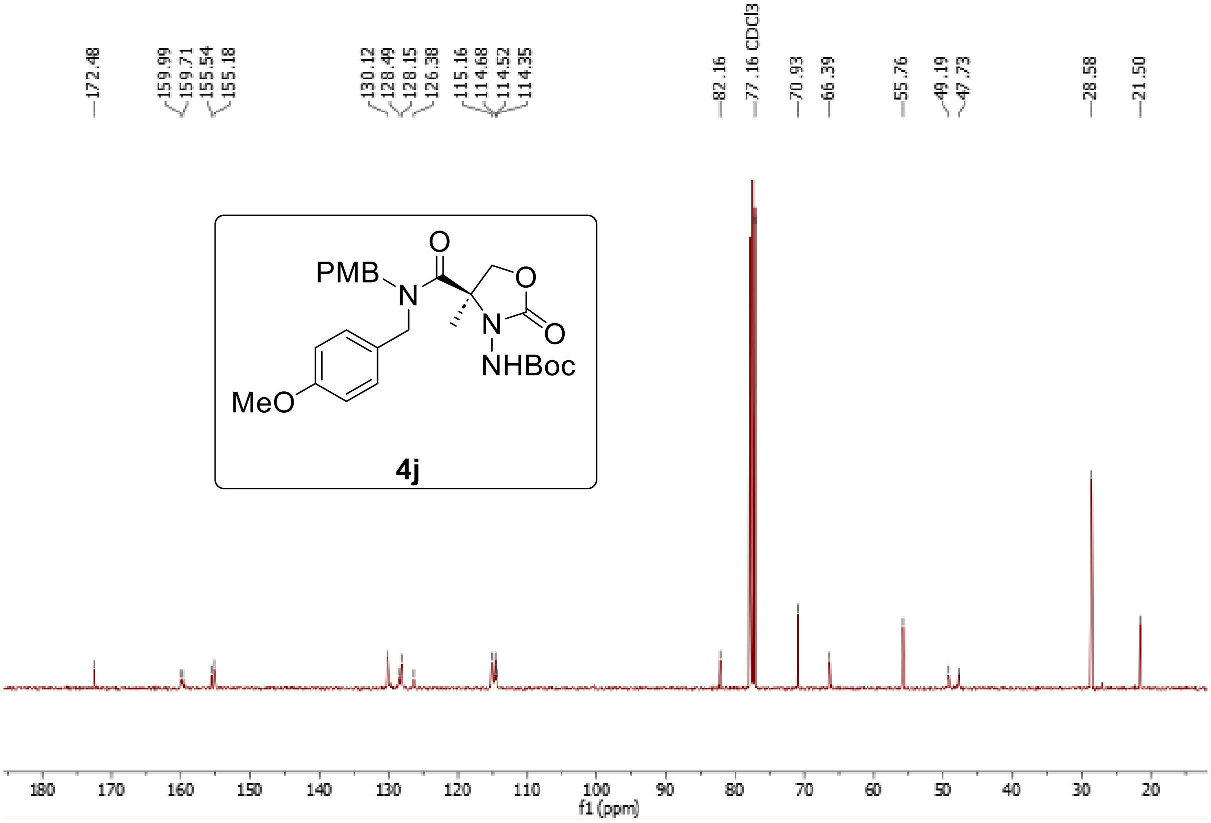

172.48
159.99
159.71
155.54
155.18
130.12
128.49
128.15
126.38
115.16
114.68
114.52
114.35
82.16
77.16 CDCl3
70.93
66.39
55.76
49.19
47.73
28.58
21.50
PMB
N
O
O
O
N
NHBoc
MeO
4j
180
170
160
150
140
130
120
110
100
90
80
70
60
50
40
30
20
f1 (ppm)

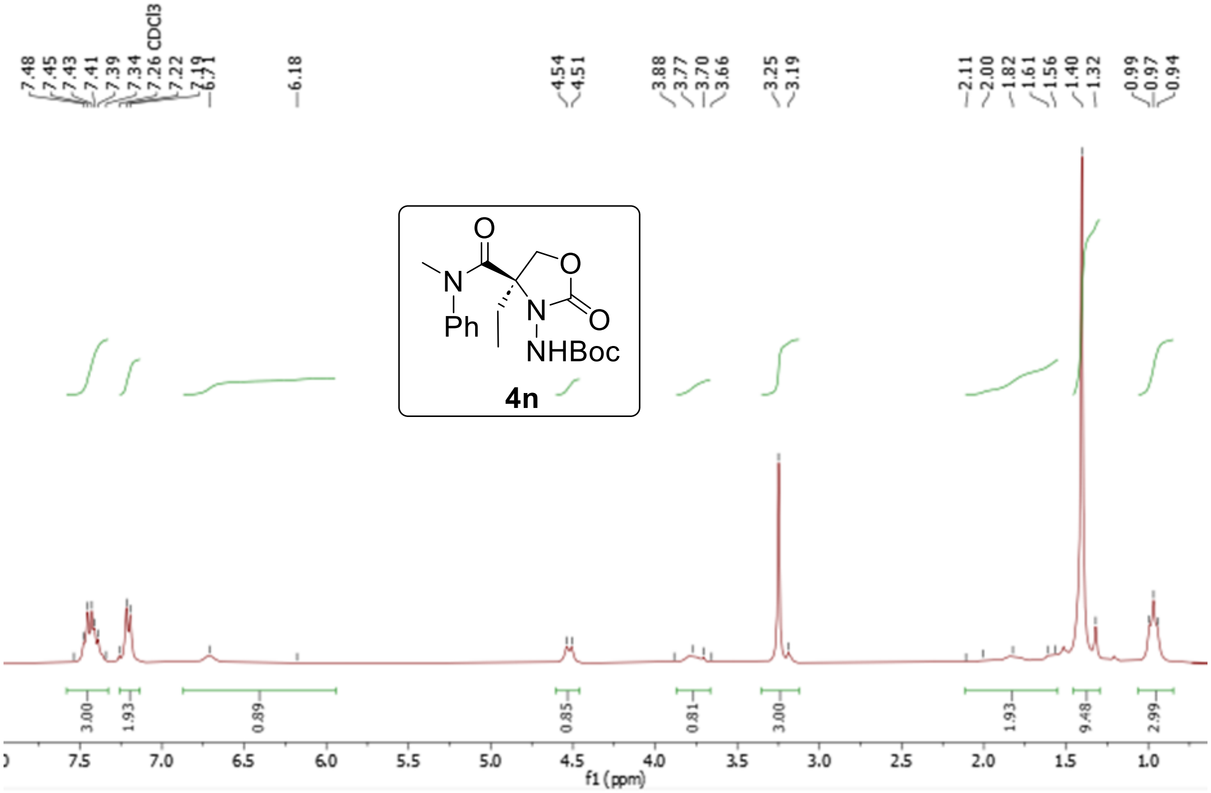
7.48
7.45
7.43
7.41
7.39
7.34
7.26 CDCl3
7.22
7.19
6.71
6.18
4.54
4.51
3.88
3.77
3.70
3.66
3.25
3.19
2.11
2.00
1.82
1.61
1.56
1.40
1.32
0.99
0.97
0.94
4n
3.00
1.93
0.89
0.85
0.81
3.00
1.93
9.48
2.99
f1 (ppm)

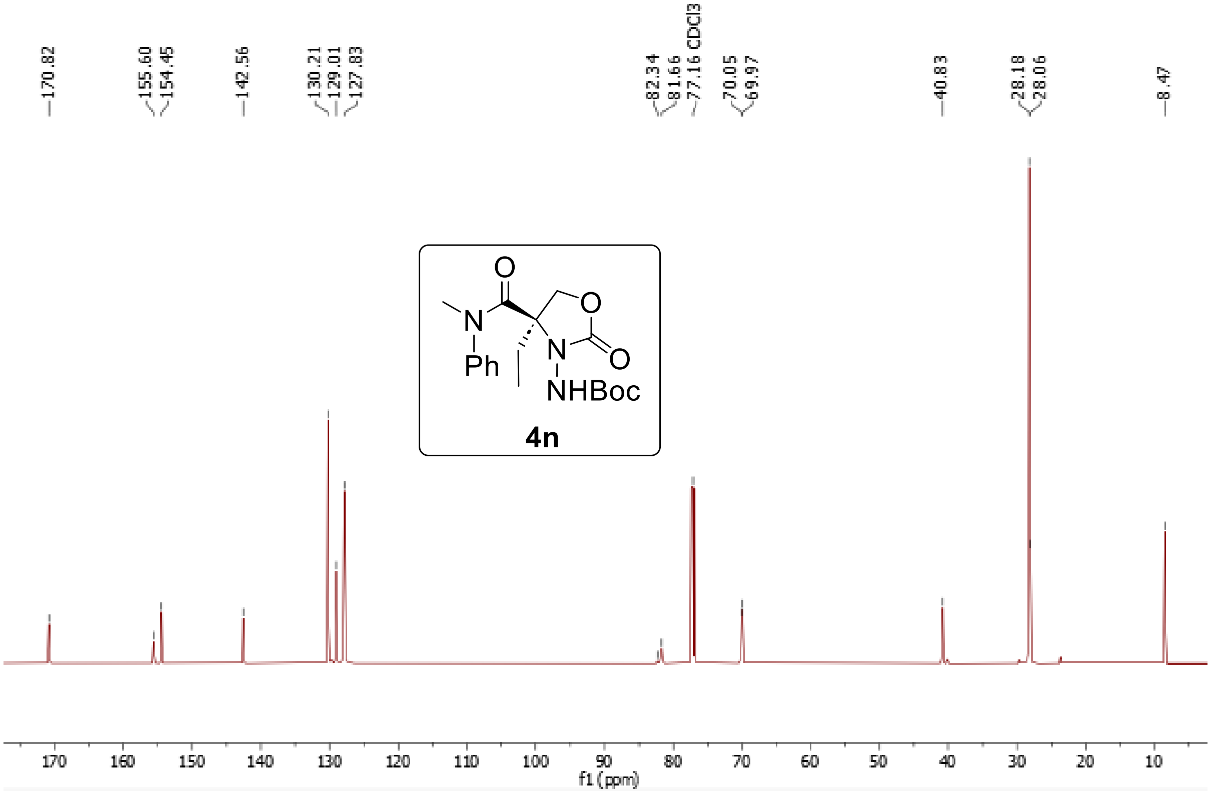
170.82
155.60
154.45
142.56
130.21
129.01
127.83
82.34
81.66
77.16 CDCl3
70.05
69.97
40.83
28.18
28.06
8.47
4n
f1 (ppm)

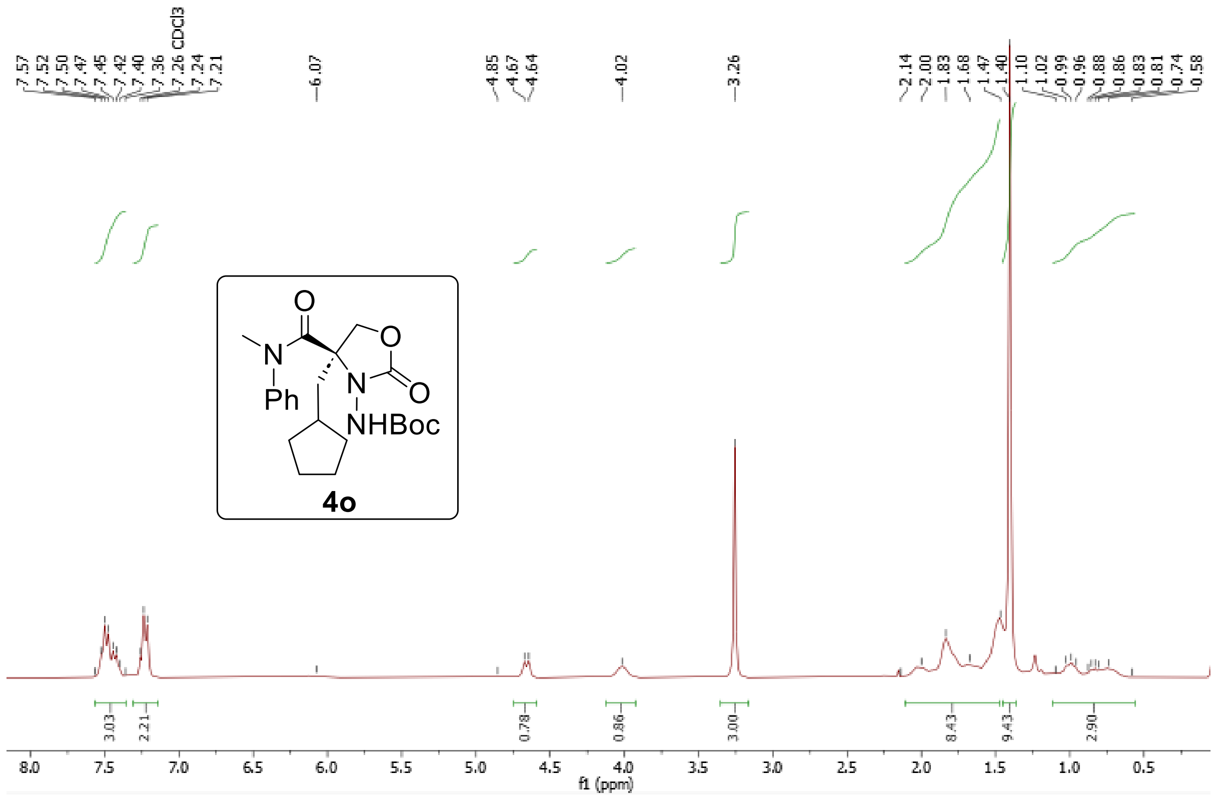

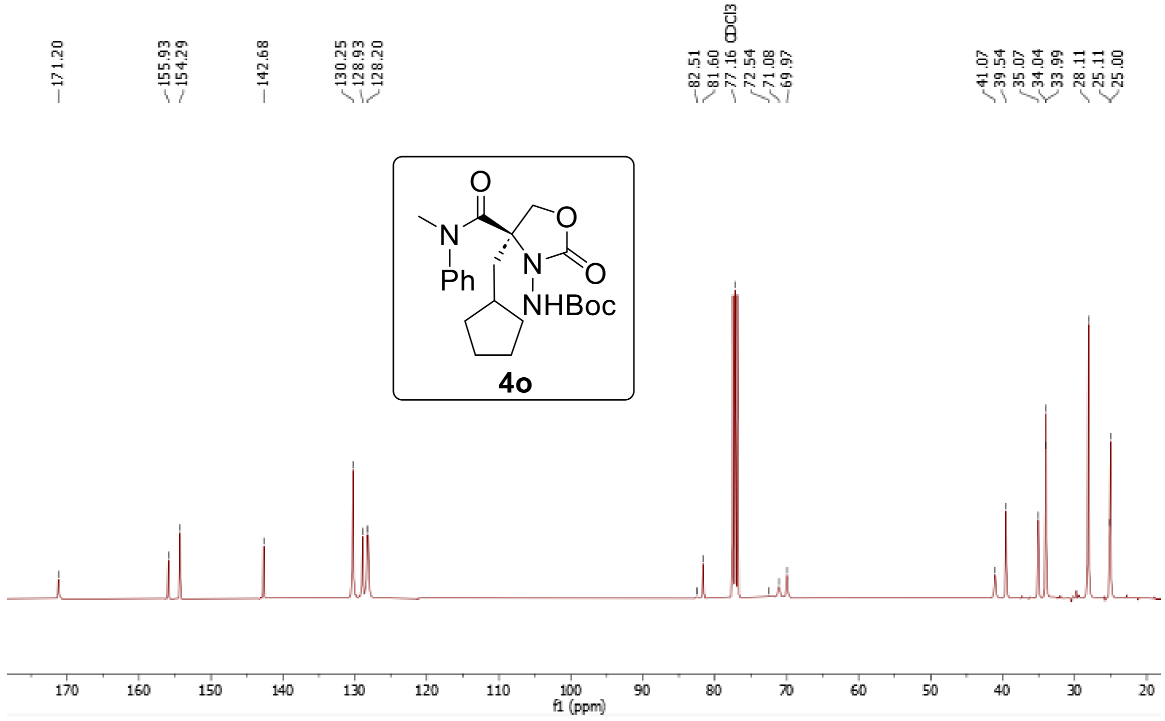

(v) Oxazolidinones 5a and 5j

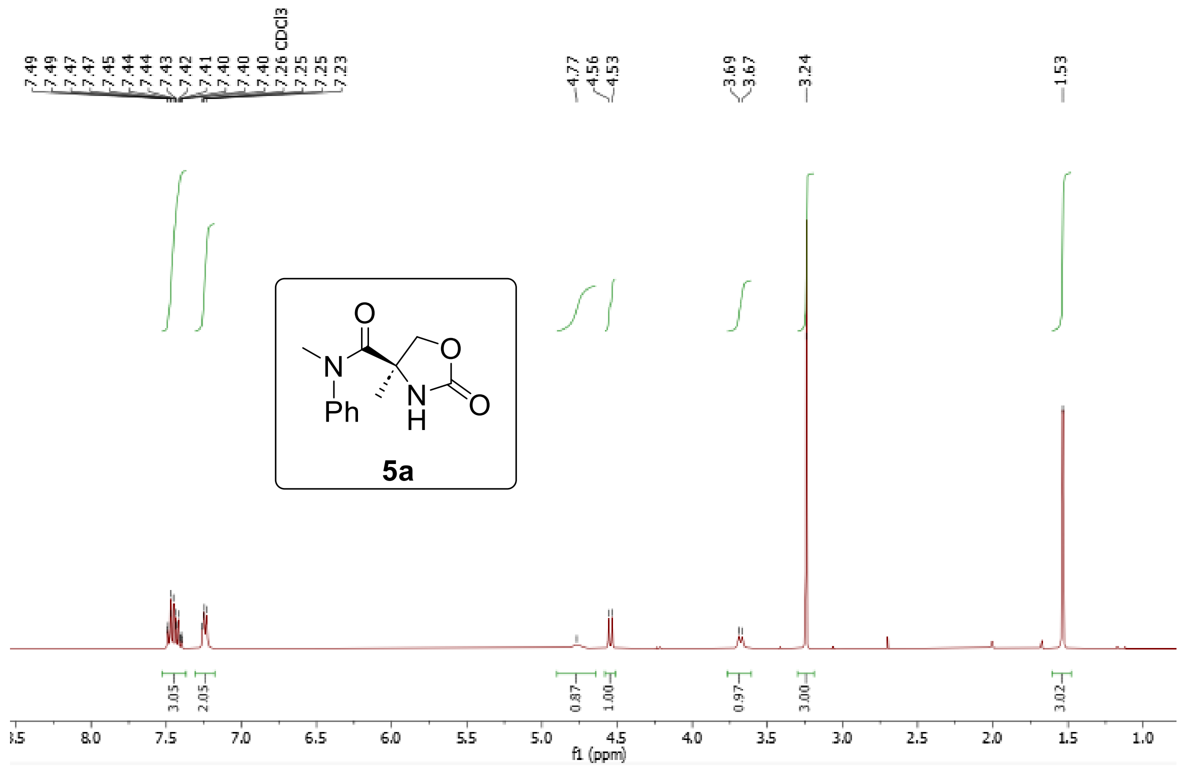

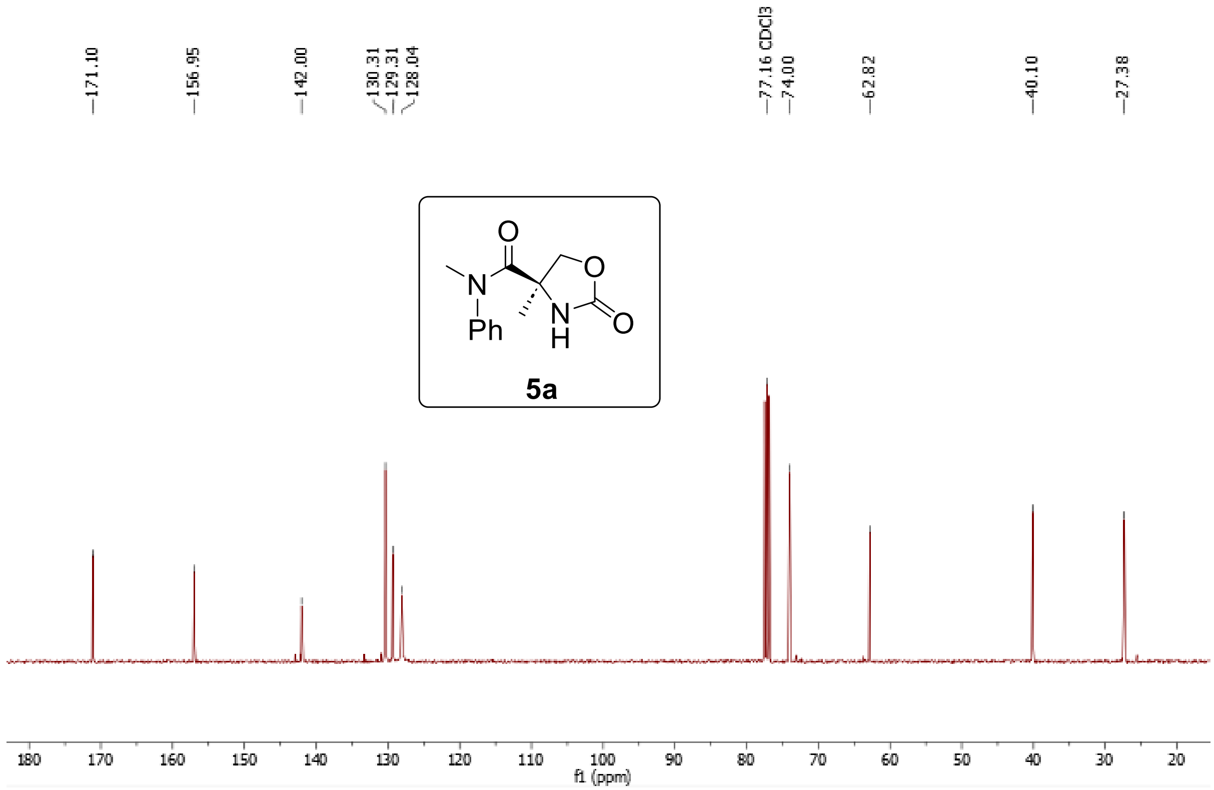

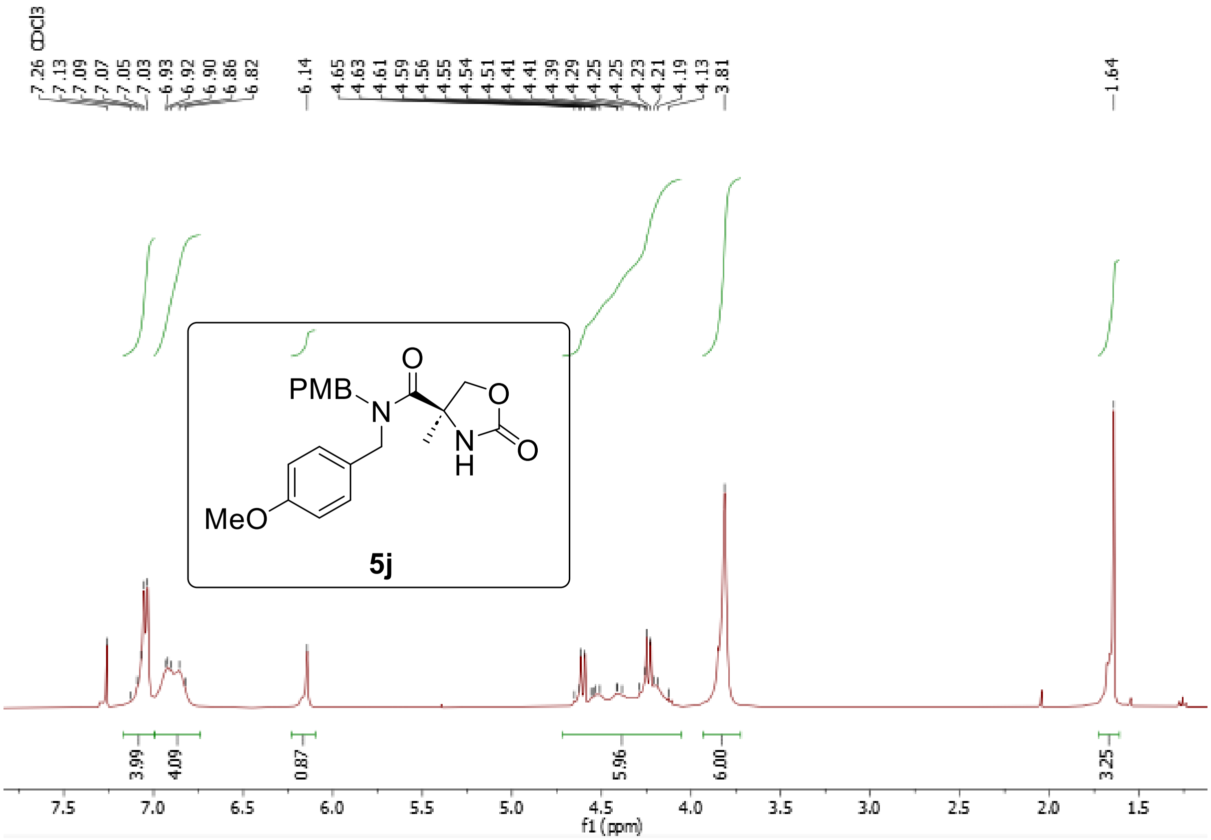
7.26 CDCl3
7.13
7.09
7.07
7.05
7.03
6.93
6.92
6.90
6.86
6.82
6.14
4.65
4.63
4.61
4.59
4.56
4.55
4.54
4.51
4.41
4.41
4.39
4.29
4.25
4.25
4.23
4.21
4.19
4.13
3.81
1.64
PMB
N
O
O
N
H
O
MeO
5j
3.99
4.09
0.87
5.96
6.00
3.25
f1 (ppm)

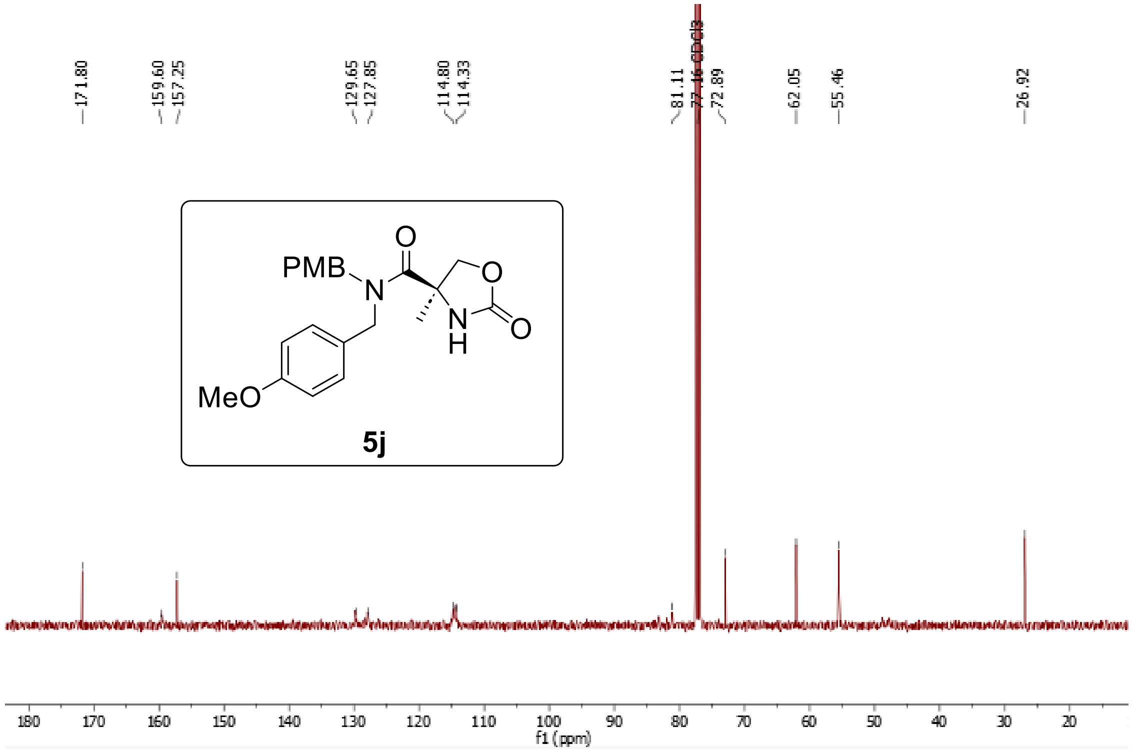
171.80
159.60
157.25
129.65
127.85
114.80
114.33
81.11
77.16 CDCl3
72.89
62.05
55.46
26.92
PMB
N
O
O
N
H
O
MeO
5j
f1 (ppm)

(v) PMB-Deprotected Oxazolidinone 6j

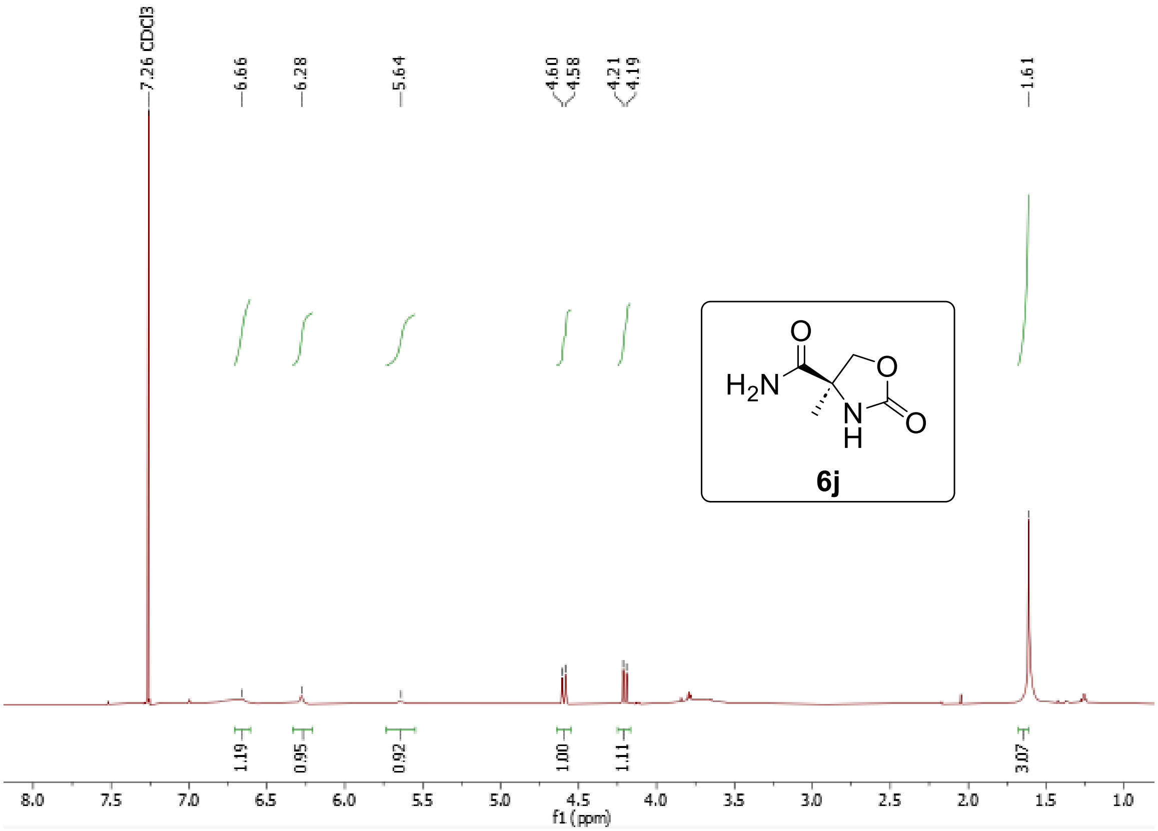

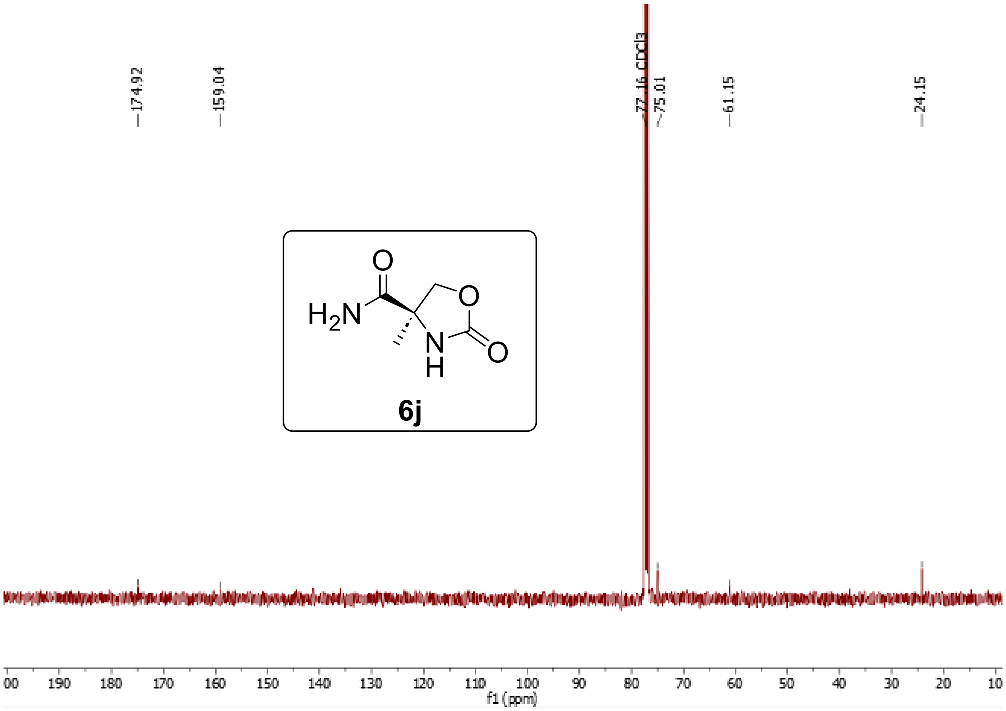

11. HPLC Traces of hydrazides 3a-u, 4a, 4c, 4j, 4n, 4o, 5a, and 5j

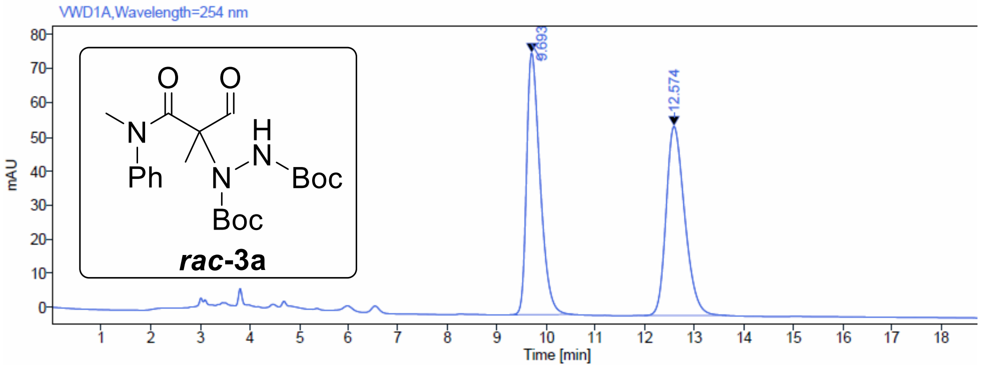

Signal: wavelength = 254 nm

No.	Ret. Time	Area%
1	9.693	50.01
2	12.574	49.99
Total		100.00

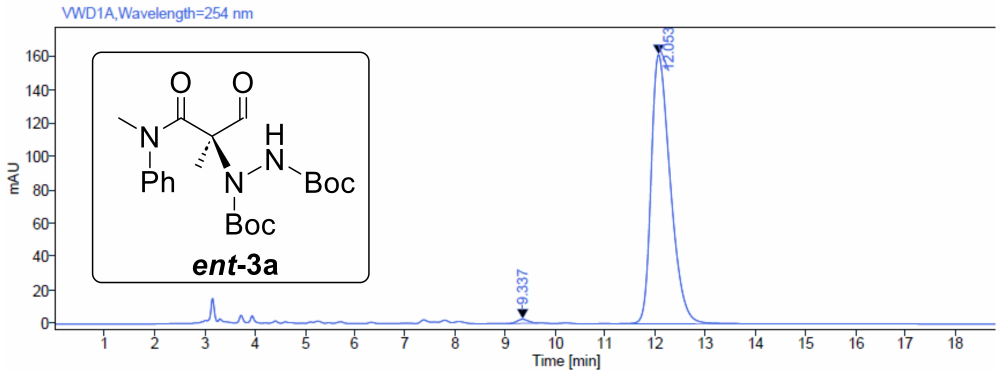

Signal: wavelength = 254 nm

No.	Ret. Time	Area%
1	9.337	0.89
2	12.053	99.11
	Total	100.00

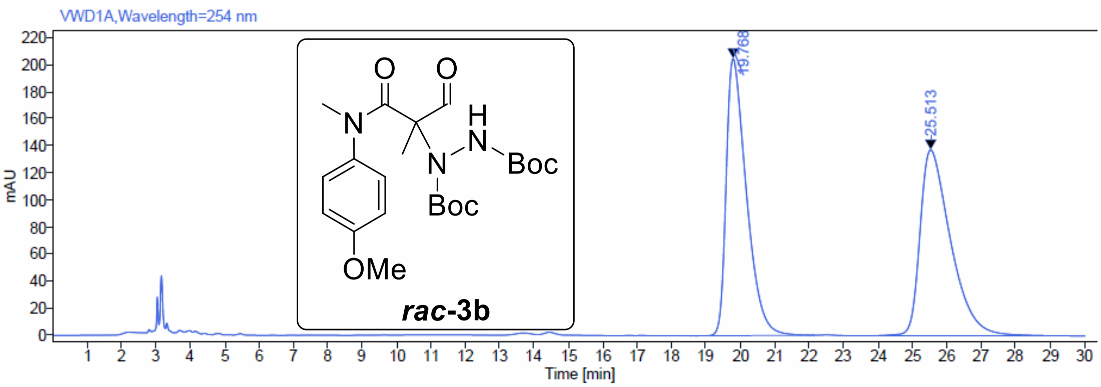

Signal: wavelength = 254 nm

No.	Ret. Time	Area%
1	19.768	49.62
2	25.513	50.38
Total		100.00

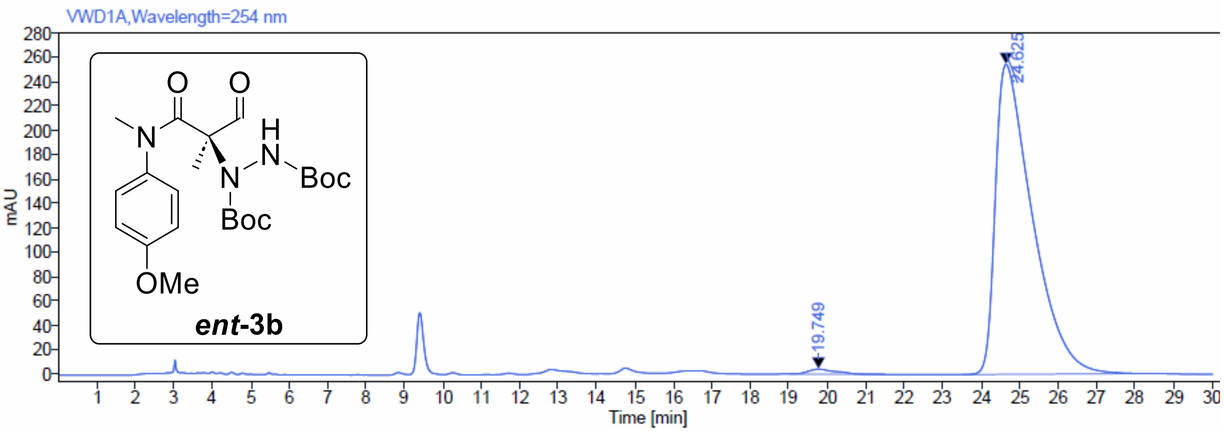

Signal: wavelength = 254 nm

No.	Ret. Time	Area%
1	19.749	1.18
2	24.629	98.82
Total		100.00

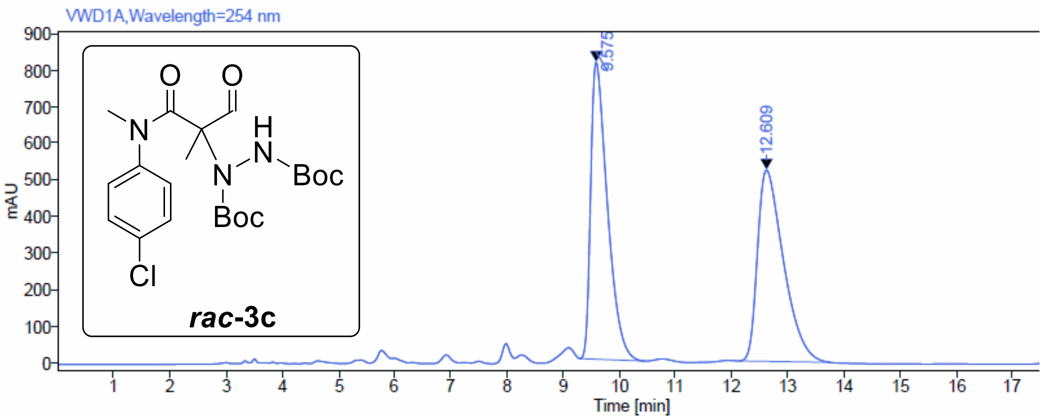

Signal: wavelength = 254 nm

No.	Ret. Time	Area%
1	9.575	49.95
2	12.609	50.05
Total		100.00

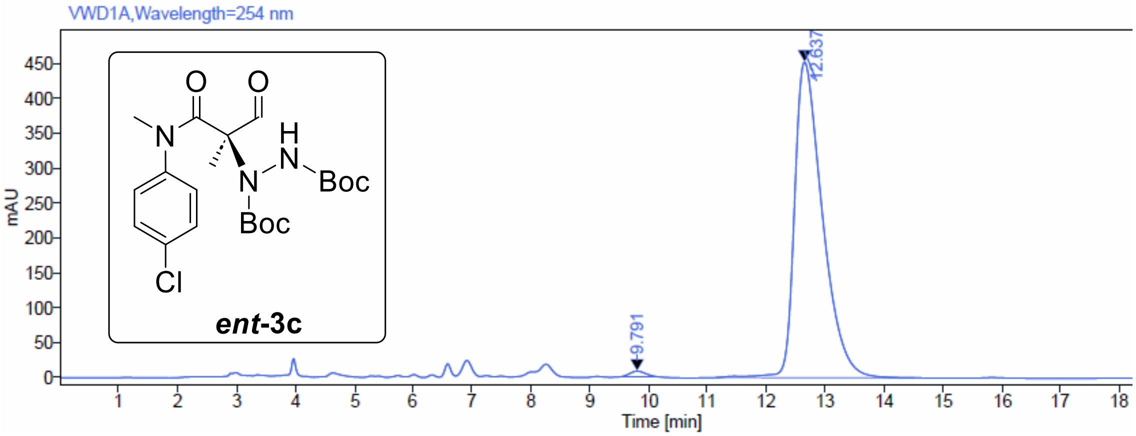

Signal: wavelength = 254 nm

No.	Ret. Time	Area%
1	9.791	1.03
2	12.637	98.97
Total		100.00

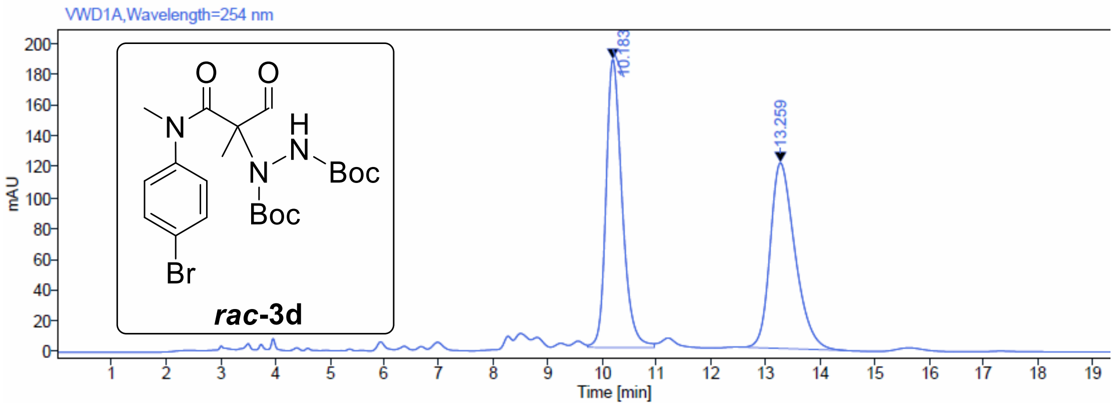

Signal: wavelength = 254 nm

No.	Ret. Time	Area%
1	10.183	50.13
2	13.259	49.87
Total		100.00

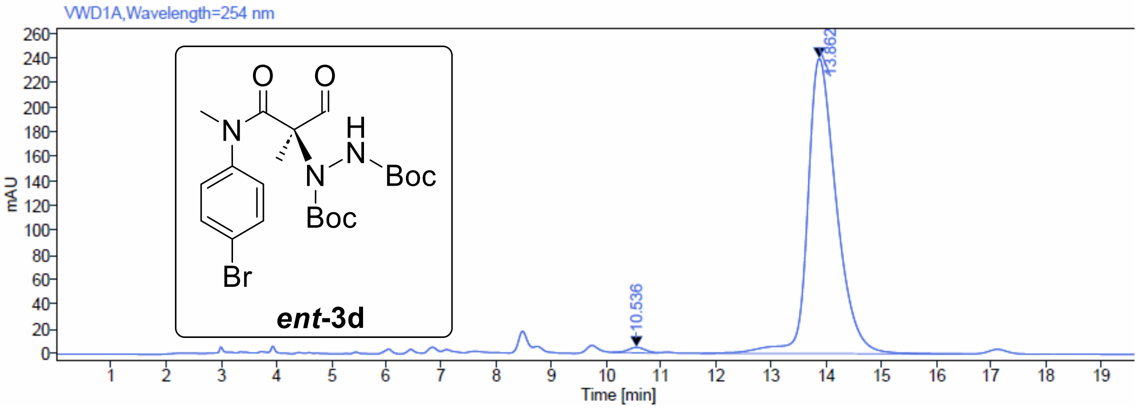

Signal: wavelength = 254 nm

No.	Ret. Time	Area%
1	10.536	1.12
2	13.862	98.88
Total		100.00

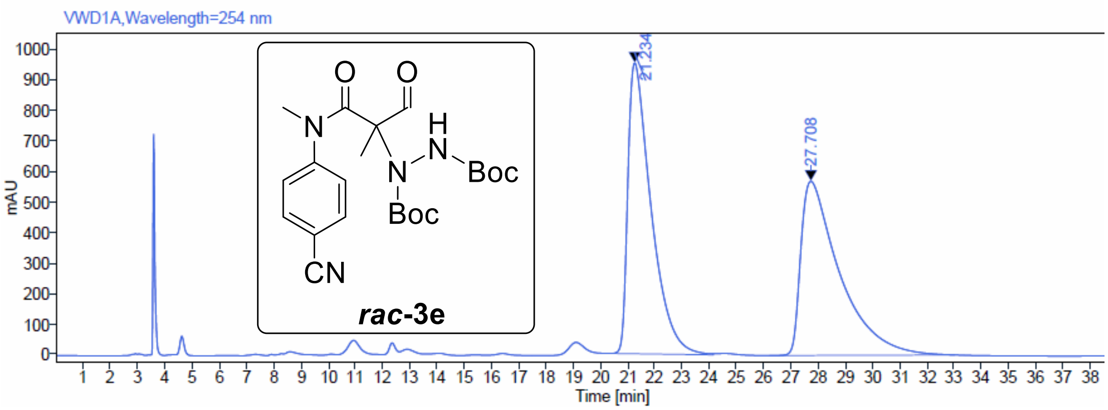

Signal: wavelength = 254 nm

No.	Ret. Time	Area%
1	21.234	50.03
2	27.708	49.97
Total		100.00

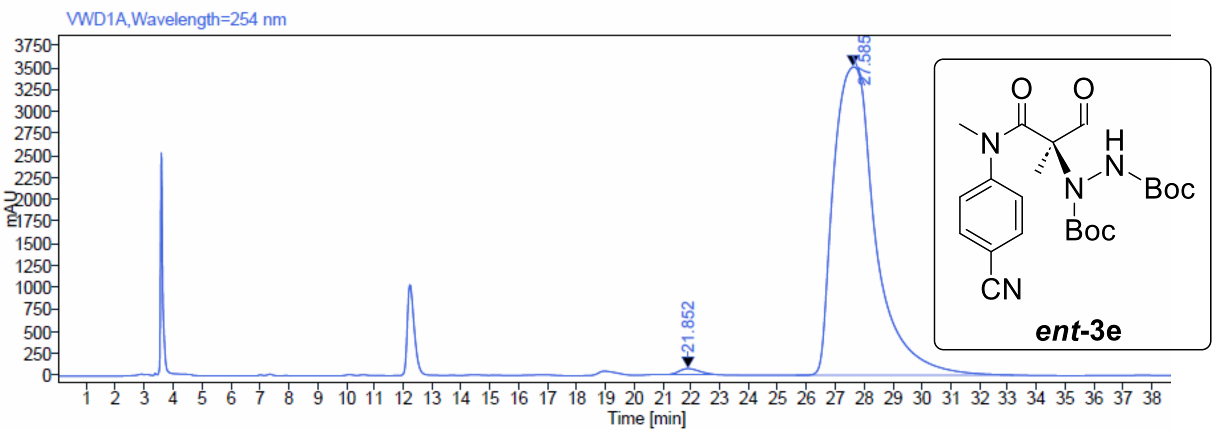

Signal: wavelength = 254 nm

No.	Ret. Time	Area%
1	21.852	0.87
2	27.585	99.13
Total		100.00

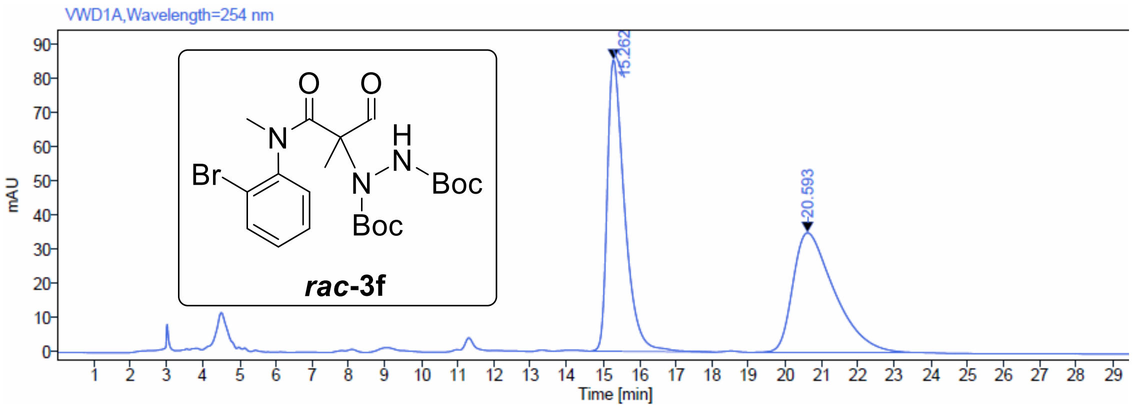

Signal: wavelength = 254 nm

No.	Ret. Time	Area%
1	15.262	50.34
2	20.593	49.66
Total		100.00

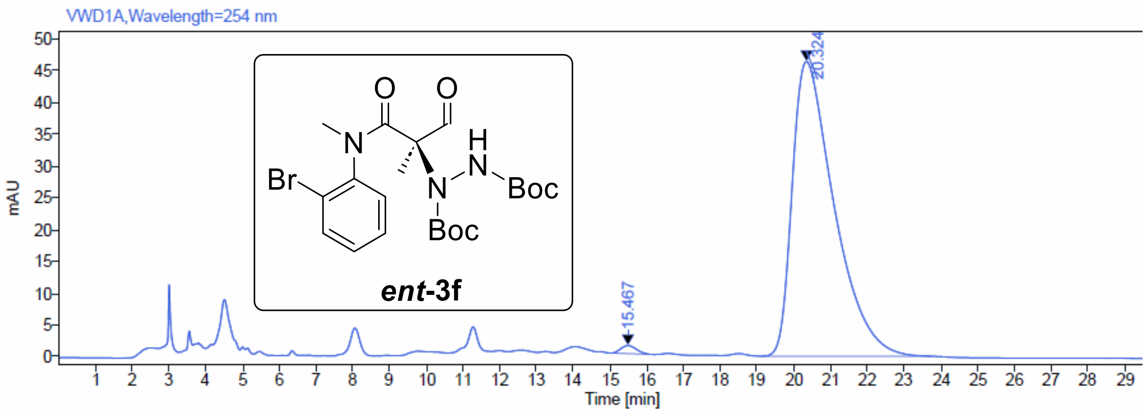

Signal: wavelength = 254 nm

No.	Ret. Time	Area%
1	15.467	1.01
2	20.324	98.99
Total		100.00

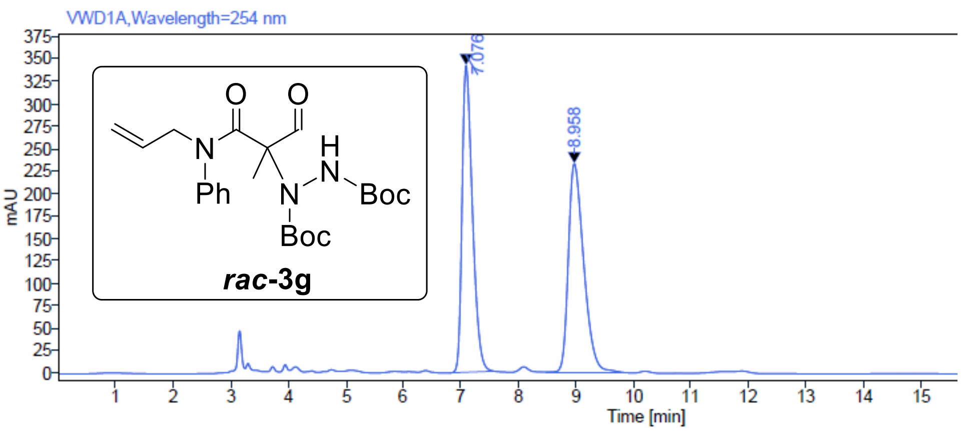

Signal: wavelength = 254 nm

No.	Ret. Time	Area%
1	7.076	49.97
2	8.958	50.03
Total		100.00

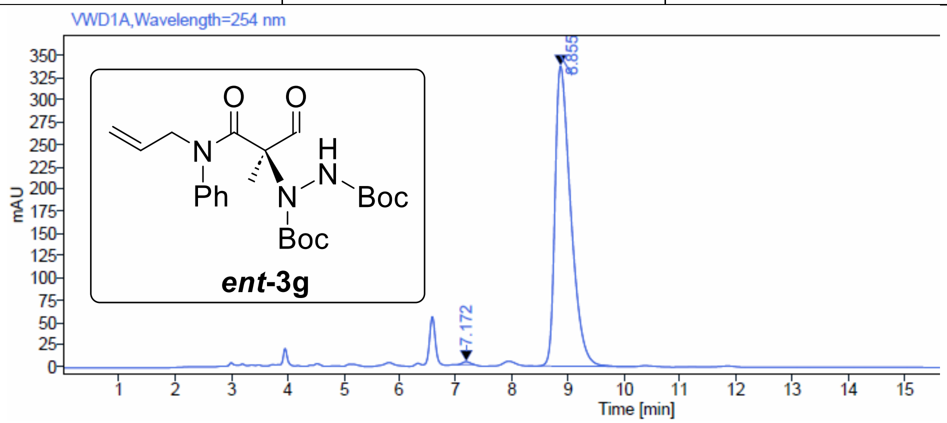

Signal: wavelength = 254 nm

No.	Ret. Time	Area%
1	7.172	0.48
2	8.855	99.52
Total		100.00

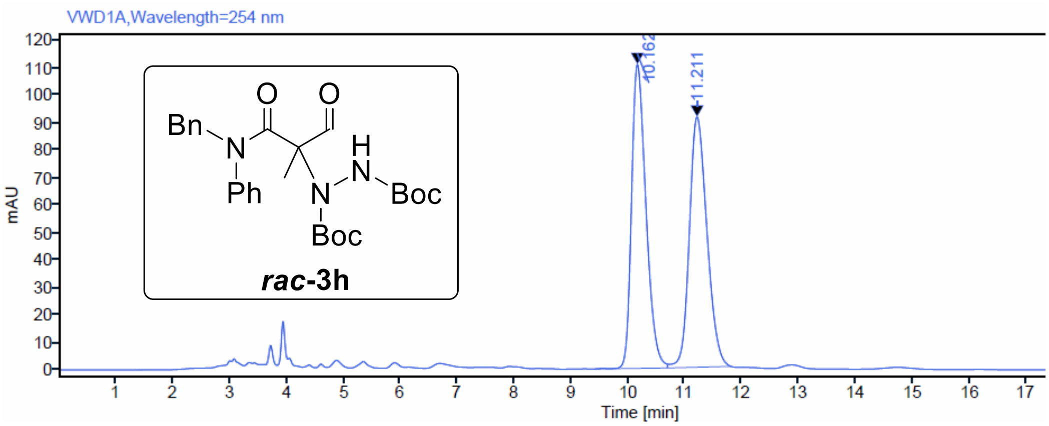

Signal: wavelength = 254 nm

No.	Ret. Time	Area%
1	10.162	49.83
2	11.211	50.17
Total		100.00

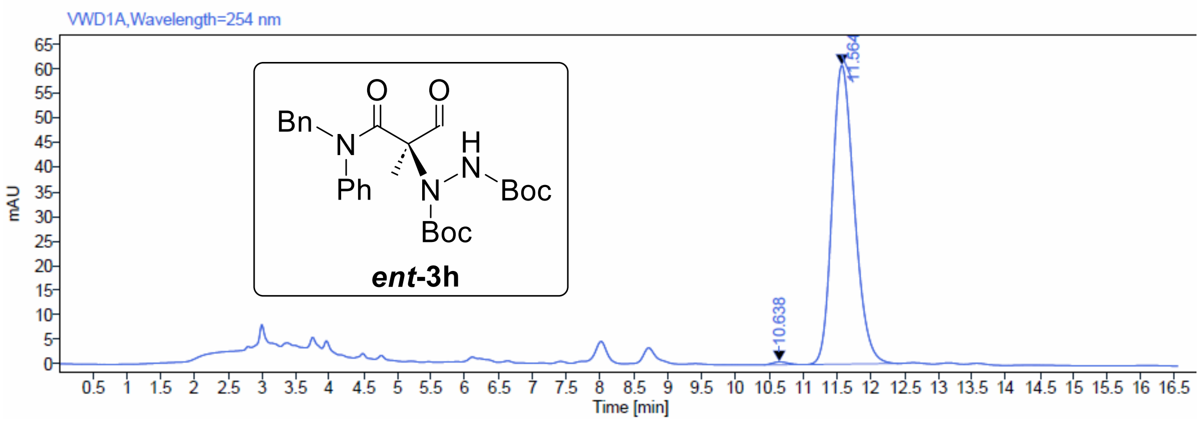

Signal: wavelength = 254 nm

No.	Ret. Time	Area%
1	10.638	0.65
2	11.564	99.35
Total		100.00

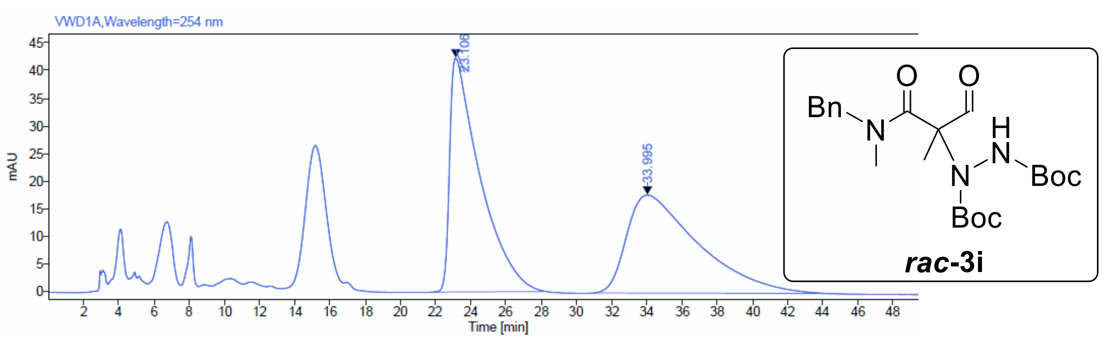

Signal: wavelength = 254 nm

No.	Ret. Time	Area %
1	23.106	50.29
2	33.995	49.71
Total		100.00

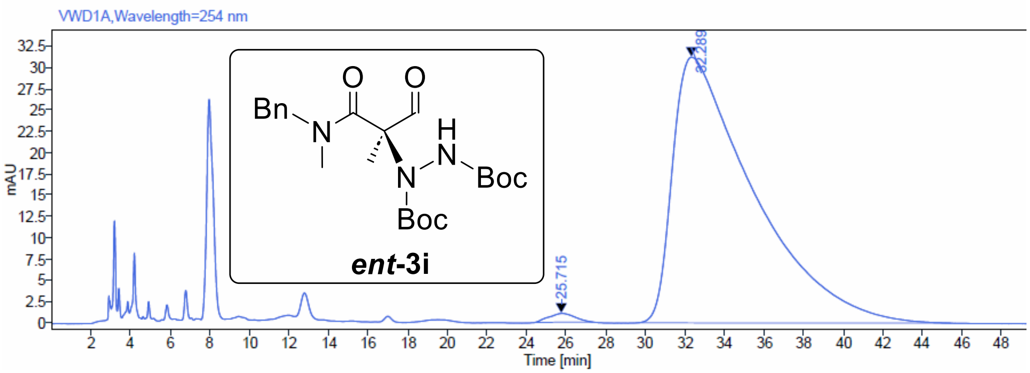

Signal: wavelength = 254 nm

No.	Ret. Time	Area%
1	25.715	1.06
2	32.289	98.94
Total		100.00

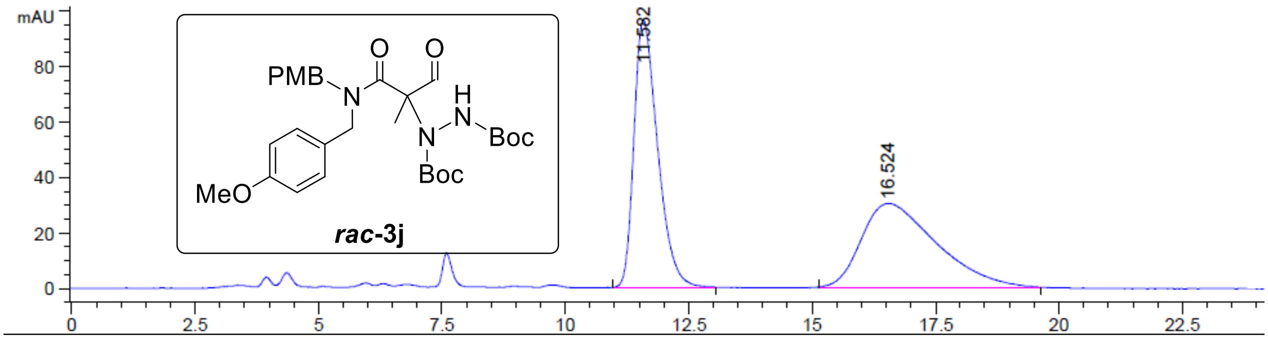

Signal: wavelength = 254 nm

No.	Ret. Time	Area%
1	11.582	50.39
2	16.524	49.61
Total		100.00

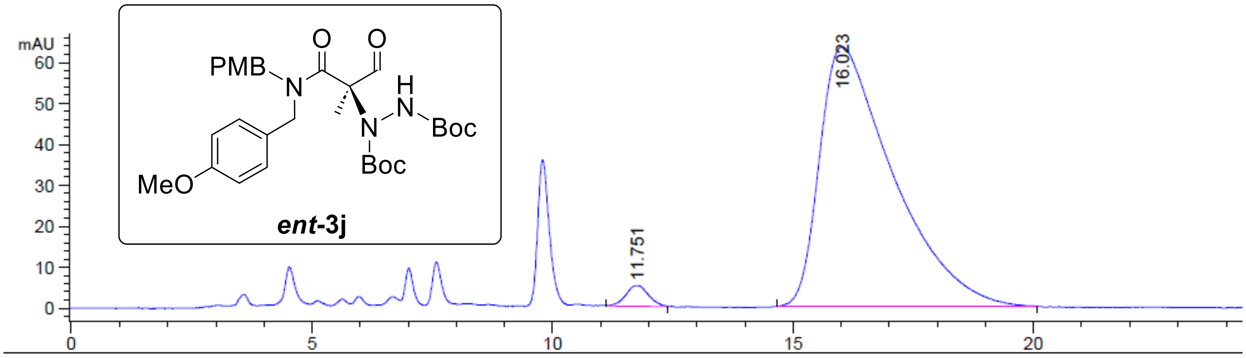

Signal: wavelength = 254 nm

No.	Ret. Time	Area%
1	11.751	2.36
2	16.023	97.64
Total		100.00

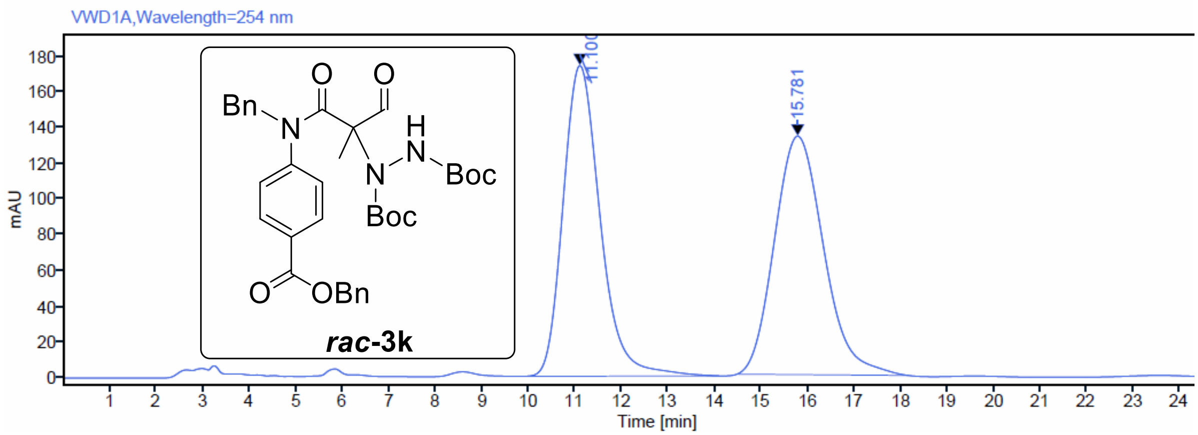

Signal: wavelength = 254 nm

No.	Ret. Time	Area%
1	11.100	49.55
2	15.781	50.45
Total		100.00

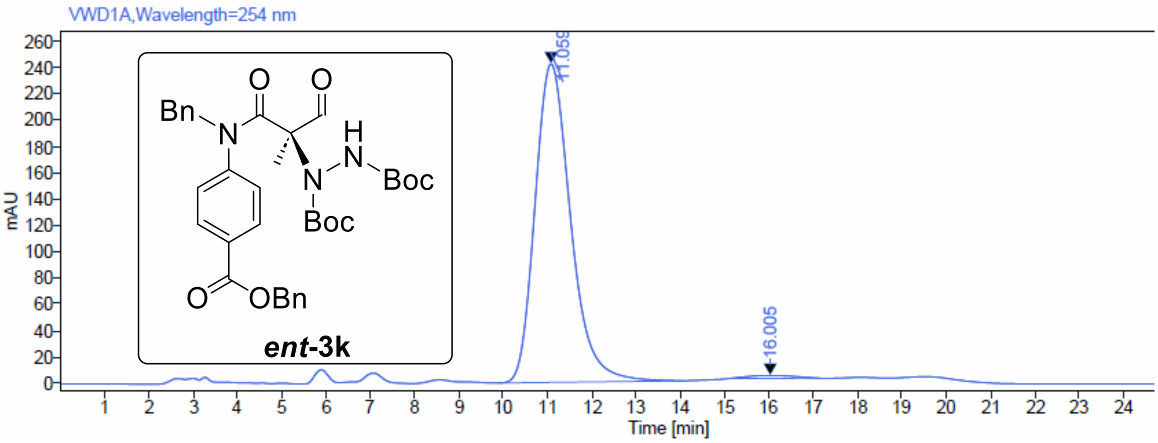

Signal: wavelength = 254 nm

No.	Ret. Time	Area%
1	11.059	98.82
2	16.005	1.18
Total		100.00

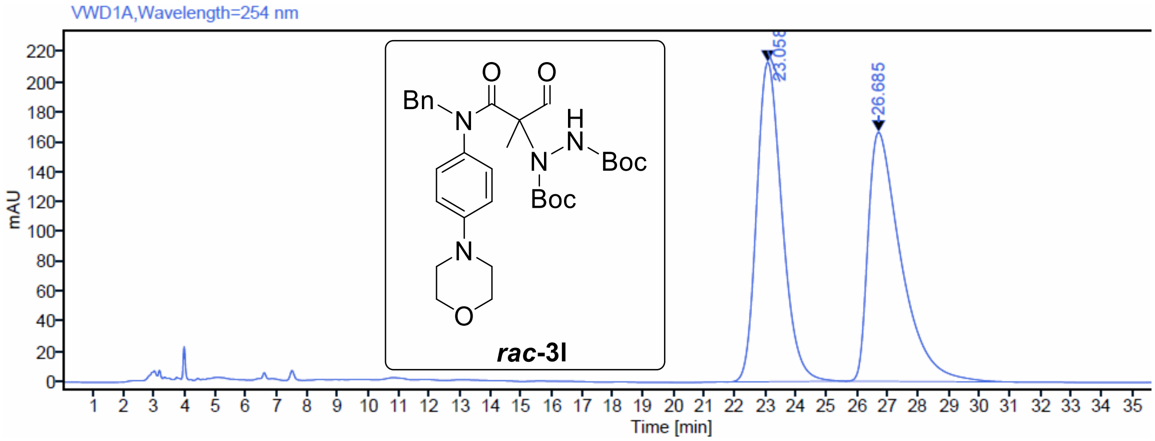

Signal: wavelength = 254 nm

No.	Ret. Time	Area%
1	23.058	49.77
2	26.685	50.23
Total		100.00

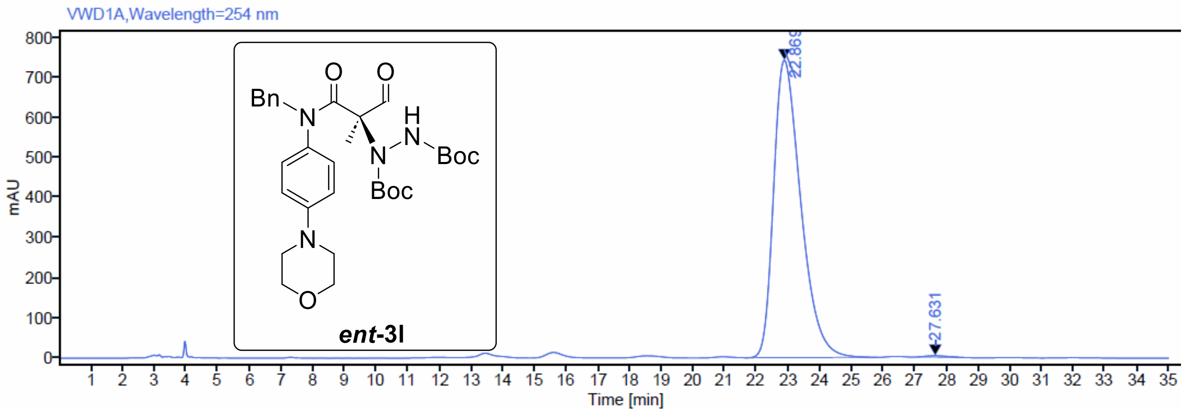

Signal: wavelength = 254 nm

No.	Ret. Time	Area%
1	22.869	99.59
2	27.631	0.41
Total		100.00

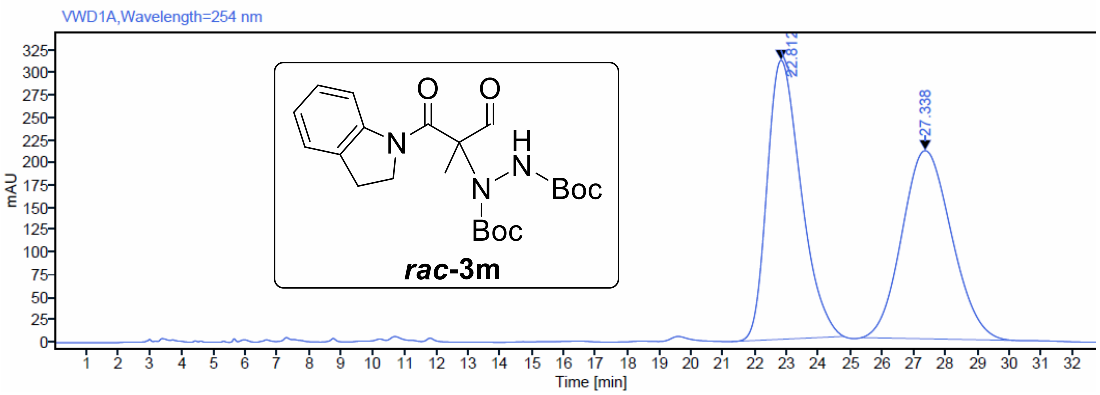

Signal: wavelength = 254 nm

No.	Ret. Time	Area%
1	22.812	50.36
2	27.338	49.64
Total		100.00

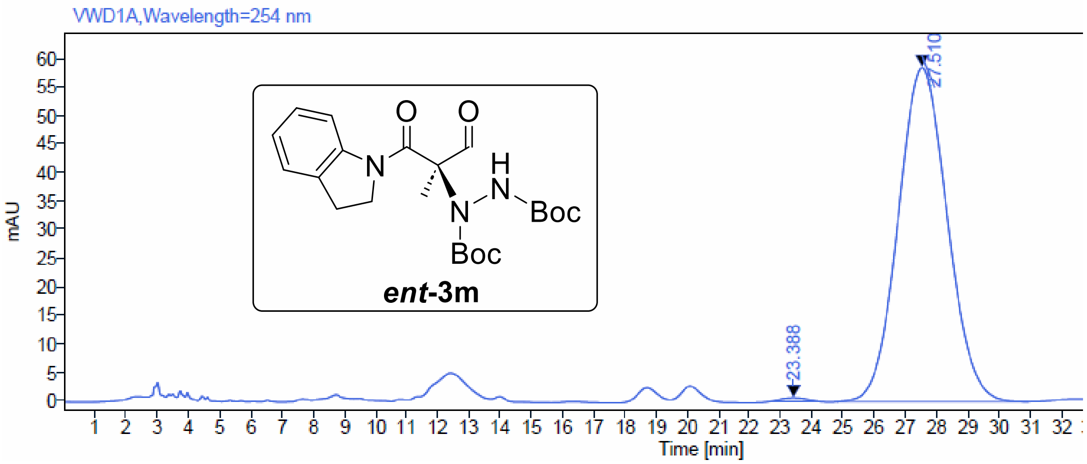

Signal: wavelength = 254 nm

No.	Ret. Time	Area%
1	23.388	0.50
2	27.510	99.50
Total		100.00

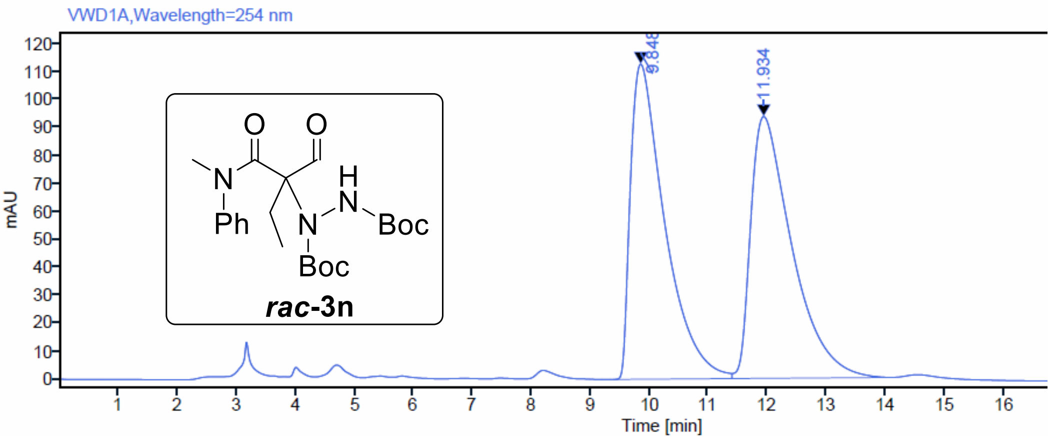

Signal: wavelength = 254 nm

No.	Ret. Time	Area%
1	9.848	50.08
2	11.934	49.92
Total		100.00

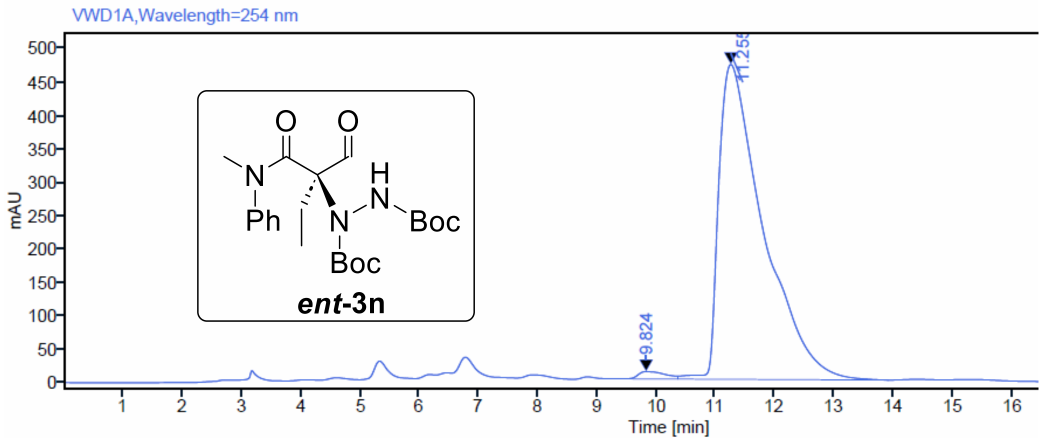

Signal: wavelength = 254 nm

No.	Ret. Time	Area%
1	9.824	1.40
2	11.255	98.60
Total		100.00

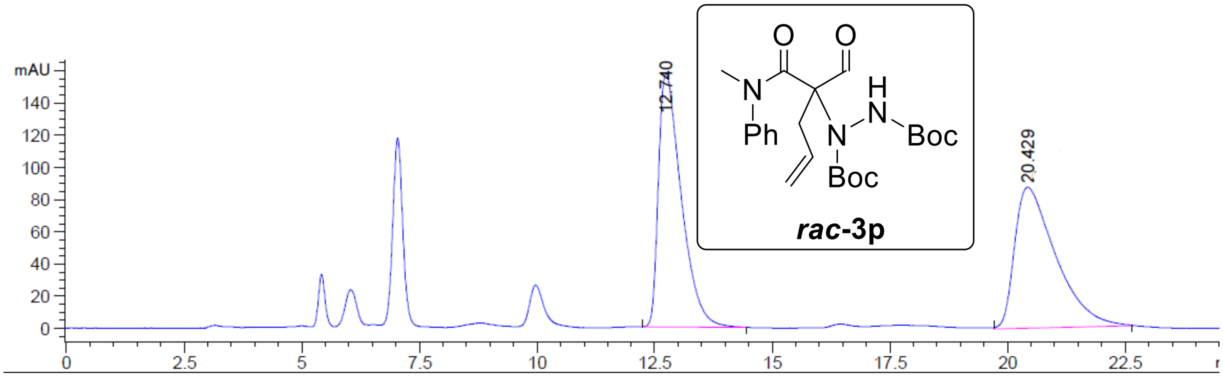

Signal: wavelength = 254 nm

No.	Ret. Time	Area%
1	12.740	50.98
2	20.429	49.12
Total		100.00

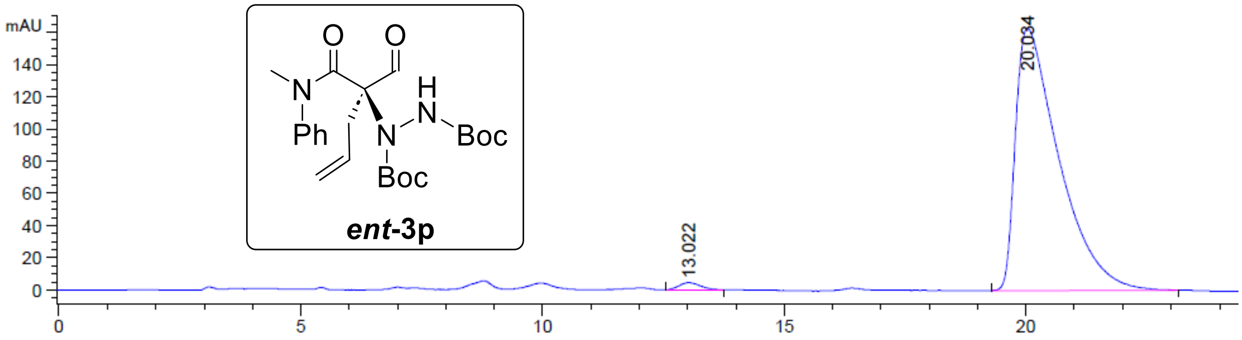

Signal: wavelength = 254 nm

No.	Ret. Time	Area%
1	13.022	1.25
2	20.034	98.75
Total		100.00

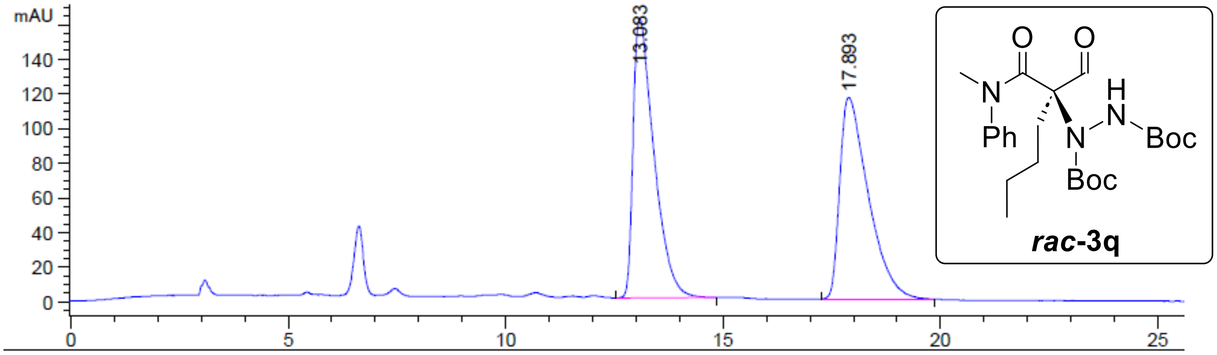

Signal: wavelength = 254 nm

No.	Ret. Time	Area%
1	13.083	50.31
2	17.893	49.69
Total		100.00

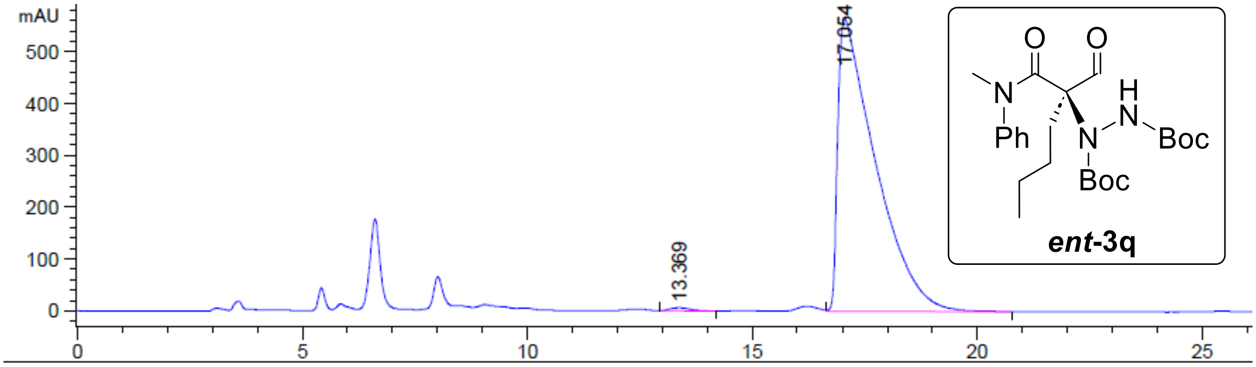

Signal: wavelength = 254 nm

No.	Ret. Time	Area%
1	13.369	0.55
2	17.054	99.45
Total		100.00

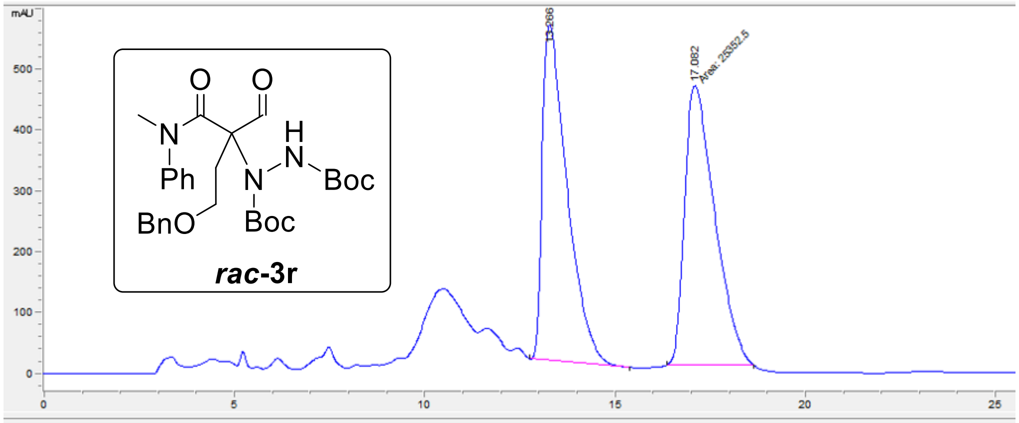

Signal: wavelength = 254 nm

No.	Ret. Time	Area%
1	13.266	49.68
2	17.082	50.32
Total		100.00

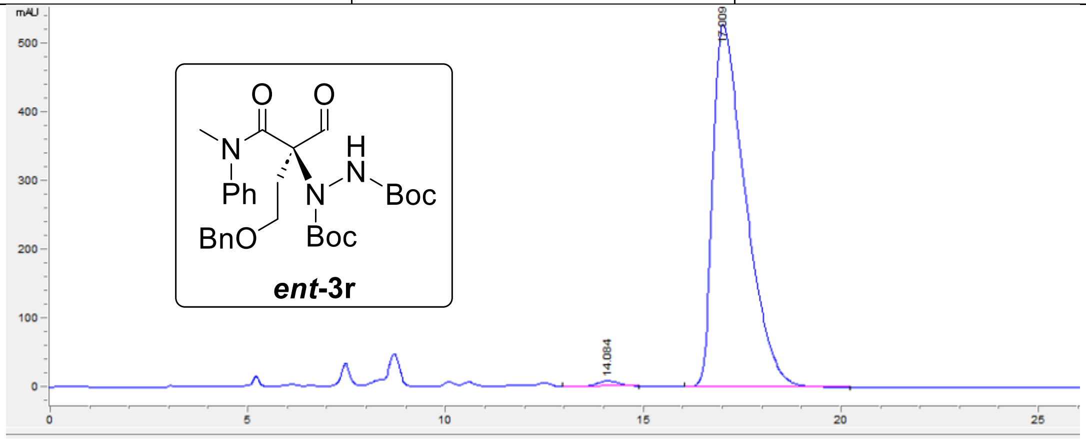

Signal: wavelength = 254 nm

No.	Ret. Time	Area%
1	14.084	0.97
2	17.009	99.03
Total		100.00

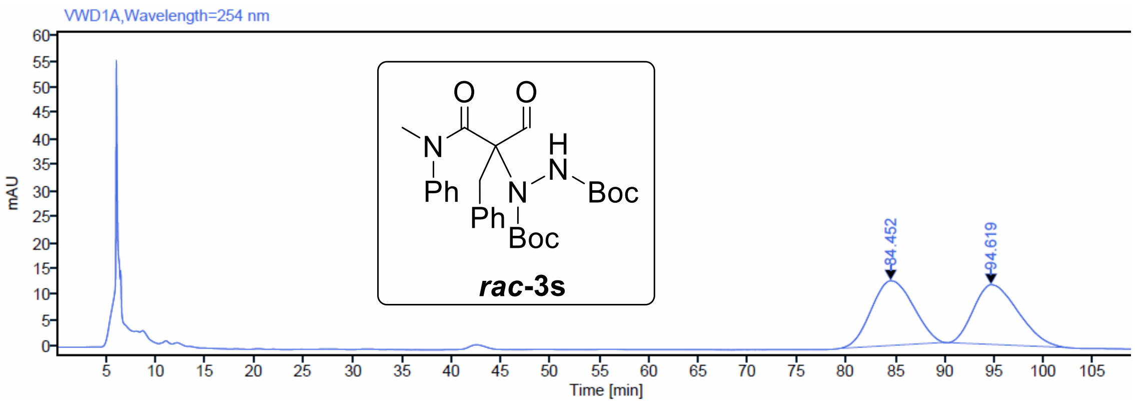

Signal: wavelength = 254 nm

No.	Ret. Time	Area%
1	84.452	50.48
2	94.619	49.52
Total		100.00

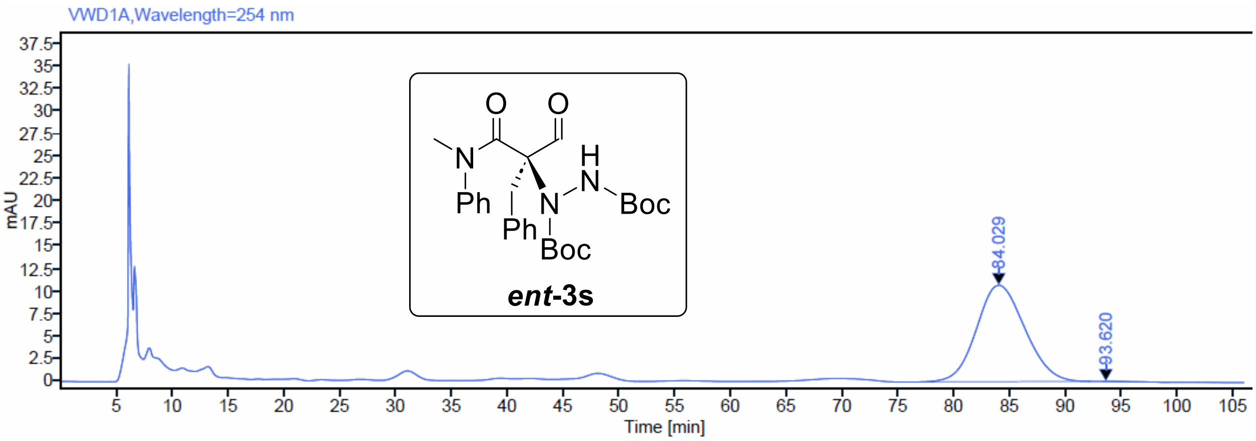

Signal: wavelength = 254 nm

No.	Ret. Time	Area%
1	84.029	99.88
2	93.620	0.12
Total		100.00

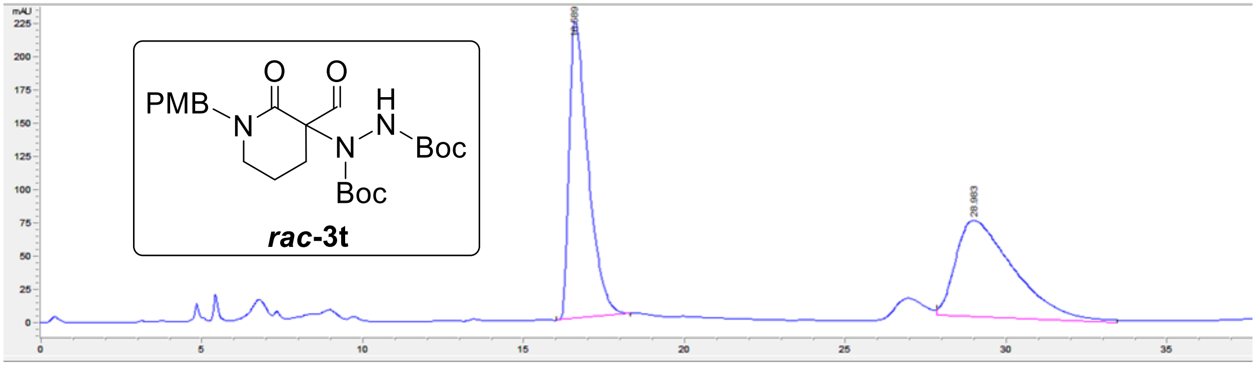

Signal: wavelength = 254 nm

No.	Ret. Time	Area%
1	16.589	49.89
2	28.983	50.11
Total		100.00

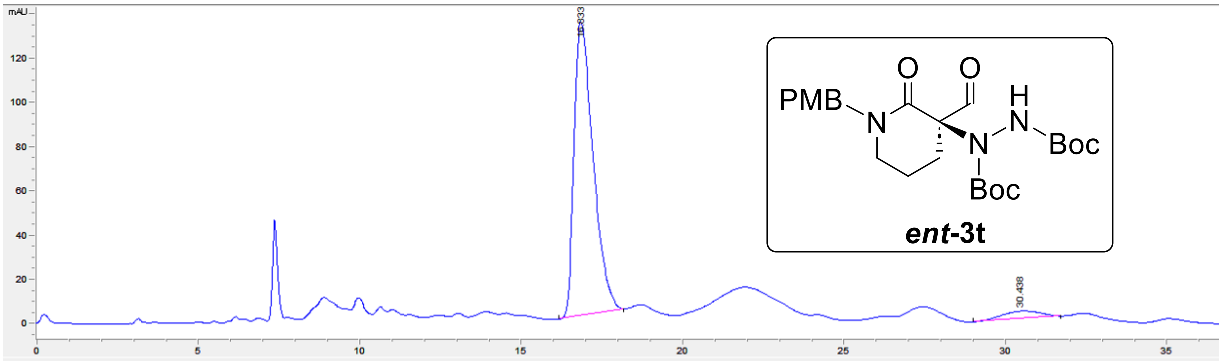

Signal: wavelength = 254 nm

No.	Ret. Time	Area%
1	16.833	94.64
2	30.438	5.39
Total		100.00

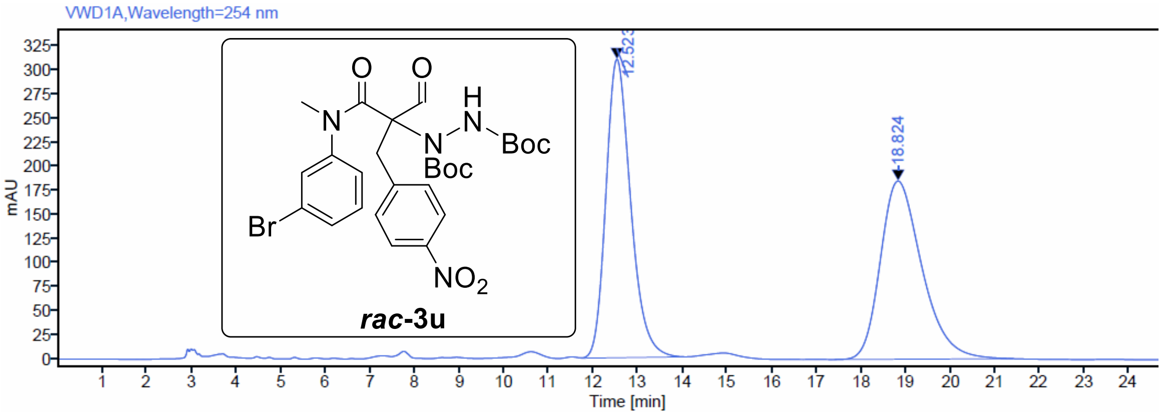

Signal: wavelength = 254 nm

No.	Ret. Time	Area%
1	12.523	49.98
2	18.824	50.02
Total		100.00

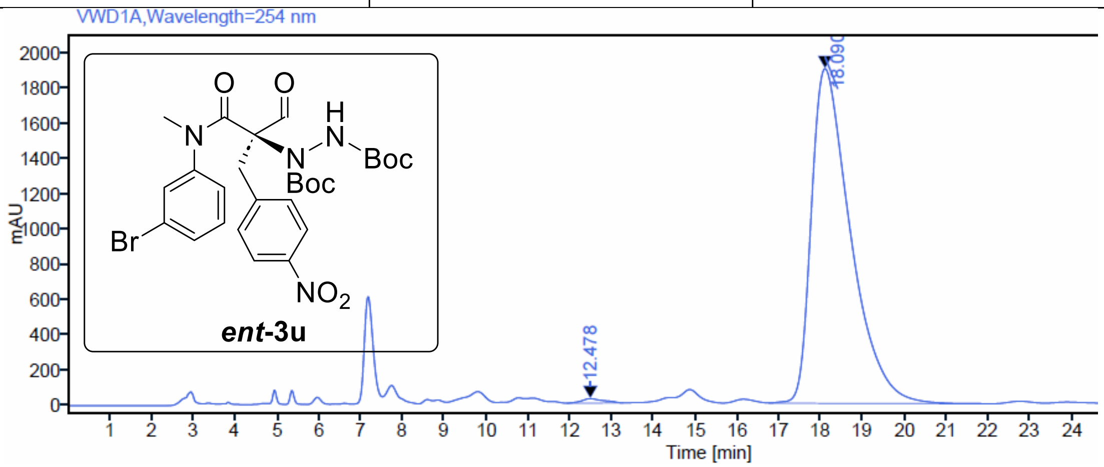

Signal: wavelength = 254 nm

No.	Ret. Time	Area%
1	12.478	0.74
2	18.090	99.26
Total		100.00

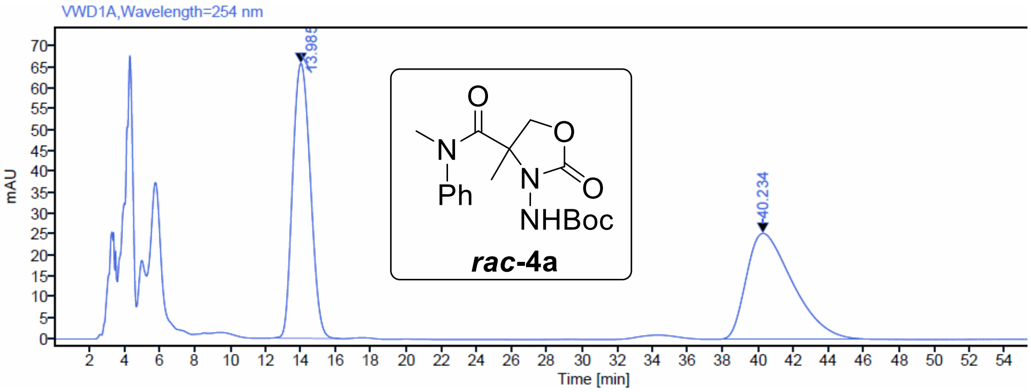

Signal: wavelength = 254 nm

No.	Ret. Time	Area%
1	13.985	49.97
2	40.234	50.03
Total		100.00

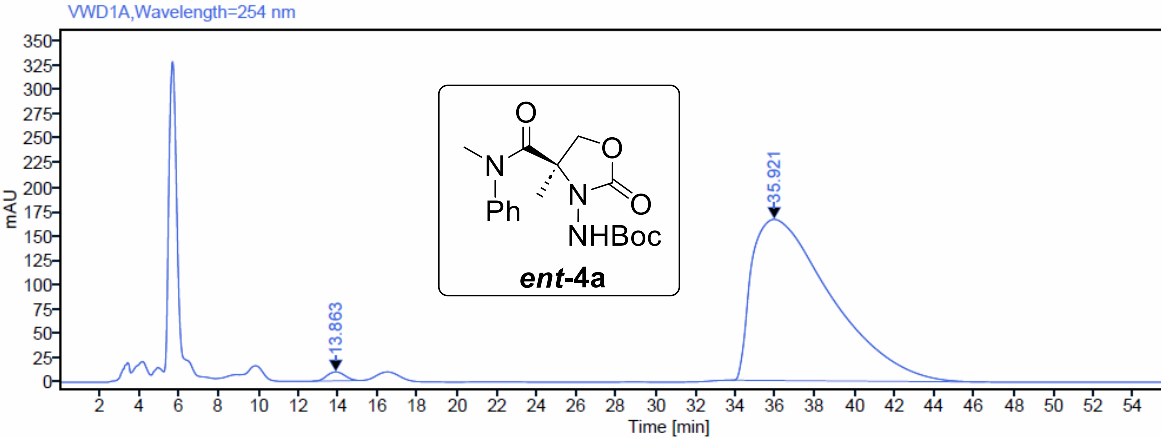

Signal: wavelength = 254 nm

No.	Ret. Time	Area%
1	13.863	1.22
2	35.921	98.78
Total		100.00

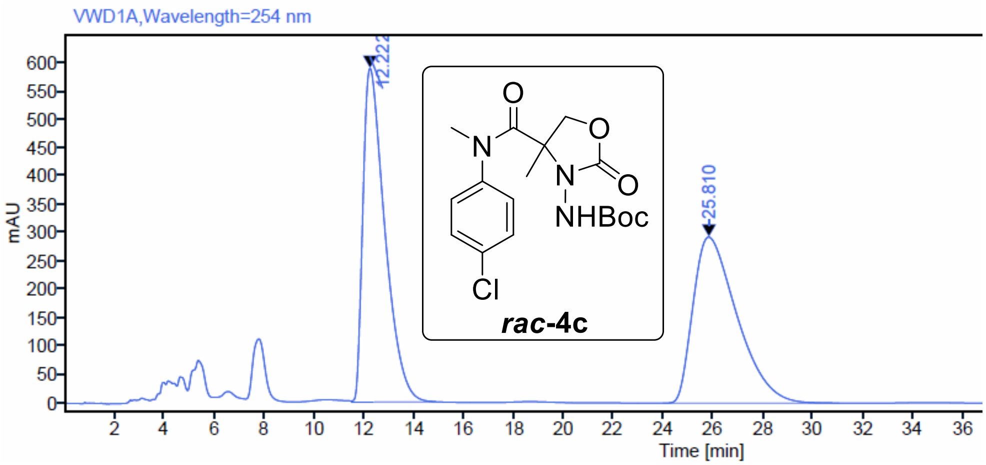

Signal: wavelength = 254 nm

No.	Ret. Time	Area%
1	12.222	50.04
2	25.810	49.96
Total		100.00

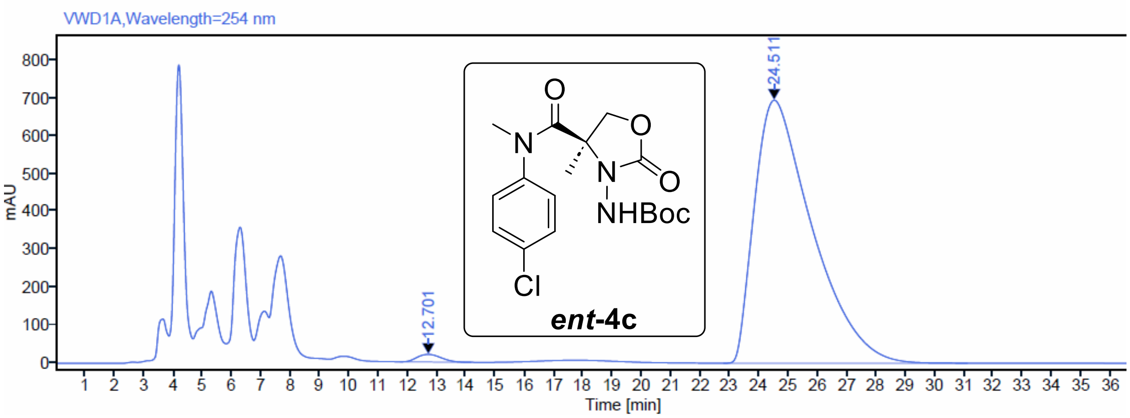

Signal: wavelength = 254 nm

No.	Ret. Time	Area%
1	12.701	1.19
2	24.511	98.81
Total		100.00

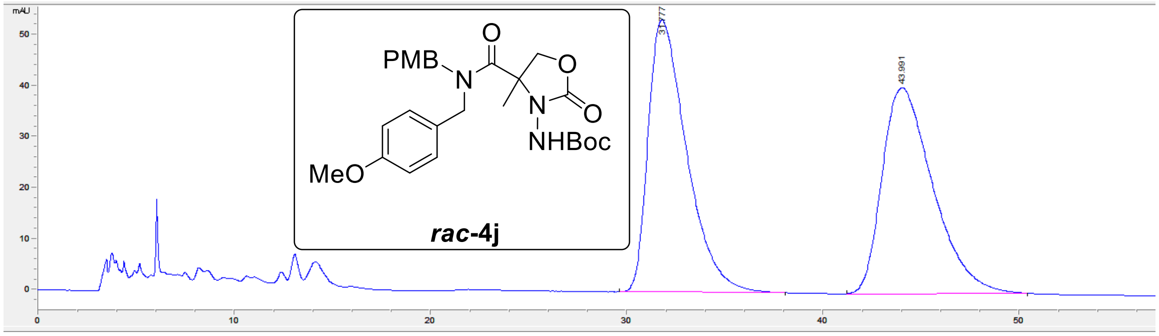

Signal: wavelength = 254 nm

No.	Ret. Time	Area%
1	31.777	49.76
2	43.991	50.24
Total		100.00

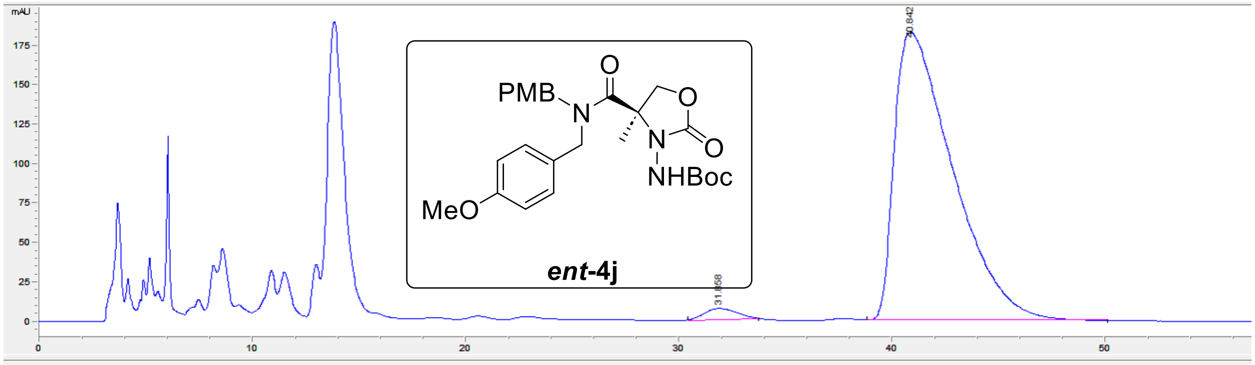

Signal: wavelength = 254 nm

No.	Ret. Time	Area%
1	31.858	2.16
2	40.842	97.84
Total		100.00

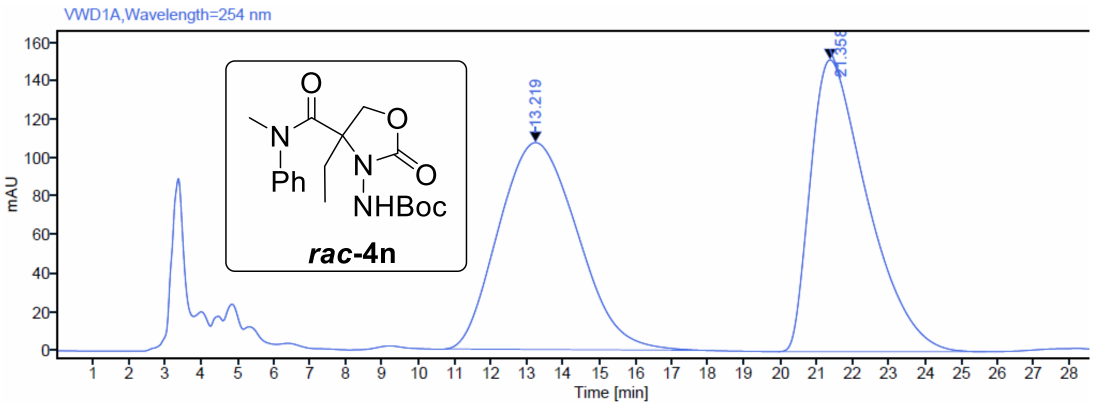

Signal: wavelength = 254 nm

No.	Ret. Time	Area%
1	13.219	49.48
2	21.358	50.52
Total		100.00

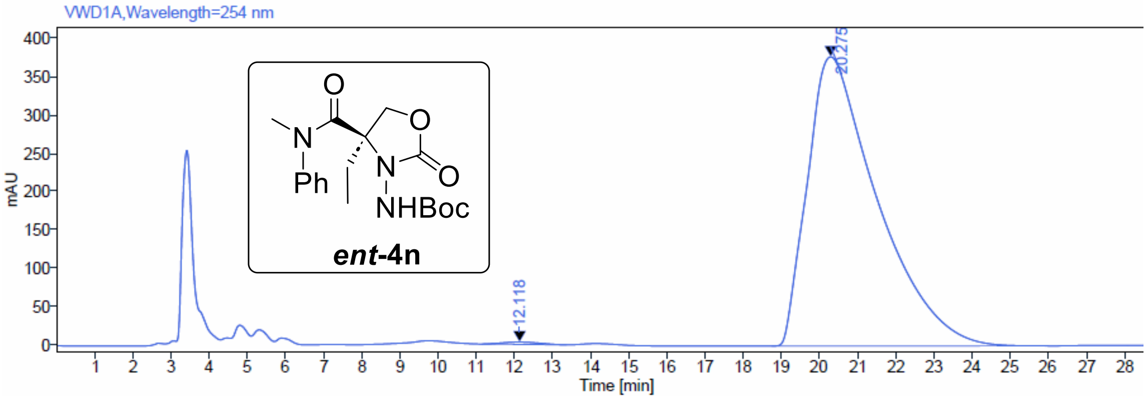

Signal: wavelength = 254 nm

No.	Ret. Time	Area%
1	12.118	0.43
2	20.275	99.57
Total		100.00

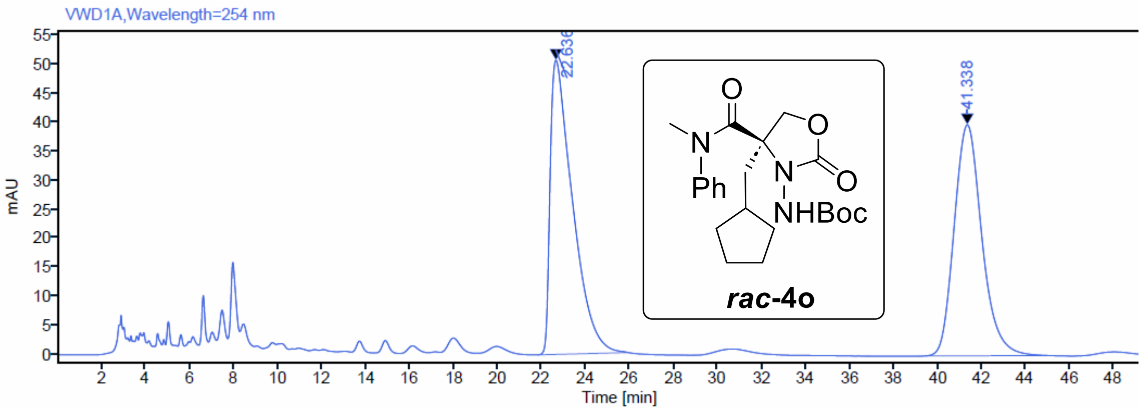

Signal: wavelength = 254 nm

No.	Ret. Time	Area %
1	22.636	50.69
2	41.338	49.31
Total		100.00

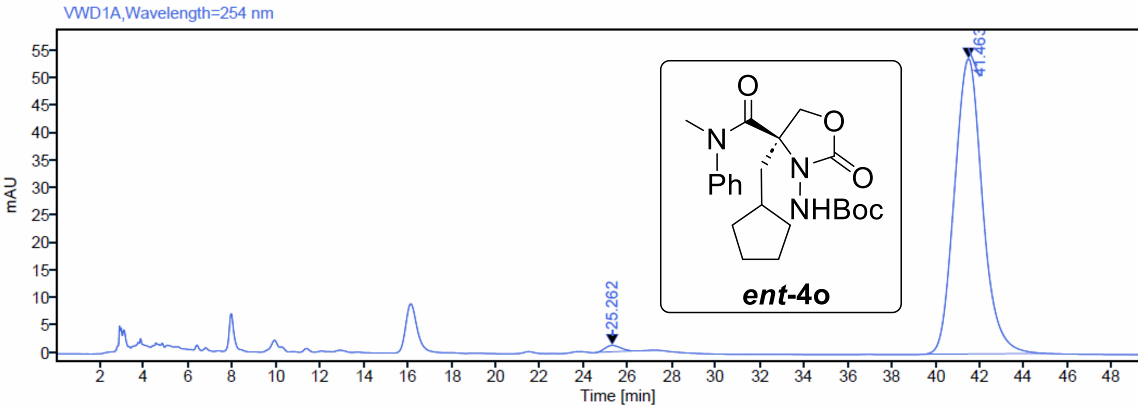

Signal: wavelength = 254 nm

No.	Ret. Time	Area%
1	25.262	1.17
2	41.463	98.83
Total		100.00

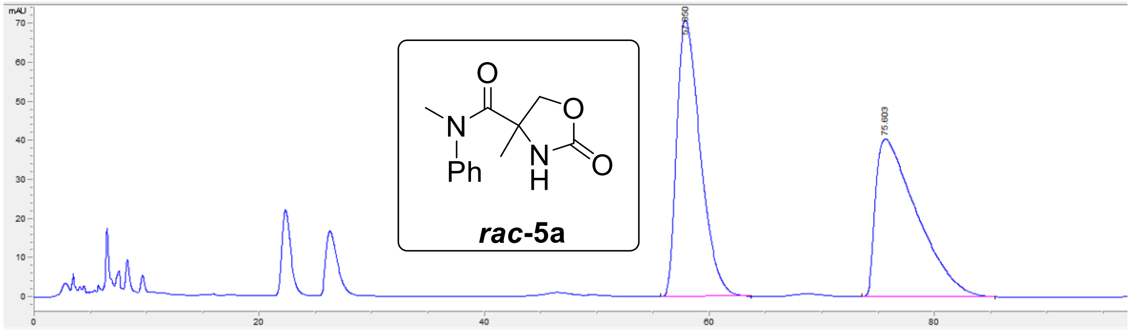

Signal: wavelength = 254 nm

No.	Ret. Time	Area%
1	57.850	50.03
2	75.603	49.97
Total		100.00

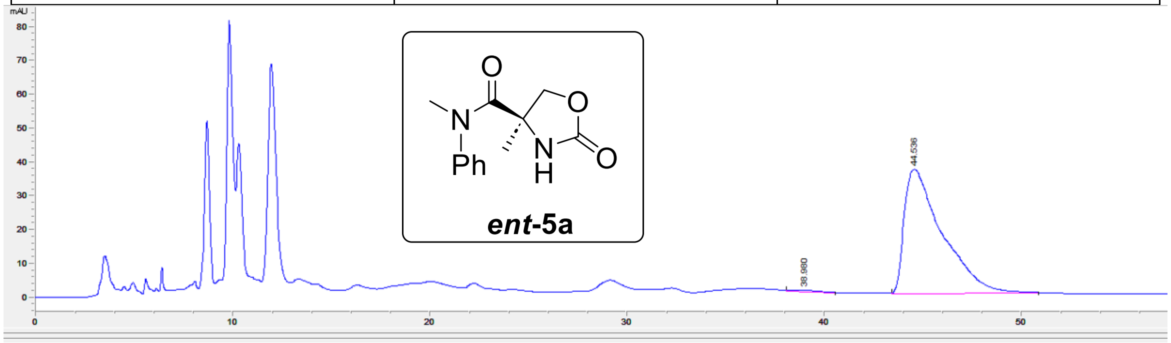

Signal: wavelength = 254 nm

No.	Ret. Time	Area%
1	38.980	1.01
2	44.536	98.99
Total		100.00

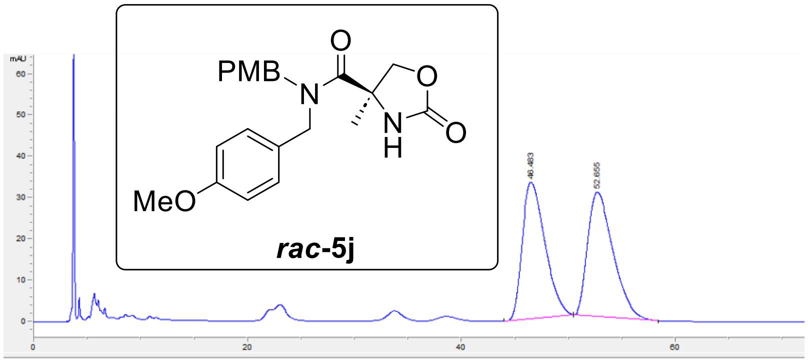

Signal: wavelength = 254 nm

No.	Ret. Time	Area%
1	46.483	49.72
2	52.655	50.28
Total		100.00

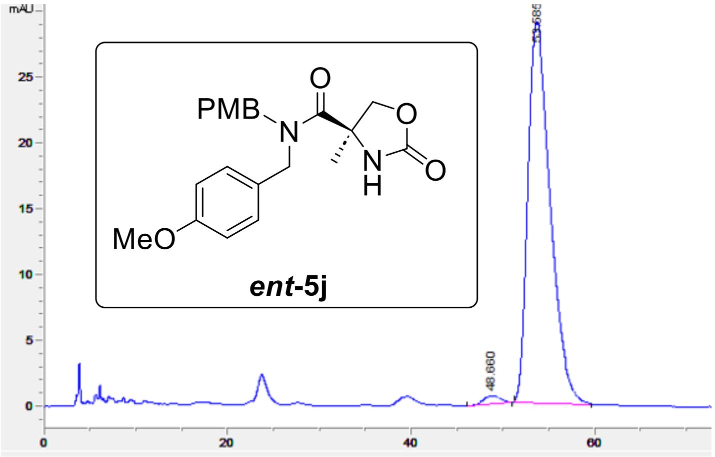

Signal: wavelength = 254 nm

No.	Ret. Time	Area%
1	48.660	2.15
2	53.585	97.85
Total		100.00

END
